# Accelerating Drug Discovery by Early Protein Drug Target Prediction Based on a Multi-Fingerprint Similarity Search †

**DOI:** 10.3390/molecules24122233

**Published:** 2019-06-14

**Authors:** Michele Montaruli, Domenico Alberga, Fulvio Ciriaco, Daniela Trisciuzzi, Anna Rita Tondo, Giuseppe Felice Mangiatordi, Orazio Nicolotti

**Affiliations:** 1 Dipartimento di Farmacia—Scienze del Farmaco, Università degli Studi di Bari “Aldo Moro”, via E. Orabona, 4, I-70125 Bari, Italy; michele.montaruli@gmail.com (M.M.); daniela.trisciuzzi@uniba.it (D.T.); 2 Cineca, Via Magnanelli 6/3, 40033 Casalecchio di Reno, Bologna, Italy; d.alberga@cineca.it; 3 Dipartimento di Chimica, Universitaà degli Studi di Bari “Aldo Moro”, via E. Orabona, 4, I-70125 Bari, Italy; fulvio.ciriaco@uniba.it; 4 Istituto di Ricerche Farmacologiche Mario Negri IRCCS, Via la Masa 19, 20156 Milano, Italy; annarita.tondo@gmail.com; 5 Istituto di Cristallografia, Consiglio Nazionale delle Ricerche, Via G. Amendola 122/O, 70126 Bari, Italy; giuseppe.mangiatordi@ic.cnr.it

**Keywords:** molecular similarity, multi-fingerprint, data quality, protein drug target prediction

## Abstract

In this continuing work, we have updated our recently proposed Multi-fingerprint Similarity Search algorithm (MuSSel) by enabling the generation of dominant ionized species at a physiological pH and the exploration of a larger data domain, which included more than half a million high-quality small molecules extracted from the latest release of ChEMBL (version 24.1, at the time of writing). Provided with a high biological assay confidence score, these selected compounds explored up to 2822 protein drug targets. To improve the data accuracy, samples marked as prodrugs or with equivocal biological annotations were not considered. Notably, MuSSel performances were overall improved by using an object-relational database management system based on PostgreSQL. In order to challenge the real effectiveness of MuSSel in predicting relevant therapeutic drug targets, we analyzed a pool of 36 external bioactive compounds published in the Journal of Medicinal Chemistry from October to December 2018. This study demonstrates that the use of highly curated chemical and biological experimental data on one side, and a powerful multi-fingerprint search algorithm on the other, can be of the utmost importance in addressing the fate of newly conceived small molecules, by strongly reducing the attrition of early phases of drug discovery programs.

## 1. Introduction

Conceiving a drug to bias a specific target is a challenging and risky bet. As is well known, drug discovery often ends in costly flops, with about 12 years being necessary to obtain a marketable drug, and research and development investments often exceeding US$ 1 billion. It goes without saying that reducing attrition in early development is by far more important than filling a pipeline with poorly chosen late-stage products, which are likely to fail and fail expensively [1]. The fiasco in the clinic is often due to sloppy early target validation as drugs do not work or are unsafe. More often than not, the key to success is the quick and accurate identification of drug targets with real-life potential.

In this scenario, we have recently developed a Multi-fingerprint Similarity Search algorithm (MuSSel) in an attempt to better approach protein drug target and bioactivity prediction [2]. By exploiting a large collection of high-quality experimental bioactivity data available from ChEMBL (version 22.1), our first release of MuSSel made use of a pool of 13 selected molecular fingerprints (*FPs*) to return an informed prediction of therapeutically relevant protein drug targets based on a consensus scheme for a given user query. In addition, MuSSel was also effective in mining ChEMBL data relevant to bioactivity prediction and, more specifically, to quantitatively assess the *K_i_* or *IC*_50_ values provided that a relevant similarity threshold was found and that activity cliffs were not experienced. The interested reader is refereed elsewhere [2] for a comprehensive description of the MuSSel architecture.

In this continuing work, our efforts were mainly directed to improving the selection of ChEMBL experimental data in an attempt to increase the overall reliability of the MuSSel results as far as protein target prediction is concerned. Building on this idea, we used the latest release of ChEMBL (version 24.1, at the time of writing) [3] as a training set, which encompassed a wider data landscape (i.e., 862,311 biologically-annotated records) compared to the previous version of ChEMBL (version 22.1) (694,532 biologically-annotated records) [4]. On the other hand, we exploited a high number of upstream molecular options to raise the level of data curation of our training set, which, for instance, applied salt stripping and SMILES standardization [5]. Great attention was then paid to ionizable compounds; these included about 250,000 compounds, thus amounting to half of the data taken from the latest release of ChEMBL (version 24.1, at the time of writing). In the present work, we generated the dominant state for each ionizable compound at pH 7.4 [6], thus allowing the user the chance to gain more realistic results after a screening campaign. For the sake of completeness, a parallel investigation was also carried out to prove the effectiveness of the 13 selected *FPs* in discerning neutral from ionized pairs. Interestingly, we observed that five out of 13 *FPs* returned similarity values that were likely to be pH dependent. The prediction power of this refined version of MuSSel was challenged by employing a more severe validation strategy, resulting in encouraging results with a significant improvement compared to our initial approach. Moreover, the predictive strength of this revised version of MuSSel was further and successfully tested on an external set of 36 properly selected bioactive drug-like compounds published in the Journal of Medicinal Chemistry in the previous three months (from October to December 2018) and thus not included in the latest release of ChEMBL (version 24.1, at the time of writing). Interestingly, we observed that MuSSel returned reliable results, being able to properly predict the reported protein drug target for 18 out of 36 bioactive drug-like compounds. This retrospective exercise gave us the valuable chance to infer some general predictive trends and, more importantly, to gain a wealth of preliminary information about some specific therapeutic classes [7]. The main aim of this study is to describe an advanced drug discovery tool, which relates newly designed small drug-like molecules to the most probable protein drug targets and unveils new potentially clinical uses for known drugs for apparently unrelated diseases.

## 2. Results and Discussion

### 2.1. A Multi-Fingerprints Similarity Analysis Comparing Ionized and Neutral Molecular Pairs

Based on our previous works [2,8], 13 different types of *FPs* were calculated by means of the RDKit [9] and Pybel [10] python packages and the CDK Java library [11,12]. The calculated *FPs* are summarized in Table 1.

**Figure 1:**
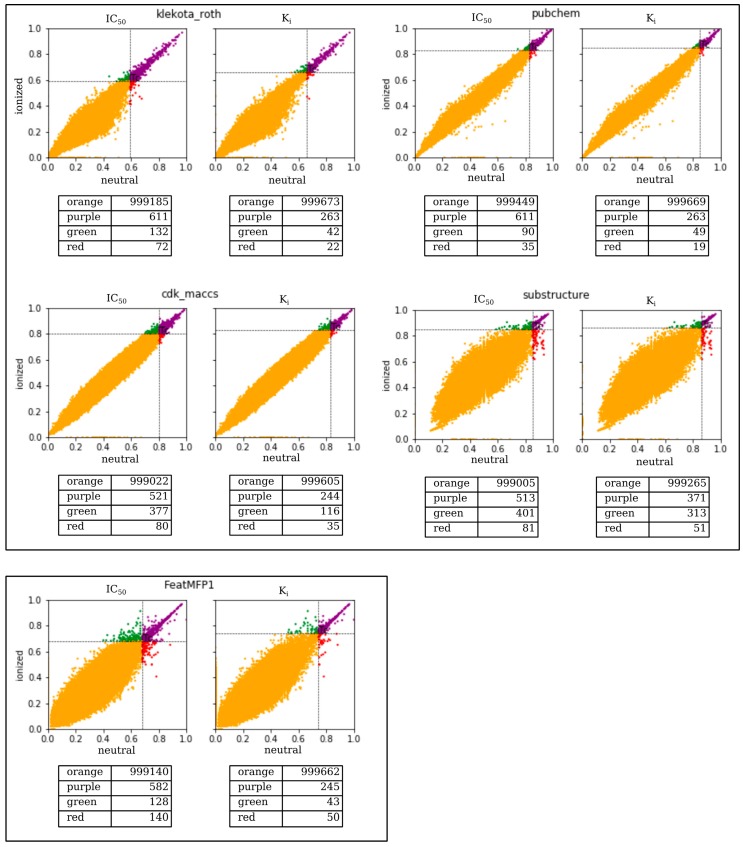
Similarity comparisons of one million neutral vs. ionized pairs of compounds by using *klekota_roth*, *cdk_maccs*, *pubchem* and *substructure*, and *FeatMFP1 FPs*. Orange/purple pairs have similarity values always under/over the threshold, respectively, irrespective of the ionization state. Green/red pairs have similarity values awarded/penalized after ionization, respectively.

As recently explained elsewhere [2,19], these *FPs* were properly selected after conducting a correlation analysis of the Tanimoto similarity coefficients (*Tc*) calculated for one million pairs that were randomly selected. To make a fair comparison of the above selected 13 *FPs*, we randomly generated 10 million pairs according to the approach suggested by Maggiora et al. [20]. In this respect, we calculated the *Tc* distributions of the 13 different *FPs* to designate a statistically significant similarity threshold *T_cm%_*, which indicated, for each considered *FP*, the value of *Tc*, which met or exceeded the percentage of comparison *m*%. For a more comprehensive view, the interested reader is referred to our recent work [2]. We then carried out a preliminary investigation to assess the sensitivity of the 13 selected *FPs* when dealing with ionized compounds at a physiological pH compared to the corresponding neutral species. To this end, we defined two groups containing the same pool of one million pairs of compounds that were first ionized at a physiological pH and in then in a neutral condition, irrespective of pH. This pool of one million pairs of compounds was obtained by random generation from the ensemble of about 250,000 ionizable entries taken from ChEMBL (version 24.1) and then made available in MuSSel. For each pair, the molecular similarity was measured considering that the partners were both ionized on one side and neutral on the other. These similarity measures were thus repeated by using all the 13 *FPs* implemented in MuSSel. Of course, identical similarity values were expected for those *FPs* unable to discern a given pair where the partners were both ionized or both neutral. Likewise, different similarity values should occur in the case of *FPs* distinguishing a given pair if the partners are both charged or both neutral. Based on this idea, we investigated the similarity values calculated by using the 13 *FPs* implemented in MuSSel for the same pool of one million pairs existing as ionized and neutral forms. Interestingly, our analysis revealed that a pH-dependent similarity was found in five out of the 13 *FPs*, including *klekota_roth*, *cdk_maccs*, *pubchem*, *substructure*, and *FeatMFP1*. For the sake of clarity, we plotted their calculated similarity values in the case of ionized (i.e., y axis) and neutral (i.e., x axis) partners for each pair of compounds randomly generated. As shown in Figure 1, each graph can be split into four areas. The first and the second areas collected pairs, colored in orange and purple, respectively, whose similarity values were always under and over their calculated statistically significant similarity threshold [2,20], irrespective of the ionization state. The third area collected pairs colored in green whose similarity values exceeded their calculated statistically significant similarity threshold, having been awarded in terms of molecular similarity on the basis of their ionized state. Finally, the fourth area collected pairs colored in red whose similarity values deteriorated following the ionization. Bearing this in mind, we could observe that *klekota_roth*, *cdk_maccs*, *pubchem*, and *substructure FPs* tended to move pairs towards the green rather than red areas. This could likely indicate that such *FPs* could have a major role in dealing with ionized pairs. On the other hand, a higher number of pairs was in the red zone when using the *FeatMFP1*, likely suggesting that this *FP*, although pH-dependent, was not very successful at screening ionized queries. For the sake of comparison, the interested reader can find the same graph generated for all the other remaining 8 *FPs* in Figure S1.

For the sake of completeness, we also assessed the overall effect of ionization on predictions. To this end, we used two copies of our entire MuSSel database. The first contained all the compounds in a neutral state and the second contained compounds as ionized species depending on the physiological pH. These two copies were thus used to predict, separately for both *K_i_* and *IC*_50_ pools, an external set made of 5000 compounds ionized at a physiological pH extracted from the relative pool. As illustrated in Table 2, the results show that the statistics are slightly improved in the case of predictions based on the ionized database. Although the improvements are small, the results reflect a more realistic picture based on the effect of a physiological pH on ionizable species.

### 2.2. K_i_ and IC_50_ based Protein Drug Target Predictions

In the present investigation, our attention was mostly directed to assessing the impact on the prediction of ionized dominant species calculated for a larger basis of data (that is about 862,311 vs. 694,532 of our previous analysis) provided with a higher biological assay confidence score. The same setting of calibration parameters already tuned in our previous work was used [2]. For the ease of comparison, we preferred to first predict the same three external sets discussed in our previous work. Basically, a prediction was flagged as correct if a match was found as the top-one (that is *p*_1_) or within the top-five (that is *p*_5_) calculated protein drug targets after selecting, by chance, one experimental biological annotation for each external set compound. In this respect, each of these three external sets contained 300 compounds that were randomly selected considering the difference between ChEMBL (version 23) and ChEMBL (version 22.1) [21,22,23]. Of course, these external set compounds were excluded from our new training set before their prediction. However, due to this difference in the collection of congeneric series recently published in the scientific literature, this widely employed validation strategy could not ensure that these external sets could really reflect the same proportion of protein drug targets existing in the training data set. In this respect, the usage of the latest release of ChEMBL (version 24.1, at the time of writing), which encompassed the previous ones, could have had the effect of resulting in an impressive improvement of statistics for both the *K_i_* and *IC*_50_ pools in comparison with our previous work.

To avoid the above mentioned risk of misrepresentation of data in the external sets, we herein carried out a further validation analysis based on the prediction of a pool of 1000 compounds blindly extracted from the latest release of ChEMBL (version 24.1, at the time of writing) and left out from our new training set. In addition, we also considered the chance of having multiple experimental biological annotations for a ligand. To deal with this more complex but indeed more realistic description of data, a prediction was herein considered successful if a match was found as the top-one (that is *p*_1_) or within the top-five (that is *p*_5_) calculated protein drug targets after scanning all the available and experimentally measured biological annotations. Based on this counting approach, this new validation test returned encouraging statistics, reported in Table 3, with top-one and top-five protein drug targets ranging from 90.77% to 94.32% in the case of *K_i_* and from 90.1% to 93.2% in the case of *IC*_50_, respectively. The interested reader can inspect all the external set data by browsing the content of the File S1 enclosed as Supplementary Materials.

### 2.3. Case Studies

The real predictive strength of MuSSel was finally challenged by conducting a retrospective exercise on a pool of drug-like small molecules whose experimentally determined protein drug targets have just been published in the Journal of Medicinal Chemistry and are therefore not yet covered in the latest release of ChEMBL (version 24.1, at the time of writing). More specifically, we only selected research papers published from October to December of the year 2018 including specific keywords (that were one of the following: discovery, synthesis, identification, design, or optimization) in the title and provided with SMILES notations. The selection was further limited to only small molecules, apart from radioligands, having experimentally established information and whose protein drug target was included among the 2822 explored by MuSSel. In this way, we collected 36 small molecules whose chemical structures were thus submitted to MuSSel to test its potential in properly pairing the real protein drug targets. We observed that the target of 18 small molecules out of 36 was properly ranked in the top-five and, very satisfactorily, in 16 out of 18 compounds, the right target was the top-one. For the sake of comparison, our previous MuSSel release was able to correctly match 15 out of 36 protein drug targets. A comprehensive view of the chemical structures of the 18 entries whose protein drug targets were successfully predicted is given in Table 4. A closer look revealed that MuSSel was effective in associating the real and predicted protein drug target, apart from compounds **2** and **10**. For the former, the Heat shock protein 90 kDa beta member 1 was predicted in place of its real isoform, which is actually the Heat shock protein 90 alpha [24], while for the latter, the fibroblast growth factor receptor 1 was predicted in place of its real isoform, which is actually the fibroblast growth factor receptor 2 [25]. The same analysis was carried out for unsuccessfully predicted protein drug targets and a comprehensive list is enclosed as Table S1. By analyzing the number of entries per targets selected in MuSSel, we observed that properly predicted targets were significantly more populated than those unsuccessfully predicted. For the sake of completeness, the same pool of 36 small molecules was also challenged by using the SwissTargetPrediction [26] and the Polypharmacology Browser 2 webserver [27]. Interestingly, a nice overlap was observed by comparing the results obtained by MuSSel with those of the other two platforms. More specifically, MuSSel, SwissTargetPrediction, and Polypharmacology Browser 2 were successful in properly matching the actual protein drug target of 18, 12, and 13 out of 36 cases, respectively. Interestingly, the combined use of the three platforms had the effect of increasing the overall accuracy to 21 out of 36 initial queries, as described in Table S2 of the Supplementary Materials. The approach herein adopted was based on a scheme implying that a given protein drug target, reported in the articles published by the Journal of Medicinal Chemistry, was successfully predicted if at least one of the three platforms returned the right answer [28]. For a more informed view, the interested reader is referred to the Supplementary Materials enclosed in the File S2, which also contains full, detailed reports provided by SwissTargetPrediction and by the Polypharmacology Browser 2 webserver.

## 3. Materials and Methods

### 3.1. Construction of the Ki and IC_50_ Database

ChEMBL (version 24.1) was downloaded as a PostgreSql cartridge format and stored in a local machine. MuSSel-related tables were associated with three ChEMBL macroareas: ‘COMPOUND INFORMATION’ (tables *molecule_dictionary* and *compound_records*); ‘EXPERIMENTAL DATA’ (tables *activities* and *assays*); and ‘TARGET INFORMATION’ (tables *target_dictionary*, *target_components*, *component_sequences*, *component_class*, and *protein_classification*). As is usual in relational databases, the table associations were based on links between primary keys and foreign keys, as described in ChEMBL [45]. A materialized view named *MuSSel_data* was thus built by querying the ChEMBL database for retrieving entries matching the following criteria: only ‘*small molecule*’ in the *molecule_type* field was chosen; molecules marked in the database as prodrugs were removed (about 300 records); a target was set if the *target_type* was indicated as ‘*SINGLE_PROTEIN*’ or ‘*PROTEIN COMPLEX*’ no restriction was applied on *organism* to avoid missing therapeutically-relevant targets, such as those typical of antiviral and antibiotic drugs; all records containing any warning on the data assay were removed (*data_validity_comment* field); only records with an assay *confidence_score* greater than 5 were considered on a scale from 1 to 9; only activity records containing ‘*IC50*’, ‘*Ki*’, ‘*Kd*’, and ‘*EC50*’ as *standard_type* were maintained; and ambiguous biological data with *standard_relation* equal to ‘>’ in the *activity* table were removed. Based on this filtering process, 1,280,553 records were selected for MuSSel. The whole pool of *MuSSel_data* was split into four main groups by *standard_type* activity values. The interested reader can find the SQL query used for *MuSSel_data* generation in Figure S2 of the Supplementary Materials and a formal scheme as Figure S3 of the Supplementary Materials. In this study, only *IC*_50_ and *K_i_* groups were analyzed according to our previous investigation [2]. Within each group, the records were split into protein drug target ensembles contacting at least 10 compounds or data were otherwise not further considered. In the case of multiple ligand annotations within the same protein drug target, the lowest activity value (i.e., the best measure for *K_i_* or *IC*_50_) was retained [2]. The *K_i_* pool contained 288,178 annotated entries covering 1265 targets, while the *IC*_50_ pool included 522,594 covering 2441 targets. The interested reader is referred to the File S3 of the Supplementary Materials for a complete list of the protein drug targets explored in MuSSel.

### 3.2. Canonicalization and Correction of Chemical Structures

Validation control was performed in a canonical SMILES format stored in *MuSSel_data*, using an in-house python script based on RDkit and the MolVS library [9]. The main steps were the fragment strip and the standardization. The fragment strip (also termed as salt strip) was based on the identification of all the fragments in SMILES strings in order to maintain only the largest one. In the case of enantiomers, only one was randomly picked. The standardization implied a sequence of structure optimization steps consisting of SMILES sanitization (i.e., valence error correction), metal disconnection, the application of normalization rules, and stereochemistry recalculation [46,47].

### 3.3. Generation of Dominant Ionized Species at a Physiological pH and Neutral Forms

Compounds were ionized at pH 7.4 by using the ChemAxon *cxcalc majormicrospecies* plugin [48]. Neutral forms were regenerated from standardized SMILES using the Rdkit package [9].

### 3.4. Fingerprints Generation

For each molecule included in the *MuSSel_data* table, 13 different types of *FPs* were calculated by means of the RDKit [9], Pybel [10], and CDK [11] packages and loaded as tables in the MuSSel database. The calculated *FPs* are summarized in Table 1. Two parallel sets of *FPs* were generated when a molecule could exist in the ionized and neutral form based on the physiological pH of 7.4. In this respect, a first set of 246,959 *FPs* was generated in the neutral form and a second parallel set of 509,058 *FPs* was derived to account for the possible ionization states. This heavy computational task was carried out by using the ReCaS-Bari system [49].

### 3.5. Construction of the External Sets

Based on the same procedures described in the previous paragraphs, three external sets were generated using the ionized SMILES strings exploiting the same external sets already challenged in our previous work and a corresponding set of *FPs* was computed [2]. Each external set contained 300 compounds that were randomly selected considering the difference between ChEMBL (version 23) and ChEMBL (version 22.1). The compounds of each external set were excluded by *MuSSel_data* before their prediction. In addition, an external set of 1000 compounds and one made of 5000 ionized compounds were randomly extracted from both the *K_i_* and *IC*_50_ pools and left out from our training set before their prediction. All the external sets are included in the File S1 enclosed as Supplementary Materials.

### 3.6. Selection of Prospective Queries From Recently Published Scientific Articles

A systematic PubMed search of scientific articles recently published in the Journal of Medicinal covering the period from October to December 2018 was performed. Papers containing at least one of the following keywords: *discovery*, *synthesis*, *identification*, *design* or *optimization*, in the titles and provided with SMILES notations in the related Supporting Information were considered. For the sake of completeness, we only selected those small molecules, apart from radioligands, having experimentally established information and whose protein drug targets were included in the MuSSel database. Finally, a pool of 36 queries was generated. Additional details are reported in Table 4 and in Table S1.

### 3.7. Protein Drug Target Multi-FPs Similarity Search Algorithm

The protein drug target multi-*FPs* similarity search algorithm returned an overall score on the basis of the similarity values measured by using the pool of selected *FPs* listed in Table 1. More specifically, the *Tc* value between the query compound and all the entries associated with each protein drug target available in MuSSel was calculated by using each of the 13 *FPs*. A given query was paired to a protein drug target if there was at least one entry having a *Tc* no lower than the pre-calculated similarity thresholds *Tc_m%_* for a minimum number of *FPs*. If this condition held true, a score (*SC*) was thus measured for the protein drug target:

$$SC=\overset{13}{\underset{\begin{array}{c} i=1\\ Tc_{i}^{max}\geq Tc_{m\%}^{T} \end{array}}{\sum }}Tc_{i}^{max}$$

where $Tc_{i}^{max}$ was the maximum *Tc* value, based on the *i-th FP* type, between the query and the molecules associated with the drug target provided that $Tc\geq Tc_{m\%}^{T}$. Finally, the selected protein drug targets were ranked according to the assigned *SC* values. For additional details, the interested reader is referred to our previous work [2].

## 4. Conclusions

In this study, we improved our multi-*FPs* similarity search algorithm, named MuSSel, by considering a wider and even higher quality pool of about half a million small drug-like molecules extracted by the latest release of ChEMBL (version 24.1, at the time of writing). This refined larger pool included biological data not limited to *Homo Sapiens* as the organism and covered 2440 experimentally determined biological targets. In addition, the multi-*FPs* similarity search was carried out considering the calculated dominant ionized species at a physiological pH for each small molecule taken from ChEMBL. Compared to our previous investigation, the net effect of these changes was a significant improvement of the external set statistics. Interestingly, this trend was substantially confirmed by a new second validation set and even by the retrospective exercise based on a pool of 36 selected molecules just published in the Journal of Medicinal Chemistry. In this work, we have launched a private platform, accessible on demand, which allows the interested users to screen single or even multiple queries at a time, as normally requested in reverse screening campaigns, which have become an integral part of drug discovery pipelines [50]. The herein proposed method showed very promising performances and can offer a useful and easy-to-run tool capable of pairing novel compounds to putative protein drug targets, as well as repurposing known drugs to apparently unrelated diseases, explicitly accounting for their potential toxicity and/or unwanted side effects. It is noteworthy that the multi-fingerprint search algorithm also demonstrated a great potential for the prediction of acute oral toxicity [51].

## Figures and Tables

**Table 1:** Fingerprint notations along with the open-source software packages used for their calculation.

Fingerprints Name	Description	Package	Reference
*MFP1*	Morgan connectivity invariants (*ECFP*-like) with radius = 1	RDKit	[13]
*FeatMFP1*	Morgan feature invariants (*FCFP*-like) with radius = 1	RDKit	[13,14]
*AP_bits*	Atom pairs fingerprint	RDKit	[15]
*Pattern*	SMARTS Pattern fingerprint	RDKit	[9]
*RDKit7*	Daylight-like topological fingerprint	RDKit	[9]
*TT_bits*	Topological torsion fingerprint	RDKit	[16]
*FP2*	Indexes linear fragments up to 7 atoms	Pybel	[10]
*pubchem*	Pubchem fingerprints	CDK	[17]
*cdk_maccs*	*MACCS* fingerprint that generates 166-bit *MACCS* keys	CDK	[11,12]
*klekota_roth*	*Klekota-Roth* fingerprints based on 4860 substructures	CDK	[18]
*graph*	Graph fingerprint which does not take bond orders into account	CDK	[11,12]
*substructure*	Bit set type fingerprint based on 307 substructures	CDK	[11,12]
*hybridization*	Fingerprint based on hybridization state of atoms	CDK	[11,12]

**Table 2:** For both the *K_i_* and *IC*_50_ pools, predictions are based on first using the neutral database and then the ionized database on the same external dataset comprised of 5000 ionized compounds at a physiological pH randomly discarded by the training set based on ChEMBL (version 24.1). Using both *K_i_* and *IC*_50_ protein drug target data, the predictions were considered successful if a match was found as the top-one (*p*_1_) or within the top-five (*p*_5_).

	*Ki MuSSel Data* ^1^		*IC50 MuSSel Data* ^1^	
	*p*_1_	*p*_5_	*p*_1_	*p*_5_
Neutral database	89.72%	92.82%	86.80%	90.20%
Ionized database	91.08%	93.16%	88.72%	92.24%

^1^ The calibration parameters were kept unchanged, as in our previous study [2].

**Table 3:** Each of Ext1, Ext2, and Ext3 comprised 300 molecules randomly taken from the difference between ChEMBL (version 23) and ChEMBL (version 22.1). Ext4 comprised 1000 compounds randomly discarded from the training set based on ChEMBL (version 24.1). Using both *K_i_* and *IC*_50_ protein drug target data, the predictions were considered successful if a match was found as the top-one (*p*_1_) or within the top-five (*p*_5_) targets.

	*Ki MuSSel Data* ^1^		*IC50 MuSSel Data* ^1^	
	*p*_1_	*p*_5_	*p*_1_	*p*_5_
Ext1 (*n* = 300)	90.67%	96.00% (56.20%) *	88.00%	93.33% (35.00%) *
Ext2 (*n* = 300)	90.33%	96.00% (48.60%) *	92.00%	95.00% (31.70%) *
Ext3 (*n* = 300)	93.67%	97.33% (51.40%) *	89.33%	92.00% (29.30%) *
Ext4 (*n* = 1000)	90.77%	94.32%	90.10%	93.20%

^1^ The calibration parameters were kept unchanged, as in our previous study [2]. * For the ease comparison, the *p5* values obtained in our previous study [2] are reported in parentheses.

**Table 4:** Chemical structures of the 18 entries selected from the Journal of Medicinal Chemistry (i.e., inspecting papers published from October to December 2018) whose protein drug targets were successfully predicted. For each entry, the name of the protein drug target with the corresponding number of associated compounds, as well as the ChEMBL ID available in MuSSel, are reported. A parallel table with the unsuccessful cases is enclosed in the Supplementary Materials.

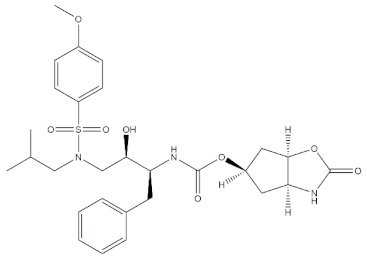	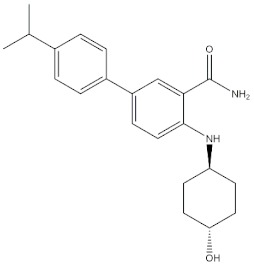	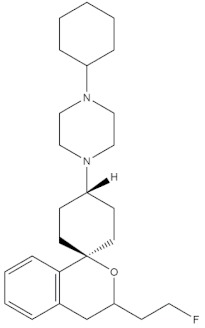
**1** HIV-1 Protease CHEMBL2366517, *n* = 997 [29]	**2** Heat shock protein 90 kDa beta member 1 CHEMBL4303, *n* = 538 [24]	**3** Sigma opioid receptor CHEMBL4153, *n* = 1426 [30]
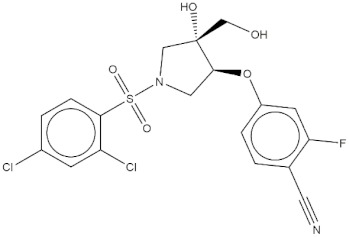	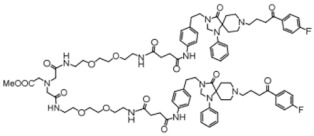	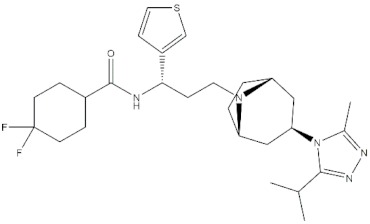
**4** Transient receptor potential cation channel subfamily V 4 CHEMBL3119, *n* = 50 [31]	**5** Dopamine D2 receptor CHEMBL339, *n* = 3934 [32]	**6** CC-Chemokine Receptor 5 CHEMBL274, *n* = 2051 [33]
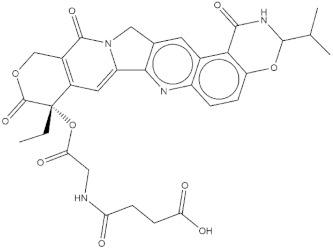	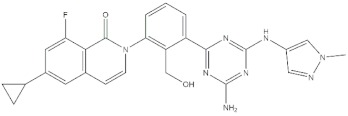	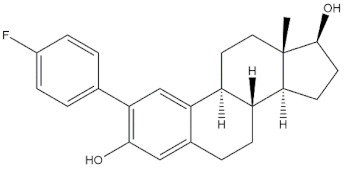
**7** DNA topoisomerase I CHEMBL1781, *n* = 347 [34]	**8** Tyrosine-protein kinase BTK CHEMBL5251, *n* = 808 [35]	**9** Cytochrome P450 (CYP) 1B1 CHEMBL1978, *n* = 1858 [36]
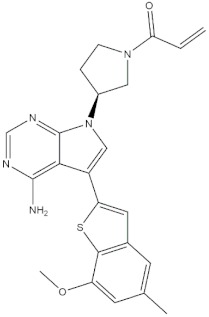	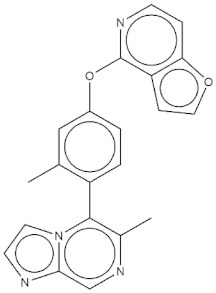	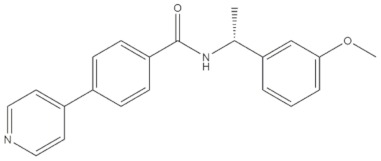
**10** Fibroblast growth factor receptor 1 CHEMBL4142, *n* = 288 [25]	**11** Dopamine D1 receptor CHEMBL2056, *n* = 986 [37]	**12** Rho-associated protein kinase 2 CHEMBL2973, *n* = 1687 [38]
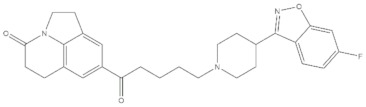	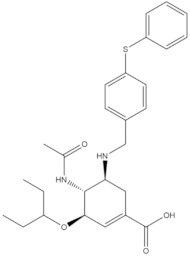	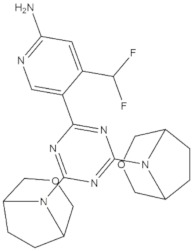
**13** Dopamine D2 receptor CHEMBL339, *n* = 3934 [39]	**14** Neuraminidase - Influenza A virus CHEMBL1667684, *n* = 35 [40]	**15** Serine/threonine-protein kinase mTOR CHEMBL2842, *n* = 3087 [41]
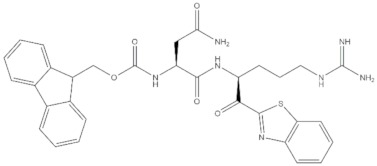	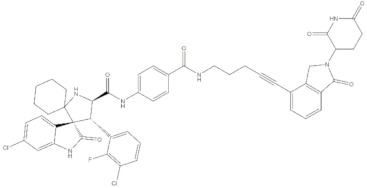	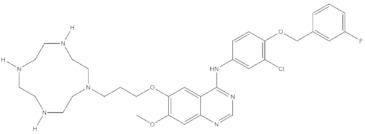
**16** Hepsin serine protease CHEMBL204, *n* = 4774 [42]	**17** p53-binding protein Mdm-2 CHEMBL5023, *n* = 1830 [43]	**18** Epidermal growth factor receptor CHEMBL203, *n* = 5187 [44]

## Supplementary Materials

The supplementary materials are available online.
