# Supplementary material for: Accelerating Drug Discovery by Early Protein Drug Target Prediction Based on a Multi-Fingerprint Similarity Search
Source: Molecules. 2019 Jun 14;24(12):2233. doi: 10.3390/molecules24122233 (PMC6631269; doi:10.3390/molecules24122233)
Supplement: Supplementary file 1 [file molecules-24-02233-s001.zip › Supporting_Materials_Molecules_20_05_2019_ON.docx]

**Supporting Materials**

Accelerating drug discovery by early protein drug target prediction based on multi-fingerprint similarity search

Michele Montaruli^1^, Domenico Alberga^2^, Fulvio Ciriaco^3^, Daniela Trisciuzzi^1^, Anna Rita Tondo^4^, Giuseppe Felice Mangiatordi^5^ and Orazio Nicolotti^1,^*

^1^ Dipartimento di Farmacia—Scienze del Farmaco, Università degli Studi di Bari “Aldo Moro”, via E. Orabona, 4, I-70125 Bari, Italy; michele.montaruli@gmail.com (M.M.); daniela.trisciuzzi@uniba.it (D.T.); orazio.nicolotti@uniba.it (O.N.)

^2^ Cineca, Via Magnanelli 6/3, 40033 Casalecchio di Reno, Bologna, Italy; d.alberga@cineca.it (D.A.)

^3^ Dipartimento di Chimica, Università degli Studi di Bari “Aldo Moro”, via E. Orabona, 4, I-70125 Bari, Italy; fulvio.ciriaco@uniba.it (F.C.)

^4^ Istituto di Ricerche Farmacologiche Mario Negri IRCCS, Via la Masa 19, 20156, Milano, Italy; annarita.tondo@gmail.com (A.T.).

^5^ Istituto di Cristallografia, Consiglio Nazionale delle Ricerche, Via G. Amendola 122/O, 70126 Bari, Italy; giuseppe.mangiatordi@ic.cnr.it (G.M.)

***** Correspondence: orazio.nicolotti@uniba.it; Tel.: +0039-080-5442551 (O.N.)

**Figures Captions**

**Figure S1.** Similarity comparisons of one million ionized *vs* neutral pairs according to *RDKit7, TT_bits, AP_bits, Pattern, hybridization, FP2, MFP1* and *graph FPs.*

**Figure S2.** SQL query used on ChEMBL 24.1 database for retrieving useful MuSSel data records.

**Figure S3.** MuSSel data synoptic table.

**Tables Captions**

**Table S1.** Chemical structures of the 18 entries selected from Journal of Medicinal Chemistry whose protein drug targets were unsuccessfully predicted. For each entry, the name of the protein drug target, the ChEMBL ID and the number of associated compounds in MuSSel are reported.

**Table S2.** Target predictions provided by the three platforms (MuSSel: Multifingerprint Similarity Search algorithm; STP: SwissTargetPrediction; PPB2: Polypharmacology Browser 2).

**Figure S1.**


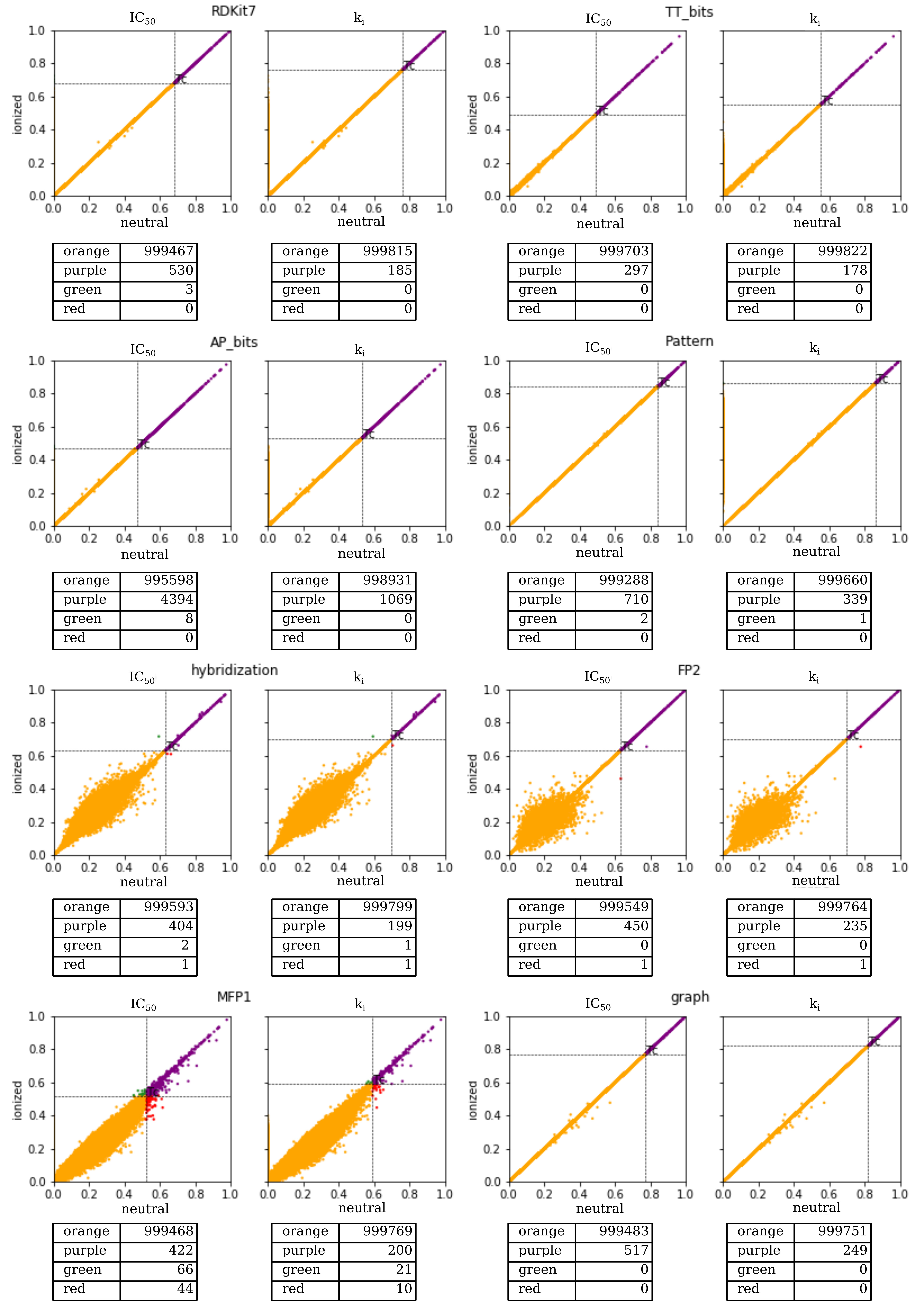


**Figure S2**


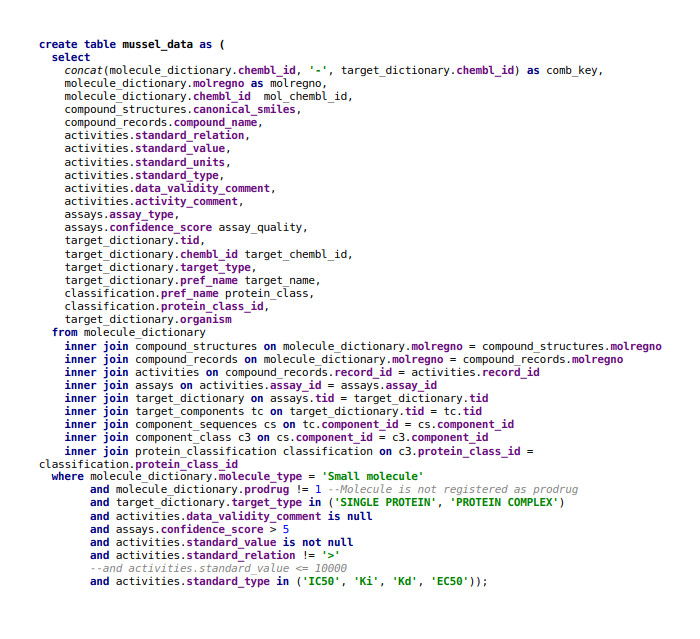


**Figure S3.**


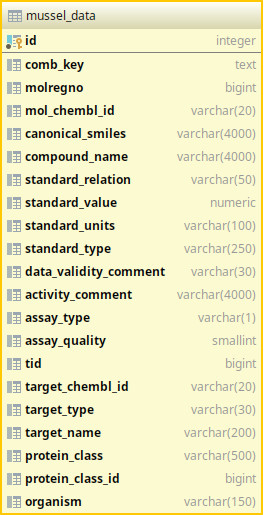


**Table S1.**

| 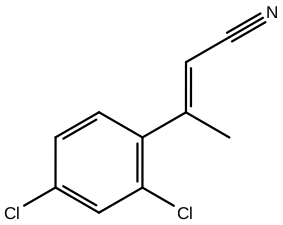 | 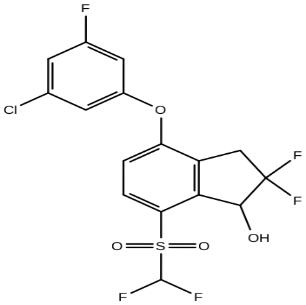 | 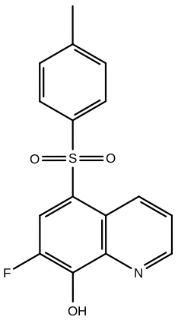 |
| --- | --- | --- |
| 1  GABA_A_R subunit β  CHEMBL3986, n=19 [1] | 2  Hypoxia-inducible factor 2α  CHEMBL1744522, n= 65 [2] | 3  Catechol O-methyltransferase  CHEMBL2023, n= 61 [3] |
| 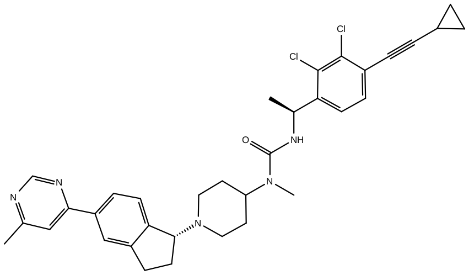 | 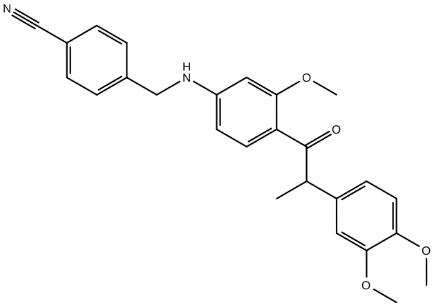 | 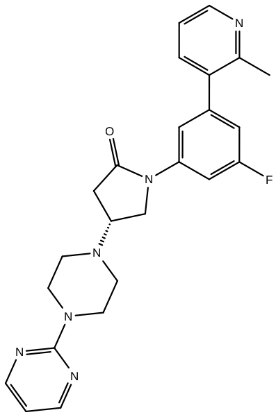 |
| 4 Ghrelin receptor  CHEMBL4616, n= 1341 [4] | 5 Hypoxia-inducible factor 1α  CHEMBL4261, n= 195 [5] | 6 Monoacylglycerol lipase  CHEMBL5774, n= 46 [6] |
| 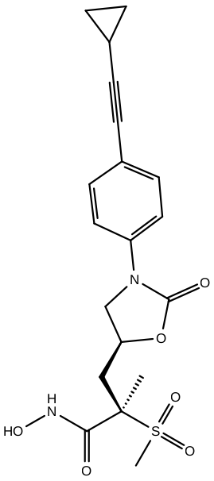 | 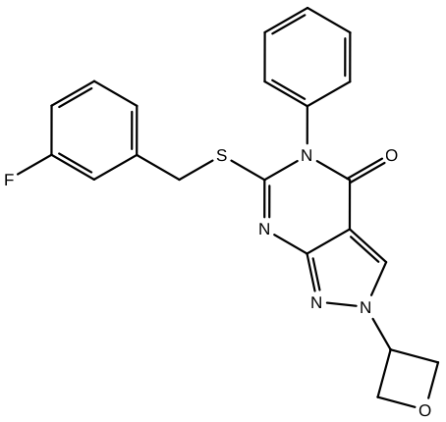 | 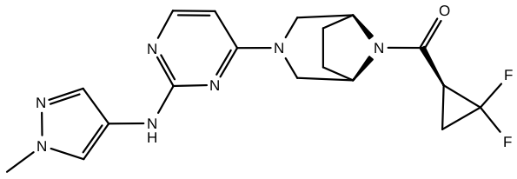 |
| 7  UDP-3-O-acyl-N-acetylglucosamine deacetylase  CHEMBL2163170, n= 23 [7] | 8  Aldehyde dehydrogenase 1A1  CHEMBL3577, n= 46 [8] | 9  Tyrosine-protein kinase TYK2  CHEMBL3553, n= 578 [9] |
| 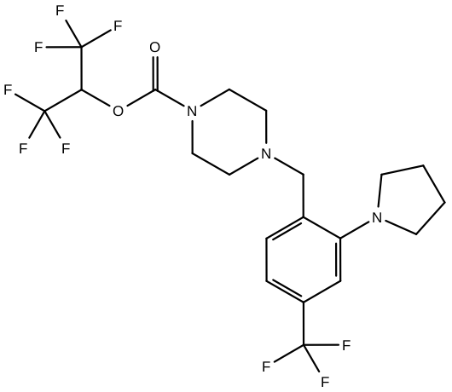 | 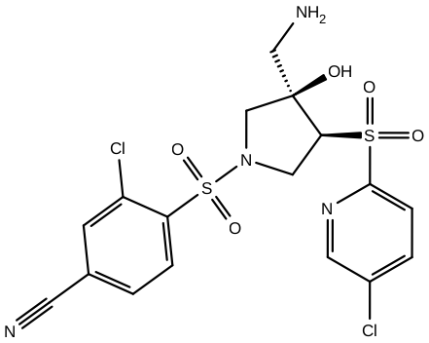 | 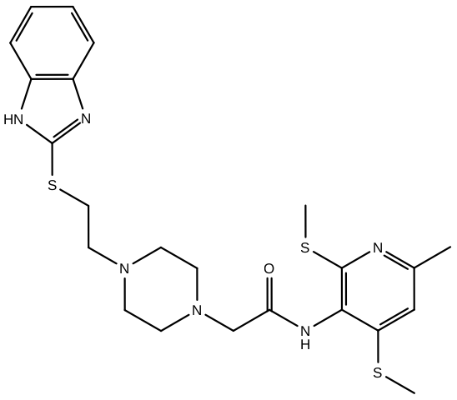 |
| 10  Monoglyceride lipase  CHEMBL4191, n= 343 [10] | 11 Transient receptor potential cation channel subfamily V  CHEMBL3119, n= 50 [11] | 12 Acyl coenzyme A: cholesterol acyltransferase  CHEMBL2265, n= 287 [12] |
| 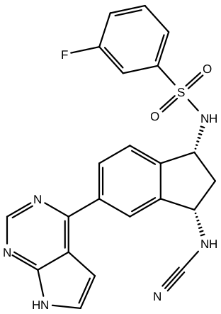 | 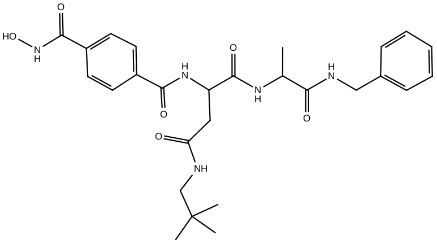 | 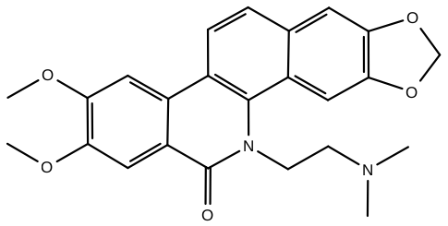 |
| 13  Tyrosine-protein kinase JAK3  CHEMBL2148, n= 2038[13] | 14  Histone deacetylase 6  CHEMBL1865, n= 1865 [14] | 15  DNA topoisomerase IB  CHEMBL1781, n= 347 [15] |
| 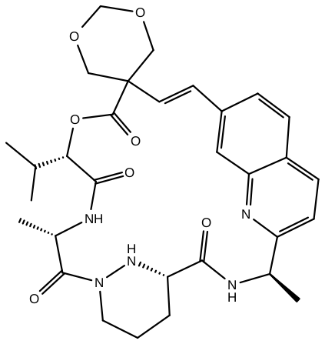 | 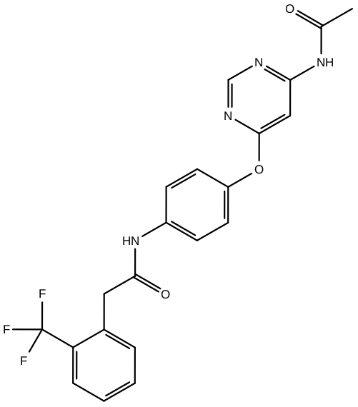 | 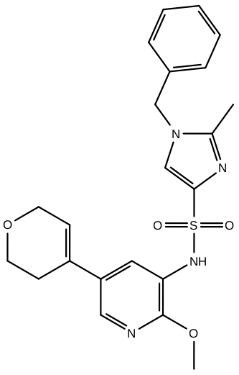 |
| 16 Cyclophilin A  CHEMBL1949, n= 132 [16] | 17  FMS-like tyrosine kinase 3  CHEMBL1974, n=1753 [17] | 18  PI3-kinase p110 subunit δ  CHEMBL3130, n= 1627 [18] |

**Table S2.**

|  | **Target name** | **MuSSel** | **STP** | **PPB2** | **Combined** |
| --- | --- | --- | --- | --- | --- |
| 1 | HIV-1 Protease | YES | NO | NO | YES |
| 2 | Heat shock protein 90 kDa β member 1 | YES | NO | YES | YES |
| 3 | Sigma opioid receptor | YES | YES | YES | YES |
| 4 | Transient receptor potential cation channel subfamily V 4 | YES | NO | NO | YES |
| 5 | Dopamine D2 receptor | YES | YES | YES | YES |
| 6 | CC-Chemokine Receptor 5 | YES | YES | YES | YES |
| 7 | DNA topoisomerase I | YES | NO | YES | YES |
| 8 | Tyrosine-protein kinase BTK | YES | YES | YES | YES |
| 9 | Cytochrome P450 (CYP) 1B1 | YES | NO | NO | YES |
| 10 | Fibroblast growth factor receptor 1 | YES | YES | YES | YES |
| 11 | Dopamine D1 receptor | YES | NO | YES | YES |
| 12 | Rho-associated protein kinase 2 | YES | YES | YES | YES |
| 13 | Dopamine D2 receptor | YES | YES | YES | YES |
| 14 | Neuraminidase - Influenza A virus | YES | NO | NO | YES |
| 15 | Serine/threonine-protein kinase mTOR | YES | YES | YES | YES |
| 16 | Hepsin Serine Protease | YES | NO | NO | YES |
| 17 | p53-binding protein Mdm-2 | YES | YES | NO | YES |
| 18 | Epidermal growth factor receptor | YES | YES | YES | YES |
| 19 | GABAA R subunit β | NO | NO | NO | NO |
| 20 | Hypoxia-inducible factor 2α | NO | NO | NO | NO |
| 21 | Catechol O-methyltransferase | NO | NO | NO | NO |
| 22 | Ghrelin receptor | NO | NO | NO | NO |
| 23 | Hypoxia-inducible factor 1 alpha | NO | NO | NO | NO |
| 24 | Monoacylglycerol lipase | NO | NO | NO | NO |
| 25 | UDP-3-O-acyl-N-acetylglucosamine deacetylase | NO | NO | NO | NO |
| 26 | Aldehyde dehydrogenase 1A1 | NO | NO | NO | NO |
| 27 | Tyrosine-protein kinase TYK2 | NO | NO | NO | NO |
| 28 | Monoglyceride lipase | NO | NO | NO | NO |
| 29 | Transient receptor potential cation channel subfamily V | NO | NO | NO | NO |
| 30 | Acyl coenzyme A: cholesterol acyltransferase | NO | NO | NO | NO |
| 31 | Tyrosine-protein kinase JAK3 | NO | NO | NO | NO |
| 32 | Histone deacetylase 6 | NO | NO | NO | NO |
| 33 | DNA Topoisomerase IB | NO | YES | NO | YES |
| 34 | Cyclophilin A | NO | NO | NO | NO |
| 35 | FMS-like tyrosine kinase 3 | NO | YES | NO | YES |
| 36 | PI3-kinase p110 subunit α | NO | NO | YES | YES |

**REFERENCES**

1. Stadler, M.; Monticelli, S.; Seidel, T.; Luger, D.; Salzer, I.; Boehm, S.; Holzer, W.; Schwarzer, C.; Urban, E.; Khom, S.; et al. Design, Synthesis, and Pharmacological Evaluation of Novel β2/3 Subunit-Selective γ-Aminobutyric Acid Type A (GABAA) Receptor Modulators. *J. Med. Chem.* **2019**, *62*, 317–341.

2. Wehn, P.M.; Rizzi, J.P.; Dixon, D.D.; Grina, J.A.; Schlachter, S.T.; Wang, B.; Xu, R.; Yang, H.; Du, X.; Han, G.; et al. Design and Activity of Specific Hypoxia-Inducible Factor-2α (HIF-2α) Inhibitors for the Treatment of Clear Cell Renal Cell Carcinoma: Discovery of Clinical Candidate (S)-3-((2,2-Difluoro-1-hydroxy-7-(methylsulfonyl)-2,3-dihydro-1H-inden-4-yl)oxy)-5-fluorobenzonitrile (PT2385). *J. Med. Chem.* **2018**, *61*, 9691–9721.

3. Buchler, I.; Akuma, D.; Au, V.; Carr, G.; de León, P.; DePasquale, M.; Ernst, G.; Huang, Y.; Kimos, M.; Kolobova, A.; et al. Optimization of 8-Hydroxyquinolines as Inhibitors of Catechol O-Methyltransferase. *J. Med. Chem.* **2018**, *61*, 9647–9665.

4. Daina, A.; Giuliano, C.; Pietra, C.; Wang, J.; Chi, Y.; Zou, Z.; Li, F.; Yan, Z.; Zhou, Y.; Guainazzi, A.; et al. Rational Design, Synthesis, and Pharmacological Characterization of Novel Ghrelin Receptor Inverse Agonists as Potential Treatment against Obesity-Related Metabolic Diseases. *J. Med. Chem.* **2018**, *61*, 11039–11060.

5. An, H.; Lee, S.; Lee, J.M.; Jo, D.H.; Kim, J.; Jeong, Y.-S.; Heo, M.J.; Cho, C.S.; Choi, H.; Seo, J.H.; et al. Novel Hypoxia-Inducible Factor 1α (HIF-1α) Inhibitors for Angiogenesis-Related Ocular Diseases: Discovery of a Novel Scaffold via Ring-Truncation Strategy. *J. Med. Chem.* **2018**, *61*, 9266–9286.

6. Aida, J.; Fushimi, M.; Kusumoto, T.; Sugiyama, H.; Arimura, N.; Ikeda, S.; Sasaki, M.; Sogabe, S.; Aoyama, K.; Koike, T. Design, Synthesis, and Evaluation of Piperazinyl Pyrrolidin-2-ones as a Novel Series of Reversible Monoacylglycerol Lipase Inhibitors. *J. Med. Chem.* **2018**, *61*, 9205–9217.

7. Lee, P.S.; Lapointe, G.; Madera, A.M.; Simmons, R.L.; Xu, W.; Yifru, A.; Tjandra, M.; Karur, S.; Rico, A.; Thompson, K.; et al. Application of Virtual Screening to the Identification of New LpxC Inhibitor Chemotypes, Oxazolidinone and Isoxazoline. *J. Med. Chem.* **2018**, *61*, 9360–9370.

8. Huddle, B.C.; Grimley, E.; Buchman, C.D.; Chtcherbinine, M.; Debnath, B.; Mehta, P.; Yang, K.; Morgan, C.A.; Li, S.; Felton, J.; et al. Structure-Based Optimization of a Novel Class of Aldehyde Dehydrogenase 1A (ALDH1A) Subfamily-Selective Inhibitors as Potential Adjuncts to Ovarian Cancer Chemotherapy. *J. Med. Chem.* **2018**, *61*, 8754–8773.

9. Fensome, A.; Ambler, C.M.; Arnold, E.; Banker, M.E.; Brown, M.F.; Chrencik, J.; Clark, J.D.; Dowty, M.E.; Efremov, I.V.; Flick, A.; et al. Dual Inhibition of TYK2 and JAK1 for the Treatment of Autoimmune Diseases: Discovery of ((S)-2,2-Difluorocyclopropyl)((1R,5S)-3-(2-((1-methyl-1H-pyrazol-4-yl)amino)pyrimidin-4-yl)-3,8-diazabicyclo[3.2.1]octan-8-yl)methanone (PF-06700841). *J. Med. Chem.* **2018**, *61*, 8597–8612.

10. Cisar, J.S.; Weber, O.D.; Clapper, J.R.; Blankman, J.L.; Henry, C.L.; Simon, G.M.; Alexander, J.P.; Jones, T.K.; Ezekowitz, R.A.B.; O’Neill, G.P.; et al. Identification of ABX-1431, a Selective Inhibitor of Monoacylglycerol Lipase and Clinical Candidate for Treatment of Neurological Disorders. *J. Med. Chem.* **2018**, *61*, 9062–9084.

11. Pero, J.E.; Matthews, J.M.; Behm, D.J.; Brnardic, E.J.; Brooks, C.; Budzik, B.W.; Costell, M.H.; Donatelli, C.A.; Eisennagel, S.H.; Erhard, K.; et al. Design and Optimization of Sulfone Pyrrolidine Sulfonamide Antagonists of Transient Receptor Potential Vanilloid-4 with in Vivo Activity in a Pulmonary Edema Model. *J. Med. Chem.* **2018**, *61*, 11209–11220.

12. Shibuya, K.; Kawamine, K.; Ozaki, C.; Ohgiya, T.; Edano, T.; Yoshinaka, Y.; Tsunenari, Y. Discovery of Clinical Candidate 2-(4-(2-((1H-Benzo[d]imidazol-2-yl)thio)ethyl)piperazin-1-yl)-N-(6-methyl-2,4-bis(methylthio)pyridin-3-yl)acetamide Hydrochloride [K-604], an Aqueous-Soluble Acyl-CoA:Cholesterol O-Acyltransferase-1 Inhibitor. *J. Med. Chem.* **2018**, *61*, 10635–10650.

13. Casimiro-Garcia, A.; Trujillo, J.I.; Vajdos, F.; Juba, B.; Banker, M.E.; Aulabaugh, A.; Balbo, P.; Bauman, J.; Chrencik, J.; Coe, J.W.; et al. Identification of Cyanamide-Based Janus Kinase 3 (JAK3) Covalent Inhibitors. *J. Med. Chem.* **2018**, *61*, 10665–10699.

14. Bhatia, S.; Krieger, V.; Groll, M.; Osko, J.D.; Reßing, N.; Ahlert, H.; Borkhardt, A.; Kurz, T.; Christianson, D.W.; Hauer, J.; et al. Discovery of the First-in-Class Dual Histone Deacetylase–Proteasome Inhibitor. *J. Med. Chem.* **2018**, *61*, 10299–10309.

15. Zhang, X.-R.; Wang, H.-W.; Tang, W.-L.; Zhang, Y.; Yang, H.; Hu, D.-X.; Ravji, A.; Marchand, C.; Kiselev, E.; Ofori-Atta, K.; et al. Discovery, Synthesis, and Evaluation of Oxynitidine Derivatives as Dual Inhibitors of DNA Topoisomerase IB (TOP1) and Tyrosyl-DNA Phosphodiesterase 1 (TDP1), and Potential Antitumor Agents. *J. Med. Chem.* **2018**, *61*, 9908–9930.

16. Brnardic, E.J.; Ye, G.; Brooks, C.; Donatelli, C.; Barton, L.; McAtee, J.; Sanchez, R.M.; Shu, A.; Erhard, K.; Terrell, L.; et al. Discovery of Pyrrolidine Sulfonamides as Selective and Orally Bioavailable Antagonists of Transient Receptor Potential Vanilloid-4 (TRPV4). *J. Med. Chem.* **2018**, *61*, 9738–9755.

17. Liang, X.; Wang, B.; Chen, C.; Wang, A.; Hu, C.; Zou, F.; Yu, K.; Liu, Q.; Li, F.; Hu, Z.; et al. Discovery of N-(4-(6-acetamidopyrimidin-4-yloxy)phenyl)-2-(2-(trifluoromethyl)phenyl)acetamide (CHMFL-FLT3-335) as a Potent Fms-like Tyrosine Kinase 3 Internal Tandem Duplications (FLT3-ITD) Mutant Selective Inhibitor for Acute Myeloid Leukemia. *J. Med. Chem.* **2018**.

18. Barton, N.; Convery, M.A.; Cooper, A.W.J.; Down, K.D.; Hamblin, N.; Inglis, G.; Peace, S.; Rowedder, J.E.; Rowland, P.; Taylor, J.A.; et al. Discovery of Potent, Efficient and Selective Inhibitors of Phosphoinositide 3-Kinase δ Through a Deconstruction and Regrowth Approach. *J. Med. Chem.* **2018,** *61*, 11061–11073.
