# Supplementary material for: Accelerating Drug Discovery by Early Protein Drug Target Prediction Based on a Multi-Fingerprint Similarity Search
Source: Molecules. 2019 Jun 14;24(12):2233. doi: 10.3390/molecules24122233 (PMC6631269; doi:10.3390/molecules24122233)
Supplement: Supplementary file 1 [file molecules-24-02233-s001.zip › File_S2.zip!/File_S2/Target_Prediction_compound#1.pdf]

# MuSSel Prediction $k_i$:

```
1 rank
Protease : Human immunodeficiency virus 1
      score: 9.261 on CHEMBL2366517 based on 12 fingerprints
Fingerprint type         Ligand  Tanimoto Similarity  activity Valid fg
       FeatMFP1  CHEMBL2165884            0.806452    11.390        *
           MFP1  CHEMBL1276099            0.625000     0.026        *
         RDKit7  CHEMBL2165897            0.788600     0.303        *
        Pattern  CHEMBL1276099            0.875668     0.026        *
        AP_bits  CHEMBL3605642            0.658711     0.012        *
        TT_bits  CHEMBL1958486            0.551402     0.020        *
            FP2  CHEMBL2165893            0.771930     0.027        *
  hybridization  CHEMBL2165887            0.796502     3.375        *
   substructure  CHEMBL2165898            0.869565     0.230        *
          graph  CHEMBL2165897            0.778098     0.303
        pubchem  CHEMBL2165890            0.858447     0.217        *
      cdk_maccs  CHEMBL1276099            0.925532     0.026        *
   klekota_roth  CHEMBL1819298            0.733333    12.500        *
      *** ki ACTIVITY *** value prediction
      based on 12 locally validated fgps ---> 3.045
["TT_bits", "substructure", "cdk_maccs", "Pattern", "FP2", "MFP1",
"klekota_roth", "pubchem", "AP_bits", "FeatMFP1", "RDKit7",
"hybridization"]

2 rank
Human immunodeficiency virus type 1 protease : Human immunodeficiency
virus 1
      score: 2.811 on CHEMBL243 based on 4 fingerprints
Fingerprint type        Ligand  Tanimoto Similarity  activity Valid fg
       FeatMFP1  CHEMBL377249            0.666667     5.300
           MFP1  CHEMBL213012            0.620690     0.140        *
         RDKit7  CHEMBL213012            0.644537     0.140
        Pattern  CHEMBL330404            0.827901   190.000
        AP_bits  CHEMBL220078            0.606459     0.235        *
        TT_bits  CHEMBL213012            0.530000     0.140
            FP2  CHEMBL213012            0.601732     0.140
  hybridization    CHEMBL1323            0.654466     0.014
   substructure  CHEMBL120843            0.791667    38.000
          graph  CHEMBL213012            0.586301     0.140
        pubchem  CHEMBL330404            0.764192   190.000
      cdk_maccs  CHEMBL219288            0.873684    10.700        *
   klekota_roth  CHEMBL213012            0.710345     0.140        *
      *** ki ACTIVITY *** value prediction
      based on 4 locally validated fgps ---> 1.971
["AP_bits", "klekota_roth", "MFP1", "cdk_maccs"]
```

# MuSSel Prediction $IC_{50}$:

```
1 rank
Protease : Human immunodeficiency virus 1
      score: 5.629 on CHEMBL2366517 based on 8 fingerprints
Fingerprint type         Ligand  Tanimoto Similarity  activity Valid fg
       FeatMFP1  CHEMBL2041942             0.818182       1.5        *
           MFP1   CHEMBL254349             0.620690       1.8        *
         RDKit7   CHEMBL507319             0.700464      10.0        *
        Pattern  CHEMBL2041942             0.838752       1.5
        AP_bits  CHEMBL2041942             0.681490       1.5        *
        TT_bits   CHEMBL254349             0.524752       1.8        *
            FP2   CHEMBL507319             0.612903      10.0
  hybridization  CHEMBL2041942             0.685210       1.5        *
   substructure   CHEMBL503571             0.760000       6.0
          graph   CHEMBL507319             0.644068      10.0
        pubchem      CHEMBL163             0.707113       0.6
      cdk_maccs  CHEMBL2041942             0.885417       1.5        *
   klekota_roth   CHEMBL509137             0.713287       3.8        *
      *** ic50 ACTIVITY *** value prediction
      based on 8 locally validated fgps ---> 5.079
["TT_bits", "cdk_maccs", "AP_bits", "MFP1", "klekota_roth", "FeatMFP1",
"RDKit7", "hybridization"]
```

# SwissTargetPrediction report:

**Reference:**
Gfeller D., Michielin O. & Zoete V.
Shaping the interaction landscape of bioactive molecules, *Bioinformatics* (2013) 29:3073-3079.

**Query Molecule**

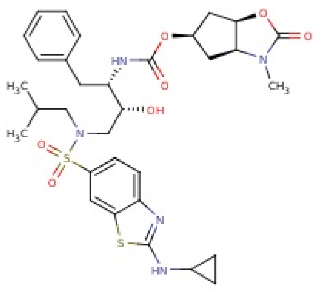

**Frequency of Target Class**

| Target | Uniprot ID | Gene code | ChEMBL ID | Probability | # sim. cmpds (3D / 2D) | Target Class |
|---|---|---|---|---|---|---|
| Cytochrome P450 3A4 | P08684 | CYP3A4 | CHEMBL340 | | 5 / 1 | Enzyme |
| Thromboxane-A synthase | P24557 | TBXAS1 | CHEMBL1835 | | 6 / 1 | Enzyme |
| Cytochrome P450 3A5 *(by homology)* | P20815 | CYP3A5 | CHEMBL3019 | | 5 / 1 | Enzyme |
| Cytochrome P450 3A7 *(by homology)* | P24462 | CYP3A7 | | | 5 / 1 | Enzyme |
| Cytochrome P450 3A43 *(by homology)* | Q9HB55 | CYP3A43 | CHEMBL5792 | | 5 / 1 | Enzyme |
| Muscleblind-like protein 1 | Q9NR56 | MBNL1 | CHEMBL1293317 | | 177 / 2 | Unclassified |
| Muscleblind-like protein 2 *(by homology)* | Q5VZF2 | MBNL2 | | | 177 / 2 | Unclassified |
| Muscleblind-like protein 3 *(by homology)* | Q9NUK0 | MBNL3 | | | 177 / 2 | Unclassified |
| Microtubule-associated protein tau | P10636 | MAPT | CHEMBL1293224 | | 181 / 13 | Unclassified |
| Dual specificity tyrosine-phosphorylation-regulated kinase 1A | Q13627 | DYRK1A | CHEMBL2292 | | 10 / 12 | Ser_Thr_Tyr Kinase |
| C-C chemokine receptor type 1 *(by homology)* | P32246 | CCR1 | CHEMBL2413 | | 106 / 13 | Membrane receptor |
| C-C chemokine receptor type 2 *(by homology)* | P41597 | CCR2 | CHEMBL4015 | | 106 / 13 | Membrane receptor |
| C-C chemokine receptor type 3 | P51677 | CCR3 | CHEMBL3473 | | 106 / 13 | Membrane receptor |
| C-C chemokine receptor type 5 | P51681 | CCR5 | CHEMBL274 | | 106 / 13 | Membrane receptor |
| C-C chemokine receptor-like 2 *(by homology)* | C9JP23 | CCRL2 | | | 102 / 13 | Membrane receptor |

# Polypharmacology Browser 2 Prediction:

Targets predicted using NN(ECfp4) + NB(ECfp4). Save Table

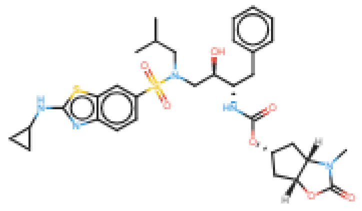

Query molecule

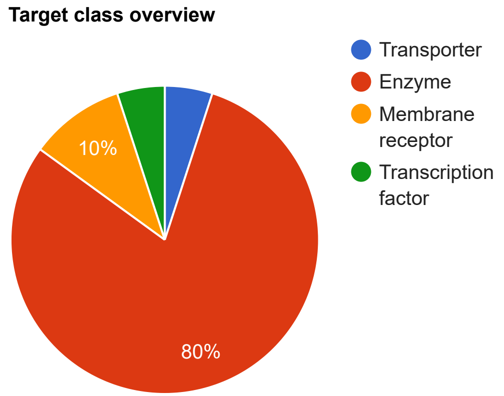

| Rank | ChEMBL ID | Common name | Nearest neighbours |
|---|---|---|---|
| 1 | CHEMBL204 (https://www.ebi.ac.uk/chembl/target/inspect/CHEMBL204) | Thrombin | Show NN |
| 2 | CHEMBL283 (https://www.ebi.ac.uk/chembl/target/inspect/CHEMBL283) | Matrix metalloproteinase 3 | Show NN |
| 3 | CHEMBL340 (https://www.ebi.ac.uk/chembl/target/inspect/CHEMBL340) | Cytochrome P450 3A4 | Show NN |
| 4 | CHEMBL332 (https://www.ebi.ac.uk/chembl/target/inspect/CHEMBL332) | Matrix metalloproteinase-1 | Show NN |
| 5 | CHEMBL2334 (https://www.ebi.ac.uk/chembl/target/inspect/CHEMBL2334) | Caspase-3 | Show NN |
| 6 | CHEMBL261 (https://www.ebi.ac.uk/chembl/target/inspect/CHEMBL261) | Carbonic anhydrase I | Show NN |
| 7 | CHEMBL205 (https://www.ebi.ac.uk/chembl/target/inspect/CHEMBL205) | Carbonic anhydrase II | Show NN |
| 8 | CHEMBL333 (https://www.ebi.ac.uk/chembl/target/inspect/CHEMBL333) | Matrix metalloproteinase-2 | Show NN |
| 9 | CHEMBL3594 (https://www.ebi.ac.uk/chembl/target/inspect/CHEMBL3594) | Carbonic anhydrase IX | Show NN |
| 10 | CHEMBL4561 (https://www.ebi.ac.uk/chembl/target/inspect/CHEMBL4561) | Neuropeptide Y receptor type 5 | Show NN |
| 11 | CHEMBL321 (https://www.ebi.ac.uk/chembl/target/inspect/CHEMBL321) | Matrix metalloproteinase 9 | Show NN |
| 12 | CHEMBL4072 (https://www.ebi.ac.uk/chembl/target/inspect/CHEMBL4072) | Cathepsin B | Show NN |
| 13 | CHEMBL280 (https://www.ebi.ac.uk/chembl/target/inspect/CHEMBL280) | Matrix metalloproteinase 13 | Show NN |
| 14 | CHEMBL3242 (https://www.ebi.ac.uk/chembl/target/inspect/CHEMBL3242) | Carbonic anhydrase XII | Show NN |
| 15 | CHEMBL4588 (https://www.ebi.ac.uk/chembl/target/inspect/CHEMBL4588) | Matrix metalloproteinase 8 | Show NN |
| 16 | CHEMBL3869 (https://www.ebi.ac.uk/chembl/target/inspect/CHEMBL3869) | Matrix metalloproteinase 14 | Show NN |
| 17 | CHEMBL3706 (https://www.ebi.ac.uk/chembl/target/inspect/CHEMBL3706) | ADAM17 | Show NN |
| 18 | CHEMBL1741186 (https://www.ebi.ac.uk/chembl/target/inspect/CHEMBL1741186) | Nuclear receptor ROR-gamma | Show NN |

| | | | |
|---|---|---|---|
| 19 | CHEMBL2871 (https://www.ebi.ac.uk/chembl/target/inspect/CHEMBL2871) | Cholecystokinin A receptor | Show NN |
| 20 | CHEMBL4076 (https://www.ebi.ac.uk/chembl/target/inspect/CHEMBL4076) | Sodium/calcium exchanger 1 | Show NN |
