# Supplementary material for: Accelerating Drug Discovery by Early Protein Drug Target Prediction Based on a Multi-Fingerprint Similarity Search
Source: Molecules. 2019 Jun 14;24(12):2233. doi: 10.3390/molecules24122233 (PMC6631269; doi:10.3390/molecules24122233)
Supplement: Supplementary file 1 [file molecules-24-02233-s001.zip › File_S2.zip!/File_S2/Target_Prediction_compound#10.pdf]

MuSSel Prediction IC$_{50}$:

1 rank
Tyrosine-protein kinase SRC : Homo sapiens
score: 6.857 on CHEMBL267 based on 10 fingerprints

Fingerprint type	Ligand	Tanimoto Similarity	activity	Valid fg
FeatMFP1	CHEMBL355269	0.735294	63.1	*
MFP1	CHEMBL168656	0.584906	270.0	*
RDKit7	CHEMBL355891	0.748187	50.0	*
Pattern	CHEMBL169043	0.811350	24.0	
AP_bits	CHEMBL352619	0.517544	400.0	*
TT_bits	CHEMBL168656	0.592105	270.0	*
FP2	CHEMBL168656	0.669856	270.0	*
hybridization	CHEMBL169043	0.671031	24.0	*
substructure	CHEMBL1873475	0.739130	262.6	
graph	CHEMBL395665	0.565217	19700.0	
pubchem	CHEMBL168656	0.833333	270.0	*
cdk_maccs	CHEMBL395664	0.833333	14400.0	*
klekota_roth	CHEMBL1873475	0.671053	262.6	*

*** ic50 ACTIVITY *** value prediction
based on 10 locally validated fgps ---> 1888.354
["TT_bits", "cdk_maccs", "AP_bits", "MFP1", "klekota_roth", "pubchem", "FP2", "FeatMFP1", "RDKit7", "hybridization"]

2 rank
Epidermal growth factor receptor erbB1 : Homo sapiens
score: 6.812 on CHEMBL203 based on 10 fingerprints

Fingerprint type	Ligand	Tanimoto Similarity	activity	Valid fg
FeatMFP1	CHEMBL355269	0.735294	750.0	*
MFP1	CHEMBL168656	0.584906	2100.0	*
RDKit7	CHEMBL355891	0.748187	830.0	*
Pattern	CHEMBL168656	0.801858	2100.0	
AP_bits	CHEMBL355891	0.511312	830.0	*
TT_bits	CHEMBL168656	0.592105	2100.0	*
FP2	CHEMBL168656	0.669856	2100.0	*
hybridization	CHEMBL168656	0.653846	2100.0	*
substructure	CHEMBL1272222	0.772727	20.0	
graph	CHEMBL3416437	0.641221	153.0	
pubchem	CHEMBL168656	0.833333	2100.0	*
cdk_maccs	CHEMBL3647970	0.802817	48.8	*
klekota_roth	CHEMBL3647973	0.680000	60.9	*

*** ic50 ACTIVITY *** value prediction
based on 10 locally validated fgps ---> 1057.807
["TT_bits", "cdk_maccs", "AP_bits", "MFP1", "klekota_roth", "pubchem", "FP2", "FeatMFP1", "RDKit7", "hybridization"]

3 rank
Fibroblast growth factor receptor 2 : Homo sapiens
score: 6.388 on CHEMBL4142 based on 9 fingerprints

Fingerprint type	Ligand	Tanimoto Similarity	activity	Valid fg
FeatMFP1	CHEMBL3701258	0.750000	0.4	*
MFP1	CHEMBL3701258	0.607843	0.4	*
RDKit7	CHEMBL3701278	0.689006	0.3	*
Pattern	CHEMBL3701259	0.846034	3.2	*
AP_bits	CHEMBL3701258	0.531915	0.4	*
TT_bits	CHEMBL3701258	0.608108	0.4	*
FP2	CHEMBL3701258	0.585586	0.4	
hybridization	CHEMBL3701258	0.650238	0.4	*
substructure	CHEMBL3701240	0.791667	6.9	
graph	CHEMBL3701258	0.656250	0.4	
pubchem	CHEMBL3701259	0.796610	3.2	
cdk_maccs	CHEMBL3701258	0.861538	0.4	*

```
   klekota_roth  CHEMBL3701258             0.842857       0.4        *
      *** ic50 ACTIVITY *** value prediction
      based on 9 locally validated fgps ---> 3.089
["TT_bits", "cdk_maccs", "Pattern", "MFP1", "klekota_roth", "AP_bits",
"FeatMFP1", "RDKit7", "hybridization"]

4 rank
Tyrosine-protein kinase SRC : Gallus gallus
      score: 6.141 on CHEMBL3655 based on 9 fingerprints
Fingerprint type        Ligand  Tanimoto Similarity  activity Valid fg
       FeatMFP1  CHEMBL168382             0.733333       2.0        *
           MFP1  CHEMBL168656             0.584906    4400.0        *
         RDKit7  CHEMBL355019             0.738929     700.0        *
        Pattern  CHEMBL169043             0.811350    3300.0
        AP_bits  CHEMBL352619             0.517544     400.0        *
        TT_bits  CHEMBL168656             0.592105    4400.0        *
            FP2  CHEMBL168656             0.669856    4400.0        *
  hybridization  CHEMBL169043             0.671031    3300.0        *
   substructure  CHEMBL168382             0.681818       2.0
          graph  CHEMBL169043             0.537594    3300.0
        pubchem  CHEMBL168656             0.833333    4400.0        *
      cdk_maccs  CHEMBL168656             0.800000    4400.0        *
   klekota_roth  CHEMBL168656             0.516854    4400.0
      *** ic50 ACTIVITY *** value prediction
      based on 8 locally validated fgps ---> 2500.662
["TT_bits", "AP_bits", "MFP1", "pubchem", "FP2", "FeatMFP1", "RDKit7",
"hybridization"]

5 rank
Tyrosine-protein kinase LCK : Homo sapiens
      score: 5.131 on CHEMBL258 based on 8 fingerprints
Fingerprint type         Ligand  Tanimoto Similarity  activity Valid fg
       FeatMFP1    CHEMBL66102             0.709677       6.0        *
           MFP1   CHEMBL262276             0.528302     290.0        *
         RDKit7   CHEMBL297278             0.698466     240.0        *
        Pattern  CHEMBL3786567             0.787834    7400.0
        AP_bits  CHEMBL1873475             0.494929       2.0        *
        TT_bits   CHEMBL172514             0.545455     620.0        *
            FP2   CHEMBL297278             0.640000     240.0        *
  hybridization  CHEMBL3786167             0.588509    4900.0
   substructure  CHEMBL1873475             0.739130       2.0
          graph   CHEMBL397143             0.582707   20800.0
        pubchem   CHEMBL297278             0.791304     240.0
      cdk_maccs   CHEMBL233540             0.842857    2950.0        *
   klekota_roth  CHEMBL1873475             0.671053       2.0        *
      *** ic50 ACTIVITY *** value prediction
      based on 8 locally validated fgps ---> 352.557
["TT_bits", "AP_bits", "MFP1", "klekota_roth", "FP2", "FeatMFP1",
"RDKit7", "cdk_maccs"]
```

SwissTargetPrediction report:

Reference:
Gfeller D., Michielin O. & Zoete V.
Shaping the interaction landscape of bioactive molecules, *Bioinformatics* (2013) 29:3073-3079.

Query Molecule

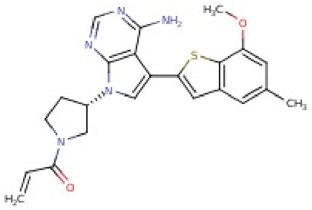

Frequency of Target Class

Target	Uniprot ID	Gene code	ChEMBL ID	Probability	# sim. cmpds (3D / 2D)	Target Class
Tyrosine-protein kinase Lck	P06239	LCK	CHEMBL258		117 / 53	Tyr Kinase
Tyrosine-protein kinase Lyn *(by homology)*	P07948	LYN	CHEMBL3905		117 / 53	Tyr Kinase
Tyrosine-protein kinase HCK *(by homology)*	P08631	HCK	CHEMBL3234		117 / 53	Tyr Kinase
Tyrosine-protein kinase Blk *(by homology)*	P51451	BLK	CHEMBL2250		117 / 53	Tyr Kinase
Epidermal growth factor receptor	P00533	EGFR	CHEMBL203		134 / 46	Tyr Kinase
Receptor tyrosine-protein kinase erbB-2	P04626	ERBB2	CHEMBL1824		133 / 46	Tyr Kinase
ERBB4 intracellular domain *(by homology)*	Q15303	ERBB4	CHEMBL3009		133 / 46	Tyr Kinase
Receptor tyrosine-protein kinase erbB-3 *(by homology)*	P21860	ERBB3	CHEMBL5838		133 / 46	Tyr Kinase
Tyrosine-protein kinase Fyn *(by homology)*	P06241	FYN	CHEMBL1841		82 / 122	Tyr Kinase
Tyrosine-protein kinase Yes *(by homology)*	P07947	YES1	CHEMBL2073		82 / 122	Tyr Kinase
Tyrosine-protein kinase Fgr *(by homology)*	P09769	FGR	CHEMBL4454		82 / 122	Tyr Kinase
Proto-oncogene tyrosine-protein kinase Src	P12931	SRC	CHEMBL267		82 / 122	Tyr Kinase
Tyrosine-protein kinase FRK *(by homology)*	P42685	FRK	CHEMBL4223		82 / 122	Tyr Kinase
Macrophage colony-stimulating factor 1 receptor	P07333	CSF1R	CHEMBL1844		134 / 3	Tyr Kinase
Mast/stem cell growth factor receptor Kit	P10721	KIT	CHEMBL1936		134 / 3	Tyr Kinase

Polypharmacology Browser 2 Prediction:

Targets predicted using NN(ECfp4) + NB(ECfp4). Save Table

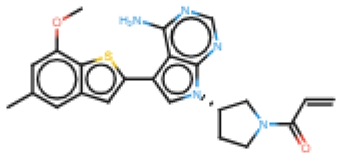

Query molecule

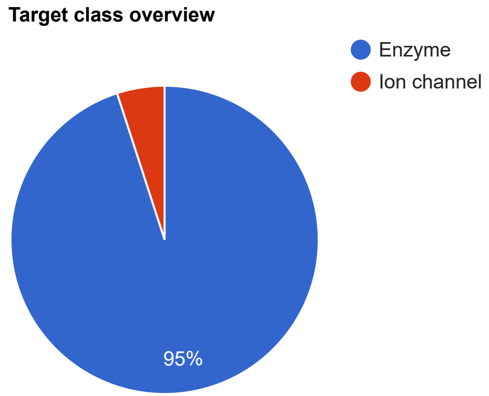

Rank	ChEMBL ID	Common name	Nearest neighbours
1	CHEMBL203 (https://www.ebi.ac.uk/chembl/target/inspect/CHEMBL203)	Epidermal growth factor receptor erbB1	Show NN
2	CHEMBL267 (https://www.ebi.ac.uk/chembl/target/inspect/CHEMBL267)	Tyrosine-protein kinase SRC	Show NN
3	CHEMBL279 (https://www.ebi.ac.uk/chembl/target/inspect/CHEMBL279)	Vascular endothelial growth factor receptor 2	Show NN
4	CHEMBL3650 (https://www.ebi.ac.uk/chembl/target/inspect/CHEMBL3650)	Fibroblast growth factor receptor 1	Show NN
5	CHEMBL4128 (https://www.ebi.ac.uk/chembl/target/inspect/CHEMBL4128)	Tyrosine-protein kinase TIE-2	Show NN
6	CHEMBL258 (https://www.ebi.ac.uk/chembl/target/inspect/CHEMBL258)	Tyrosine-protein kinase LCK	Show NN
7	CHEMBL1981 (https://www.ebi.ac.uk/chembl/target/inspect/CHEMBL1981)	Insulin receptor	Show NN
8	CHEMBL1957 (https://www.ebi.ac.uk/chembl/target/inspect/CHEMBL1957)	Insulin-like growth factor I receptor	Show NN
9	CHEMBL3905 (https://www.ebi.ac.uk/chembl/target/inspect/CHEMBL3905)	Tyrosine-protein kinase Lyn	Show NN
10	CHEMBL2742 (https://www.ebi.ac.uk/chembl/target/inspect/CHEMBL2742)	Fibroblast growth factor receptor 3	Show NN
11	CHEMBL4142 (https://www.ebi.ac.uk/chembl/target/inspect/CHEMBL4142)	Fibroblast growth factor receptor 2	Show NN
12	CHEMBL2959 (https://www.ebi.ac.uk/chembl/target/inspect/CHEMBL2959)	Tyrosine-protein kinase ITK/TSK	Show NN
13	CHEMBL240 (https://www.ebi.ac.uk/chembl/target/inspect/CHEMBL240)	HERG	Show NN
14	CHEMBL2185 (https://www.ebi.ac.uk/chembl/target/inspect/CHEMBL2185)	Serine/threonine-protein kinase Aurora-B	Show NN
15	CHEMBL2148 (https://www.ebi.ac.uk/chembl/target/inspect/CHEMBL2148)	Tyrosine-protein kinase JAK3	Show NN
16	CHEMBL3835 (https://www.ebi.ac.uk/chembl/target/inspect/CHEMBL3835)	Serine/threonine-protein kinase NEK2	Show NN
17	CHEMBL5251 (https://www.ebi.ac.uk/chembl/target/inspect/CHEMBL5251)	Tyrosine-protein kinase BTK	Show NN
18	CHEMBL1974 (https://www.ebi.ac.uk/chembl/target/inspect/CHEMBL1974)	Tyrosine-protein kinase receptor FLT3	Show NN

19	CHEMBL3267 (https://www.ebi.ac.uk/chembl/target/inspect/CHEMBL3267)	PI3-kinase p110-gamma subunit	Show NN
20	CHEMBL1862 (https://www.ebi.ac.uk/chembl/target/inspect/CHEMBL1862)	Tyrosine-protein kinase ABL	Show NN
