# Supplementary material for: Accelerating Drug Discovery by Early Protein Drug Target Prediction Based on a Multi-Fingerprint Similarity Search
Source: Molecules. 2019 Jun 14;24(12):2233. doi: 10.3390/molecules24122233 (PMC6631269; doi:10.3390/molecules24122233)
Supplement: Supplementary file 1 [file molecules-24-02233-s001.zip › File_S2.zip!/File_S2/Target_Prediction_compound#11.pdf]

MuSSel Prediction k_i:

```
1 rank
Dopamine D1 receptor : Homo sapiens
     score: 8.188 on CHEMBL2056 based on 10 fingerprints
Fingerprint type         Ligand  Tanimoto Similarity  activity Valid fg
       FeatMFP1  CHEMBL3671282             0.782609      7.98        *
           MFP1  CHEMBL3671282             0.741935      7.98        *
         RDKit7  CHEMBL3671282             0.803384      7.98        *
        Pattern  CHEMBL3671282             0.934236      7.98        *
        AP_bits  CHEMBL3671282             0.865613      7.98        *
        TT_bits  CHEMBL3671282             0.843750      7.98        *
            FP2  CHEMBL3671282             0.732558      7.98        *
  hybridization  CHEMBL3671282             0.797619      7.98        *
   substructure  CHEMBL3671273             0.857143     34.80
          graph  CHEMBL3671282             0.800000      7.98
        pubchem  CHEMBL3671282             0.817073      7.98
      cdk_maccs  CHEMBL3671282             0.886364      7.98        *
   klekota_roth  CHEMBL3671282             0.800000      7.98        *
     *** ki ACTIVITY *** value prediction
     based on 10 locally validated fgps ---> 32.870
["TT_bits", "cdk_maccs", "Pattern", "FP2", "MFP1", "klekota_roth",
"AP_bits", "FeatMFP1", "RDKit7", "hybridization"]
```

SwissTargetPrediction report:

Reference:
Gfeller D., Michielin O. & Zoete V.
Shaping the interaction landscape of bioactive molecules, *Bioinformatics* (2013) 29:3073-3079.

Query Molecule

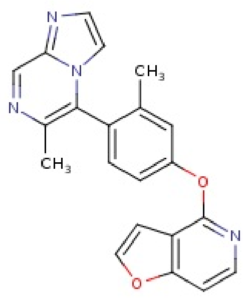

Frequency of Target Class

Target	Uniprot ID	Gene code	ChEMBL ID	Probability	# sim. cmpds (3D / 2D)	Target Class
Microtubule-associated protein tau	P10636	MAPT	CHEMBL1293224		386 / 13	Unclassified
Muscleblind-like protein 1	Q9NR56	MBNL1	CHEMBL1293317		185 / 3	Unclassified
Muscleblind-like protein 2 *(by homology)*	Q5VZF2	MBNL2			185 / 3	Unclassified
Muscleblind-like protein 3 *(by homology)*	Q9NUK0	MBNL3			185 / 3	Unclassified
Adenosine receptor A2a	P29274	ADORA2A	CHEMBL251		127 / 2	Membrane receptor
Adenosine receptor A1 *(by homology)*	P30542	ADORA1	CHEMBL226		127 / 2	Membrane receptor
cAMP and cAMP-inhibited cGMP 3',5'-cyclic phosphodiesterase 10A	Q9Y233	PDE10A	CHEMBL4409		27 / 12	Enzyme
cGMP-specific 3',5'-cyclic phosphodiesterase	O76074	PDE5A	CHEMBL1827		13 / 4	Enzyme
Dual 3',5'-cyclic-AMP and -GMP phosphodiesterase 11A	Q9HCR9	PDE11A	CHEMBL2717		10 / 4	Enzyme
Vascular endothelial growth factor receptor 1 *(by homology)*	P17948	FLT1	CHEMBL1868		107 / 4	Tyr Kinase
Vascular endothelial growth factor receptor 3 *(by homology)*	P35916	FLT4	CHEMBL1955		107 / 4	Tyr Kinase
Vascular endothelial growth factor receptor 2	P35968	KDR	CHEMBL279		107 / 4	Tyr Kinase
Glycogen synthase kinase-3 alpha *(by homology)*	P49840	GSK3A	CHEMBL2850		53 / 4	Ser_Thr Kinase
Glycogen synthase kinase-3 beta	P49841	GSK3B	CHEMBL262		53 / 4	Ser_Thr Kinase
Histamine H3 receptor	Q9Y5N1	HRH3	CHEMBL264		3 / 22	Membrane receptor

Polypharmacology Browser 2 Prediction:

Targets predicted using NN(ECfp4) + NB(ECfp4). Save Table

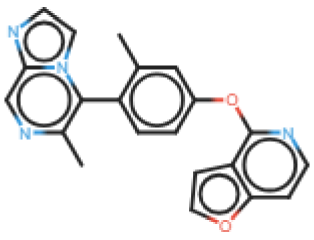

Query molecule

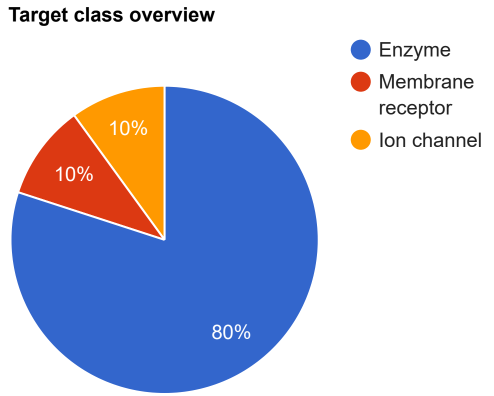

Rank	ChEMBL ID	Common name	Nearest neighbours
1	CHEMBL2056 (https://www.ebi.ac.uk/chembl/target/inspect/CHEMBL2056)	Dopamine D1 receptor	Show NN
2	CHEMBL279 (https://www.ebi.ac.uk/chembl/target/inspect/CHEMBL279)	Vascular endothelial growth factor receptor 2	Show NN
3	CHEMBL262 (https://www.ebi.ac.uk/chembl/target/inspect/CHEMBL262)	Glycogen synthase kinase-3 beta	Show NN
4	CHEMBL4409 (https://www.ebi.ac.uk/chembl/target/inspect/CHEMBL4409)	Phosphodiesterase 10A	Show NN
5	CHEMBL340 (https://www.ebi.ac.uk/chembl/target/inspect/CHEMBL340)	Cytochrome P450 3A4	Show NN
6	CHEMBL6140 (https://www.ebi.ac.uk/chembl/target/inspect/CHEMBL6140)	cAMP and cAMP-inhibited cGMP 3',5'-cyclic phosphodiesterase 10A	Show NN
7	CHEMBL1974 (https://www.ebi.ac.uk/chembl/target/inspect/CHEMBL1974)	Tyrosine-protein kinase receptor FLT3	Show NN
8	CHEMBL260 (https://www.ebi.ac.uk/chembl/target/inspect/CHEMBL260)	MAP kinase p38 alpha	Show NN
9	CHEMBL1957 (https://www.ebi.ac.uk/chembl/target/inspect/CHEMBL1957)	Insulin-like growth factor I receptor	Show NN
10	CHEMBL4296 (https://www.ebi.ac.uk/chembl/target/inspect/CHEMBL4296)	Sodium channel protein type IX alpha subunit	Show NN
11	CHEMBL2185 (https://www.ebi.ac.uk/chembl/target/inspect/CHEMBL2185)	Serine/threonine-protein kinase Aurora-B	Show NN
12	CHEMBL1955 (https://www.ebi.ac.uk/chembl/target/inspect/CHEMBL1955)	Vascular endothelial growth factor receptor 3	Show NN
13	CHEMBL240 (https://www.ebi.ac.uk/chembl/target/inspect/CHEMBL240)	HERG	Show NN

14	CHEMBL4005 (https://www.ebi.ac.uk/chembl/target/inspect/CHEMBL4005)	PI3-kinase p110-alpha subunit	Show NN
15	CHEMBL258 (https://www.ebi.ac.uk/chembl/target/inspect/CHEMBL258)	Tyrosine-protein kinase LCK	Show NN
16	CHEMBL2276 (https://www.ebi.ac.uk/chembl/target/inspect/CHEMBL2276)	c-Jun N-terminal kinase 1	Show NN
17	CHEMBL4722 (https://www.ebi.ac.uk/chembl/target/inspect/CHEMBL4722)	Serine/threonine-protein kinase Aurora-A	Show NN
18	CHEMBL1951 (https://www.ebi.ac.uk/chembl/target/inspect/CHEMBL1951)	Monoamine oxidase A	Show NN
19	CHEMBL4439 (https://www.ebi.ac.uk/chembl/target/inspect/CHEMBL4439)	TGF-beta receptor type I	Show NN
20	CHEMBL226 (https://www.ebi.ac.uk/chembl/target/inspect/CHEMBL226)	Adenosine A1 receptor	Show NN
