# Supplementary material for: Accelerating Drug Discovery by Early Protein Drug Target Prediction Based on a Multi-Fingerprint Similarity Search
Source: Molecules. 2019 Jun 14;24(12):2233. doi: 10.3390/molecules24122233 (PMC6631269; doi:10.3390/molecules24122233)
Supplement: Supplementary file 1 [file molecules-24-02233-s001.zip › File_S2.zip!/File_S2/Target_Prediction_compound#12.pdf]

MuSSel Prediction k_i:

```
1 rank
Homeodomain-interacting protein kinase 4 : Homo sapiens
      score: 12.707 on CHEMBL1075167 based on 13 fingerprints
Fingerprint type         Ligand  Tanimoto Similarity  activity Valid fg
       FeatMFP1  CHEMBL1975121             1.000000    794.33        *
           MFP1  CHEMBL1975121             1.000000    794.33        *
         RDKit7  CHEMBL1975121             1.000000    794.33        *
        Pattern  CHEMBL1975121             1.000000    794.33        *
        AP_bits  CHEMBL1975121             1.000000    794.33        *
        TT_bits  CHEMBL1975121             1.000000    794.33        *
            FP2  CHEMBL1975121             1.000000    794.33        *
  hybridization  CHEMBL1975121             0.877451    794.33        *
   substructure  CHEMBL1969049             1.000000    398.11        *
          graph  CHEMBL1969049             0.829268    398.11        *
        pubchem  CHEMBL1970217             1.000000   1258.93        *
      cdk_maccs  CHEMBL1975121             1.000000    794.33        *
   klekota_roth  CHEMBL1975121             1.000000    794.33        *
      *** ki ACTIVITY *** value prediction
      based on 13 locally validated fgps ---> 1176.319
["TT_bits", "substructure", "cdk_maccs", "Pattern", "FP2", "MFP1",
"klekota_roth", "pubchem", "graph", "AP_bits", "FeatMFP1", "RDKit7",
"hybridization"]

2 rank
Serine/threonine-protein kinase AKT2 : Homo sapiens
      score: 12.707 on CHEMBL2431 based on 13 fingerprints
Fingerprint type         Ligand  Tanimoto Similarity  activity Valid fg
       FeatMFP1  CHEMBL1975121             1.000000   2511.89        *
           MFP1  CHEMBL1975121             1.000000   2511.89        *
         RDKit7  CHEMBL1975121             1.000000   2511.89        *
        Pattern  CHEMBL1975121             1.000000   2511.89        *
        AP_bits  CHEMBL1975121             1.000000   2511.89        *
        TT_bits  CHEMBL1975121             1.000000   2511.89        *
            FP2  CHEMBL1975121             1.000000   2511.89        *
  hybridization  CHEMBL1975121             0.877451   2511.89        *
   substructure  CHEMBL1969049             1.000000   3981.07        *
          graph  CHEMBL1969049             0.829268   3981.07        *
        pubchem  CHEMBL1970217             1.000000   1258.93        *
      cdk_maccs  CHEMBL1975121             1.000000   2511.89        *
   klekota_roth  CHEMBL1975121             1.000000   2511.89        *
      *** ki ACTIVITY *** value prediction
      based on 13 locally validated fgps ---> 2572.927
["TT_bits", "substructure", "cdk_maccs", "Pattern", "FP2", "MFP1",
"klekota_roth", "pubchem", "graph", "AP_bits", "FeatMFP1", "RDKit7",
"hybridization"]

3 rank
Glycogen synthase kinase-3 beta : Homo sapiens
      score: 12.707 on CHEMBL262 based on 13 fingerprints
Fingerprint type         Ligand  Tanimoto Similarity  activity Valid fg
       FeatMFP1  CHEMBL1975121             1.000000    630.96        *
           MFP1  CHEMBL1975121             1.000000    630.96        *
         RDKit7  CHEMBL1975121             1.000000    630.96        *
        Pattern  CHEMBL1975121             1.000000    630.96        *
        AP_bits  CHEMBL1975121             1.000000    630.96        *
        TT_bits  CHEMBL1975121             1.000000    630.96        *
            FP2  CHEMBL1975121             1.000000    630.96        *
  hybridization  CHEMBL1975121             0.877451    630.96        *
   substructure  CHEMBL1969049             1.000000    100.00        *
          graph  CHEMBL1969049             0.829268    100.00        *
```

```
        pubchem  CHEMBL1970217             1.000000    251.19        *
      cdk_maccs  CHEMBL1975121             1.000000    630.96        *
   klekota_roth  CHEMBL1975121             1.000000    630.96        *
      *** ki ACTIVITY *** value prediction
      based on 13 locally validated fgps ---> 581.682
["TT_bits", "substructure", "cdk_maccs", "Pattern", "FP2", "MFP1",
"klekota_roth", "pubchem", "graph", "AP_bits", "FeatMFP1", "RDKit7",
"hybridization"]

4 rank
Glycogen synthase kinase-3 alpha : Homo sapiens
      score: 12.707 on CHEMBL2850 based on 13 fingerprints
Fingerprint type         Ligand  Tanimoto Similarity  activity Valid fg
       FeatMFP1  CHEMBL1975121             1.000000    630.96        *
           MFP1  CHEMBL1975121             1.000000    630.96        *
         RDKit7  CHEMBL1975121             1.000000    630.96        *
        Pattern  CHEMBL1975121             1.000000    630.96        *
        AP_bits  CHEMBL1975121             1.000000    630.96        *
        TT_bits  CHEMBL1975121             1.000000    630.96        *
            FP2  CHEMBL1975121             1.000000    630.96        *
  hybridization  CHEMBL1975121             0.877451    630.96        *
   substructure  CHEMBL1969049             1.000000    251.19        *
          graph  CHEMBL1969049             0.829268    251.19        *
        pubchem  CHEMBL1970217             1.000000    501.19        *
      cdk_maccs  CHEMBL1975121             1.000000    630.96        *
   klekota_roth  CHEMBL1975121             1.000000    630.96        *
      *** ki ACTIVITY *** value prediction
      based on 13 locally validated fgps ---> 786.432
["TT_bits", "substructure", "cdk_maccs", "Pattern", "FP2", "MFP1",
"klekota_roth", "pubchem", "graph", "AP_bits", "FeatMFP1", "RDKit7",
"hybridization"]

5 rank
cGMP-dependent protein kinase 2 : Homo sapiens
      score: 12.707 on CHEMBL2896 based on 13 fingerprints
Fingerprint type         Ligand  Tanimoto Similarity  activity Valid fg
       FeatMFP1  CHEMBL1975121             1.000000     15.85        *
           MFP1  CHEMBL1975121             1.000000     15.85        *
         RDKit7  CHEMBL1975121             1.000000     15.85        *
        Pattern  CHEMBL1975121             1.000000     15.85        *
        AP_bits  CHEMBL1975121             1.000000     15.85        *
        TT_bits  CHEMBL1975121             1.000000     15.85        *
            FP2  CHEMBL1975121             1.000000     15.85        *
  hybridization  CHEMBL1975121             0.877451     15.85        *
   substructure  CHEMBL1969049             1.000000     79.43        *
          graph  CHEMBL1969049             0.829268     79.43        *
        pubchem  CHEMBL1970217             1.000000     50.12        *
      cdk_maccs  CHEMBL1975121             1.000000     15.85        *
   klekota_roth  CHEMBL1975121             1.000000     15.85        *
      *** ki ACTIVITY *** value prediction
      based on 13 locally validated fgps ---> 48.815
["TT_bits", "substructure", "cdk_maccs", "Pattern", "FP2", "MFP1",
"klekota_roth", "pubchem", "graph", "AP_bits", "FeatMFP1", "RDKit7",
"hybridization"]

6 rank
Protein kinase C gamma : Homo sapiens
      score: 12.707 on CHEMBL2938 based on 13 fingerprints
Fingerprint type         Ligand  Tanimoto Similarity  activity Valid fg
       FeatMFP1  CHEMBL1975121             1.000000   1000.00        *
```

```
           MFP1  CHEMBL1975121             1.000000   1000.00        *
         RDKit7  CHEMBL1975121             1.000000   1000.00        *
        Pattern  CHEMBL1975121             1.000000   1000.00        *
        AP_bits  CHEMBL1975121             1.000000   1000.00        *
        TT_bits  CHEMBL1975121             1.000000   1000.00        *
            FP2  CHEMBL1975121             1.000000   1000.00        *
  hybridization  CHEMBL1975121             0.877451   1000.00        *
   substructure  CHEMBL1970217             1.000000    794.33        *
          graph  CHEMBL1970217             0.829268    794.33        *
        pubchem  CHEMBL1970217             1.000000    794.33        *
      cdk_maccs  CHEMBL1975121             1.000000   1000.00        *
   klekota_roth  CHEMBL1975121             1.000000   1000.00        *
      *** ki ACTIVITY *** value prediction
      based on 13 locally validated fgps ---> 1173.481
["TT_bits", "substructure", "cdk_maccs", "Pattern", "FP2", "MFP1",
"klekota_roth", "pubchem", "graph", "AP_bits", "FeatMFP1", "RDKit7",
"hybridization"]

7 rank
Rho-associated protein kinase 2 : Homo sapiens
      score: 12.707 on CHEMBL2973 based on 13 fingerprints
Fingerprint type         Ligand  Tanimoto Similarity  activity Valid fg
       FeatMFP1  CHEMBL1975121             1.000000    199.53        *
           MFP1  CHEMBL1975121             1.000000    199.53        *
         RDKit7  CHEMBL1975121             1.000000    199.53        *
        Pattern  CHEMBL1975121             1.000000    199.53        *
        AP_bits  CHEMBL1975121             1.000000    199.53        *
        TT_bits  CHEMBL1975121             1.000000    199.53        *
            FP2  CHEMBL1975121             1.000000    199.53        *
  hybridization  CHEMBL1975121             0.877451    199.53        *
   substructure  CHEMBL1975121             1.000000    199.53        *
          graph  CHEMBL1975121             0.829268    199.53        *
        pubchem  CHEMBL1975121             1.000000    199.53        *
      cdk_maccs  CHEMBL1975121             1.000000    199.53        *
   klekota_roth  CHEMBL1975121             1.000000    199.53        *
      *** ki ACTIVITY *** value prediction
      based on 13 locally validated fgps ---> 122.606
["TT_bits", "substructure", "cdk_maccs", "Pattern", "FP2", "MFP1",
"klekota_roth", "pubchem", "graph", "AP_bits", "FeatMFP1", "RDKit7",
"hybridization"]

8 rank
Protein kinase C delta : Homo sapiens
      score: 12.707 on CHEMBL2996 based on 13 fingerprints
Fingerprint type         Ligand  Tanimoto Similarity  activity Valid fg
       FeatMFP1  CHEMBL1975121             1.000000     63.10        *
           MFP1  CHEMBL1975121             1.000000     63.10        *
         RDKit7  CHEMBL1975121             1.000000     63.10        *
        Pattern  CHEMBL1975121             1.000000     63.10        *
        AP_bits  CHEMBL1975121             1.000000     63.10        *
        TT_bits  CHEMBL1975121             1.000000     63.10        *
            FP2  CHEMBL1975121             1.000000     63.10        *
  hybridization  CHEMBL1975121             0.877451     63.10        *
   substructure  CHEMBL1969049             1.000000    125.89        *
          graph  CHEMBL1969049             0.829268    125.89        *
        pubchem  CHEMBL1970217             1.000000     39.81        *
      cdk_maccs  CHEMBL1975121             1.000000     63.10        *
   klekota_roth  CHEMBL1975121             1.000000     63.10        *
      *** ki ACTIVITY *** value prediction
      based on 13 locally validated fgps ---> 159.905
```

```
["TT_bits", "substructure", "cdk_maccs", "Pattern", "FP2", "MFP1",
"klekota_roth", "pubchem", "graph", "AP_bits", "FeatMFP1", "RDKit7",
"hybridization"]

9 rank
Cyclin-dependent kinase 2 : Homo sapiens
      score: 12.707 on CHEMBL301 based on 13 fingerprints
Fingerprint type         Ligand  Tanimoto Similarity  activity Valid fg
       FeatMFP1  CHEMBL1975121             1.000000   2511.89        *
           MFP1  CHEMBL1975121             1.000000   2511.89        *
         RDKit7  CHEMBL1975121             1.000000   2511.89        *
        Pattern  CHEMBL1975121             1.000000   2511.89        *
        AP_bits  CHEMBL1975121             1.000000   2511.89        *
        TT_bits  CHEMBL1975121             1.000000   2511.89        *
            FP2  CHEMBL1975121             1.000000   2511.89        *
  hybridization  CHEMBL1975121             0.877451   2511.89        *
   substructure  CHEMBL1969049             1.000000   3981.07        *
          graph  CHEMBL1969049             0.829268   3981.07        *
        pubchem  CHEMBL1970217             1.000000   1995.26        *
      cdk_maccs  CHEMBL1975121             1.000000   2511.89        *
   klekota_roth  CHEMBL1975121             1.000000   2511.89        *
      *** ki ACTIVITY *** value prediction
      based on 13 locally validated fgps ---> 2671.123
["TT_bits", "substructure", "cdk_maccs", "Pattern", "FP2", "MFP1",
"klekota_roth", "pubchem", "graph", "AP_bits", "FeatMFP1", "RDKit7",
"hybridization"]

10 rank
Protein kinase N2 : Homo sapiens
      score: 12.707 on CHEMBL3032 based on 13 fingerprints
Fingerprint type         Ligand  Tanimoto Similarity  activity Valid fg
       FeatMFP1  CHEMBL1975121             1.000000     79.43        *
           MFP1  CHEMBL1975121             1.000000     79.43        *
         RDKit7  CHEMBL1975121             1.000000     79.43        *
        Pattern  CHEMBL1975121             1.000000     79.43        *
        AP_bits  CHEMBL1975121             1.000000     79.43        *
        TT_bits  CHEMBL1975121             1.000000     79.43        *
            FP2  CHEMBL1975121             1.000000     79.43        *
  hybridization  CHEMBL1975121             0.877451     79.43        *
   substructure  CHEMBL1969049             1.000000    125.89        *
          graph  CHEMBL1969049             0.829268    125.89        *
        pubchem  CHEMBL1970217             1.000000     79.43        *
      cdk_maccs  CHEMBL1975121             1.000000     79.43        *
   klekota_roth  CHEMBL1975121             1.000000     79.43        *
      *** ki ACTIVITY *** value prediction
      based on 13 locally validated fgps ---> 280.648
["TT_bits", "substructure", "cdk_maccs", "Pattern", "FP2", "MFP1",
"klekota_roth", "pubchem", "graph", "AP_bits", "FeatMFP1", "RDKit7",
"hybridization"]

11 rank
Cyclin-dependent kinase 1 : Homo sapiens
      score: 12.707 on CHEMBL308 based on 13 fingerprints
Fingerprint type         Ligand  Tanimoto Similarity  activity Valid fg
       FeatMFP1  CHEMBL1975121             1.000000   3162.28        *
           MFP1  CHEMBL1975121             1.000000   3162.28        *
         RDKit7  CHEMBL1975121             1.000000   3162.28        *
        Pattern  CHEMBL1975121             1.000000   3162.28        *
        AP_bits  CHEMBL1975121             1.000000   3162.28        *
        TT_bits  CHEMBL1975121             1.000000   3162.28        *
```

```
            FP2  CHEMBL1975121            1.000000   3162.28        *
  hybridization  CHEMBL1975121            0.877451   3162.28        *
   substructure  CHEMBL1970217            1.000000   1584.89        *
          graph  CHEMBL1970217            0.829268   1584.89        *
        pubchem  CHEMBL1970217            1.000000   1584.89        *
      cdk_maccs  CHEMBL1975121            1.000000   3162.28        *
   klekota_roth  CHEMBL1975121            1.000000   3162.28        *
      *** ki ACTIVITY *** value prediction
      based on 13 locally validated fgps ---> 3099.333
["TT_bits", "substructure", "cdk_maccs", "Pattern", "FP2", "MFP1",
"klekota_roth", "pubchem", "graph", "AP_bits", "FeatMFP1", "RDKit7",
"hybridization"]

12 rank
Rho-associated protein kinase 1 : Homo sapiens
      score: 12.707 on CHEMBL3231 based on 13 fingerprints
Fingerprint type         Ligand  Tanimoto Similarity  activity Valid fg
       FeatMFP1  CHEMBL1975121            1.000000     2.512        *
           MFP1  CHEMBL1975121            1.000000     2.512        *
         RDKit7  CHEMBL1975121            1.000000     2.512        *
        Pattern  CHEMBL1975121            1.000000     2.512        *
        AP_bits  CHEMBL1975121            1.000000     2.512        *
        TT_bits  CHEMBL1975121            1.000000     2.512        *
            FP2  CHEMBL1975121            1.000000     2.512        *
  hybridization  CHEMBL1975121            0.877451     2.512        *
   substructure  CHEMBL1969049            1.000000    25.120        *
          graph  CHEMBL1969049            0.829268    25.120        *
        pubchem  CHEMBL1970217            1.000000    19.950        *
      cdk_maccs  CHEMBL1975121            1.000000     2.512        *
   klekota_roth  CHEMBL1975121            1.000000     2.512        *
      *** ki ACTIVITY *** value prediction
      based on 13 locally validated fgps ---> 14.191
["TT_bits", "substructure", "cdk_maccs", "Pattern", "FP2", "MFP1",
"klekota_roth", "pubchem", "graph", "AP_bits", "FeatMFP1", "RDKit7",
"hybridization"]

13 rank
Interleukin-1 receptor-associated kinase 1 : Homo sapiens
      score: 12.707 on CHEMBL3357 based on 13 fingerprints
Fingerprint type         Ligand  Tanimoto Similarity  activity Valid fg
       FeatMFP1  CHEMBL1975121            1.000000   1000.00        *
           MFP1  CHEMBL1975121            1.000000   1000.00        *
         RDKit7  CHEMBL1975121            1.000000   1000.00        *
        Pattern  CHEMBL1975121            1.000000   1000.00        *
        AP_bits  CHEMBL1975121            1.000000   1000.00        *
        TT_bits  CHEMBL1975121            1.000000   1000.00        *
            FP2  CHEMBL1975121            1.000000   1000.00        *
  hybridization  CHEMBL1975121            0.877451   1000.00        *
   substructure  CHEMBL1970217            1.000000    630.96        *
          graph  CHEMBL1970217            0.829268    630.96        *
        pubchem  CHEMBL1970217            1.000000    630.96        *
      cdk_maccs  CHEMBL1975121            1.000000   1000.00        *
   klekota_roth  CHEMBL1975121            1.000000   1000.00        *
      *** ki ACTIVITY *** value prediction
      based on 13 locally validated fgps ---> 1080.154
["TT_bits", "substructure", "cdk_maccs", "Pattern", "FP2", "MFP1",
"klekota_roth", "pubchem", "graph", "AP_bits", "FeatMFP1", "RDKit7",
"hybridization"]

14 rank
```

```
Protein kinase C theta : Homo sapiens
      score: 12.707 on CHEMBL3920 based on 13 fingerprints
Fingerprint type         Ligand  Tanimoto Similarity  activity Valid fg
       FeatMFP1  CHEMBL1975121             1.000000    125.89        *
           MFP1  CHEMBL1975121             1.000000    125.89        *
         RDKit7  CHEMBL1975121             1.000000    125.89        *
        Pattern  CHEMBL1975121             1.000000    125.89        *
        AP_bits  CHEMBL1975121             1.000000    125.89        *
        TT_bits  CHEMBL1975121             1.000000    125.89        *
            FP2  CHEMBL1975121             1.000000    125.89        *
  hybridization  CHEMBL1975121             0.877451    125.89        *
   substructure  CHEMBL1975121             1.000000    125.89        *
          graph  CHEMBL1975121             0.829268    125.89        *
        pubchem  CHEMBL1975121             1.000000    125.89        *
      cdk_maccs  CHEMBL1975121             1.000000    125.89        *
   klekota_roth  CHEMBL1975121             1.000000    125.89        *
      *** ki ACTIVITY *** value prediction
      based on 13 locally validated fgps ---> 259.806
["TT_bits", "substructure", "cdk_maccs", "Pattern", "FP2", "MFP1",
"klekota_roth", "pubchem", "graph", "AP_bits", "FeatMFP1", "RDKit7",
"hybridization"]

15 rank
Cyclin-dependent kinase 5 : Homo sapiens
      score: 12.707 on CHEMBL4036 based on 13 fingerprints
Fingerprint type         Ligand  Tanimoto Similarity  activity Valid fg
       FeatMFP1  CHEMBL1975121             1.000000   3981.07        *
           MFP1  CHEMBL1975121             1.000000   3981.07        *
         RDKit7  CHEMBL1975121             1.000000   3981.07        *
        Pattern  CHEMBL1975121             1.000000   3981.07        *
        AP_bits  CHEMBL1975121             1.000000   3981.07        *
        TT_bits  CHEMBL1975121             1.000000   3981.07        *
            FP2  CHEMBL1975121             1.000000   3981.07        *
  hybridization  CHEMBL1975121             0.877451   3981.07        *
   substructure  CHEMBL1970217             1.000000   7943.28        *
          graph  CHEMBL1970217             0.829268   7943.28        *
        pubchem  CHEMBL1970217             1.000000   7943.28        *
      cdk_maccs  CHEMBL1975121             1.000000   3981.07        *
   klekota_roth  CHEMBL1975121             1.000000   3981.07        *
      *** ki ACTIVITY *** value prediction
      based on 13 locally validated fgps ---> 5508.760
["TT_bits", "substructure", "cdk_maccs", "Pattern", "FP2", "MFP1",
"klekota_roth", "pubchem", "graph", "AP_bits", "FeatMFP1", "RDKit7",
"hybridization"]

16 rank
MAP kinase ERK2 : Homo sapiens
      score: 12.707 on CHEMBL4040 based on 13 fingerprints
Fingerprint type         Ligand  Tanimoto Similarity  activity Valid fg
       FeatMFP1  CHEMBL1975121             1.000000    501.19        *
           MFP1  CHEMBL1975121             1.000000    501.19        *
         RDKit7  CHEMBL1975121             1.000000    501.19        *
        Pattern  CHEMBL1975121             1.000000    501.19        *
        AP_bits  CHEMBL1975121             1.000000    501.19        *
        TT_bits  CHEMBL1975121             1.000000    501.19        *
            FP2  CHEMBL1975121             1.000000    501.19        *
  hybridization  CHEMBL1975121             0.877451    501.19        *
   substructure  CHEMBL1969049             1.000000    630.96        *
          graph  CHEMBL1969049             0.829268    630.96        *
        pubchem  CHEMBL1970217             1.000000    251.19        *
```

```
      cdk_maccs  CHEMBL1975121             1.000000    501.19        *
   klekota_roth  CHEMBL1975121             1.000000    501.19        *
      *** ki ACTIVITY *** value prediction
      based on 13 locally validated fgps ---> 655.112
["TT_bits", "substructure", "cdk_maccs", "Pattern", "FP2", "MFP1",
"klekota_roth", "pubchem", "graph", "AP_bits", "FeatMFP1", "RDKit7",
"hybridization"]

17 rank
cAMP-dependent protein kinase alpha-catalytic subunit : Homo sapiens
      score: 12.707 on CHEMBL4101 based on 13 fingerprints
Fingerprint type         Ligand  Tanimoto Similarity  activity Valid fg
       FeatMFP1  CHEMBL1975121             1.000000     12.59        *
           MFP1  CHEMBL1975121             1.000000     12.59        *
         RDKit7  CHEMBL1975121             1.000000     12.59        *
        Pattern  CHEMBL1975121             1.000000     12.59        *
        AP_bits  CHEMBL1975121             1.000000     12.59        *
        TT_bits  CHEMBL1975121             1.000000     12.59        *
            FP2  CHEMBL1975121             1.000000     12.59        *
  hybridization  CHEMBL1975121             0.877451     12.59        *
   substructure  CHEMBL1969049             1.000000     79.43        *
          graph  CHEMBL1969049             0.829268     79.43        *
        pubchem  CHEMBL1970217             1.000000     25.12        *
      cdk_maccs  CHEMBL1975121             1.000000     12.59        *
   klekota_roth  CHEMBL1975121             1.000000     12.59        *
      *** ki ACTIVITY *** value prediction
      based on 13 locally validated fgps ---> 136.537
["TT_bits", "substructure", "cdk_maccs", "Pattern", "FP2", "MFP1",
"klekota_roth", "pubchem", "graph", "AP_bits", "FeatMFP1", "RDKit7",
"hybridization"]

18 rank
Dual specificity protein kinase CLK4 : Homo sapiens
      score: 12.707 on CHEMBL4203 based on 13 fingerprints
Fingerprint type         Ligand  Tanimoto Similarity  activity Valid fg
       FeatMFP1  CHEMBL1975121             1.000000    251.19        *
           MFP1  CHEMBL1975121             1.000000    251.19        *
         RDKit7  CHEMBL1975121             1.000000    251.19        *
        Pattern  CHEMBL1975121             1.000000    251.19        *
        AP_bits  CHEMBL1975121             1.000000    251.19        *
        TT_bits  CHEMBL1975121             1.000000    251.19        *
            FP2  CHEMBL1975121             1.000000    251.19        *
  hybridization  CHEMBL1975121             0.877451    251.19        *
   substructure  CHEMBL1969049             1.000000   1995.26        *
          graph  CHEMBL1969049             0.829268   1995.26        *
        pubchem  CHEMBL1970217             1.000000    794.33        *
      cdk_maccs  CHEMBL1975121             1.000000    251.19        *
   klekota_roth  CHEMBL1975121             1.000000    251.19        *
      *** ki ACTIVITY *** value prediction
      based on 13 locally validated fgps ---> 705.105
["TT_bits", "substructure", "cdk_maccs", "Pattern", "FP2", "MFP1",
"klekota_roth", "pubchem", "graph", "AP_bits", "FeatMFP1", "RDKit7",
"hybridization"]

19 rank
Ribosomal protein S6 kinase alpha 5 : Homo sapiens
      score: 12.707 on CHEMBL4237 based on 13 fingerprints
Fingerprint type         Ligand  Tanimoto Similarity  activity Valid fg
       FeatMFP1  CHEMBL1975121             1.000000     19.95        *
           MFP1  CHEMBL1975121             1.000000     19.95        *
```

RDKit7	CHEMBL1975121	1.000000	19.95	*
Pattern	CHEMBL1975121	1.000000	19.95	*
AP_bits	CHEMBL1975121	1.000000	19.95	*
TT_bits	CHEMBL1975121	1.000000	19.95	*
FP2	CHEMBL1975121	1.000000	19.95	*
hybridization	CHEMBL1975121	0.877451	19.95	*
substructure	CHEMBL1969049	1.000000	1584.89	*
graph	CHEMBL1969049	0.829268	1584.89	*
pubchem	CHEMBL1970217	1.000000	199.53	*
cdk_maccs	CHEMBL1975121	1.000000	19.95	*
klekota_roth	CHEMBL1975121	1.000000	19.95	*

*** ki ACTIVITY *** value prediction
based on 13 locally validated fgps ---> 461.660
["TT_bits", "substructure", "cdk_maccs", "Pattern", "FP2", "MFP1", "klekota_roth", "pubchem", "graph", "AP_bits", "FeatMFP1", "RDKit7", "hybridization"]

20 rank
cGMP-dependent protein kinase 1 beta : Homo sapiens
score: 12.707 on CHEMBL4273 based on 13 fingerprints

Fingerprint type	Ligand	Tanimoto Similarity	activity	Valid fg
FeatMFP1	CHEMBL1975121	1.000000	7.943	*
MFP1	CHEMBL1975121	1.000000	7.943	*
RDKit7	CHEMBL1975121	1.000000	7.943	*
Pattern	CHEMBL1975121	1.000000	7.943	*
AP_bits	CHEMBL1975121	1.000000	7.943	*
TT_bits	CHEMBL1975121	1.000000	7.943	*
FP2	CHEMBL1975121	1.000000	7.943	*
hybridization	CHEMBL1975121	0.877451	7.943	*
substructure	CHEMBL1969049	1.000000	63.100	*
graph	CHEMBL1969049	0.829268	63.100	*
pubchem	CHEMBL1970217	1.000000	1000.000	*
cdk_maccs	CHEMBL1975121	1.000000	7.943	*
klekota_roth	CHEMBL1975121	1.000000	7.943	*

*** ki ACTIVITY *** value prediction
based on 13 locally validated fgps ---> 216.852
["TT_bits", "substructure", "cdk_maccs", "Pattern", "FP2", "MFP1", "klekota_roth", "pubchem", "graph", "AP_bits", "FeatMFP1", "RDKit7", "hybridization"]

21 rank
Ribosomal protein S6 kinase 1 : Homo sapiens
score: 12.707 on CHEMBL4501 based on 13 fingerprints

Fingerprint type	Ligand	Tanimoto Similarity	activity	Valid fg
FeatMFP1	CHEMBL1975121	1.000000	630.96	*
MFP1	CHEMBL1975121	1.000000	630.96	*
RDKit7	CHEMBL1975121	1.000000	630.96	*
Pattern	CHEMBL1975121	1.000000	630.96	*
AP_bits	CHEMBL1975121	1.000000	630.96	*
TT_bits	CHEMBL1975121	1.000000	630.96	*
FP2	CHEMBL1975121	1.000000	630.96	*
hybridization	CHEMBL1975121	0.877451	630.96	*
substructure	CHEMBL1969049	1.000000	2511.89	*
graph	CHEMBL1969049	0.829268	2511.89	*
pubchem	CHEMBL1970217	1.000000	398.11	*
cdk_maccs	CHEMBL1975121	1.000000	630.96	*
klekota_roth	CHEMBL1975121	1.000000	630.96	*

*** ki ACTIVITY *** value prediction
based on 13 locally validated fgps ---> 1349.714

["TT_bits", "substructure", "cdk_maccs", "Pattern", "FP2", "MFP1", "klekota_roth", "pubchem", "graph", "AP_bits", "FeatMFP1", "RDKit7", "hybridization"]

22 rank
Serine/threonine-protein kinase MRCK-A : Homo sapiens
score: 12.707 on CHEMBL4516 based on 13 fingerprints

Fingerprint type	Ligand	Tanimoto Similarity	activity	Valid fg
FeatMFP1	CHEMBL1975121	1.000000	1258.93	*
MFP1	CHEMBL1975121	1.000000	1258.93	*
RDKit7	CHEMBL1975121	1.000000	1258.93	*
Pattern	CHEMBL1975121	1.000000	1258.93	*
AP_bits	CHEMBL1975121	1.000000	1258.93	*
TT_bits	CHEMBL1975121	1.000000	1258.93	*
FP2	CHEMBL1975121	1.000000	1258.93	*
hybridization	CHEMBL1975121	0.877451	1258.93	*
substructure	CHEMBL1969049	1.000000	1995.26	*
graph	CHEMBL1969049	0.829268	1995.26	*
pubchem	CHEMBL1970217	1.000000	1995.26	*
cdk_maccs	CHEMBL1975121	1.000000	1258.93	*
klekota_roth	CHEMBL1975121	1.000000	1258.93	*

*** ki ACTIVITY *** value prediction
based on 13 locally validated fgps ---> 1487.018
["TT_bits", "substructure", "cdk_maccs", "Pattern", "FP2", "MFP1", "klekota_roth", "pubchem", "graph", "AP_bits", "FeatMFP1", "RDKit7", "hybridization"]

23 rank
Serine/threonine-protein kinase Chk1 : Homo sapiens
score: 12.707 on CHEMBL4630 based on 13 fingerprints

Fingerprint type	Ligand	Tanimoto Similarity	activity	Valid fg
FeatMFP1	CHEMBL1975121	1.000000	7943.28	*
MFP1	CHEMBL1975121	1.000000	7943.28	*
RDKit7	CHEMBL1975121	1.000000	7943.28	*
Pattern	CHEMBL1975121	1.000000	7943.28	*
AP_bits	CHEMBL1975121	1.000000	7943.28	*
TT_bits	CHEMBL1975121	1.000000	7943.28	*
FP2	CHEMBL1975121	1.000000	7943.28	*
hybridization	CHEMBL1975121	0.877451	7943.28	*
substructure	CHEMBL1970217	1.000000	5011.87	*
graph	CHEMBL1970217	0.829268	5011.87	*
pubchem	CHEMBL1970217	1.000000	5011.87	*
cdk_maccs	CHEMBL1975121	1.000000	7943.28	*
klekota_roth	CHEMBL1975121	1.000000	7943.28	*

*** ki ACTIVITY *** value prediction
based on 13 locally validated fgps ---> 6854.637
["TT_bits", "substructure", "cdk_maccs", "Pattern", "FP2", "MFP1", "klekota_roth", "pubchem", "graph", "AP_bits", "FeatMFP1", "RDKit7", "hybridization"]

24 rank
Misshapen-like kinase 1 : Homo sapiens
score: 12.707 on CHEMBL5518 based on 13 fingerprints

Fingerprint type	Ligand	Tanimoto Similarity	activity	Valid fg
FeatMFP1	CHEMBL1975121	1.000000	7943.28	*
MFP1	CHEMBL1975121	1.000000	7943.28	*
RDKit7	CHEMBL1975121	1.000000	7943.28	*
Pattern	CHEMBL1975121	1.000000	7943.28	*
AP_bits	CHEMBL1975121	1.000000	7943.28	*
TT_bits	CHEMBL1975121	1.000000	7943.28	*

```
            FP2  CHEMBL1975121             1.000000   7943.28        *
  hybridization  CHEMBL1975121             0.877451   7943.28        *
   substructure  CHEMBL1975121             1.000000   7943.28        *
          graph  CHEMBL1975121             0.829268   7943.28        *
        pubchem  CHEMBL1975121             1.000000   7943.28        *
      cdk_maccs  CHEMBL1975121             1.000000   7943.28        *
   klekota_roth  CHEMBL1975121             1.000000   7943.28        *
     *** ki ACTIVITY *** value prediction
     based on 13 locally validated fgps ---> 6813.416
["TT_bits", "substructure", "cdk_maccs", "Pattern", "FP2", "MFP1",
"klekota_roth", "pubchem", "graph", "AP_bits", "FeatMFP1", "RDKit7",
"hybridization"]

25 rank
Serine/threonine-protein kinase Sgk2 : Homo sapiens
     score: 12.707 on CHEMBL5794 based on 13 fingerprints
Fingerprint type         Ligand  Tanimoto Similarity  activity Valid fg
       FeatMFP1  CHEMBL1975121             1.000000    398.11        *
           MFP1  CHEMBL1975121             1.000000    398.11        *
         RDKit7  CHEMBL1975121             1.000000    398.11        *
        Pattern  CHEMBL1975121             1.000000    398.11        *
        AP_bits  CHEMBL1975121             1.000000    398.11        *
        TT_bits  CHEMBL1975121             1.000000    398.11        *
            FP2  CHEMBL1975121             1.000000    398.11        *
  hybridization  CHEMBL1975121             0.877451    398.11        *
   substructure  CHEMBL1969049             1.000000    316.23        *
          graph  CHEMBL1969049             0.829268    316.23        *
        pubchem  CHEMBL1970217             1.000000    199.53        *
      cdk_maccs  CHEMBL1975121             1.000000    398.11        *
   klekota_roth  CHEMBL1975121             1.000000    398.11        *
     *** ki ACTIVITY *** value prediction
     based on 13 locally validated fgps ---> 605.572
["TT_bits", "substructure", "cdk_maccs", "Pattern", "FP2", "MFP1",
"klekota_roth", "pubchem", "graph", "AP_bits", "FeatMFP1", "RDKit7",
"hybridization"]

26 rank
Serine/threonine-protein kinase PRKX : Homo sapiens
     score: 12.707 on CHEMBL5818 based on 13 fingerprints
Fingerprint type         Ligand  Tanimoto Similarity  activity Valid fg
       FeatMFP1  CHEMBL1975121             1.000000     39.81        *
           MFP1  CHEMBL1975121             1.000000     39.81        *
         RDKit7  CHEMBL1975121             1.000000     39.81        *
        Pattern  CHEMBL1975121             1.000000     39.81        *
        AP_bits  CHEMBL1975121             1.000000     39.81        *
        TT_bits  CHEMBL1975121             1.000000     39.81        *
            FP2  CHEMBL1975121             1.000000     39.81        *
  hybridization  CHEMBL1975121             0.877451     39.81        *
   substructure  CHEMBL1969049             1.000000     63.10        *
          graph  CHEMBL1969049             0.829268     63.10        *
        pubchem  CHEMBL1970217             1.000000     39.81        *
      cdk_maccs  CHEMBL1975121             1.000000     39.81        *
   klekota_roth  CHEMBL1975121             1.000000     39.81        *
     *** ki ACTIVITY *** value prediction
     based on 13 locally validated fgps ---> 154.267
["TT_bits", "substructure", "cdk_maccs", "Pattern", "FP2", "MFP1",
"klekota_roth", "pubchem", "graph", "AP_bits", "FeatMFP1", "RDKit7",
"hybridization"]

27 rank
```

Serine/threonine-protein kinase Nek4 : Homo sapiens
score: 12.707 on CHEMBL5819 based on 13 fingerprints

Fingerprint type	Ligand	Tanimoto Similarity	activity	Valid fg
FeatMFP1	CHEMBL1975121	1.000000	3162.28	*
MFP1	CHEMBL1975121	1.000000	3162.28	*
RDKit7	CHEMBL1975121	1.000000	3162.28	*
Pattern	CHEMBL1975121	1.000000	3162.28	*
AP_bits	CHEMBL1975121	1.000000	3162.28	*
TT_bits	CHEMBL1975121	1.000000	3162.28	*
FP2	CHEMBL1975121	1.000000	3162.28	*
hybridization	CHEMBL1975121	0.877451	3162.28	*
substructure	CHEMBL1970217	1.000000	5011.87	*
graph	CHEMBL1970217	0.829268	5011.87	*
pubchem	CHEMBL1970217	1.000000	5011.87	*
cdk_maccs	CHEMBL1975121	1.000000	3162.28	*
klekota_roth	CHEMBL1975121	1.000000	3162.28	*

*** ki ACTIVITY *** value prediction
based on 13 locally validated fgps ---> 3514.274
["TT_bits", "substructure", "cdk_maccs", "Pattern", "FP2", "MFP1", "klekota_roth", "pubchem", "graph", "AP_bits", "FeatMFP1", "RDKit7", "hybridization"]

28 rank
Ribosomal protein S6 kinase alpha 3 : Homo sapiens
score: 11.841 on CHEMBL2345 based on 13 fingerprints

Fingerprint type	Ligand	Tanimoto Similarity	activity	Valid fg
FeatMFP1	CHEMBL1969049	0.947368	3981.07	*
MFP1	CHEMBL1969049	0.892857	3981.07	*
RDKit7	CHEMBL1976240	0.896082	316.23	*
Pattern	CHEMBL1976240	0.978395	316.23	*
AP_bits	CHEMBL1969049	0.875536	3981.07	*
TT_bits	CHEMBL1976240	0.833333	316.23	*
FP2	CHEMBL1969049	0.922222	3981.07	*
hybridization	CHEMBL1970217	0.806306	398.11	*
substructure	CHEMBL1969049	1.000000	3981.07	*
graph	CHEMBL1969049	0.829268	3981.07	*
pubchem	CHEMBL1970217	1.000000	398.11	*
cdk_maccs	CHEMBL1969049	0.911765	3981.07	*
klekota_roth	CHEMBL1970217	0.947368	398.11	*

*** ki ACTIVITY *** value prediction
based on 13 locally validated fgps ---> 1703.729
["TT_bits", "substructure", "cdk_maccs", "Pattern", "FP2", "MFP1", "klekota_roth", "pubchem", "graph", "AP_bits", "FeatMFP1", "RDKit7", "hybridization"]

29 rank
Serine/threonine-protein kinase AKT : Homo sapiens
score: 11.841 on CHEMBL4282 based on 13 fingerprints

Fingerprint type	Ligand	Tanimoto Similarity	activity	Valid fg
FeatMFP1	CHEMBL1969049	0.947368	1995.26	*
MFP1	CHEMBL1969049	0.892857	1995.26	*
RDKit7	CHEMBL1976240	0.896082	199.53	*
Pattern	CHEMBL1976240	0.978395	199.53	*
AP_bits	CHEMBL1969049	0.875536	1995.26	*
TT_bits	CHEMBL1976240	0.833333	199.53	*
FP2	CHEMBL1969049	0.922222	1995.26	*
hybridization	CHEMBL1970217	0.806306	630.96	*
substructure	CHEMBL1969049	1.000000	1995.26	*
graph	CHEMBL1969049	0.829268	1995.26	*
pubchem	CHEMBL1970217	1.000000	630.96	*

```
      cdk_maccs  CHEMBL1969049             0.911765   1995.26        *
   klekota_roth  CHEMBL1970217             0.947368    630.96        *
      *** ki ACTIVITY *** value prediction
      based on 13 locally validated fgps ---> 1020.087
["TT_bits", "substructure", "cdk_maccs", "Pattern", "FP2", "MFP1",
"klekota_roth", "pubchem", "graph", "AP_bits", "FeatMFP1", "RDKit7",
"hybridization"]

30 rank
Serine/threonine-protein kinase AKT3 : Homo sapiens
      score: 11.841 on CHEMBL4816 based on 13 fingerprints
Fingerprint type         Ligand  Tanimoto Similarity  activity Valid fg
       FeatMFP1  CHEMBL1969049             0.947368   7943.28        *
           MFP1  CHEMBL1969049             0.892857   7943.28        *
         RDKit7  CHEMBL1976240             0.896082    630.96        *
        Pattern  CHEMBL1976240             0.978395    630.96        *
        AP_bits  CHEMBL1969049             0.875536   7943.28        *
        TT_bits  CHEMBL1976240             0.833333    630.96        *
            FP2  CHEMBL1969049             0.922222   7943.28        *
  hybridization  CHEMBL1970217             0.806306   1995.26        *
   substructure  CHEMBL1969049             1.000000   7943.28        *
          graph  CHEMBL1969049             0.829268   7943.28        *
        pubchem  CHEMBL1970217             1.000000   1995.26        *
      cdk_maccs  CHEMBL1969049             0.911765   7943.28        *
   klekota_roth  CHEMBL1970217             0.947368   1995.26        *
      *** ki ACTIVITY *** value prediction
      based on 13 locally validated fgps ---> 4060.324
["TT_bits", "substructure", "cdk_maccs", "Pattern", "FP2", "MFP1",
"klekota_roth", "pubchem", "graph", "AP_bits", "FeatMFP1", "RDKit7",
"hybridization"]

31 rank
Dual-specificity tyrosine-phosphorylation regulated kinase 1A : Homo
sapiens
      score: 11.773 on CHEMBL2292 based on 13 fingerprints
Fingerprint type         Ligand  Tanimoto Similarity  activity Valid fg
       FeatMFP1  CHEMBL1970217             0.947368   2511.89        *
           MFP1  CHEMBL1970217             0.862069   2511.89        *
         RDKit7  CHEMBL1976240             0.896082   5011.87        *
        Pattern  CHEMBL1976240             0.978395   5011.87        *
        AP_bits  CHEMBL1970217             0.848739   2511.89        *
        TT_bits  CHEMBL1976240             0.833333   5011.87        *
            FP2  CHEMBL1970217             0.912088   2511.89        *
  hybridization  CHEMBL1970217             0.806306   2511.89        *
   substructure  CHEMBL1970217             1.000000   2511.89        *
          graph  CHEMBL1970217             0.829268   2511.89        *
        pubchem  CHEMBL1970217             1.000000   2511.89        *
      cdk_maccs  CHEMBL1970217             0.911765   2511.89        *
   klekota_roth  CHEMBL1970217             0.947368   2511.89        *
      *** ki ACTIVITY *** value prediction
      based on 13 locally validated fgps ---> 3258.708
["TT_bits", "substructure", "cdk_maccs", "Pattern", "FP2", "MFP1",
"klekota_roth", "pubchem", "graph", "AP_bits", "FeatMFP1", "RDKit7",
"hybridization"]

32 rank
Mitogen-activated protein kinase kinase kinase kinase 4 : Homo sapiens
      score: 11.773 on CHEMBL6166 based on 13 fingerprints
Fingerprint type         Ligand  Tanimoto Similarity  activity Valid fg
       FeatMFP1  CHEMBL1970217             0.947368   2511.89        *
```

```
          MFP1  CHEMBL1970217             0.862069   2511.89        *
        RDKit7  CHEMBL1976240             0.896082   1995.26        *
       Pattern  CHEMBL1976240             0.978395   1995.26        *
       AP_bits  CHEMBL1970217             0.848739   2511.89        *
       TT_bits  CHEMBL1976240             0.833333   1995.26        *
           FP2  CHEMBL1970217             0.912088   2511.89        *
 hybridization  CHEMBL1970217             0.806306   2511.89        *
  substructure  CHEMBL1970217             1.000000   2511.89        *
         graph  CHEMBL1970217             0.829268   2511.89        *
       pubchem  CHEMBL1970217             1.000000   2511.89        *
     cdk_maccs  CHEMBL1970217             0.911765   2511.89        *
  klekota_roth  CHEMBL1970217             0.947368   2511.89        *
     *** ki ACTIVITY *** value prediction
     based on 13 locally validated fgps ---> 3083.520
["TT_bits", "substructure", "cdk_maccs", "Pattern", "FP2", "MFP1",
"klekota_roth", "pubchem", "graph", "AP_bits", "FeatMFP1", "RDKit7",
"hybridization"]

33 rank
Dual specificity protein kinase CLK2 : Homo sapiens
     score: 11.614 on CHEMBL4225 based on 13 fingerprints
Fingerprint type         Ligand  Tanimoto Similarity  activity Valid fg
      FeatMFP1  CHEMBL1970217             0.947368   3981.07        *
          MFP1  CHEMBL1970217             0.862069   3981.07        *
        RDKit7  CHEMBL1970217             0.863711   3981.07        *
       Pattern  CHEMBL1970217             0.929619   3981.07        *
       AP_bits  CHEMBL1970217             0.848739   3981.07        *
       TT_bits  CHEMBL1970217             0.755556   3981.07        *
           FP2  CHEMBL1970217             0.912088   3981.07        *
 hybridization  CHEMBL1970217             0.806306   3981.07        *
  substructure  CHEMBL1970217             1.000000   3981.07        *
         graph  CHEMBL1970217             0.829268   3981.07        *
       pubchem  CHEMBL1970217             1.000000   3981.07        *
     cdk_maccs  CHEMBL1970217             0.911765   3981.07        *
  klekota_roth  CHEMBL1970217             0.947368   3981.07        *
     *** ki ACTIVITY *** value prediction
     based on 13 locally validated fgps ---> 4095.601
["TT_bits", "substructure", "cdk_maccs", "Pattern", "FP2", "MFP1",
"klekota_roth", "pubchem", "graph", "AP_bits", "FeatMFP1", "RDKit7",
"hybridization"]

34 rank
Serine/threonine-protein kinase Aurora-B : Homo sapiens
     score: 10.576 on CHEMBL2185 based on 12 fingerprints
Fingerprint type         Ligand  Tanimoto Similarity  activity Valid fg
      FeatMFP1  CHEMBL1984633             0.947368   3162.28        *
          MFP1  CHEMBL1994724             0.827586    398.11        *
        RDKit7  CHEMBL1976240             0.896082   6309.57        *
       Pattern  CHEMBL1976240             0.978395   6309.57        *
       AP_bits  CHEMBL1976240             0.772908   6309.57        *
       TT_bits  CHEMBL1976240             0.833333   6309.57        *
           FP2  CHEMBL1976240             0.892473   6309.57        *
 hybridization  CHEMBL1976240             0.797357   6309.57        *
  substructure  CHEMBL1994724             0.937500    398.11        *
         graph  CHEMBL1976240             0.755556   6309.57
       pubchem  CHEMBL1976240             0.983146   6309.57        *
     cdk_maccs  CHEMBL1994724             0.837838    398.11        *
  klekota_roth  CHEMBL1984633             0.871795   3162.28        *
     *** ki ACTIVITY *** value prediction
     based on 12 locally validated fgps ---> 3348.419
```

["TT_bits", "substructure", "cdk_maccs", "Pattern", "FP2", "MFP1", "klekota_roth", "pubchem", "AP_bits", "FeatMFP1", "RDKit7", "hybridization"]

35 rank
Vascular endothelial growth factor receptor 3 : Homo sapiens
score: 10.260 on CHEMBL1955 based on 12 fingerprints

Fingerprint type	Ligand	Tanimoto Similarity	activity	Valid fg
FeatMFP1	CHEMBL2004934	0.857143	6309.57	*
MFP1	CHEMBL2004934	0.862069	6309.57	*
RDKit7	CHEMBL2004934	0.790977	6309.57	*
Pattern	CHEMBL1999428	0.895899	501.19	*
AP_bits	CHEMBL2004934	0.834025	6309.57	*
TT_bits	CHEMBL2004934	0.777778	6309.57	*
FP2	CHEMBL2004934	0.873684	6309.57	*
hybridization	CHEMBL2004934	0.795556	6309.57	*
substructure	CHEMBL2004934	0.937500	6309.57	*
graph	CHEMBL2004934	0.723404	6309.57	
pubchem	CHEMBL2004934	0.983146	6309.57	*
cdk_maccs	CHEMBL2004934	0.837838	6309.57	*
klekota_roth	CHEMBL2004934	0.813953	6309.57	*

*** ki ACTIVITY *** value prediction
based on 12 locally validated fgps ---> 4188.072
["TT_bits", "substructure", "cdk_maccs", "Pattern", "FP2", "MFP1", "klekota_roth", "pubchem", "AP_bits", "FeatMFP1", "RDKit7", "hybridization"]

36 rank
Dual-specificity tyrosine-phosphorylation regulated kinase 3 : Homo sapiens
score: 9.666 on CHEMBL4575 based on 11 fingerprints

Fingerprint type	Ligand	Tanimoto Similarity	activity	Valid fg
FeatMFP1	CHEMBL1984633	0.947368	1995.26	*
MFP1	CHEMBL1984633	0.774194	1995.26	*
RDKit7	CHEMBL2007296	0.939286	794.33	*
Pattern	CHEMBL2007296	0.969419	794.33	*
AP_bits	CHEMBL2007296	0.722628	794.33	*
TT_bits	CHEMBL2007296	0.857143	794.33	*
FP2	CHEMBL2007296	0.922222	794.33	*
hybridization	CHEMBL2007296	0.746888	794.33	*
substructure	CHEMBL2007296	0.937500	794.33	*
graph	CHEMBL2007296	0.739130	794.33	
pubchem	CHEMBL2007296	0.977654	794.33	*
cdk_maccs	CHEMBL1984633	0.775000	1995.26	
klekota_roth	CHEMBL1984633	0.871795	1995.26	*

*** ki ACTIVITY *** value prediction
based on 11 locally validated fgps ---> 948.221
["TT_bits", "substructure", "Pattern", "FP2", "MFP1", "klekota_roth", "pubchem", "AP_bits", "FeatMFP1", "RDKit7", "hybridization"]

37 rank
Serine/threonine-protein kinase Chk2 : Homo sapiens
score: 9.495 on CHEMBL2527 based on 11 fingerprints

Fingerprint type	Ligand	Tanimoto Similarity	activity	Valid fg
FeatMFP1	CHEMBL1976240	0.850000	398.11	*
MFP1	CHEMBL1976240	0.766667	398.11	*
RDKit7	CHEMBL1976240	0.896082	398.11	*
Pattern	CHEMBL1976240	0.978395	398.11	*
AP_bits	CHEMBL1976240	0.772908	398.11	*
TT_bits	CHEMBL1976240	0.833333	398.11	*

```
             FP2  CHEMBL1976240             0.892473    398.11        *
   hybridization  CHEMBL1976240             0.797357    398.11        *
    substructure  CHEMBL1969264             0.875000    794.33        *
           graph  CHEMBL1976240             0.755556    398.11
         pubchem  CHEMBL1976240             0.983146    398.11        *
       cdk_maccs  CHEMBL1976240             0.731707    398.11
    klekota_roth  CHEMBL1976240             0.850000    398.11        *
      *** ki ACTIVITY *** value prediction
      based on 11 locally validated fgps ---> 450.893
["TT_bits", "substructure", "Pattern", "FP2", "MFP1", "klekota_roth",
"pubchem", "AP_bits", "FeatMFP1", "RDKit7", "hybridization"]

38 rank
AMP-activated protein kinase, alpha-1 subunit : Homo sapiens
      score: 9.495 on CHEMBL4045 based on 11 fingerprints
Fingerprint type         Ligand  Tanimoto Similarity  activity Valid fg
        FeatMFP1  CHEMBL1976240             0.850000    251.19        *
            MFP1  CHEMBL1976240             0.766667    251.19        *
          RDKit7  CHEMBL1976240             0.896082    251.19        *
         Pattern  CHEMBL1976240             0.978395    251.19        *
         AP_bits  CHEMBL1976240             0.772908    251.19        *
         TT_bits  CHEMBL1976240             0.833333    251.19        *
             FP2  CHEMBL1976240             0.892473    251.19        *
   hybridization  CHEMBL1976240             0.797357    251.19        *
    substructure  CHEMBL1969264             0.875000    501.19        *
           graph  CHEMBL1976240             0.755556    251.19
         pubchem  CHEMBL1976240             0.983146    251.19        *
       cdk_maccs  CHEMBL1976240             0.731707    251.19
    klekota_roth  CHEMBL1976240             0.850000    251.19        *
      *** ki ACTIVITY *** value prediction
      based on 11 locally validated fgps ---> 326.755
["TT_bits", "substructure", "Pattern", "FP2", "MFP1", "klekota_roth",
"pubchem", "AP_bits", "FeatMFP1", "RDKit7", "hybridization"]

39 rank
Casein kinase I alpha : Homo sapiens
      score: 8.135 on CHEMBL2793 based on 10 fingerprints
Fingerprint type         Ligand  Tanimoto Similarity  activity Valid fg
        FeatMFP1  CHEMBL1994724             0.900000   1584.89        *
            MFP1  CHEMBL1994724             0.827586   1584.89        *
          RDKit7  CHEMBL1990254             0.716753   5011.87
         Pattern  CHEMBL1990254             0.911950   5011.87        *
         AP_bits  CHEMBL1994724             0.620690   1584.89        *
         TT_bits  CHEMBL1994724             0.702128   1584.89        *
             FP2  CHEMBL1990254             0.706522   5011.87        *
   hybridization  CHEMBL1994724             0.640569   1584.89
    substructure  CHEMBL1994724             0.937500   1584.89        *
           graph  CHEMBL1997275             0.698925   2511.89
         pubchem  CHEMBL1994724             0.880829   1584.89        *
       cdk_maccs  CHEMBL1994724             0.837838   1584.89        *
    klekota_roth  CHEMBL1994724             0.809524   1584.89        *
      *** ki ACTIVITY *** value prediction
      based on 10 locally validated fgps ---> 2453.148
["TT_bits", "substructure", "Pattern", "AP_bits", "MFP1", "pubchem",
"FP2", "FeatMFP1", "klekota_roth", "cdk_maccs"]

40 rank
Macrophage colony stimulating factor receptor : Homo sapiens
      score: 8.129 on CHEMBL1844 based on 10 fingerprints
Fingerprint type         Ligand  Tanimoto Similarity  activity Valid fg
```

```
       FeatMFP1  CHEMBL1999428             0.833333   2511.89        *
           MFP1  CHEMBL1999428             0.800000   2511.89        *
         RDKit7  CHEMBL1999428             0.785171   2511.89        *
        Pattern  CHEMBL1999428             0.895899   2511.89        *
        AP_bits  CHEMBL1999428             0.768519   2511.89        *
        TT_bits  CHEMBL1999428             0.756098   2511.89        *
            FP2  CHEMBL1999428             0.783133   2511.89        *
  hybridization  CHEMBL1999428             0.651961   2511.89
   substructure  CHEMBL1999428             0.933333   2511.89        *
          graph  CHEMBL1999428             0.597561   2511.89
        pubchem  CHEMBL1991356             0.866667   2511.89        *
      cdk_maccs  CHEMBL1991356             0.731707   2511.89
   klekota_roth  CHEMBL1999428             0.707317   2511.89        *
      *** ki ACTIVITY *** value prediction
      based on 10 locally validated fgps ---> 2486.892
["TT_bits", "substructure", "Pattern", "FP2", "MFP1", "klekota_roth",
"pubchem", "AP_bits", "FeatMFP1", "RDKit7"]

41 rank
Serine/threonine-protein kinase c-TAK1 : Homo sapiens
      score: 8.062 on CHEMBL5600 based on 10 fingerprints
Fingerprint type         Ligand  Tanimoto Similarity  activity Valid fg
       FeatMFP1  CHEMBL1991188             0.857143   3162.28        *
           MFP1  CHEMBL1991188             0.750000   3162.28        *
         RDKit7  CHEMBL1990254             0.716753   5011.87
        Pattern  CHEMBL1990254             0.911950   5011.87        *
        AP_bits  CHEMBL1991188             0.680556   3162.28        *
        TT_bits  CHEMBL2006188             0.700000   5011.87        *
            FP2  CHEMBL1991188             0.790476   3162.28        *
  hybridization  CHEMBL1991188             0.728745   3162.28        *
   substructure  CHEMBL1991188             0.882353   3162.28        *
          graph  CHEMBL2006188             0.680000   5011.87
        pubchem  CHEMBL1991188             0.951087   3162.28        *
      cdk_maccs  CHEMBL1991188             0.756098   3162.28
   klekota_roth  CHEMBL1991188             0.809524   3162.28        *
      *** ki ACTIVITY *** value prediction
      based on 10 locally validated fgps ---> 3883.412
["TT_bits", "substructure", "Pattern", "AP_bits", "MFP1", "pubchem",
"FP2", "FeatMFP1", "klekota_roth", "hybridization"]

42 rank
Serine/threonine-protein kinase 2 : Homo sapiens
      score: 7.984 on CHEMBL4202 based on 10 fingerprints
Fingerprint type         Ligand  Tanimoto Similarity  activity Valid fg
       FeatMFP1  CHEMBL1991188             0.857143   6309.57        *
           MFP1  CHEMBL1991188             0.750000   6309.57        *
         RDKit7  CHEMBL1990254             0.716753   2511.89
        Pattern  CHEMBL1990254             0.911950   2511.89        *
        AP_bits  CHEMBL1991188             0.680556   6309.57        *
        TT_bits  CHEMBL1990254             0.622222   2511.89        *
            FP2  CHEMBL1991188             0.790476   6309.57        *
  hybridization  CHEMBL1991188             0.728745   6309.57        *
   substructure  CHEMBL1991188             0.882353   6309.57        *
          graph  CHEMBL1991188             0.644860   6309.57
        pubchem  CHEMBL1991188             0.951087   6309.57        *
      cdk_maccs  CHEMBL1991188             0.756098   6309.57
   klekota_roth  CHEMBL1991188             0.809524   6309.57        *
      *** ki ACTIVITY *** value prediction
      based on 10 locally validated fgps ---> 4579.270
```

```
["TT_bits", "substructure", "Pattern", "AP_bits", "MFP1", "pubchem",
"FP2", "FeatMFP1", "klekota_roth", "hybridization"]

43 rank
Mitogen-activated protein kinase kinase kinase kinase 2 : Homo sapiens
      score: 7.984 on CHEMBL5330 based on 10 fingerprints
Fingerprint type         Ligand  Tanimoto Similarity  activity Valid fg
       FeatMFP1  CHEMBL1991188             0.857143   6309.57         *
           MFP1  CHEMBL1991188             0.750000   6309.57         *
         RDKit7  CHEMBL1990254             0.716753   3981.07
        Pattern  CHEMBL1990254             0.911950   3981.07         *
        AP_bits  CHEMBL1991188             0.680556   6309.57         *
        TT_bits  CHEMBL1990254             0.622222   3981.07         *
            FP2  CHEMBL1991188             0.790476   6309.57         *
  hybridization  CHEMBL1991188             0.728745   6309.57         *
   substructure  CHEMBL1991188             0.882353   6309.57         *
          graph  CHEMBL1991188             0.644860   6309.57
        pubchem  CHEMBL1991188             0.951087   6309.57         *
      cdk_maccs  CHEMBL1991188             0.756098   6309.57
   klekota_roth  CHEMBL1991188             0.809524   6309.57         *
      *** ki ACTIVITY *** value prediction
      based on 10 locally validated fgps ---> 4857.933
["TT_bits", "substructure", "Pattern", "AP_bits", "MFP1", "pubchem",
"FP2", "FeatMFP1", "klekota_roth", "hybridization"]

44 rank
Cell division protein kinase 8 : Homo sapiens
      score: 7.435 on CHEMBL5719 based on 9 fingerprints
Fingerprint type         Ligand  Tanimoto Similarity  activity Valid fg
       FeatMFP1  CHEMBL1994724             0.900000   3162.28         *
           MFP1  CHEMBL1994724             0.827586   3162.28         *
         RDKit7  CHEMBL1986143             0.688633   2511.89
        Pattern  CHEMBL1986143             0.919003   2511.89         *
        AP_bits  CHEMBL1994724             0.620690   3162.28         *
        TT_bits  CHEMBL1994724             0.702128   3162.28         *
            FP2  CHEMBL1986143             0.676768   2511.89
  hybridization  CHEMBL1994724             0.640569   3162.28
   substructure  CHEMBL1994724             0.937500   3162.28         *
          graph  CHEMBL1997275             0.698925    251.19
        pubchem  CHEMBL1994724             0.880829   3162.28         *
      cdk_maccs  CHEMBL1994724             0.837838   3162.28         *
   klekota_roth  CHEMBL1994724             0.809524   3162.28         *
      *** ki ACTIVITY *** value prediction
      based on 9 locally validated fgps ---> 2940.331
["TT_bits", "substructure", "Pattern", "MFP1", "pubchem", "AP_bits",
"FeatMFP1", "klekota_roth", "cdk_maccs"]

45 rank
G protein-coupled receptor kinase 5 : Homo sapiens
      score: 7.397 on CHEMBL5678 based on 9 fingerprints
Fingerprint type         Ligand  Tanimoto Similarity  activity Valid fg
       FeatMFP1  CHEMBL1994724             0.900000   1000.00         *
           MFP1  CHEMBL1994724             0.827586   1000.00         *
         RDKit7  CHEMBL1994724             0.554632   1000.00
        Pattern  CHEMBL2004159             0.875758   3981.07         *
        AP_bits  CHEMBL2004159             0.625483   3981.07         *
        TT_bits  CHEMBL1994724             0.702128   1000.00         *
            FP2  CHEMBL1994724             0.640625   1000.00
  hybridization  CHEMBL1994724             0.640569   1000.00
   substructure  CHEMBL1994724             0.937500   1000.00         *
```

```
          graph  CHEMBL1986590             0.577778   3162.28
        pubchem  CHEMBL1994724             0.880829   1000.00        *
      cdk_maccs  CHEMBL1994724             0.837838   1000.00        *
   klekota_roth  CHEMBL1994724             0.809524   1000.00        *
      *** ki ACTIVITY *** value prediction
      based on 9 locally validated fgps ---> 1801.323
["TT_bits", "substructure", "Pattern", "MFP1", "pubchem", "AP_bits",
"FeatMFP1", "klekota_roth", "cdk_maccs"]

46 rank
Serine/threonine-protein kinase Aurora-A : Homo sapiens
      score: 7.377 on CHEMBL4722 based on 9 fingerprints
Fingerprint type         Ligand  Tanimoto Similarity  activity Valid fg
       FeatMFP1  CHEMBL1994724             0.900000    199.53        *
           MFP1  CHEMBL1994724             0.827586    199.53        *
         RDKit7  CHEMBL1994724             0.554632    199.53
        Pattern  CHEMBL1994724             0.860795    199.53        *
        AP_bits  CHEMBL1994724             0.620690    199.53        *
        TT_bits  CHEMBL1994724             0.702128    199.53        *
            FP2  CHEMBL1994724             0.640625    199.53
  hybridization  CHEMBL1994724             0.640569    199.53
   substructure  CHEMBL1994724             0.937500    199.53        *
          graph  CHEMBL1965845             0.583333     63.10
        pubchem  CHEMBL1994724             0.880829    199.53        *
      cdk_maccs  CHEMBL1994724             0.837838    199.53        *
   klekota_roth  CHEMBL1994724             0.809524    199.53        *
      *** ki ACTIVITY *** value prediction
      based on 9 locally validated fgps ---> 295.853
["TT_bits", "substructure", "Pattern", "MFP1", "pubchem", "AP_bits",
"FeatMFP1", "klekota_roth", "cdk_maccs"]

47 rank
Activin receptor type-1 : Homo sapiens
      score: 7.377 on CHEMBL5903 based on 9 fingerprints
Fingerprint type         Ligand  Tanimoto Similarity  activity Valid fg
       FeatMFP1  CHEMBL1994724             0.900000   2511.89        *
           MFP1  CHEMBL1994724             0.827586   2511.89        *
         RDKit7  CHEMBL1994724             0.554632   2511.89
        Pattern  CHEMBL1994724             0.860795   2511.89        *
        AP_bits  CHEMBL1994724             0.620690   2511.89        *
        TT_bits  CHEMBL1994724             0.702128   2511.89        *
            FP2  CHEMBL1994724             0.640625   2511.89
  hybridization  CHEMBL1994724             0.640569   2511.89
   substructure  CHEMBL1994724             0.937500   2511.89        *
          graph  CHEMBL1980329             0.563830   3162.28
        pubchem  CHEMBL1994724             0.880829   2511.89        *
      cdk_maccs  CHEMBL1994724             0.837838   2511.89        *
   klekota_roth  CHEMBL1994724             0.809524   2511.89        *
      *** ki ACTIVITY *** value prediction
      based on 9 locally validated fgps ---> 2546.777
["TT_bits", "substructure", "Pattern", "MFP1", "pubchem", "AP_bits",
"FeatMFP1", "klekota_roth", "cdk_maccs"]

48 rank
LIM domain kinase 1 : Homo sapiens
      score: 7.268 on CHEMBL3836 based on 9 fingerprints
Fingerprint type         Ligand  Tanimoto Similarity  activity Valid fg
       FeatMFP1  CHEMBL1999428             0.833333   7943.28        *
           MFP1  CHEMBL1999428             0.800000   7943.28        *
         RDKit7  CHEMBL1999428             0.785171   7943.28        *
```

```
        Pattern  CHEMBL1996931             0.901493   3162.28        *
        AP_bits  CHEMBL1999428             0.768519   7943.28        *
        TT_bits  CHEMBL1999428             0.756098   7943.28        *
            FP2  CHEMBL1999428             0.783133   7943.28        *
  hybridization  CHEMBL1999428             0.651961   7943.28
   substructure  CHEMBL1999428             0.933333   7943.28        *
          graph  CHEMBL1999428             0.597561   7943.28
        pubchem  CHEMBL1999428             0.817143   7943.28
      cdk_maccs  CHEMBL1999428             0.709677   7943.28
   klekota_roth  CHEMBL1999428             0.707317   7943.28        *
      *** ki ACTIVITY *** value prediction
      based on 9 locally validated fgps ---> 7266.786
["TT_bits", "substructure", "Pattern", "FP2", "MFP1", "klekota_roth",
"AP_bits", "FeatMFP1", "RDKit7"]

49 rank
Serine/threonine-protein kinase PLK3 : Homo sapiens
      score: 7.267 on CHEMBL4897 based on 9 fingerprints
Fingerprint type         Ligand  Tanimoto Similarity  activity Valid fg
       FeatMFP1  CHEMBL1999428             0.833333   1995.26        *
           MFP1  CHEMBL1999428             0.800000   1995.26        *
         RDKit7  CHEMBL1999428             0.785171   1995.26        *
        Pattern  CHEMBL1999428             0.895899   1995.26        *
        AP_bits  CHEMBL1999428             0.768519   1995.26        *
        TT_bits  CHEMBL1999428             0.756098   1995.26        *
            FP2  CHEMBL1999428             0.783133   1995.26        *
  hybridization  CHEMBL1999428             0.651961   1995.26
   substructure  CHEMBL2005375             0.937500     12.59        *
          graph  CHEMBL1999428             0.597561   1995.26
        pubchem  CHEMBL1999428             0.817143   1995.26
      cdk_maccs  CHEMBL1999428             0.709677   1995.26
   klekota_roth  CHEMBL1999428             0.707317   1995.26        *
      *** ki ACTIVITY *** value prediction
      based on 9 locally validated fgps ---> 1884.866
["TT_bits", "substructure", "Pattern", "FP2", "MFP1", "klekota_roth",
"AP_bits", "FeatMFP1", "RDKit7"]

50 rank
Vascular endothelial growth factor receptor 1 : Homo sapiens
      score: 7.263 on CHEMBL1868 based on 9 fingerprints
Fingerprint type         Ligand  Tanimoto Similarity  activity Valid fg
       FeatMFP1  CHEMBL1999428             0.833333    158.49        *
           MFP1  CHEMBL1999428             0.800000    158.49        *
         RDKit7  CHEMBL1999428             0.785171    158.49        *
        Pattern  CHEMBL1999428             0.895899    158.49        *
        AP_bits  CHEMBL1999428             0.768519    158.49        *
        TT_bits  CHEMBL1999428             0.756098    158.49        *
            FP2  CHEMBL1999428             0.783133    158.49        *
  hybridization  CHEMBL1999428             0.651961    158.49
   substructure  CHEMBL1999428             0.933333    158.49        *
          graph  CHEMBL1999428             0.597561    158.49
        pubchem  CHEMBL1999428             0.817143    158.49
      cdk_maccs  CHEMBL1999428             0.709677    158.49
   klekota_roth  CHEMBL1999428             0.707317    158.49        *
      *** ki ACTIVITY *** value prediction
      based on 9 locally validated fgps ---> 319.984
["TT_bits", "substructure", "Pattern", "FP2", "MFP1", "klekota_roth",
"AP_bits", "FeatMFP1", "RDKit7"]

51 rank
```

Tyrosine-protein kinase receptor FLT3 : Homo sapiens
score: 7.263 on CHEMBL1974 based on 9 fingerprints

Fingerprint type	Ligand	Tanimoto Similarity	activity	Valid fg
FeatMFP1	CHEMBL1999428	0.833333	316.23	*
MFP1	CHEMBL1999428	0.800000	316.23	*
RDKit7	CHEMBL1999428	0.785171	316.23	*
Pattern	CHEMBL1999428	0.895899	316.23	*
AP_bits	CHEMBL1999428	0.768519	316.23	*
TT_bits	CHEMBL1999428	0.756098	316.23	*
FP2	CHEMBL1999428	0.783133	316.23	*
hybridization	CHEMBL1999428	0.651961	316.23	
substructure	CHEMBL1999428	0.933333	316.23	*
graph	CHEMBL1997275	0.698925	1584.89	
pubchem	CHEMBL1997275	0.842640	1584.89	
cdk_maccs	CHEMBL1982924	0.731707	398.11	
klekota_roth	CHEMBL1999428	0.707317	316.23	*

*** ki ACTIVITY *** value prediction
based on 9 locally validated fgps ---> 320.632
["TT_bits", "substructure", "Pattern", "FP2", "MFP1", "klekota_roth", "AP_bits", "FeatMFP1", "RDKit7"]

52 rank
Vascular endothelial growth factor receptor 2 : Homo sapiens
score: 7.263 on CHEMBL279 based on 9 fingerprints

Fingerprint type	Ligand	Tanimoto Similarity	activity	Valid fg
FeatMFP1	CHEMBL1999428	0.833333	6309.57	*
MFP1	CHEMBL1999428	0.800000	6309.57	*
RDKit7	CHEMBL1999428	0.785171	6309.57	*
Pattern	CHEMBL1999428	0.895899	6309.57	*
AP_bits	CHEMBL1999428	0.768519	6309.57	*
TT_bits	CHEMBL1999428	0.756098	6309.57	*
FP2	CHEMBL1999428	0.783133	6309.57	*
hybridization	CHEMBL1999428	0.651961	6309.57	
substructure	CHEMBL1999428	0.933333	6309.57	*
graph	CHEMBL1997275	0.698925	2511.89	
pubchem	CHEMBL1997275	0.842640	2511.89	
cdk_maccs	CHEMBL1999428	0.709677	6309.57	
klekota_roth	CHEMBL1999428	0.707317	6309.57	*

*** ki ACTIVITY *** value prediction
based on 9 locally validated fgps ---> 6184.382
["TT_bits", "substructure", "Pattern", "FP2", "MFP1", "klekota_roth", "AP_bits", "FeatMFP1", "RDKit7"]

53 rank
Macrophage-stimulating protein receptor : Homo sapiens
score: 6.864 on CHEMBL2689 based on 8 fingerprints

Fingerprint type	Ligand	Tanimoto Similarity	activity	Valid fg
FeatMFP1	CHEMBL2003514	0.947368	398.11	*
MFP1	CHEMBL2003514	0.925926	398.11	*
RDKit7	CHEMBL2003514	0.575967	398.11	
Pattern	CHEMBL2003514	0.957576	398.11	*
AP_bits	CHEMBL2003514	0.804979	398.11	*
TT_bits	CHEMBL2003514	0.620000	398.11	*
FP2	CHEMBL2003514	0.640351	398.11	
hybridization	CHEMBL2003514	0.593625	398.11	
substructure	CHEMBL1984296	0.875000	3981.07	*
graph	CHEMBL1997275	0.698925	2511.89	
pubchem	CHEMBL1997275	0.842640	2511.89	
cdk_maccs	CHEMBL2003514	0.861111	398.11	*
klekota_roth	CHEMBL2003514	0.871795	398.11	*

```
      *** ki ACTIVITY *** value prediction
      based on 8 locally validated fgps ---> 622.045
["TT_bits", "substructure", "Pattern", "MFP1", "AP_bits", "FeatMFP1",
"klekota_roth", "cdk_maccs"]

54 rank
Serine/threonine-protein kinase 17A : Homo sapiens
      score: 6.301 on CHEMBL4525 based on 8 fingerprints
Fingerprint type         Ligand  Tanimoto Similarity  activity Valid fg
       FeatMFP1  CHEMBL1984633            0.947368   3162.28        *
           MFP1  CHEMBL1984633            0.774194   3162.28        *
         RDKit7  CHEMBL1990254            0.716753   1584.89
        Pattern  CHEMBL1990254            0.911950   1584.89        *
        AP_bits  CHEMBL1990254            0.600000   1584.89        *
        TT_bits  CHEMBL1990254            0.622222   1584.89        *
            FP2  CHEMBL1990254            0.706522   1584.89        *
  hybridization  CHEMBL1990254            0.605381   1584.89
   substructure  CHEMBL1978099            0.823529   1000.00
          graph  CHEMBL1965845            0.583333   1258.93
        pubchem  CHEMBL1991356            0.866667    630.96        *
      cdk_maccs  CHEMBL1984633            0.775000   3162.28
   klekota_roth  CHEMBL1984633            0.871795   3162.28        *
      *** ki ACTIVITY *** value prediction
      based on 8 locally validated fgps ---> 2246.329
["TT_bits", "Pattern", "AP_bits", "MFP1", "pubchem", "FP2", "FeatMFP1",
"klekota_roth"]

55 rank
Serine/threonine-protein kinase PIM1 : Homo sapiens
      score: 5.872 on CHEMBL2147 based on 8 fingerprints
Fingerprint type         Ligand  Tanimoto Similarity  activity Valid fg
       FeatMFP1  CHEMBL1990254            0.700000   3981.07
           MFP1  CHEMBL1997534            0.594595   3162.28        *
         RDKit7  CHEMBL1990254            0.716753   3981.07
        Pattern  CHEMBL1990254            0.911950   3981.07        *
        AP_bits  CHEMBL1990254            0.600000   3981.07        *
        TT_bits  CHEMBL1990254            0.622222   3981.07        *
            FP2  CHEMBL1990254            0.706522   3981.07        *
  hybridization  CHEMBL1990254            0.605381   3981.07
   substructure  CHEMBL1997534            0.882353   3162.28        *
          graph   CHEMBL272476            0.654762   4500.00
        pubchem  CHEMBL1991356            0.866667    501.19        *
      cdk_maccs  CHEMBL1991356            0.731707    501.19
   klekota_roth  CHEMBL1997534            0.687500   3162.28        *
      *** ki ACTIVITY *** value prediction
      based on 8 locally validated fgps ---> 3289.999
["TT_bits", "substructure", "Pattern", "AP_bits", "MFP1", "pubchem",
"FP2", "klekota_roth"]

56 rank
Casein kinase I gamma 1 : Homo sapiens
      score: 5.559 on CHEMBL2426 based on 7 fingerprints
Fingerprint type         Ligand  Tanimoto Similarity  activity Valid fg
       FeatMFP1  CHEMBL1984633            0.947368   5011.87        *
           MFP1  CHEMBL1984633            0.774194   5011.87        *
         RDKit7  CHEMBL1996931            0.517442    630.96
        Pattern  CHEMBL1996931            0.901493    630.96        *
        AP_bits  CHEMBL1984633            0.557971   5011.87        *
        TT_bits  CHEMBL1984633            0.568627   5011.87        *
            FP2  CHEMBL1996931            0.556522    630.96
```

```
  hybridization  CHEMBL1984633             0.472727   5011.87
   substructure  CHEMBL2005375             0.937500   1995.26        *
          graph  CHEMBL1996831             0.566265   1584.89
        pubchem  CHEMBL1982924             0.804762   1000.00
      cdk_maccs  CHEMBL1984633             0.775000   5011.87
   klekota_roth  CHEMBL1984633             0.871795   5011.87        *
     *** ki ACTIVITY *** value prediction
     based on 7 locally validated fgps ---> 3886.443
["TT_bits", "substructure", "Pattern", "MFP1", "AP_bits", "FeatMFP1",
"klekota_roth"]

57 rank
Protein kinase C nu : Homo sapiens
     score: 5.060 on CHEMBL2595 based on 7 fingerprints
Fingerprint type         Ligand  Tanimoto Similarity  activity Valid fg
       FeatMFP1  CHEMBL1990254             0.700000   2511.89
           MFP1  CHEMBL1997534             0.594595    794.33        *
         RDKit7  CHEMBL1990254             0.716753   2511.89
        Pattern  CHEMBL1990254             0.911950   2511.89        *
        AP_bits  CHEMBL1990254             0.600000   2511.89        *
        TT_bits  CHEMBL1990254             0.622222   2511.89        *
            FP2  CHEMBL1990254             0.706522   2511.89        *
  hybridization  CHEMBL1990254             0.605381   2511.89
   substructure  CHEMBL2005375             0.937500   1995.26        *
          graph  CHEMBL1997275             0.698925    794.33
        pubchem  CHEMBL1997275             0.842640    794.33
      cdk_maccs  CHEMBL1982924             0.731707   2511.89
   klekota_roth  CHEMBL1997534             0.687500    794.33        *
     *** ki ACTIVITY *** value prediction
     based on 7 locally validated fgps ---> 1864.173
["TT_bits", "substructure", "Pattern", "AP_bits", "MFP1", "FP2",
"klekota_roth"]

58 rank
CaM kinase II delta : Homo sapiens
     score: 5.060 on CHEMBL2801 based on 7 fingerprints
Fingerprint type         Ligand  Tanimoto Similarity  activity Valid fg
       FeatMFP1  CHEMBL1990254             0.700000   2511.89
           MFP1  CHEMBL1997534             0.594595   5011.87        *
         RDKit7  CHEMBL1990254             0.716753   2511.89
        Pattern  CHEMBL1990254             0.911950   2511.89        *
        AP_bits  CHEMBL1990254             0.600000   2511.89        *
        TT_bits  CHEMBL1990254             0.622222   2511.89        *
            FP2  CHEMBL1990254             0.706522   2511.89        *
  hybridization  CHEMBL1990254             0.605381   2511.89
   substructure  CHEMBL2005375             0.937500   5011.87        *
          graph  CHEMBL2002649             0.581197   1584.89
        pubchem  CHEMBL1982924             0.804762   1258.93
      cdk_maccs  CHEMBL1982924             0.731707   1258.93
   klekota_roth  CHEMBL1997534             0.687500   5011.87        *
     *** ki ACTIVITY *** value prediction
     based on 7 locally validated fgps ---> 3583.310
["TT_bits", "substructure", "Pattern", "AP_bits", "MFP1", "FP2",
"klekota_roth"]

59 rank
CaM kinase II gamma : Homo sapiens
     score: 5.060 on CHEMBL3829 based on 7 fingerprints
Fingerprint type         Ligand  Tanimoto Similarity  activity Valid fg
       FeatMFP1  CHEMBL1990254             0.700000   3162.28
```

```
           MFP1  CHEMBL1997534            0.594595   5011.87        *
         RDKit7  CHEMBL1990254            0.716753   3162.28
        Pattern  CHEMBL1990254            0.911950   3162.28        *
        AP_bits  CHEMBL1990254            0.600000   3162.28        *
        TT_bits  CHEMBL1990254            0.622222   3162.28        *
            FP2  CHEMBL1990254            0.706522   3162.28        *
  hybridization  CHEMBL1990254            0.605381   3162.28
   substructure  CHEMBL2005375            0.937500   2511.89        *
          graph  CHEMBL1965845            0.583333   5011.87
        pubchem  CHEMBL1982924            0.804762    630.96
      cdk_maccs  CHEMBL1982924            0.731707    630.96
   klekota_roth  CHEMBL1997534            0.687500   5011.87        *
      *** ki ACTIVITY *** value prediction
      based on 7 locally validated fgps ---> 3770.980
["TT_bits", "substructure", "Pattern", "AP_bits", "MFP1", "FP2",
"klekota_roth"]

60 rank
Serine/threonine-protein kinase D2 : Homo sapiens
      score: 5.060 on CHEMBL4900 based on 7 fingerprints
Fingerprint type         Ligand  Tanimoto Similarity  activity Valid fg
       FeatMFP1  CHEMBL1990254            0.700000   1258.93
           MFP1  CHEMBL1997534            0.594595    398.11        *
         RDKit7  CHEMBL1990254            0.716753   1258.93
        Pattern  CHEMBL1990254            0.911950   1258.93        *
        AP_bits  CHEMBL1990254            0.600000   1258.93        *
        TT_bits  CHEMBL1990254            0.622222   1258.93        *
            FP2  CHEMBL1990254            0.706522   1258.93        *
  hybridization  CHEMBL1990254            0.605381   1258.93
   substructure  CHEMBL2005375            0.937500   5011.87        *
          graph  CHEMBL1965845            0.583333    501.19
        pubchem  CHEMBL1982924            0.804762   1995.26
      cdk_maccs  CHEMBL1982924            0.731707   1995.26
   klekota_roth  CHEMBL1997534            0.687500    398.11        *
      *** ki ACTIVITY *** value prediction
      based on 7 locally validated fgps ---> 1229.548
["TT_bits", "substructure", "Pattern", "AP_bits", "MFP1", "FP2",
"klekota_roth"]

61 rank
Mitogen-activated protein kinase kinase kinase kinase 5 : Homo sapiens
      score: 5.022 on CHEMBL4852 based on 7 fingerprints
Fingerprint type         Ligand  Tanimoto Similarity  activity Valid fg
       FeatMFP1  CHEMBL1990254            0.700000   2511.89
           MFP1  CHEMBL1997534            0.594595    316.23        *
         RDKit7  CHEMBL1990254            0.716753   2511.89
        Pattern  CHEMBL1990254            0.911950   2511.89        *
        AP_bits  CHEMBL1990254            0.600000   2511.89        *
        TT_bits  CHEMBL1990254            0.622222   2511.89        *
            FP2  CHEMBL1977135            0.722892   7943.28        *
  hybridization  CHEMBL1977135            0.627451   7943.28
   substructure  CHEMBL1997534            0.882353    316.23        *
          graph  CHEMBL1977135            0.597561   7943.28
        pubchem  CHEMBL1982924            0.804762   2511.89
      cdk_maccs  CHEMBL1982924            0.731707   2511.89
   klekota_roth  CHEMBL1997534            0.687500    316.23        *
      *** ki ACTIVITY *** value prediction
      based on 7 locally validated fgps ---> 2494.818
["TT_bits", "substructure", "Pattern", "AP_bits", "MFP1", "FP2",
"klekota_roth"]
```

```
62 rank
CaM kinase II beta : Homo sapiens
      score: 5.005 on CHEMBL4121 based on 7 fingerprints
Fingerprint type         Ligand  Tanimoto Similarity  activity Valid fg
       FeatMFP1  CHEMBL1990254            0.700000   7943.28
           MFP1  CHEMBL1997534            0.594595   3162.28        *
         RDKit7  CHEMBL1990254            0.716753   7943.28
        Pattern  CHEMBL1990254            0.911950   7943.28        *
        AP_bits  CHEMBL1990254            0.600000   7943.28        *
        TT_bits  CHEMBL1990254            0.622222   7943.28        *
            FP2  CHEMBL1990254            0.706522   7943.28        *
  hybridization  CHEMBL1990254            0.605381   7943.28
   substructure  CHEMBL1997534            0.882353   3162.28        *
          graph  CHEMBL1965845            0.583333   7943.28
        pubchem  CHEMBL1982924            0.804762   1584.89
      cdk_maccs  CHEMBL1982924            0.731707   1584.89
   klekota_roth  CHEMBL1997534            0.687500   3162.28        *
      *** ki ACTIVITY *** value prediction
      based on 7 locally validated fgps ---> 5782.081
["TT_bits", "substructure", "Pattern", "AP_bits", "MFP1", "FP2",
"klekota_roth"]

63 rank
MAP kinase signal-integrating kinase 2 : Homo sapiens
      score: 4.590 on CHEMBL4204 based on 6 fingerprints
Fingerprint type         Ligand  Tanimoto Similarity  activity Valid fg
       FeatMFP1  CHEMBL1990254            0.700000   3981.07
           MFP1  CHEMBL1990254            0.566667   3981.07
         RDKit7  CHEMBL1990254            0.716753   3981.07
        Pattern  CHEMBL1990254            0.911950   3981.07        *
        AP_bits  CHEMBL1990254            0.600000   3981.07        *
        TT_bits  CHEMBL1990254            0.622222   3981.07        *
            FP2  CHEMBL1990254            0.706522   3981.07        *
  hybridization  CHEMBL1990254            0.605381   3981.07
   substructure  CHEMBL3632744            0.882353     18.00        *
          graph  CHEMBL1965845            0.583333    630.96
        pubchem  CHEMBL1991356            0.866667   3981.07        *
      cdk_maccs  CHEMBL1982924            0.731707   1584.89
   klekota_roth  CHEMBL1990254            0.595238   3981.07
      *** ki ACTIVITY *** value prediction
      based on 6 locally validated fgps ---> 3320.558
["TT_bits", "substructure", "Pattern", "AP_bits", "pubchem", "FP2"]

64 rank
Casein kinase I delta : Homo sapiens
      score: 4.582 on CHEMBL2828 based on 6 fingerprints
Fingerprint type         Ligand  Tanimoto Similarity  activity Valid fg
       FeatMFP1  CHEMBL1990254            0.700000   1000.00
           MFP1  CHEMBL1990254            0.566667   1000.00
         RDKit7  CHEMBL1990254            0.716753   1000.00
        Pattern  CHEMBL1990254            0.911950   1000.00        *
        AP_bits  CHEMBL1990254            0.600000   1000.00        *
        TT_bits  CHEMBL1990254            0.622222   1000.00        *
            FP2  CHEMBL1990254            0.706522   1000.00        *
  hybridization  CHEMBL1990254            0.605381   1000.00
   substructure  CHEMBL1969264            0.875000   1258.93        *
          graph  CHEMBL2002649            0.581197   3162.28
        pubchem  CHEMBL1991356            0.866667    158.49        *
      cdk_maccs  CHEMBL1982924            0.731707   3981.07
```

```
   klekota_roth  CHEMBL1990254             0.595238   1000.00
      *** ki ACTIVITY *** value prediction
      based on 6 locally validated fgps ---> 1129.748
["TT_bits", "substructure", "Pattern", "AP_bits", "pubchem", "FP2"]

65 rank
Cytochrome P450 3A4 : Homo sapiens
      score: 3.876 on CHEMBL340 based on 5 fingerprints
Fingerprint type         Ligand  Tanimoto Similarity  activity Valid fg
       FeatMFP1  CHEMBL3338848             0.695652    5600.0
           MFP1  CHEMBL2152014             0.593750     540.0        *
         RDKit7  CHEMBL3338848             0.334451    5600.0
        Pattern  CHEMBL1743350             0.829851    2600.0
        AP_bits  CHEMBL2152010             0.530534    3100.0        *
        TT_bits  CHEMBL2152014             0.354839     540.0
            FP2  CHEMBL2152014             0.488189     540.0
  hybridization  CHEMBL3338848             0.433702    5600.0
   substructure  CHEMBL3338848             1.000000    5600.0        *
          graph  CHEMBL2152010             0.727273    3100.0
        pubchem  CHEMBL2152014             0.869110     540.0        *
      cdk_maccs  CHEMBL2152010             0.882353    3100.0        *
   klekota_roth  CHEMBL2152014             0.307692     540.0
      *** ki ACTIVITY *** value prediction
      based on 5 locally validated fgps ---> 4783.422
["AP_bits", "cdk_maccs", "MFP1", "substructure", "pubchem"]

66 rank
Neurokinin 3 receptor : Homo sapiens
      score: 3.739 on CHEMBL4429 based on 5 fingerprints
Fingerprint type         Ligand  Tanimoto Similarity  activity Valid fg
       FeatMFP1  CHEMBL2113673             0.772727      1.80        *
           MFP1   CHEMBL297736             0.625000     57.00        *
         RDKit7  CHEMBL1277075             0.462779    158.49
        Pattern    CHEMBL51352             0.754386    355.00
        AP_bits    CHEMBL50291             0.550314    666.00        *
        TT_bits  CHEMBL1277075             0.385965    158.49
            FP2    CHEMBL51120             0.567797    870.00
  hybridization    CHEMBL10295             0.630522   1144.00
   substructure    CHEMBL10536             0.933333    295.00        *
          graph    CHEMBL10079             0.730337     13.80
        pubchem    CHEMBL50742             0.826531    919.00
      cdk_maccs    CHEMBL53699             0.857143   1671.00        *
   klekota_roth   CHEMBL274763             0.480769    251.19
      *** ki ACTIVITY *** value prediction
      based on 5 locally validated fgps ---> 752.461
["FeatMFP1", "AP_bits", "MFP1", "substructure", "cdk_maccs"]

67 rank
BR serine/threonine-protein kinase 1 : Homo sapiens
      score: 3.719 on CHEMBL5650 based on 5 fingerprints
Fingerprint type         Ligand  Tanimoto Similarity  activity Valid fg
       FeatMFP1  CHEMBL1990254             0.700000    398.11
           MFP1  CHEMBL1974288             0.586207   5011.87
         RDKit7  CHEMBL1990254             0.716753    398.11
        Pattern  CHEMBL1974288             0.912500   5011.87        *
        AP_bits  CHEMBL1990254             0.600000    398.11        *
        TT_bits  CHEMBL1974288             0.622222   5011.87        *
            FP2  CHEMBL1974288             0.709677   5011.87        *
  hybridization  CHEMBL1990254             0.605381    398.11
   substructure  CHEMBL1982924             0.875000   6309.57        *
```

```
           graph  CHEMBL1965845             0.583333   1584.89
         pubchem  CHEMBL1974288             0.824176   5011.87
       cdk_maccs  CHEMBL1982924             0.731707   6309.57
    klekota_roth  CHEMBL1990254             0.595238    398.11
      *** ki ACTIVITY *** value prediction
      based on 5 locally validated fgps ---> 3789.103
["Pattern", "FP2", "TT_bits", "substructure", "AP_bits"]

68 rank
Casein kinase I gamma 2 : Homo sapiens
      score: 3.716 on CHEMBL2543 based on 5 fingerprints
Fingerprint type         Ligand  Tanimoto Similarity  activity Valid fg
        FeatMFP1  CHEMBL1990254             0.700000   2511.89
            MFP1  CHEMBL1990254             0.566667   2511.89
          RDKit7  CHEMBL1990254             0.716753   2511.89
         Pattern  CHEMBL1990254             0.911950   2511.89         *
         AP_bits  CHEMBL1990254             0.600000   2511.89         *
         TT_bits  CHEMBL1990254             0.622222   2511.89         *
             FP2  CHEMBL1990254             0.706522   2511.89         *
   hybridization  CHEMBL1990254             0.605381   2511.89
    substructure  CHEMBL1982924             0.875000   6309.57         *
           graph  CHEMBL2007064             0.567010   6309.57
         pubchem  CHEMBL1982924             0.804762   6309.57
       cdk_maccs  CHEMBL1982924             0.731707   6309.57
    klekota_roth  CHEMBL1990254             0.595238   2511.89
      *** ki ACTIVITY *** value prediction
      based on 5 locally validated fgps ---> 3271.426
["Pattern", "FP2", "TT_bits", "substructure", "AP_bits"]

69 rank
Cyclin-dependent kinase 9 : Homo sapiens
      score: 3.716 on CHEMBL3116 based on 5 fingerprints
Fingerprint type         Ligand  Tanimoto Similarity  activity Valid fg
        FeatMFP1  CHEMBL1990254             0.700000    158.49
            MFP1  CHEMBL1990254             0.566667    158.49
          RDKit7  CHEMBL1990254             0.716753    158.49
         Pattern  CHEMBL1990254             0.911950    158.49         *
         AP_bits  CHEMBL1990254             0.600000    158.49         *
         TT_bits  CHEMBL1990254             0.622222    158.49         *
             FP2  CHEMBL1990254             0.706522    158.49         *
   hybridization  CHEMBL1990254             0.605381    158.49
    substructure  CHEMBL1984296             0.875000    398.11         *
           graph  CHEMBL2002649             0.581197    501.19
         pubchem  CHEMBL1990254             0.802260    158.49
       cdk_maccs  CHEMBL2002649             0.613636    501.19
    klekota_roth  CHEMBL1990254             0.595238    158.49
      *** ki ACTIVITY *** value prediction
      based on 5 locally validated fgps ---> 206.414
["Pattern", "FP2", "TT_bits", "substructure", "AP_bits"]

70 rank
Interleukin-1 receptor-associated kinase 4 : Homo sapiens
      score: 3.716 on CHEMBL3778 based on 5 fingerprints
Fingerprint type         Ligand  Tanimoto Similarity  activity Valid fg
        FeatMFP1  CHEMBL1990254             0.700000  10000.00
            MFP1  CHEMBL1990254             0.566667  10000.00
          RDKit7  CHEMBL1990254             0.716753  10000.00
         Pattern  CHEMBL1990254             0.911950  10000.00         *
         AP_bits  CHEMBL1990254             0.600000  10000.00         *
         TT_bits  CHEMBL1990254             0.622222  10000.00         *
```

```
            FP2  CHEMBL1990254            0.706522  10000.00        *
  hybridization  CHEMBL1990254            0.605381  10000.00
   substructure  CHEMBL1982924            0.875000   2511.89        *
          graph  CHEMBL1965845            0.583333    794.33
        pubchem  CHEMBL1982924            0.804762   2511.89
      cdk_maccs  CHEMBL1982924            0.731707   2511.89
   klekota_roth  CHEMBL1990254            0.595238  10000.00
     *** ki ACTIVITY *** value prediction
     based on 5 locally validated fgps ---> 8409.678
["Pattern", "FP2", "TT_bits", "substructure", "AP_bits"]

71 rank
MAP/microtubule affinity-regulating kinase 2 : Homo sapiens
     score: 3.716 on CHEMBL3831 based on 5 fingerprints
Fingerprint type         Ligand  Tanimoto Similarity  activity Valid fg
       FeatMFP1  CHEMBL1990254            0.700000   6309.57
           MFP1  CHEMBL1990254            0.566667   6309.57
         RDKit7  CHEMBL1990254            0.716753   6309.57
        Pattern  CHEMBL1990254            0.911950   6309.57        *
        AP_bits  CHEMBL1990254            0.600000   6309.57        *
        TT_bits  CHEMBL1990254            0.622222   6309.57        *
            FP2  CHEMBL1990254            0.706522   6309.57        *
  hybridization  CHEMBL1990254            0.605381   6309.57
   substructure  CHEMBL1982924            0.875000   1258.93        *
          graph  CHEMBL1965845            0.583333    794.33
        pubchem  CHEMBL1982924            0.804762   1258.93
      cdk_maccs  CHEMBL1982924            0.731707   1258.93
   klekota_roth  CHEMBL1990254            0.595238   6309.57
     *** ki ACTIVITY *** value prediction
     based on 5 locally validated fgps ---> 5332.038
["Pattern", "FP2", "TT_bits", "substructure", "AP_bits"]

72 rank
Dual specificity tyrosine-phosphorylation-regulated kinase 1B : Homo
sapiens
     score: 3.716 on CHEMBL5543 based on 5 fingerprints
Fingerprint type         Ligand  Tanimoto Similarity  activity Valid fg
       FeatMFP1  CHEMBL1990254            0.700000   1584.89
           MFP1  CHEMBL1990254            0.566667   1584.89
         RDKit7  CHEMBL1990254            0.716753   1584.89
        Pattern  CHEMBL1990254            0.911950   1584.89        *
        AP_bits  CHEMBL1990254            0.600000   1584.89        *
        TT_bits  CHEMBL1990254            0.622222   1584.89        *
            FP2  CHEMBL1990254            0.706522   1584.89        *
  hybridization  CHEMBL1990254            0.605381   1584.89
   substructure  CHEMBL1984296            0.875000   1000.00        *
          graph  CHEMBL1968394            0.609756  19952.62
        pubchem  CHEMBL1990254            0.802260   1584.89
      cdk_maccs   CHEMBL269538            0.648649    166.00
   klekota_roth  CHEMBL1990254            0.595238   1584.89
     *** ki ACTIVITY *** value prediction
     based on 5 locally validated fgps ---> 1467.912
["Pattern", "FP2", "TT_bits", "substructure", "AP_bits"]

73 rank
Serine/threonine-protein kinase PAK 4 : Homo sapiens
     score: 3.701 on CHEMBL4482 based on 5 fingerprints
Fingerprint type         Ligand  Tanimoto Similarity  activity Valid fg
       FeatMFP1  CHEMBL1974288            0.666667   2511.89
           MFP1  CHEMBL1974288            0.586207   2511.89
```

```
         RDKit7  CHEMBL1974288             0.712821   2511.89
        Pattern  CHEMBL1974288             0.912500   2511.89        *
        AP_bits  CHEMBL1974288             0.582031   2511.89        *
        TT_bits  CHEMBL1974288             0.622222   2511.89        *
            FP2  CHEMBL1974288             0.709677   2511.89        *
  hybridization  CHEMBL1974288             0.587719   2511.89
   substructure  CHEMBL1984296             0.875000   3981.07        *
          graph  CHEMBL1974288             0.556818   2511.89
        pubchem  CHEMBL1974288             0.824176   2511.89
      cdk_maccs  CHEMBL1984296             0.609756   3981.07
   klekota_roth  CHEMBL1974288             0.530612   2511.89
      *** ki ACTIVITY *** value prediction
      based on 5 locally validated fgps ---> 2805.726
["Pattern", "FP2", "TT_bits", "substructure", "AP_bits"]

74 rank
Serotonin 1a (5-HT1a) receptor : Rattus norvegicus
      score: 3.479 on CHEMBL273 based on 4 fingerprints
Fingerprint type         Ligand  Tanimoto Similarity   activity Valid fg
       FeatMFP1   CHEMBL104593             0.652174      0.360
           MFP1   CHEMBL147483             0.500000     27.000
         RDKit7   CHEMBL294747             0.455335      3.162
        Pattern   CHEMBL156845             0.885886    432.000        *
        AP_bits    CHEMBL72981             0.422343   1258.930
        TT_bits   CHEMBL309021             0.392857  19952.620
            FP2   CHEMBL294747             0.568966      3.162
  hybridization  CHEMBL1161083             0.463343      2.600
   substructure  CHEMBL2112905             0.882353     58.600        *
          graph   CHEMBL315684             0.831325      2.800        *
        pubchem   CHEMBL309021             0.878947  19952.620        *
      cdk_maccs  CHEMBL2112774             0.591837     17.000
   klekota_roth  CHEMBL2112384             0.459016   4300.000
      *** ki ACTIVITY *** value prediction
      based on 4 locally validated fgps ---> 2746.154
["Pattern", "pubchem", "substructure", "graph"]

75 rank
MAP kinase p38 delta : Homo sapiens
      score: 2.844 on CHEMBL2939 based on 4 fingerprints
Fingerprint type         Ligand  Tanimoto Similarity  activity Valid fg
       FeatMFP1  CHEMBL1990254             0.700000    794.33
           MFP1  CHEMBL1974288             0.586207   5011.87
         RDKit7  CHEMBL1990254             0.716753    794.33
        Pattern  CHEMBL1974288             0.912500   5011.87        *
        AP_bits  CHEMBL1990254             0.600000    794.33        *
        TT_bits  CHEMBL1974288             0.622222   5011.87        *
            FP2  CHEMBL1974288             0.709677   5011.87        *
  hybridization  CHEMBL1990254             0.605381    794.33
   substructure  CHEMBL1991078             0.823529    501.19
          graph  CHEMBL1990254             0.563218    794.33
        pubchem  CHEMBL1974288             0.824176   5011.87
      cdk_maccs  CHEMBL1997759             0.625000    251.19
   klekota_roth  CHEMBL1990254             0.595238    794.33
      *** ki ACTIVITY *** value prediction
      based on 4 locally validated fgps ---> 2896.420
["Pattern", "FP2", "TT_bits", "AP_bits"]

76 rank
MAP kinase-activated protein kinase 2 : Homo sapiens
      score: 2.841 on CHEMBL2208 based on 4 fingerprints
```

Fingerprint type	Ligand	Tanimoto Similarity	activity	Valid fg
FeatMFP1	CHEMBL1990254	0.700000	794.33	
MFP1	CHEMBL1990254	0.566667	794.33	
RDKit7	CHEMBL1990254	0.716753	794.33	
Pattern	CHEMBL1990254	0.911950	794.33	*
AP_bits	CHEMBL1990254	0.600000	794.33	*
TT_bits	CHEMBL1990254	0.622222	794.33	*
FP2	CHEMBL1990254	0.706522	794.33	*
hybridization	CHEMBL1990254	0.605381	794.33	
substructure	CHEMBL1987009	0.823529	6309.57	
graph	CHEMBL235658	0.613636	330.00	
pubchem	CHEMBL1990254	0.802260	794.33	
cdk_maccs	CHEMBL1982506	0.617021	3981.07	
klekota_roth	CHEMBL1990254	0.595238	794.33	

*** ki ACTIVITY *** value prediction
based on 4 locally validated fgps ---> 794.330
["Pattern", "FP2", "TT_bits", "AP_bits"]

77 rank
Serine/threonine-protein kinase PAK 1 : Homo sapiens
score: 2.841 on CHEMBL4600 based on 4 fingerprints

Fingerprint type	Ligand	Tanimoto Similarity	activity	Valid fg
FeatMFP1	CHEMBL1990254	0.700000	6309.57	
MFP1	CHEMBL1990254	0.566667	6309.57	
RDKit7	CHEMBL1990254	0.716753	6309.57	
Pattern	CHEMBL1990254	0.911950	6309.57	*
AP_bits	CHEMBL1990254	0.600000	6309.57	*
TT_bits	CHEMBL1990254	0.622222	6309.57	*
FP2	CHEMBL1990254	0.706522	6309.57	*
hybridization	CHEMBL1990254	0.605381	6309.57	
substructure	CHEMBL1999931	0.764706	3981.07	
graph	CHEMBL1990254	0.563218	6309.57	
pubchem	CHEMBL1990254	0.802260	6309.57	
cdk_maccs	CHEMBL1967302	0.583333	7943.28	
klekota_roth	CHEMBL1990254	0.595238	6309.57	

*** ki ACTIVITY *** value prediction
based on 4 locally validated fgps ---> 6309.570
["Pattern", "FP2", "TT_bits", "AP_bits"]

78 rank
MAP kinase p38 gamma : Homo sapiens
score: 2.841 on CHEMBL4674 based on 4 fingerprints

Fingerprint type	Ligand	Tanimoto Similarity	activity	Valid fg
FeatMFP1	CHEMBL1990254	0.700000	3162.28	
MFP1	CHEMBL1990254	0.566667	3162.28	
RDKit7	CHEMBL1990254	0.716753	3162.28	
Pattern	CHEMBL1990254	0.911950	3162.28	*
AP_bits	CHEMBL1990254	0.600000	3162.28	*
TT_bits	CHEMBL1990254	0.622222	3162.28	*
FP2	CHEMBL1990254	0.706522	3162.28	*
hybridization	CHEMBL1990254	0.605381	3162.28	
substructure	CHEMBL1978099	0.823529	3162.28	
graph	CHEMBL1990254	0.563218	3162.28	
pubchem	CHEMBL1990254	0.802260	3162.28	
cdk_maccs	CHEMBL116070	0.613636	5011.87	
klekota_roth	CHEMBL1990254	0.595238	3162.28	

*** ki ACTIVITY *** value prediction
based on 4 locally validated fgps ---> 3162.280
["Pattern", "FP2", "TT_bits", "AP_bits"]

MuSSel Prediction IC_{50}:

```
1 rank
Rho-associated protein kinase 2 : Homo sapiens
     score: 8.799 on CHEMBL2973 based on 11 fingerprints
Fingerprint type         Ligand  Tanimoto Similarity  activity Valid fg
       FeatMFP1  CHEMBL3578250            0.947368      0.51        *
           MFP1  CHEMBL3578250            0.727273      0.51        *
         RDKit7  CHEMBL3578250            0.595696      0.51
        Pattern   CHEMBL597321            0.889231   5000.00        *
        AP_bits  CHEMBL3578250            0.714815      0.51        *
        TT_bits  CHEMBL3578250            0.589286      0.51        *
            FP2  CHEMBL1923167            0.708738      2.00        *
  hybridization  CHEMBL3578253            0.647619      1.16        *
   substructure  CHEMBL3578250            1.000000      0.51        *
          graph  CHEMBL3578253            0.788462      1.16        *
        pubchem  CHEMBL3578250            0.935829      0.51        *
      cdk_maccs  CHEMBL3578250            0.769231      0.51
   klekota_roth  CHEMBL3578253            0.850000      1.16        *
     *** ic50 ACTIVITY *** value prediction
     based on 11 locally validated fgps ---> 233.845
["TT_bits", "substructure", "Pattern", "AP_bits", "MFP1", "pubchem",
"graph", "FP2", "FeatMFP1", "klekota_roth", "hybridization"]

2 rank
MAP kinase p38 alpha : Homo sapiens
     score: 7.489 on CHEMBL260 based on 10 fingerprints
Fingerprint type         Ligand  Tanimoto Similarity  activity Valid fg
       FeatMFP1   CHEMBL197753            0.695652     130.0        *
           MFP1  CHEMBL2059876            0.545455   22000.0        *
         RDKit7   CHEMBL517671            0.590258      23.0
        Pattern  CHEMBL1796293            0.884146     600.0        *
        AP_bits   CHEMBL186802            0.529595     140.0        *
        TT_bits   CHEMBL158477            0.436364     420.0
            FP2   CHEMBL517671            0.656566      23.0        *
  hybridization   CHEMBL517671            0.680000      23.0        *
   substructure   CHEMBL184555            1.000000      70.0        *
          graph   CHEMBL517671            0.817073      23.0        *
        pubchem   CHEMBL492266            0.880829       0.6        *
      cdk_maccs  CHEMBL1427458            0.800000    4700.0        *
   klekota_roth   CHEMBL252535            0.583333       2.2
     *** ic50 ACTIVITY *** value prediction
     based on 9 locally validated fgps ---> 4528.984
["substructure", "Pattern", "AP_bits", "MFP1", "pubchem", "graph", "FP2",
"FeatMFP1", "hybridization"]

3 rank
Rho-associated protein kinase 1 : Homo sapiens
     score: 7.466 on CHEMBL3231 based on 10 fingerprints
Fingerprint type         Ligand  Tanimoto Similarity  activity Valid fg
       FeatMFP1  CHEMBL3218282            0.772727      30.0        *
           MFP1  CHEMBL3218282            0.696970      30.0        *
         RDKit7  CHEMBL1923173            0.547750       2.0
        Pattern   CHEMBL598336            0.854396    4300.0        *
        AP_bits  CHEMBL1923173            0.652330       2.0        *
        TT_bits  CHEMBL3218282            0.518519      30.0        *
            FP2  CHEMBL1923167            0.708738      11.0        *
  hybridization  CHEMBL1923167            0.644689      11.0        *
   substructure  CHEMBL1923163            0.937500    1200.0        *
          graph  CHEMBL1923052            0.670000    1900.0
        pubchem   CHEMBL598336            0.875000    4300.0        *
      cdk_maccs  CHEMBL1923167            0.697674      11.0
```

```
   klekota_roth  CHEMBL1923181             0.804878       6.0        *
      *** ic50 ACTIVITY *** value prediction
      based on 10 locally validated fgps ---> 1103.528
["TT_bits", "substructure", "Pattern", "AP_bits", "MFP1", "pubchem",
"FP2", "FeatMFP1", "klekota_roth", "hybridization"]

4 rank
Acetyl-CoA carboxylase 2 : Homo sapiens
      score: 7.156 on CHEMBL4829 based on 9 fingerprints
Fingerprint type         Ligand  Tanimoto Similarity  activity Valid fg
       FeatMFP1  CHEMBL3690672             0.800000     530.0        *
           MFP1   CHEMBL576037             0.714286     373.0        *
         RDKit7   CHEMBL568145             0.524862     609.0
        Pattern   CHEMBL576037             0.913174     373.0        *
        AP_bits   CHEMBL567936             0.461300      33.0
        TT_bits   CHEMBL576037             0.595745     373.0        *
            FP2   CHEMBL565418             0.628866     113.0
  hybridization  CHEMBL3690674             0.478261    2700.0
   substructure  CHEMBL3659849             0.882353     530.0        *
          graph  CHEMBL3698542             0.770115    3400.0        *
        pubchem  CHEMBL3690650             0.897297     930.0        *
      cdk_maccs   CHEMBL585491             0.909091      32.0        *
   klekota_roth   CHEMBL576037             0.673469     373.0        *
      *** ic50 ACTIVITY *** value prediction
      based on 9 locally validated fgps ---> 705.977
["TT_bits", "substructure", "Pattern", "MFP1", "pubchem", "graph",
"FeatMFP1", "klekota_roth", "cdk_maccs"]

5 rank
Matrix metalloproteinase 13 : Homo sapiens
      score: 6.703 on CHEMBL280 based on 9 fingerprints
Fingerprint type         Ligand  Tanimoto Similarity  activity Valid fg
       FeatMFP1  CHEMBL1796289             0.684211  39810.72        *
           MFP1   CHEMBL605201             0.551724   1350.00        *
         RDKit7  CHEMBL1796289             0.531876  39810.72
        Pattern  CHEMBL1796282             0.940439  50118.72        *
        AP_bits  CHEMBL1796289             0.510373  39810.72        *
        TT_bits  CHEMBL1796289             0.468085  39810.72
            FP2  CHEMBL1796289             0.686047  39810.72        *
  hybridization   CHEMBL605201             0.715640   1350.00        *
   substructure   CHEMBL511942             0.933333     72.00        *
          graph   CHEMBL596494             0.700000     77.00
        pubchem  CHEMBL1427458             0.875000  10000.00        *
      cdk_maccs  CHEMBL1796287             0.806452  25118.86        *
   klekota_roth  CHEMBL1796281             0.510638  25118.86
      *** ic50 ACTIVITY *** value prediction
      based on 9 locally validated fgps ---> 18351.504
["substructure", "Pattern", "AP_bits", "MFP1", "pubchem", "FP2",
"FeatMFP1", "cdk_maccs", "hybridization"]

6 rank
Platelet activating factor receptor : Homo sapiens
      score: 6.602 on CHEMBL250 based on 8 fingerprints
Fingerprint type        Ligand  Tanimoto Similarity  activity Valid fg
       FeatMFP1  CHEMBL432324             0.809524      50.0        *
           MFP1   CHEMBL51238             0.718750      20.0        *
         RDKit7  CHEMBL412441             0.533632     280.0
        Pattern   CHEMBL51238             0.936937      20.0        *
        AP_bits  CHEMBL299760             0.457227      50.0
        TT_bits   CHEMBL51238             0.551020      20.0        *
```

```
           FP2  CHEMBL412441            0.620690    280.0
 hybridization  CHEMBL299856            0.448387     30.0
  substructure  CHEMBL432324            0.937500     50.0       *
         graph   CHEMBL99294            0.816092      1.0       *
       pubchem   CHEMBL51238            0.994286     20.0       *
     cdk_maccs   CHEMBL53014            0.837838     10.0       *
  klekota_roth   CHEMBL51238            0.524590     20.0
     *** ic50 ACTIVITY *** value prediction
     based on 8 locally validated fgps ---> 52.564
["TT_bits", "substructure", "Pattern", "MFP1", "pubchem", "graph",
"FeatMFP1", "cdk_maccs"]

7 rank
Cytochrome P450 3A4 : Homo sapiens
     score: 6.586 on CHEMBL340 based on 9 fingerprints
Fingerprint type         Ligand  Tanimoto Similarity  activity Valid fg
      FeatMFP1   CHEMBL271622            0.714286   33000.0        *
          MFP1  CHEMBL1951443            0.655172     130.0        *
        RDKit7   CHEMBL598336            0.486150    4000.0
       Pattern  CHEMBL1796293            0.884146    1000.0        *
       AP_bits  CHEMBL1951330            0.537415     200.0        *
       TT_bits  CHEMBL1951443            0.540000     130.0        *
           FP2   CHEMBL598336            0.642202    4000.0        *
 hybridization    CHEMBL10188            0.578947    4000.0
  substructure   CHEMBL271326            0.937500   34000.0        *
         graph  CHEMBL1650646            0.684783    2050.0
       pubchem  CHEMBL1427458            0.875000    1000.0        *
     cdk_maccs  CHEMBL1427458            0.800000    1000.0        *
  klekota_roth  CHEMBL2017287            0.510204    2000.0
     *** ic50 ACTIVITY *** value prediction
     based on 8 locally validated fgps ---> 6704.417
["TT_bits", "substructure", "Pattern", "AP_bits", "MFP1", "pubchem",
"FP2", "FeatMFP1"]

8 rank
Cytochrome P450 11B1 : Homo sapiens
     score: 6.164 on CHEMBL1908 based on 8 fingerprints
Fingerprint type         Ligand  Tanimoto Similarity  activity Valid fg
      FeatMFP1   CHEMBL502823            0.750000   41557.0        *
          MFP1   CHEMBL438802            0.607143   10505.0        *
        RDKit7  CHEMBL3421782            0.575472    1150.0
       Pattern  CHEMBL3421782            0.934132    1150.0        *
       AP_bits   CHEMBL500393            0.504167     151.0        *
       TT_bits  CHEMBL1173259            0.416667    3100.0
           FP2  CHEMBL3421782            0.723810    1150.0        *
 hybridization   CHEMBL176098            0.406844     206.0
  substructure  CHEMBL3421634            0.875000    1207.0        *
         graph  CHEMBL1765226            0.758621     128.0
       pubchem  CHEMBL3421781            0.912568     594.0        *
     cdk_maccs   CHEMBL502823            0.857143   41557.0        *
  klekota_roth  CHEMBL3286405            0.461538    2300.0
     *** ic50 ACTIVITY *** value prediction
     based on 8 locally validated fgps ---> 9019.047
["substructure", "Pattern", "AP_bits", "MFP1", "pubchem", "FP2",
"FeatMFP1", "cdk_maccs"]

9 rank
Cytochrome P450 11B2 : Homo sapiens
     score: 6.164 on CHEMBL2722 based on 8 fingerprints
Fingerprint type         Ligand  Tanimoto Similarity  activity Valid fg
```

```
       FeatMFP1   CHEMBL502823             0.750000      94.0        *
           MFP1   CHEMBL438802             0.607143      72.0        *
         RDKit7  CHEMBL3421782             0.575472     816.0
        Pattern  CHEMBL3421782             0.934132     816.0        *
        AP_bits   CHEMBL500393             0.504167       4.8        *
        TT_bits  CHEMBL1173259             0.416667    3450.0
            FP2  CHEMBL3421782             0.723810     816.0        *
  hybridization   CHEMBL175979             0.411067      22.0
   substructure  CHEMBL3421634             0.875000     756.0        *
          graph  CHEMBL1765226             0.758621       2.4
        pubchem  CHEMBL3421781             0.912568     299.0        *
      cdk_maccs   CHEMBL502823             0.857143      94.0        *
   klekota_roth  CHEMBL3286405             0.461538       5.0
      *** ic50 ACTIVITY *** value prediction
      based on 8 locally validated fgps ---> 352.011
["substructure", "Pattern", "AP_bits", "MFP1", "pubchem", "FP2",
"FeatMFP1", "cdk_maccs"]

10 rank
Gamma-secretase : Homo sapiens
      score: 5.520 on CHEMBL2094135 based on 7 fingerprints
Fingerprint type         Ligand  Tanimoto Similarity  activity Valid fg
       FeatMFP1  CHEMBL2415087             0.666667     158.0
           MFP1  CHEMBL3086721             0.625000     150.0        *
         RDKit7  CHEMBL3087026             0.549142    2120.0
        Pattern  CHEMBL3087026             0.876106    2120.0        *
        AP_bits  CHEMBL3086738             0.616935    6000.0        *
        TT_bits  CHEMBL2018334             0.431034    2600.0
            FP2  CHEMBL3087026             0.576577    2120.0
  hybridization  CHEMBL3792849             0.528455   95000.0
   substructure  CHEMBL2415087             0.937500     158.0        *
          graph  CHEMBL3086721             0.674699     150.0
        pubchem  CHEMBL3086721             0.857143     150.0        *
      cdk_maccs  CHEMBL3086721             0.911765     150.0        *
   klekota_roth  CHEMBL2018334             0.695652    2600.0        *
      *** ic50 ACTIVITY *** value prediction
      based on 7 locally validated fgps ---> 1832.581
["substructure", "Pattern", "MFP1", "pubchem", "AP_bits", "klekota_roth",
"cdk_maccs"]

11 rank
Sentrin-specific protease 7 : Homo sapiens
      score: 5.479 on CHEMBL1741213 based on 7 fingerprints
Fingerprint type         Ligand  Tanimoto Similarity  activity Valid fg
       FeatMFP1  CHEMBL1387142             0.636364    4980.0
           MFP1  CHEMBL1475973             0.571429    5270.0        *
         RDKit7  CHEMBL1573380             0.392744    5620.0
        Pattern  CHEMBL1475973             0.879880    5270.0        *
        AP_bits  CHEMBL1387142             0.592742    4980.0        *
        TT_bits  CHEMBL1578879             0.479167    2680.0
            FP2  CHEMBL1573380             0.567010    5620.0
  hybridization  CHEMBL1319034             0.484496    6130.0
   substructure  CHEMBL1507938             0.933333    3980.0        *
          graph  CHEMBL1578879             0.780488    2680.0        *
        pubchem  CHEMBL1387142             0.892473    4980.0        *
      cdk_maccs  CHEMBL1387142             0.828571    4980.0        *
   klekota_roth  CHEMBL1469986             0.454545   18400.0
      *** ic50 ACTIVITY *** value prediction
      based on 7 locally validated fgps ---> 4170.557
```

```
["substructure", "Pattern", "AP_bits", "MFP1", "pubchem", "graph",
"cdk_maccs"]

12 rank
Caspase-3 : Homo sapiens
      score: 5.479 on CHEMBL2334 based on 7 fingerprints
Fingerprint type         Ligand  Tanimoto Similarity  activity Valid fg
       FeatMFP1  CHEMBL1387142             0.636364   17500.0
           MFP1  CHEMBL1475973             0.571429   30300.0        *
         RDKit7  CHEMBL1573380             0.392744   21800.0
        Pattern  CHEMBL1475973             0.879880   30300.0        *
        AP_bits  CHEMBL1387142             0.592742   17500.0        *
        TT_bits  CHEMBL1578879             0.479167    5560.0
            FP2  CHEMBL1573380             0.567010   21800.0
  hybridization   CHEMBL205983             0.584000    4630.0
   substructure  CHEMBL1507938             0.933333   15600.0        *
          graph  CHEMBL1578879             0.780488    5560.0        *
        pubchem  CHEMBL1387142             0.892473   17500.0        *
      cdk_maccs  CHEMBL1387142             0.828571   17500.0        *
   klekota_roth  CHEMBL2391777             0.471698      85.0
      *** ic50 ACTIVITY *** value prediction
      based on 7 locally validated fgps ---> 14761.051
["substructure", "Pattern", "AP_bits", "MFP1", "pubchem", "graph",
"cdk_maccs"]

13 rank
Poly [ADP-ribose] polymerase-1 : Homo sapiens
      score: 5.467 on CHEMBL3105 based on 7 fingerprints
Fingerprint type         Ligand  Tanimoto Similarity  activity Valid fg
       FeatMFP1   CHEMBL480233             0.714286   62000.0        *
           MFP1   CHEMBL123978             0.500000   17000.0
         RDKit7  CHEMBL3105430             0.412162     820.0
        Pattern   CHEMBL480710             0.894895      18.0        *
        AP_bits   CHEMBL501290             0.481356    4533.0        *
        TT_bits   CHEMBL480233             0.396226   62000.0
            FP2   CHEMBL480233             0.514019   62000.0
  hybridization   CHEMBL594596             0.516432     540.0
   substructure  CHEMBL3589258             0.866667   43000.0        *
          graph   CHEMBL294995             0.809524     184.0        *
        pubchem   CHEMBL480233             0.842932   62000.0        *
      cdk_maccs   CHEMBL480233             0.857143   62000.0        *
   klekota_roth  CHEMBL3827329             0.444444    2404.0
      *** ic50 ACTIVITY *** value prediction
      based on 7 locally validated fgps ---> 24685.036
["substructure", "Pattern", "AP_bits", "pubchem", "graph", "FeatMFP1",
"cdk_maccs"]

14 rank
Luciferase : Luciola lateralis
      score: 5.325 on CHEMBL3701 based on 7 fingerprints
Fingerprint type        Ligand  Tanimoto Similarity  activity Valid fg
       FeatMFP1  CHEMBL467971             0.750000     310.0        *
           MFP1  CHEMBL467971             0.586207     310.0        *
         RDKit7  CHEMBL467971             0.409731     310.0
        Pattern  CHEMBL467971             0.852410     310.0        *
        AP_bits  CHEMBL467971             0.496124     310.0        *
        TT_bits  CHEMBL467971             0.384615     310.0
            FP2  CHEMBL467971             0.460938     310.0
  hybridization  CHEMBL467971             0.295547     310.0
   substructure  CHEMBL405914             0.937500     610.0        *
```

```
          graph  CHEMBL467971             0.567308     310.0
        pubchem  CHEMBL467971             0.869110     310.0        *
      cdk_maccs  CHEMBL467971             0.833333     310.0        *
   klekota_roth  CHEMBL467971             0.372093     310.0
      *** ic50 ACTIVITY *** value prediction
      based on 7 locally validated fgps ---> 755.039
["substructure", "Pattern", "MFP1", "pubchem", "AP_bits", "FeatMFP1",
"cdk_maccs"]

15 rank
Vascular endothelial growth factor receptor 2 : Homo sapiens
      score: 5.314 on CHEMBL279 based on 7 fingerprints
Fingerprint type        Ligand  Tanimoto Similarity    activity Valid fg
       FeatMFP1  CHEMBL269871             0.727273      1.2880        *
           MFP1  CHEMBL408211             0.606061      5.0000        *
         RDKit7  CHEMBL271271             0.481956  16000.0000
        Pattern  CHEMBL427725             0.866667    200.0000        *
        AP_bits  CHEMBL518652             0.517460    110.0000        *
        TT_bits  CHEMBL526639             0.403846    500.0000
            FP2  CHEMBL270342             0.558824      0.4467
  hybridization  CHEMBL184223             0.619926     21.0000
   substructure  CHEMBL269871             0.937500      1.2880        *
          graph  CHEMBL452812             0.656250  13000.0000
        pubchem  CHEMBL245745             0.830918      5.0000        *
      cdk_maccs  CHEMBL272252             0.828571      1.2000        *
   klekota_roth  CHEMBL271271             0.500000  16000.0000
      *** ic50 ACTIVITY *** value prediction
      based on 7 locally validated fgps ---> 1281.064
["substructure", "Pattern", "MFP1", "pubchem", "AP_bits", "FeatMFP1",
"cdk_maccs"]

16 rank
HERG : Homo sapiens
      score: 5.299 on CHEMBL240 based on 7 fingerprints
Fingerprint type         Ligand  Tanimoto Similarity  activity Valid fg
       FeatMFP1  CHEMBL1671897             0.727273  10000.00        *
           MFP1  CHEMBL1779014             0.514286  18800.00
         RDKit7  CHEMBL1671897             0.437204  10000.00
        Pattern  CHEMBL1779014             0.911504  18800.00        *
        AP_bits  CHEMBL3185736             0.470383  12400.00        *
        TT_bits  CHEMBL2069407             0.370968   2511.89
            FP2   CHEMBL271801             0.594340    363.08
  hybridization  CHEMBL3099899             0.630435   6950.00        *
   substructure  CHEMBL1671897             0.875000  10000.00        *
          graph  CHEMBL1671885             0.772727    100.00        *
        pubchem  CHEMBL1671897             0.912088  10000.00        *
      cdk_maccs  CHEMBL1779014             0.714286  18800.00
   klekota_roth  CHEMBL2069416             0.444444   3981.07
      *** ic50 ACTIVITY *** value prediction
      based on 7 locally validated fgps ---> 12025.348
["substructure", "Pattern", "AP_bits", "pubchem", "graph", "FeatMFP1",
"hybridization"]

17 rank
Serine/threonine-protein kinase Aurora-B : Homo sapiens
      score: 5.290 on CHEMBL2185 based on 7 fingerprints
Fingerprint type         Ligand  Tanimoto Similarity  activity Valid fg
       FeatMFP1   CHEMBL269871             0.727273     46.00        *
           MFP1   CHEMBL408211             0.606061    190.00        *
         RDKit7  CHEMBL3343058             0.362220  18100.00
```

```
        Pattern  CHEMBL3343044            0.848901   5910.00        *
        AP_bits   CHEMBL382590            0.493976   1250.00        *
        TT_bits  CHEMBL3680499            0.424242      0.94
            FP2   CHEMBL408211            0.542857    190.00
  hybridization   CHEMBL452812            0.498246   4900.00
   substructure   CHEMBL269871            0.937500     46.00        *
          graph   CHEMBL388956            0.715789   2140.00
        pubchem  CHEMBL3670416            0.847716      8.20        *
      cdk_maccs   CHEMBL272252            0.828571    130.00        *
   klekota_roth   CHEMBL408211            0.473684    190.00
      *** ic50 ACTIVITY *** value prediction
      based on 7 locally validated fgps ---> 2157.602
["substructure", "Pattern", "MFP1", "pubchem", "AP_bits", "FeatMFP1",
"cdk_maccs"]

18 rank
Mitochondrial import inner membrane translocase subunit TIM10 :
Saccharomyces cerevisiae S288c
      score: 5.240 on CHEMBL1741194 based on 7 fingerprints
Fingerprint type         Ligand  Tanimoto Similarity  activity Valid fg
       FeatMFP1  CHEMBL1467168            0.600000   17200.0
           MFP1  CHEMBL1389690            0.592593   24400.0        *
         RDKit7   CHEMBL438729            0.547382   47300.0
        Pattern  CHEMBL1475973            0.879880   27300.0        *
        AP_bits  CHEMBL1475973            0.496479   27300.0        *
        TT_bits  CHEMBL1398317            0.361702    3420.0
            FP2   CHEMBL438729            0.633929   47300.0        *
  hybridization  CHEMBL1363382            0.536765    2890.0
   substructure  CHEMBL1482509            1.000000   22800.0        *
          graph  CHEMBL1336269            0.780488    6800.0        *
        pubchem  CHEMBL1482509            0.856410   22800.0        *
      cdk_maccs  CHEMBL1389690            0.727273   24400.0
   klekota_roth  CHEMBL1389690            0.431818   24400.0
      *** ic50 ACTIVITY *** value prediction
      based on 7 locally validated fgps ---> 22781.988
["substructure", "Pattern", "AP_bits", "MFP1", "pubchem", "graph", "FP2"]

19 rank
Acetyl-CoA carboxylase 1 : Homo sapiens
      score: 5.197 on CHEMBL3351 based on 7 fingerprints
Fingerprint type         Ligand  Tanimoto Similarity  activity Valid fg
       FeatMFP1   CHEMBL566681            0.727273     415.0        *
           MFP1   CHEMBL585885            0.642857      41.0        *
         RDKit7   CHEMBL568145            0.524862      36.0
        Pattern   CHEMBL567934            0.896450      81.0        *
        AP_bits   CHEMBL567936            0.461300      17.0
        TT_bits   CHEMBL568145            0.543860      36.0        *
            FP2   CHEMBL565418            0.628866      35.0
  hybridization   CHEMBL566024            0.435737      35.0
   substructure  CHEMBL1630706            0.812500   14600.0
          graph   CHEMBL566025            0.600000    2770.0
        pubchem   CHEMBL566862            0.885870      69.0        *
      cdk_maccs   CHEMBL585491            0.909091      49.0        *
   klekota_roth   CHEMBL585491            0.591837      49.0        *
      *** ic50 ACTIVITY *** value prediction
      based on 7 locally validated fgps ---> 1602.694
["TT_bits", "Pattern", "MFP1", "pubchem", "klekota_roth", "FeatMFP1",
"cdk_maccs"]

20 rank
```

```
Epidermal growth factor receptor erbB1 : Homo sapiens
      score: 5.195 on CHEMBL203 based on 7 fingerprints
Fingerprint type         Ligand  Tanimoto Similarity  activity Valid fg
       FeatMFP1   CHEMBL143968            0.619048   1100.00
           MFP1  CHEMBL3753836            0.531250    131.09         *
         RDKit7   CHEMBL143160            0.559006   5400.00
        Pattern   CHEMBL142831            0.846154  10000.00         *
        AP_bits   CHEMBL186802            0.529595   5600.00         *
        TT_bits    CHEMBL55979            0.415094    400.00
            FP2   CHEMBL143160            0.677778   5400.00         *
  hybridization   CHEMBL143160            0.608491   5400.00
   substructure   CHEMBL186747            1.000000   3200.00         *
          graph  CHEMBL2336357            0.780488   9050.00         *
        pubchem   CHEMBL148514            0.830000  30500.00         *
      cdk_maccs  CHEMBL2336357            0.705882   9050.00
   klekota_roth  CHEMBL3758278            0.450980      2.00
      *** ic50 ACTIVITY *** value prediction
      based on 6 locally validated fgps ---> 12761.728
["substructure", "Pattern", "AP_bits", "MFP1", "graph", "FP2"]

21 rank
Signal transducer and activator of transcription 3 : Homo sapiens
      score: 5.180 on CHEMBL4026 based on 7 fingerprints
Fingerprint type         Ligand  Tanimoto Similarity  activity Valid fg
       FeatMFP1  CHEMBL1530735            0.636364    2106.0
           MFP1  CHEMBL1447188            0.545455   16420.0         *
         RDKit7  CHEMBL1478316            0.396197    2157.0
        Pattern  CHEMBL1427458            0.883281    5360.0         *
        AP_bits  CHEMBL1353939            0.500000    1298.0         *
        TT_bits  CHEMBL1478316            0.478261    2157.0
            FP2  CHEMBL1427458            0.638298    5360.0         *
  hybridization  CHEMBL3133457            0.517606   52000.0
   substructure  CHEMBL1530735            0.937500    2106.0         *
          graph  CHEMBL3133461            0.738636   33300.0
        pubchem  CHEMBL1427458            0.875000    5360.0         *
      cdk_maccs  CHEMBL1427458            0.800000    5360.0         *
   klekota_roth  CHEMBL1339432            0.362069    3832.0
      *** ic50 ACTIVITY *** value prediction
      based on 6 locally validated fgps ---> 6426.275
["substructure", "Pattern", "AP_bits", "MFP1", "pubchem", "FP2"]

22 rank
Vanilloid receptor : Homo sapiens
      score: 5.174 on CHEMBL4794 based on 7 fingerprints
Fingerprint type         Ligand  Tanimoto Similarity  activity Valid fg
       FeatMFP1   CHEMBL214274            0.625000      62.0
           MFP1   CHEMBL483866            0.629630     147.0         *
         RDKit7  CHEMBL3769572            0.468333    7110.0
        Pattern   CHEMBL249943            0.855114     680.0         *
        AP_bits   CHEMBL483866            0.567227     147.0         *
        TT_bits   CHEMBL483866            0.510638     147.0         *
            FP2   CHEMBL400880            0.622807     370.0
  hybridization  CHEMBL3769572            0.491379    7110.0
   substructure   CHEMBL215015            0.933333    2585.0         *
          graph   CHEMBL195257            0.780488     140.0         *
        pubchem   CHEMBL215439            0.897297     974.0         *
      cdk_maccs   CHEMBL483866            0.735294     147.0
   klekota_roth   CHEMBL231392            0.530612     749.0
      *** ic50 ACTIVITY *** value prediction
      based on 7 locally validated fgps ---> 1011.643
```

["TT_bits", "substructure", "Pattern", "AP_bits", "MFP1", "pubchem", "graph"]

23 rank
Estradiol 17-beta-dehydrogenase 2 : Homo sapiens
score: 5.129 on CHEMBL2789 based on 7 fingerprints

Fingerprint type	Ligand	Tanimoto Similarity	activity	Valid fg
FeatMFP1	CHEMBL3289858	0.666667	621.0	
MFP1	CHEMBL3645224	0.607143	601.0	*
RDKit7	CHEMBL1915937	0.645161	265.0	
Pattern	CHEMBL1915936	0.903323	594.0	*
AP_bits	CHEMBL1915937	0.538732	265.0	*
TT_bits	CHEMBL3645223	0.416667	1760.0	
FP2	CHEMBL1915935	0.744186	494.0	*
hybridization	CHEMBL1915937	0.693694	265.0	*
substructure	CHEMBL2324690	0.764706	58.0	
graph	CHEMBL1915935	0.829268	494.0	*
pubchem	CHEMBL1097417	0.823204	2970.0	
cdk_maccs	CHEMBL1097417	0.812500	2970.0	*
klekota_roth	CHEMBL1915935	0.431373	494.0	

*** ic50 ACTIVITY *** value prediction
based on 7 locally validated fgps ---> 875.823
["Pattern", "AP_bits", "MFP1", "graph", "FP2", "cdk_maccs", "hybridization"]

24 rank
Cytochrome P450 2C9 : Homo sapiens
score: 5.020 on CHEMBL3397 based on 7 fingerprints

Fingerprint type	Ligand	Tanimoto Similarity	activity	Valid fg
FeatMFP1	CHEMBL271622	0.714286	39000.0	*
MFP1	CHEMBL598336	0.562500	16000.0	*
RDKit7	CHEMBL598336	0.486150	16000.0	
Pattern	CHEMBL560537	0.855799	30000.0	*
AP_bits	CHEMBL2332097	0.488294	600.0	*
TT_bits	CHEMBL485968	0.388889	3000.0	
FP2	CHEMBL598336	0.642202	16000.0	*
hybridization	CHEMBL10188	0.578947	2000.0	
substructure	CHEMBL2348850	0.882353	16000.0	*
graph	CHEMBL1650646	0.684783	224.0	
pubchem	CHEMBL598336	0.875000	16000.0	*
cdk_maccs	CHEMBL3822473	0.756757	5030.0	
klekota_roth	CHEMBL2017287	0.510204	1300.0	

*** ic50 ACTIVITY *** value prediction
based on 7 locally validated fgps ---> 15476.314
["substructure", "Pattern", "AP_bits", "MFP1", "pubchem", "FP2", "FeatMFP1"]

SwissTargetPrediction report:

Reference:
Gfeller D., Michielin O. & Zoete V.
Shaping the interaction landscape of bioactive molecules, *Bioinformatics* (2013) 29:3073-3079.

Query Molecule

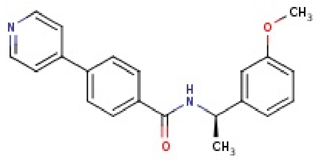

Frequency of Target Class

Target	Uniprot ID	Gene code	ChEMBL ID	Probability	# sim. cmpds (3D / 2D)	Target Class
Corticosteroid 11-beta-dehydrogenase isozyme 2	P80365	HSD11B2	CHEMBL3746		39 / 16	Enzyme
Rho-associated protein kinase 2	O75116	ROCK2	CHEMBL2973		159 / 41	Ser_Thr Kinase
Rho-associated protein kinase 1	Q13464	ROCK1	CHEMBL3231		159 / 41	Ser_Thr Kinase
Estradiol 17-beta-dehydrogenase 2	P37059	HSD17B2	CHEMBL2789		13 / 16	Enzyme
Epidermal growth factor receptor	P00533	EGFR	CHEMBL203		156 / 10	Tyr Kinase
Receptor tyrosine-protein kinase erbB-2	P04626	ERBB2	CHEMBL1824		156 / 9	Tyr Kinase
Receptor tyrosine-protein kinase erbB-3 *(by homology)*	P21860	ERBB3	CHEMBL5838		156 / 9	Tyr Kinase
ERBB4 intracellular domain *(by homology)*	Q15303	ERBB4	CHEMBL3009		156 / 9	Tyr Kinase
Mitogen-activated protein kinase 11 *(by homology)*	Q15759	MAPK11	CHEMBL3961		435 / 48	Ser_Thr Kinase
Mitogen-activated protein kinase 14	Q16539	MAPK14	CHEMBL260		435 / 48	Ser_Thr Kinase
Muscleblind-like protein 1	Q9NR56	MBNL1	CHEMBL1293317		709 / 67	Unclassified
Muscleblind-like protein 2 *(by homology)*	Q5VZF2	MBNL2			709 / 67	Unclassified
Muscleblind-like protein 3 *(by homology)*	Q9NUK0	MBNL3			709 / 67	Unclassified
Mitogen-activated protein kinase 8 *(by homology)*	P45983	MAPK8	CHEMBL2276		425 / 48	Ser_Thr Kinase
Mitogen-activated protein kinase 9 *(by homology)*	P45984	MAPK9	CHEMBL4179		425 / 48	Ser_Thr Kinase

Polypharmacology Browser 2 Prediction:

Targets predicted using NN(ECfp4) + NB(ECfp4). Save Table

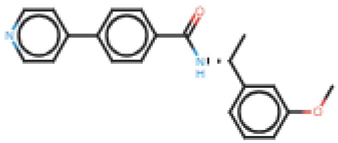

Query molecule

Target class overview

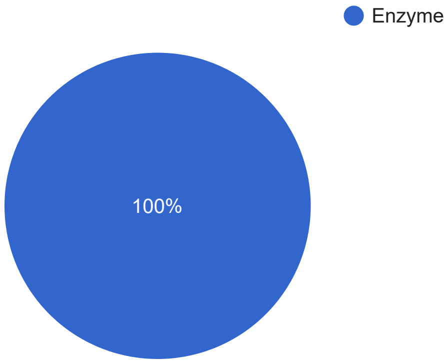

Rank	ChEMBL ID	Common name	Nearest neighbours
1	CHEMBL3231 (https://www.ebi.ac.uk/chembl/target/inspect/CHEMBL3231)	Rho-associated protein kinase 1	Show NN
2	CHEMBL262 (https://www.ebi.ac.uk/chembl/target/inspect/CHEMBL262)	Glycogen synthase kinase-3 beta	Show NN
3	CHEMBL4101 (https://www.ebi.ac.uk/chembl/target/inspect/CHEMBL4101)	cAMP-dependent protein kinase alpha-catalytic subunit	Show NN
4	CHEMBL2850 (https://www.ebi.ac.uk/chembl/target/inspect/CHEMBL2850)	Glycogen synthase kinase-3 alpha	Show NN
5	CHEMBL4203 (https://www.ebi.ac.uk/chembl/target/inspect/CHEMBL4203)	Dual specificity protein kinase CLK4	Show NN
6	CHEMBL2973 (https://www.ebi.ac.uk/chembl/target/inspect/CHEMBL2973)	Rho-associated protein kinase 2	Show NN
7	CHEMBL5818 (https://www.ebi.ac.uk/chembl/target/inspect/CHEMBL5818)	Serine/threonine-protein kinase PRKX	Show NN
8	CHEMBL3032 (https://www.ebi.ac.uk/chembl/target/inspect/CHEMBL3032)	Protein kinase N2	Show NN
9	CHEMBL4273 (https://www.ebi.ac.uk/chembl/target/inspect/CHEMBL4273)	cGMP-dependent protein kinase 1 beta	Show NN
10	CHEMBL4040 (https://www.ebi.ac.uk/chembl/target/inspect/CHEMBL4040)	MAP kinase ERK2	Show NN
11	CHEMBL5794 (https://www.ebi.ac.uk/chembl/target/inspect/CHEMBL5794)	Serine/threonine-protein kinase Sgk2	Show NN
12	CHEMBL4501 (https://www.ebi.ac.uk/chembl/target/inspect/CHEMBL4501)	Ribosomal protein S6 kinase 1	Show NN
13	CHEMBL2996 (https://www.ebi.ac.uk/chembl/target/inspect/CHEMBL2996)	Protein kinase C delta	Show NN

14	CHEMBL301 (https://www.ebi.ac.uk/chembl/target/inspect/CHEMBL301)	Cyclin-dependent kinase 2	Show NN
15	CHEMBL2896 (https://www.ebi.ac.uk/chembl/target/inspect/CHEMBL2896)	cGMP-dependent protein kinase 2	Show NN
16	CHEMBL4516 (https://www.ebi.ac.uk/chembl/target/inspect/CHEMBL4516)	Serine/threonine-protein kinase MRCK-A	Show NN
17	CHEMBL2345 (https://www.ebi.ac.uk/chembl/target/inspect/CHEMBL2345)	Ribosomal protein S6 kinase alpha 3	Show NN
18	CHEMBL4282 (https://www.ebi.ac.uk/chembl/target/inspect/CHEMBL4282)	Serine/threonine-protein kinase AKT	Show NN
19	CHEMBL2938 (https://www.ebi.ac.uk/chembl/target/inspect/CHEMBL2938)	Protein kinase C gamma	Show NN
20	CHEMBL6166 (https://www.ebi.ac.uk/chembl/target/inspect/CHEMBL6166)	Mitogen-activated protein kinase kinase kinase kinase 4	Show NN
