# Supplementary material for: Accelerating Drug Discovery by Early Protein Drug Target Prediction Based on a Multi-Fingerprint Similarity Search
Source: Molecules. 2019 Jun 14;24(12):2233. doi: 10.3390/molecules24122233 (PMC6631269; doi:10.3390/molecules24122233)
Supplement: Supplementary file 1 [file molecules-24-02233-s001.zip › File_S2.zip!/File_S2/Target_Prediction_compound#13.pdf]

MuSSel Prediction k_i:

```
1 rank
Dopamine D2 receptor : Homo sapiens
     score: 6.431 on CHEMBL217 based on 8 fingerprints
Fingerprint type         Ligand  Tanimoto Similarity  activity Valid fg
       FeatMFP1   CHEMBL143027             0.913043     16.22        *
           MFP1  CHEMBL3104647             0.636364     46.77        *
         RDKit7  CHEMBL3104516             0.669746     50.12
        Pattern  CHEMBL3104517             0.815592     17.38
        AP_bits  CHEMBL3220218             0.541578    199.53        *
        TT_bits  CHEMBL3104647             0.511628     46.77
            FP2  CHEMBL3104516             0.653409     50.12
  hybridization       CHEMBL85             0.570016      0.44
   substructure   CHEMBL155486             0.954545     57.54        *
          graph  CHEMBL2207489             0.913043     34.00        *
        pubchem   CHEMBL198599             0.858586     35.00        *
      cdk_maccs  CHEMBL3104516             0.921875     50.12        *
   klekota_roth  CHEMBL2059305             0.691489     42.00        *
     *** ki ACTIVITY *** value prediction
     based on 8 locally validated fgps ---> 57.147
["substructure", "AP_bits", "MFP1", "pubchem", "graph", "FeatMFP1",
"klekota_roth", "cdk_maccs"]

2 rank
Serotonin 2a (5-HT2a) receptor : Rattus norvegicus
     score: 5.897 on CHEMBL322 based on 7 fingerprints
Fingerprint type         Ligand  Tanimoto Similarity  activity Valid fg
       FeatMFP1   CHEMBL110948             0.913043     2.754        *
           MFP1  CHEMBL3740807             0.666667    19.000        *
         RDKit7   CHEMBL155486             0.649260     2.692
        Pattern   CHEMBL333174             0.822767     1.585
        AP_bits  CHEMBL3120699             0.635220   125.600        *
        TT_bits  CHEMBL3740807             0.523810    19.000
            FP2  CHEMBL3742070             0.642045    26.900
  hybridization  CHEMBL3634824             0.588869    38.100
   substructure   CHEMBL155486             0.954545     2.692        *
          graph  CHEMBL3741516             0.924370  2115.000        *
        pubchem  CHEMBL3741596             0.896739   113.800        *
      cdk_maccs   CHEMBL143027             0.906250     5.495        *
   klekota_roth  CHEMBL3742362             0.561905   215.600
     *** ki ACTIVITY *** value prediction
     based on 7 locally validated fgps ---> 193.388
["substructure", "AP_bits", "MFP1", "pubchem", "graph", "FeatMFP1",
"cdk_maccs"]

3 rank
Dopamine D2 receptor : Rattus norvegicus
     score: 5.855 on CHEMBL339 based on 7 fingerprints
Fingerprint type         Ligand  Tanimoto Similarity  activity Valid fg
       FeatMFP1   CHEMBL143027             0.913043      9.55        *
           MFP1  CHEMBL3740807             0.666667     86.80        *
         RDKit7  CHEMBL3233412             0.662634    192.00
        Pattern   CHEMBL333174             0.822767    141.25
        AP_bits  CHEMBL3120699             0.635220    623.80        *
        TT_bits  CHEMBL3740807             0.523810     86.80
            FP2  CHEMBL3233412             0.644330    192.00
  hybridization  CHEMBL3634824             0.588869     53.10
   substructure   CHEMBL143027             0.913043      9.55        *
          graph  CHEMBL3741596             0.924370   1273.80        *
        pubchem  CHEMBL3741596             0.896739   1273.80        *
      cdk_maccs   CHEMBL143027             0.906250      9.55        *
```

```
   klekota_roth  CHEMBL3233427             0.578431     27.00
      *** ki ACTIVITY *** value prediction
      based on 7 locally validated fgps ---> 375.772
["substructure", "AP_bits", "MFP1", "pubchem", "graph", "FeatMFP1",
"cdk_maccs"]

4 rank
Serotonin 1a (5-HT1a) receptor : Rattus norvegicus
      score: 5.788 on CHEMBL273 based on 7 fingerprints
Fingerprint type         Ligand  Tanimoto Similarity  activity Valid fg
       FeatMFP1  CHEMBL3740807             0.869565     156.9        *
           MFP1  CHEMBL3740807             0.666667     156.9        *
         RDKit7  CHEMBL3742070             0.630400     276.5
        Pattern  CHEMBL2387233             0.812139    3071.6
        AP_bits  CHEMBL3120688             0.628998      47.5        *
        TT_bits  CHEMBL3740807             0.523810     156.9
            FP2  CHEMBL3742070             0.642045     276.5
  hybridization  CHEMBL3634824             0.588869      31.1
   substructure  CHEMBL3740807             0.913043     156.9        *
          graph  CHEMBL3741596             0.924370     598.9        *
        pubchem  CHEMBL3741596             0.896739     598.9        *
      cdk_maccs  CHEMBL3740807             0.888889     156.9        *
   klekota_roth   CHEMBL314430             0.575758     250.0
      *** ki ACTIVITY *** value prediction
      based on 7 locally validated fgps ---> 352.759
["substructure", "AP_bits", "MFP1", "pubchem", "graph", "FeatMFP1",
"cdk_maccs"]

5 rank
Serotonin 2a (5-HT2a) receptor : Homo sapiens
      score: 5.717 on CHEMBL224 based on 7 fingerprints
Fingerprint type         Ligand  Tanimoto Similarity  activity Valid fg
       FeatMFP1   CHEMBL143027             0.913043     5.495        *
           MFP1  CHEMBL3104647             0.636364     5.012        *
         RDKit7  CHEMBL3104516             0.669746    31.620
        Pattern   CHEMBL333174             0.822767     1.175
        AP_bits  CHEMBL3220218             0.541578    19.950        *
        TT_bits  CHEMBL3104647             0.511628     5.012
            FP2  CHEMBL3104516             0.653409    31.620
  hybridization       CHEMBL85             0.570016     0.099
   substructure   CHEMBL155486             0.954545     2.692        *
          graph   CHEMBL155486             0.890756     2.692        *
        pubchem   CHEMBL198599             0.858586     0.280        *
      cdk_maccs  CHEMBL3104516             0.921875    31.620        *
   klekota_roth   CHEMBL163904             0.612245     1.200
      *** ki ACTIVITY *** value prediction
      based on 7 locally validated fgps ---> 10.793
["substructure", "AP_bits", "MFP1", "pubchem", "graph", "FeatMFP1",
"cdk_maccs"]

6 rank
Serotonin 2c (5-HT2c) receptor : Homo sapiens
      score: 5.665 on CHEMBL225 based on 7 fingerprints
Fingerprint type         Ligand  Tanimoto Similarity  activity Valid fg
       FeatMFP1   CHEMBL110948             0.913043    125.89        *
           MFP1   CHEMBL111352             0.600000     69.18        *
         RDKit7  CHEMBL3233412             0.662634    300.00
        Pattern   CHEMBL333174             0.822767     27.54
        AP_bits   CHEMBL111352             0.544393     69.18        *
        TT_bits   CHEMBL421801             0.483516    512.86
```

```
           FP2  CHEMBL3233412             0.644330    300.00
  hybridization       CHEMBL85            0.570016      6.40
   substructure   CHEMBL155486            0.954545    104.71        *
          graph   CHEMBL155486            0.890756    104.71        *
        pubchem   CHEMBL155486            0.856383    104.71        *
      cdk_maccs   CHEMBL143027            0.906250    338.84        *
   klekota_roth   CHEMBL163904            0.612245     71.00
      *** ki ACTIVITY *** value prediction
      based on 7 locally validated fgps ---> 193.629
["substructure", "AP_bits", "MFP1", "pubchem", "graph", "FeatMFP1",
"cdk_maccs"]

7 rank
Dopamine D1 receptor : Rattus norvegicus
      score: 3.900 on CHEMBL265 based on 5 fingerprints
Fingerprint type        Ligand  Tanimoto Similarity  activity Valid fg
       FeatMFP1  CHEMBL143027            0.913043   28.8400        *
           MFP1  CHEMBL541842            0.600000  134.9000        *
         RDKit7  CHEMBL539544            0.616715  128.8200
        Pattern  CHEMBL393466            0.773533    0.6427
        AP_bits  CHEMBL151475            0.567227  239.0000        *
        TT_bits  CHEMBL541842            0.454545  134.9000
            FP2  CHEMBL541842            0.536585  134.9000
  hybridization      CHEMBL85            0.570016   22.0000
   substructure  CHEMBL143027            0.913043   28.8400        *
          graph  CHEMBL143027            0.727891   28.8400
        pubchem      CHEMBL85            0.845361   22.0000
      cdk_maccs  CHEMBL143027            0.906250   28.8400        *
   klekota_roth      CHEMBL85            0.554545   22.0000
      *** ki ACTIVITY *** value prediction
      based on 5 locally validated fgps ---> 112.249
["FeatMFP1", "AP_bits", "MFP1", "substructure", "cdk_maccs"]

8 rank
Dopamine D3 receptor : Homo sapiens
      score: 3.232 on CHEMBL234 based on 4 fingerprints
Fingerprint type         Ligand  Tanimoto Similarity  activity Valid fg
       FeatMFP1   CHEMBL572708            0.875000    281.84        *
           MFP1  CHEMBL1258036            0.585366      9.90
         RDKit7   CHEMBL572708            0.603026    281.84
        Pattern  CHEMBL3676930            0.788839    145.21
        AP_bits   CHEMBL151475            0.567227      5.40        *
        TT_bits   CHEMBL566508            0.442105     44.67
            FP2   CHEMBL572708            0.579208    281.84
  hybridization       CHEMBL85            0.570016      6.70
   substructure   CHEMBL572708            0.913043    281.84        *
          graph   CHEMBL572708            0.744828    281.84
        pubchem       CHEMBL85            0.845361      6.70
      cdk_maccs   CHEMBL572708            0.876923    281.84        *
   klekota_roth  CHEMBL1256170            0.583333      4.20
      *** ki ACTIVITY *** value prediction
      based on 4 locally validated fgps ---> 168.147
["FeatMFP1", "AP_bits", "substructure", "cdk_maccs"]

9 rank
Serotonin 1a (5-HT1a) receptor : Homo sapiens
      score: 3.096 on CHEMBL214 based on 4 fingerprints
Fingerprint type         Ligand  Tanimoto Similarity  activity Valid fg
       FeatMFP1   CHEMBL246484            0.777778   1513.56        *
           MFP1       CHEMBL85            0.583333     21.00
```

RDKit7	CHEMBL3233412	0.662634	364.00	
Pattern	CHEMBL3233412	0.797844	364.00	
AP_bits	CHEMBL1774085	0.530992	1.20	*
TT_bits	CHEMBL246484	0.434783	1513.56	
FP2	CHEMBL3233412	0.644330	364.00	
hybridization	CHEMBL85	0.570016	21.00	
substructure	CHEMBL445651	0.913043	160.00	*
graph	CHEMBL445651	0.780303	160.00	
pubchem	CHEMBL445651	0.874346	160.00	*
cdk_maccs	CHEMBL246484	0.826087	1513.56	
klekota_roth	CHEMBL3233427	0.578431	206.00	

```
   *** ki ACTIVITY *** value prediction
   based on 4 locally validated fgps ---> 436.758
["FeatMFP1", "AP_bits", "substructure", "pubchem"]
```

SwissTargetPrediction report:

Reference:
Gfeller D., Michielin O. & Zoete V.
Shaping the interaction landscape of bioactive molecules, *Bioinformatics* (2013) 29:3073-3079.

Query Molecule

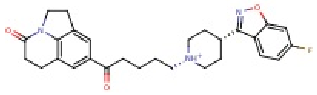

Frequency of Target Class

Target	Uniprot ID	Gene code	ChEMBL ID	Probability	# sim. cmpds (3D / 2D)	Target Class
Cholinesterase	P06276	BCHE	CHEMBL1914		187 / 68	Enzyme
Acetylcholinesterase	P22303	ACHE	CHEMBL220		187 / 68	Enzyme
D(2) dopamine receptor	P14416	DRD2	CHEMBL217		1056 / 139	Membrane receptor
5-hydroxytryptamine receptor 2A	P28223	HTR2A	CHEMBL224		385 / 47	Membrane receptor
D(3) dopamine receptor *(by homology)*	P35462	DRD3	CHEMBL234		654 / 68	Membrane receptor
5-hydroxytryptamine receptor 1A	P08908	HTR1A	CHEMBL214		420 / 19	Membrane receptor
5-hydroxytryptamine receptor 2C *(by homology)*	P28335	HTR2C	CHEMBL225		279 / 27	Membrane receptor
5-hydroxytryptamine receptor 2B *(by homology)*	P41595	HTR2B	CHEMBL1833		279 / 27	Membrane receptor
5-hydroxytryptamine receptor 1B *(by homology)*	P28222	HTR1B	CHEMBL1898		423 / 18	Membrane receptor
D(1A) dopamine receptor	P21728	DRD1	CHEMBL2056		59 / 6	Membrane receptor
5-hydroxytryptamine receptor 1D	P28221	HTR1D	CHEMBL1983		172 / 10	Membrane receptor
5-hydroxytryptamine receptor 1E *(by homology)*	P28566	HTR1E	CHEMBL2182		152 / 10	Membrane receptor
5-hydroxytryptamine receptor 1F	P30939	HTR1F	CHEMBL1805		152 / 10	Membrane receptor
D(4) dopamine receptor	P21917	DRD4	CHEMBL219		429 / 30	Membrane receptor
Mitogen-activated protein kinase 8 *(by homology)*	P45983	MAPK8	CHEMBL2276		74 / 26	Ser_Thr Kinase

Polypharmacology Browser 2 Prediction:

Targets predicted using NN(ECfp4) + NB(ECfp4). Save Table

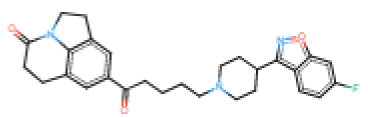

Query molecule

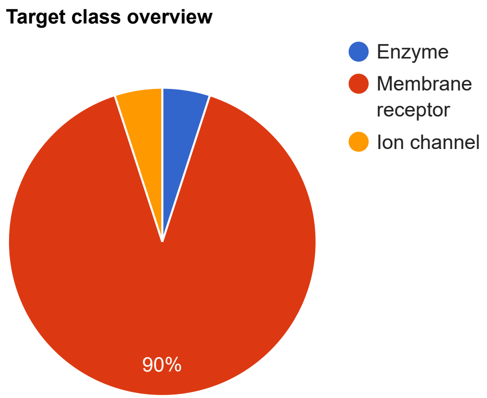

Rank	ChEMBL ID	Common name	Nearest neighbours
1	CHEMBL224 (https://www.ebi.ac.uk/chembl/target/inspect/CHEMBL224)	Serotonin 2a (5-HT2a) receptor	Show NN
2	CHEMBL217 (https://www.ebi.ac.uk/chembl/target/inspect/CHEMBL217)	Dopamine D2 receptor	Show NN
3	CHEMBL339 (https://www.ebi.ac.uk/chembl/target/inspect/CHEMBL339)	Dopamine D2 receptor	Show NN
4	CHEMBL322 (https://www.ebi.ac.uk/chembl/target/inspect/CHEMBL322)	Serotonin 2a (5-HT2a) receptor	Show NN
5	CHEMBL3371 (https://www.ebi.ac.uk/chembl/target/inspect/CHEMBL3371)	Serotonin 6 (5-HT6) receptor	Show NN
6	CHEMBL273 (https://www.ebi.ac.uk/chembl/target/inspect/CHEMBL273)	Serotonin 1a (5-HT1a) receptor	Show NN
7	CHEMBL225 (https://www.ebi.ac.uk/chembl/target/inspect/CHEMBL225)	Serotonin 2c (5-HT2c) receptor	Show NN
8	CHEMBL240 (https://www.ebi.ac.uk/chembl/target/inspect/CHEMBL240)	HERG	Show NN
9	CHEMBL234 (https://www.ebi.ac.uk/chembl/target/inspect/CHEMBL234)	Dopamine D3 receptor	Show NN
10	CHEMBL3155 (https://www.ebi.ac.uk/chembl/target/inspect/CHEMBL3155)	Serotonin 7 (5-HT7) receptor	Show NN
11	CHEMBL3138 (https://www.ebi.ac.uk/chembl/target/inspect/CHEMBL3138)	Dopamine D3 receptor	Show NN
12	CHEMBL231 (https://www.ebi.ac.uk/chembl/target/inspect/CHEMBL231)	Histamine H1 receptor	Show NN
13	CHEMBL265 (https://www.ebi.ac.uk/chembl/target/inspect/CHEMBL265)	Dopamine D1 receptor	Show NN
14	CHEMBL219 (https://www.ebi.ac.uk/chembl/target/inspect/CHEMBL219)	Dopamine D4 receptor	Show NN
15	CHEMBL2056 (https://www.ebi.ac.uk/chembl/target/inspect/CHEMBL2056)	Dopamine D1 receptor	Show NN
16	CHEMBL324 (https://www.ebi.ac.uk/chembl/target/inspect/CHEMBL324)	Serotonin 2c (5-HT2c) receptor	Show NN
17	CHEMBL287 (https://www.ebi.ac.uk/chembl/target/inspect/CHEMBL287)	Sigma opioid receptor	Show NN
18	CHEMBL214 (https://www.ebi.ac.uk/chembl/target/inspect/CHEMBL214)	Serotonin 1a (5-HT1a) receptor	Show NN

19	CHEMBL319 (https://www.ebi.ac.uk/chembl/target/inspect/CHEMBL319)	Alpha-1a adrenergic receptor	Show NN
20	CHEMBL220 (https://www.ebi.ac.uk/chembl/target/inspect/CHEMBL220)	Acetylcholinesterase	Show NN
