# Supplementary material for: Accelerating Drug Discovery by Early Protein Drug Target Prediction Based on a Multi-Fingerprint Similarity Search
Source: Molecules. 2019 Jun 14;24(12):2233. doi: 10.3390/molecules24122233 (PMC6631269; doi:10.3390/molecules24122233)
Supplement: Supplementary file 1 [file molecules-24-02233-s001.zip › File_S2.zip!/File_S2/Target_Prediction_compound#14.pdf]

MuSSel Prediction IC_{50}:

```
1 rank
Neuraminidase : Influenza A virus
      score: 11.797 on CHEMBL1667684 based on 13 fingerprints
Fingerprint type         Ligand  Tanimoto Similarity  activity Valid fg
       FeatMFP1  CHEMBL3342471            0.923077       7.1         *
           MFP1  CHEMBL3342471            0.918919       7.1         *
         RDKit7  CHEMBL3342471            0.929990       7.1         *
        Pattern  CHEMBL3342478            0.965753       1.9         *
        AP_bits  CHEMBL3342478            0.792363       1.9         *
        TT_bits  CHEMBL3342471            0.830189       7.1         *
            FP2  CHEMBL3342471            0.891892       7.1         *
  hybridization  CHEMBL3342485            0.966469       2.1         *
   substructure  CHEMBL3342471            0.920000       7.1         *
          graph  CHEMBL3342485            0.921053       2.1         *
        pubchem  CHEMBL3342485            0.892405       2.1         *
      cdk_maccs  CHEMBL3342478            0.913793       1.9         *
   klekota_roth  CHEMBL3342478            0.930693       1.9         *
      *** ic50 ACTIVITY *** value prediction
      based on 13 locally validated fgps ---> 7.924
["TT_bits", "substructure", "cdk_maccs", "Pattern", "FP2", "MFP1",
"klekota_roth", "pubchem", "graph", "AP_bits", "FeatMFP1", "RDKit7",
"hybridization"]

2 rank
Neuraminidase : Influenza A virus (strain A/USSR/90/1977 H1N1)
      score: 11.797 on CHEMBL3559643 based on 13 fingerprints
Fingerprint type         Ligand  Tanimoto Similarity  activity Valid fg
       FeatMFP1  CHEMBL3342471            0.923077      19.0         *
           MFP1  CHEMBL3342471            0.918919      19.0         *
         RDKit7  CHEMBL3342471            0.929990      19.0         *
        Pattern  CHEMBL3342478            0.965753       3.8         *
        AP_bits  CHEMBL3342478            0.792363       3.8         *
        TT_bits  CHEMBL3342471            0.830189      19.0         *
            FP2  CHEMBL3342471            0.891892      19.0         *
  hybridization  CHEMBL3342485            0.966469       4.3         *
   substructure  CHEMBL3342471            0.920000      19.0         *
          graph  CHEMBL3342485            0.921053       4.3         *
        pubchem  CHEMBL3342485            0.892405       4.3         *
      cdk_maccs  CHEMBL3342478            0.913793       3.8         *
   klekota_roth  CHEMBL3342478            0.930693       3.8         *
      *** ic50 ACTIVITY *** value prediction
      based on 13 locally validated fgps ---> 18.838
["TT_bits", "substructure", "cdk_maccs", "Pattern", "FP2", "MFP1",
"klekota_roth", "pubchem", "graph", "AP_bits", "FeatMFP1", "RDKit7",
"hybridization"]

3 rank
Neuraminidase : Influenza A virus (A/Puerto Rico/8/1934(H1N1))
      score: 7.557 on CHEMBL2051 based on 10 fingerprints
Fingerprint type        Ligand  Tanimoto Similarity  activity Valid fg
       FeatMFP1  CHEMBL310217            0.814815     620.0         *
           MFP1   CHEMBL79499            0.714286      12.0         *
         RDKit7     CHEMBL674            0.774295       1.0         *
        Pattern   CHEMBL81083            0.912644      90.0         *
        AP_bits   CHEMBL81083            0.545652      90.0         *
        TT_bits   CHEMBL79499            0.573770      12.0         *
            FP2   CHEMBL79499            0.725000      12.0         *
  hybridization   CHEMBL81717            0.760748       0.5         *
   substructure  CHEMBL310217            0.846154     620.0
          graph   CHEMBL53768            0.672043    1500.0
```

```
        pubchem   CHEMBL81083              0.846667      90.0        *
      cdk_maccs   CHEMBL53336              0.771930    2300.0
   klekota_roth     CHEMBL674              0.888889       1.0        *
      *** ic50 ACTIVITY *** value prediction
      based on 10 locally validated fgps ---> 21.359
["TT_bits", "Pattern", "FP2", "MFP1", "klekota_roth", "pubchem",
"AP_bits", "FeatMFP1", "RDKit7", "hybridization"]

4 rank
Neuraminidase : Influenza B virus (strain B/Lee/1940)
      score: 6.560 on CHEMBL3377 based on 9 fingerprints
Fingerprint type        Ligand  Tanimoto Similarity  activity Valid fg
       FeatMFP1   CHEMBL79499              0.814815      35.0        *
           MFP1   CHEMBL79499              0.714286      35.0        *
         RDKit7     CHEMBL674              0.774295       3.0        *
        Pattern   CHEMBL98906              0.822811       1.6
        AP_bits  CHEMBL352318              0.532946       0.3        *
        TT_bits   CHEMBL79499              0.573770      35.0        *
            FP2   CHEMBL79499              0.725000      35.0        *
  hybridization     CHEMBL674              0.698189       3.0        *
   substructure   CHEMBL79499              0.846154      35.0
          graph  CHEMBL311273              0.656085   36000.0
        pubchem   CHEMBL79499              0.837838      35.0        *
      cdk_maccs     CHEMBL674              0.771930       3.0
   klekota_roth     CHEMBL674              0.888889       3.0        *
      *** ic50 ACTIVITY *** value prediction
      based on 9 locally validated fgps ---> 40.557
["TT_bits", "AP_bits", "MFP1", "klekota_roth", "pubchem", "FP2",
"FeatMFP1", "RDKit7", "hybridization"]

5 rank
Neuraminidase : Influenza A virus (strain A/Wilson-Smith/1933 H1N1)
(Influenza A virus(strain A/WS/1933 H1N1))
      score: 5.582 on CHEMBL1287610 based on 8 fingerprints
Fingerprint type         Ligand  Tanimoto Similarity  activity Valid fg
       FeatMFP1      CHEMBL674              0.730769       2.6        *
           MFP1      CHEMBL674              0.675000       2.6        *
         RDKit7      CHEMBL674              0.774295       2.6        *
        Pattern  CHEMBL3357205              0.817181    6946.0
        AP_bits  CHEMBL3263942              0.498099      24.6        *
        TT_bits  CHEMBL3357205              0.593750    6946.0        *
            FP2      CHEMBL674              0.722973       2.6        *
  hybridization      CHEMBL674              0.698189       2.6        *
   substructure      CHEMBL674              0.807692       2.6
          graph      CHEMBL674              0.650273       2.6
        pubchem  CHEMBL3357202              0.776316    1010.0
      cdk_maccs      CHEMBL674              0.771930       2.6
   klekota_roth      CHEMBL674              0.888889       2.6        *
      *** ic50 ACTIVITY *** value prediction
      based on 8 locally validated fgps ---> 1425.604
["TT_bits", "AP_bits", "MFP1", "klekota_roth", "FP2", "FeatMFP1",
"RDKit7", "hybridization"]

6 rank
Neuraminidase : Influenza A virus
      score: 5.505 on CHEMBL6135 based on 8 fingerprints
Fingerprint type         Ligand  Tanimoto Similarity  activity Valid fg
       FeatMFP1      CHEMBL674              0.730769       0.4        *
           MFP1      CHEMBL674              0.675000       0.4        *
         RDKit7      CHEMBL674              0.774295       0.4        *
```

Pattern	CHEMBL3263942	0.772277	3.2	
AP_bits	CHEMBL3263939	0.515267	1.7	*
TT_bits	CHEMBL674	0.500000	0.4	*
FP2	CHEMBL674	0.722973	0.4	*
hybridization	CHEMBL674	0.698189	0.4	*
substructure	CHEMBL674	0.807692	0.4	
graph	CHEMBL674	0.650273	0.4	
pubchem	CHEMBL3263938	0.673203	1.8	
cdk_maccs	CHEMBL674	0.771930	0.4	
klekota_roth	CHEMBL674	0.888889	0.4	*

*** ic50 ACTIVITY *** value prediction
based on 8 locally validated fgps ---> 0.649
["TT_bits", "AP_bits", "MFP1", "klekota_roth", "FP2", "FeatMFP1", "RDKit7", "hybridization"]

SwissTargetPrediction report:

Reference:
Gfeller D., Michielin O. & Zoete V.
Shaping the interaction landscape of bioactive molecules, *Bioinformatics* (2013) 29:3073-3079.

Query Molecule

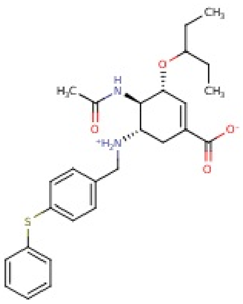

Frequency of Target Class

Target	Uniprot ID	Gene code	ChEMBL ID	Probability	# sim. cmpds (3D / 2D)	Target Class
5-hydroxytryptamine receptor 2A	P28223	HTR2A	CHEMBL224		16 / 13	Membrane receptor
5-hydroxytryptamine receptor 2C *(by homology)*	P28335	HTR2C	CHEMBL225		4 / 13	Membrane receptor
5-hydroxytryptamine receptor 2B *(by homology)*	P41595	HTR2B	CHEMBL1833		4 / 13	Membrane receptor
Activation peptide fragment 1	P00734	F2	CHEMBL204		49 / 4	Serine Protease
Muscleblind-like protein 1	Q9NR56	MBNL1	CHEMBL1293317		24 / 6	Unclassified
Muscleblind-like protein 2 *(by homology)*	Q5VZF2	MBNL2			24 / 6	Unclassified
Muscleblind-like protein 3 *(by homology)*	Q9NUK0	MBNL3			24 / 6	Unclassified
Factor X light chain	P00742	F10	CHEMBL244		45 / 3	Serine Protease
Coagulation factor VII	P08709	F7	CHEMBL3991		45 / 3	Serine Protease
Coagulation factor IXa heavy chain *(by homology)*	P00740	F9	CHEMBL2016		45 / 3	Serine Protease
Microtubule-associated protein tau	P10636	MAPT	CHEMBL1293224		52 / 9	Unclassified
Thromboxane A2 receptor	P21731	TBXA2R	CHEMBL2069		139 / 6	Membrane receptor
Alpha-trypsin chain 1	P07477	PRSS1	CHEMBL209		10 / 2	Serine Protease
Trypsin-2 *(by homology)*	P07478	PRSS2	CHEMBL3159		10 / 2	Serine Protease
Trypsin-3 *(by homology)*	P35030	PRSS3	CHEMBL4551		10 / 2	Serine Protease

Polypharmacology Browser 2 Prediction:

Targets predicted using NN(ECfp4) + NB(ECfp4). Save Table

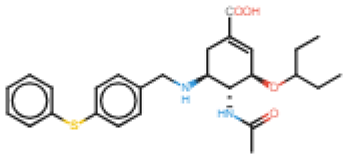

Query molecule

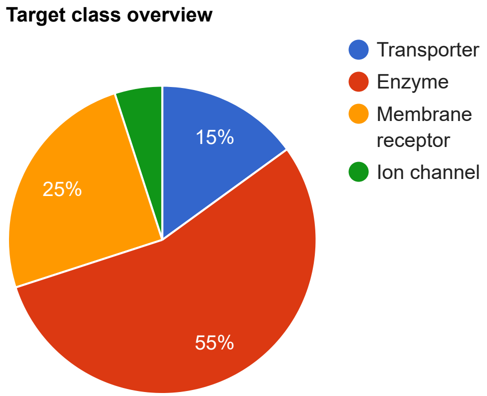

Rank	ChEMBL ID	Common name	Nearest neighbours
1	CHEMBL332 (https://www.ebi.ac.uk/chembl/target/inspect/CHEMBL332)	Matrix metalloproteinase-1	Show NN
2	CHEMBL333 (https://www.ebi.ac.uk/chembl/target/inspect/CHEMBL333)	Matrix metalloproteinase-2	Show NN
3	CHEMBL338 (https://www.ebi.ac.uk/chembl/target/inspect/CHEMBL338)	Dopamine transporter	Show NN
4	CHEMBL313 (https://www.ebi.ac.uk/chembl/target/inspect/CHEMBL313)	Serotonin transporter	Show NN
5	CHEMBL280 (https://www.ebi.ac.uk/chembl/target/inspect/CHEMBL280)	Matrix metalloproteinase 13	Show NN
6	CHEMBL4822 (https://www.ebi.ac.uk/chembl/target/inspect/CHEMBL4822)	Beta-secretase 1	Show NN
7	CHEMBL2625 (https://www.ebi.ac.uk/chembl/target/inspect/CHEMBL2625)	Angiotensin-converting enzyme	Show NN
8	CHEMBL283 (https://www.ebi.ac.uk/chembl/target/inspect/CHEMBL283)	Matrix metalloproteinase 3	Show NN
9	CHEMBL321 (https://www.ebi.ac.uk/chembl/target/inspect/CHEMBL321)	Matrix metalloproteinase 9	Show NN
10	CHEMBL304 (https://www.ebi.ac.uk/chembl/target/inspect/CHEMBL304)	Norepinephrine transporter	Show NN
11	CHEMBL233 (https://www.ebi.ac.uk/chembl/target/inspect/CHEMBL233)	Mu opioid receptor	Show NN
12	CHEMBL236 (https://www.ebi.ac.uk/chembl/target/inspect/CHEMBL236)	Delta opioid receptor	Show NN
13	CHEMBL1944 (https://www.ebi.ac.uk/chembl/target/inspect/CHEMBL1944)	Neprilysin	Show NN
14	CHEMBL240 (https://www.ebi.ac.uk/chembl/target/inspect/CHEMBL240)	HERG	Show NN
15	CHEMBL2581 (https://www.ebi.ac.uk/chembl/target/inspect/CHEMBL2581)	Cathepsin D	Show NN
16	CHEMBL3369 (https://www.ebi.ac.uk/chembl/target/inspect/CHEMBL3369)	Neprilysin	Show NN
17	CHEMBL1945 (https://www.ebi.ac.uk/chembl/target/inspect/CHEMBL1945)	Melatonin receptor 1A	Show NN
18	CHEMBL217 (https://www.ebi.ac.uk/chembl/target/inspect/CHEMBL217)	Dopamine D2 receptor	Show NN

19	CHEMBL4588 (https://www.ebi.ac.uk/chembl/target/inspect/CHEMBL4588)	Matrix metalloproteinase 8	Show NN
20	CHEMBL249 (https://www.ebi.ac.uk/chembl/target/inspect/CHEMBL249)	Neurokinin 1 receptor	Show NN
