# Supplementary material for: Accelerating Drug Discovery by Early Protein Drug Target Prediction Based on a Multi-Fingerprint Similarity Search
Source: Molecules. 2019 Jun 14;24(12):2233. doi: 10.3390/molecules24122233 (PMC6631269; doi:10.3390/molecules24122233)
Supplement: Supplementary file 1 [file molecules-24-02233-s001.zip › File_S2.zip!/File_S2/Target_Prediction_compound#15.pdf]

MuSSel Prediction k_i:

1 rank

Mitogen-activated protein kinase kinase kinase 12 : Homo sapiens

score: 4.006 on CHEMBL1908389 based on 5 fingerprints

Fingerprint type	Ligand	Tanimoto Similarity	activity	Valid fg
FeatMFP1	CHEMBL3730276	0.703704	4.63	
MFP1	CHEMBL3715814	0.552632	12.00	
RDKit7	CHEMBL3728787	0.552040	130.00	
Pattern	CHEMBL3729398	0.824089	210.00	
AP_bits	CHEMBL3728787	0.596923	130.00	*
TT_bits	CHEMBL3728787	0.414634	130.00	
FP2	CHEMBL3729749	0.614213	137.00	
hybridization	CHEMBL3728787	0.674731	130.00	
substructure	CHEMBL3727745	1.000000	650.00	*
graph	CHEMBL3728787	0.800000	130.00	
pubchem	CHEMBL3730820	0.874286	220.00	*
cdk_maccs	CHEMBL3728787	0.851852	130.00	*
klekota_roth	CHEMBL3730148	0.682540	1360.00	*

*** ki ACTIVITY *** value prediction

based on 5 locally validated fgps ---> 365.364

["AP_bits", "cdk_maccs", "klekota_roth", "substructure", "pubchem"]

MuSSel Prediction IC_{50}:

```
1 rank
PI3-kinase p110-alpha subunit : Homo sapiens
     score: 8.198 on CHEMBL4005 based on 11 fingerprints
Fingerprint type          Ligand  Tanimoto Similarity  activity Valid fg
       FeatMFP1  CHEMBL2017974           0.720000      30.0        *
           MFP1  CHEMBL2017974           0.472222      30.0
         RDKit7  CHEMBL1258517           0.667313       8.0
        Pattern  CHEMBL1091596           0.866667     253.0        *
        AP_bits  CHEMBL2017974           0.533898      30.0        *
        TT_bits  CHEMBL1092394           0.493671     226.0        *
            FP2  CHEMBL1258517           0.646707       8.0        *
  hybridization  CHEMBL1258517           0.736196       8.0        *
   substructure  CHEMBL1672330           0.923077      13.0        *
          graph  CHEMBL1258517           0.789474       8.0        *
        pubchem  CHEMBL2017974           0.877907      30.0        *
      cdk_maccs  CHEMBL3736010           0.910714     220.0        *
   klekota_roth  CHEMBL3601897           0.700000    2200.0        *
     *** ic50 ACTIVITY *** value prediction
     based on 11 locally validated fgps ---> 253.529
["TT_bits", "substructure", "Pattern", "AP_bits", "klekota_roth",
"pubchem", "graph", "FP2", "FeatMFP1", "cdk_maccs", "hybridization"]

2 rank
Serine/threonine-protein kinase mTOR : Homo sapiens
     score: 8.103 on CHEMBL2842 based on 11 fingerprints
Fingerprint type          Ligand  Tanimoto Similarity  activity Valid fg
       FeatMFP1  CHEMBL2017974           0.720000   4600.00        *
           MFP1  CHEMBL2017974           0.472222   4600.00
         RDKit7  CHEMBL1258517           0.667313      0.42
        Pattern  CHEMBL1091596           0.866667      0.18        *
        AP_bits  CHEMBL2017974           0.533898   4600.00        *
        TT_bits  CHEMBL1092394           0.493671      0.89        *
            FP2  CHEMBL1258517           0.646707      0.42        *
  hybridization  CHEMBL1258517           0.736196      0.42        *
   substructure  CHEMBL1684984           0.857143   1600.00        *
          graph  CHEMBL1258517           0.789474      0.42        *
        pubchem  CHEMBL2017974           0.877907   4600.00        *
      cdk_maccs   CHEMBL586702           0.890909    350.00        *
   klekota_roth  CHEMBL1092394           0.690141      0.89        *
     *** ic50 ACTIVITY *** value prediction
     based on 11 locally validated fgps ---> 1162.658
["TT_bits", "substructure", "Pattern", "AP_bits", "klekota_roth",
"pubchem", "graph", "FP2", "FeatMFP1", "cdk_maccs", "hybridization"]

3 rank
PI3-kinase p110-beta subunit : Homo sapiens
     score: 6.765 on CHEMBL3145 based on 9 fingerprints
Fingerprint type          Ligand  Tanimoto Similarity  activity Valid fg
       FeatMFP1  CHEMBL2017974           0.720000     166.0        *
           MFP1  CHEMBL2017974           0.472222     166.0
         RDKit7  CHEMBL1258517           0.667313      24.0
        Pattern  CHEMBL1258517           0.820896      24.0
        AP_bits  CHEMBL2017974           0.533898     166.0        *
        TT_bits  CHEMBL1258517           0.329670      24.0
            FP2  CHEMBL1258517           0.646707      24.0        *
  hybridization  CHEMBL1258517           0.736196      24.0        *
   substructure  CHEMBL1684984           0.857143     120.0        *
          graph  CHEMBL1258517           0.789474      24.0        *
        pubchem  CHEMBL2017974           0.877907     166.0        *
      cdk_maccs  CHEMBL1911005           0.927273      41.0        *
```

```
   klekota_roth  CHEMBL1258517             0.676056      24.0        *
      *** ic50 ACTIVITY *** value prediction
      based on 9 locally validated fgps ---> 73.466
["substructure", "AP_bits", "FP2", "klekota_roth", "pubchem", "graph",
"FeatMFP1", "cdk_maccs", "hybridization"]

4 rank
PI3-kinase p110-gamma subunit : Homo sapiens
      score: 6.738 on CHEMBL3267 based on 9 fingerprints
Fingerprint type         Ligand  Tanimoto Similarity  activity Valid fg
       FeatMFP1  CHEMBL2017974             0.720000     262.0        *
           MFP1  CHEMBL2017974             0.472222     262.0
         RDKit7  CHEMBL1258517             0.667313      74.0
        Pattern  CHEMBL1258744             0.825806      33.0
        AP_bits  CHEMBL2017974             0.533898     262.0        *
        TT_bits  CHEMBL1258634             0.351648      99.0
            FP2  CHEMBL1258517             0.646707      74.0        *
  hybridization  CHEMBL1258517             0.736196      74.0        *
   substructure  CHEMBL1684984             0.857143      36.0        *
          graph  CHEMBL1258517             0.789474      74.0        *
        pubchem  CHEMBL2017974             0.877907     262.0        *
      cdk_maccs   CHEMBL586702             0.890909       5.0        *
   klekota_roth  CHEMBL1258516             0.685714     150.0        *
      *** ic50 ACTIVITY *** value prediction
      based on 9 locally validated fgps ---> 143.917
["substructure", "AP_bits", "FP2", "klekota_roth", "pubchem", "graph",
"FeatMFP1", "cdk_maccs", "hybridization"]

5 rank
PI3-kinase p110-delta subunit : Homo sapiens
      score: 6.728 on CHEMBL3130 based on 9 fingerprints
Fingerprint type         Ligand  Tanimoto Similarity  activity Valid fg
       FeatMFP1  CHEMBL2017974             0.720000     116.0        *
           MFP1  CHEMBL2017974             0.472222     116.0
         RDKit7  CHEMBL1258517             0.667313      77.0
        Pattern  CHEMBL1258517             0.820896      77.0
        AP_bits  CHEMBL2017974             0.533898     116.0        *
        TT_bits  CHEMBL1258517             0.329670      77.0
            FP2  CHEMBL1258517             0.646707      77.0        *
  hybridization  CHEMBL1258517             0.736196      77.0        *
   substructure  CHEMBL1684984             0.857143     500.0        *
          graph  CHEMBL1258517             0.789474      77.0        *
        pubchem  CHEMBL2017974             0.877907     116.0        *
      cdk_maccs   CHEMBL586702             0.890909       3.0        *
   klekota_roth  CHEMBL1258517             0.676056      77.0        *
      *** ic50 ACTIVITY *** value prediction
      based on 9 locally validated fgps ---> 105.529
["substructure", "AP_bits", "FP2", "klekota_roth", "pubchem", "graph",
"FeatMFP1", "cdk_maccs", "hybridization"]
```

SwissTargetPrediction report:

Reference:
Gfeller D., Michielin O. & Zoete V.
Shaping the interaction landscape of bioactive molecules, *Bioinformatics* (2013) 29:3073-3079.

Query Molecule

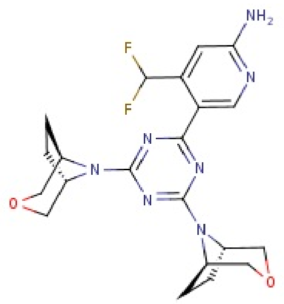

Frequency of Target Class

Target	Uniprot ID	Gene code	ChEMBL ID	Probability	# sim. cmpds (3D / 2D)	Target Class
Phosphatidylinositol 4,5-bisphosphate 3-kinase catalytic subunit delta isoform	O00329	PIK3CD	CHEMBL3130		95 / 129	Enzyme
Phosphatidylinositol 4,5-bisphosphate 3-kinase catalytic subunit alpha isoform	P42336	PIK3CA	CHEMBL4005		95 / 129	Enzyme
Phosphatidylinositol 4,5-bisphosphate 3-kinase catalytic subunit beta isoform	P42338	PIK3CB	CHEMBL3145		95 / 129	Enzyme
Serine/threonine-protein kinase mTOR	P42345	MTOR	CHEMBL2842		28 / 111	Enzyme
Phosphatidylinositol 4,5-bisphosphate 3-kinase catalytic subunit gamma isoform	P48736	PIK3CG	CHEMBL3267		29 / 44	Enzyme
Cyclin-dependent kinase 1	P06493	CDK1	CHEMBL308		33 / 5	Ser_Thr Kinase
Cyclin-dependent kinase 4 *(by homology)*	P11802	CDK4	CHEMBL331		33 / 5	Ser_Thr Kinase
Cyclin-dependent kinase 2	P24941	CDK2	CHEMBL301		33 / 5	Ser_Thr Kinase
Cyclin-dependent kinase 3 *(by homology)*	Q00526	CDK3	CHEMBL4442		33 / 5	Ser_Thr Kinase
Cyclin-dependent kinase 6 *(by homology)*	Q00534	CDK6	CHEMBL2508		33 / 5	Ser_Thr Kinase
Aurora kinase A	O14965	AURKA	CHEMBL4722		40 / 5	Ser_Thr Kinase
Aurora kinase B	Q96GD4	AURKB	CHEMBL2185		40 / 5	Ser_Thr Kinase
Aurora kinase C *(by homology)*	Q9UQB9	AURKC	CHEMBL3935		40 / 5	Ser_Thr Kinase
Vascular endothelial growth factor receptor 1 *(by homology)*	P17948	FLT1	CHEMBL1868		48 / 1	Tyr Kinase
Vascular endothelial growth factor receptor 3 *(by homology)*	P35916	FLT4	CHEMBL1955		48 / 1	Tyr Kinase

Polypharmacology Browser 2 Prediction:

Targets predicted using NN(ECfp4) + NB(ECfp4). Save Table

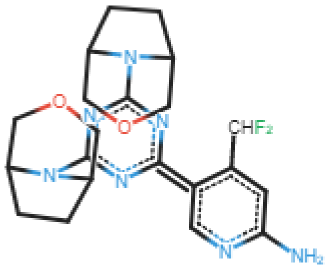

Query molecule

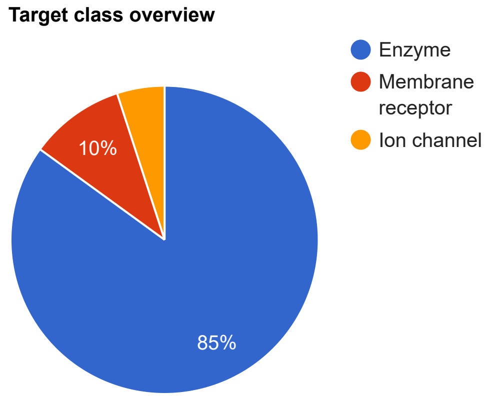

Rank	ChEMBL ID	Common name	Nearest neighbours
1	CHEMBL4005 (https://www.ebi.ac.uk/chembl/target/inspect/CHEMBL4005)	PI3-kinase p110-alpha subunit	Show NN
2	CHEMBL2842 (https://www.ebi.ac.uk/chembl/target/inspect/CHEMBL2842)	Serine/threonine-protein kinase mTOR	Show NN
3	CHEMBL3267 (https://www.ebi.ac.uk/chembl/target/inspect/CHEMBL3267)	PI3-kinase p110-gamma subunit	Show NN
4	CHEMBL3130 (https://www.ebi.ac.uk/chembl/target/inspect/CHEMBL3130)	PI3-kinase p110-delta subunit	Show NN
5	CHEMBL3145 (https://www.ebi.ac.uk/chembl/target/inspect/CHEMBL3145)	PI3-kinase p110-beta subunit	Show NN
6	CHEMBL2148 (https://www.ebi.ac.uk/chembl/target/inspect/CHEMBL2148)	Tyrosine-protein kinase JAK3	Show NN
7	CHEMBL1862 (https://www.ebi.ac.uk/chembl/target/inspect/CHEMBL1862)	Tyrosine-protein kinase ABL	Show NN
8	CHEMBL2971 (https://www.ebi.ac.uk/chembl/target/inspect/CHEMBL2971)	Tyrosine-protein kinase JAK2	Show NN
9	CHEMBL203 (https://www.ebi.ac.uk/chembl/target/inspect/CHEMBL203)	Epidermal growth factor receptor erbB1	Show NN
10	CHEMBL240 (https://www.ebi.ac.uk/chembl/target/inspect/CHEMBL240)	HERG	Show NN
11	CHEMBL1974 (https://www.ebi.ac.uk/chembl/target/inspect/CHEMBL1974)	Tyrosine-protein kinase receptor FLT3	Show NN
12	CHEMBL2276 (https://www.ebi.ac.uk/chembl/target/inspect/CHEMBL2276)	c-Jun N-terminal kinase 1	Show NN
13	CHEMBL253 (https://www.ebi.ac.uk/chembl/target/inspect/CHEMBL253)	Cannabinoid CB2 receptor	Show NN
14	CHEMBL4179 (https://www.ebi.ac.uk/chembl/target/inspect/CHEMBL4179)	c-Jun N-terminal kinase 2	Show NN
15	CHEMBL2185 (https://www.ebi.ac.uk/chembl/target/inspect/CHEMBL2185)	Serine/threonine-protein kinase Aurora-B	Show NN
16	CHEMBL4282 (https://www.ebi.ac.uk/chembl/target/inspect/CHEMBL4282)	Serine/threonine-protein kinase AKT	Show NN
17	CHEMBL218 (https://www.ebi.ac.uk/chembl/target/inspect/CHEMBL218)	Cannabinoid CB1 receptor	Show NN
18	CHEMBL2147 (https://www.ebi.ac.uk/chembl/target/inspect/CHEMBL2147)	Serine/threonine-protein kinase PIM1	Show NN

19	CHEMBL4040 (https://www.ebi.ac.uk/chembl/target/inspect/CHEMBL4040)	MAP kinase ERK2	Show NN
20	CHEMBL1075104 (https://www.ebi.ac.uk/chembl/target/inspect/CHEMBL1075104)	Leucine-rich repeat serine/threonine-protein kinase 2	Show NN
