# Supplementary material for: Accelerating Drug Discovery by Early Protein Drug Target Prediction Based on a Multi-Fingerprint Similarity Search
Source: Molecules. 2019 Jun 14;24(12):2233. doi: 10.3390/molecules24122233 (PMC6631269; doi:10.3390/molecules24122233)
Supplement: Supplementary file 1 [file molecules-24-02233-s001.zip › File_S2.zip!/File_S2/Target_Prediction_compound#16.pdf]

MuSSel Prediction k_i:

```
1 rank
Thrombin : Homo sapiens
      score: 7.800 on CHEMBL204 based on 10 fingerprints
Fingerprint type        Ligand  Tanimoto Similarity  activity Valid fg
       FeatMFP1  CHEMBL179210            0.766667      1.10        *
           MFP1  CHEMBL164138            0.666667  12300.00        *
         RDKit7  CHEMBL366882            0.824427      3.10        *
        Pattern  CHEMBL366882            0.921348      3.10        *
        AP_bits  CHEMBL366882            0.620787      3.10        *
        TT_bits  CHEMBL366882            0.594059      3.10        *
            FP2  CHEMBL366882            0.826667      3.10        *
  hybridization  CHEMBL403735            0.773616      1.10        *
   substructure  CHEMBL164138            0.913043  12300.00        *
          graph  CHEMBL319615            0.762658   5300.00
        pubchem  CHEMBL366882            0.892523      3.10        *
      cdk_maccs  CHEMBL322307            0.813333     50.00
   klekota_roth  CHEMBL282830            0.596639      0.65
      *** ki ACTIVITY *** value prediction
      based on 10 locally validated fgps ---> 1850.557
["TT_bits", "substructure", "Pattern", "FP2", "MFP1", "pubchem",
"AP_bits", "FeatMFP1", "RDKit7", "hybridization"]

2 rank
Trypsin I : Bos taurus
      score: 7.782 on CHEMBL3769 based on 10 fingerprints
Fingerprint type        Ligand  Tanimoto Similarity  activity Valid fg
       FeatMFP1  CHEMBL179210            0.766667      22.0        *
           MFP1  CHEMBL360684            0.666667      30.0        *
         RDKit7  CHEMBL366882            0.824427       2.0        *
        Pattern  CHEMBL366882            0.921348       2.0        *
        AP_bits  CHEMBL366882            0.620787       2.0        *
        TT_bits  CHEMBL366882            0.594059       2.0        *
            FP2  CHEMBL366882            0.826667       2.0        *
  hybridization  CHEMBL366882            0.756058       2.0        *
   substructure  CHEMBL360684            0.913043      30.0        *
          graph  CHEMBL257898            0.760714      16.0
        pubchem  CHEMBL366882            0.892523       2.0        *
      cdk_maccs  CHEMBL368894            0.805556      22.0
   klekota_roth  CHEMBL180219            0.636364     250.0
      *** ki ACTIVITY *** value prediction
      based on 10 locally validated fgps ---> 75.164
["TT_bits", "substructure", "Pattern", "FP2", "MFP1", "pubchem",
"AP_bits", "FeatMFP1", "RDKit7", "hybridization"]

3 rank
Serine protease hepsin : Homo sapiens
      score: 3.655 on CHEMBL2079849 based on 5 fingerprints
Fingerprint type         Ligand  Tanimoto Similarity  activity Valid fg
       FeatMFP1  CHEMBL3746517            0.700000      2.91
           MFP1  CHEMBL3746517            0.652174      2.91        *
         RDKit7  CHEMBL3746517            0.727202      2.91
        Pattern  CHEMBL3402437            0.709635   3500.00
        AP_bits  CHEMBL3402438            0.538776   2600.00        *
        TT_bits  CHEMBL3746517            0.537500      2.91
            FP2  CHEMBL3746517            0.740385      2.91        *
  hybridization  CHEMBL3746517            0.807207      2.91        *
   substructure  CHEMBL3747468            0.916667      2.88        *
          graph  CHEMBL3746517            0.773438      2.91
        pubchem  CHEMBL3746517            0.805000      2.91
      cdk_maccs  CHEMBL3746517            0.746479      2.91
```

```
   klekota_roth  CHEMBL3746517             0.605042      2.91
      *** ki ACTIVITY *** value prediction
      based on 5 locally validated fgps ---> 522.183
["AP_bits", "hybridization", "MFP1", "substructure", "FP2"]

4 rank
Trypsin I : Homo sapiens
      score: 3.610 on CHEMBL209 based on 5 fingerprints
Fingerprint type        Ligand  Tanimoto Similarity  activity Valid fg
       FeatMFP1  CHEMBL164138             0.724138      30.0
           MFP1  CHEMBL164138             0.666667      30.0        *
         RDKit7  CHEMBL341148             0.716385     160.0
        Pattern  CHEMBL332716             0.808140    4000.0
        AP_bits  CHEMBL311198             0.568460      13.5        *
        TT_bits  CHEMBL164138             0.481481      30.0
            FP2  CHEMBL341148             0.710407     160.0        *
  hybridization  CHEMBL164138             0.751351      30.0        *
   substructure  CHEMBL164138             0.913043      30.0        *
          graph  CHEMBL339703             0.778210      53.0
        pubchem  CHEMBL341148             0.831683     160.0
      cdk_maccs  CHEMBL340098             0.808824     260.0
   klekota_roth  CHEMBL164138             0.572650      30.0
      *** ki ACTIVITY *** value prediction
      based on 5 locally validated fgps ---> 52.604
["AP_bits", "hybridization", "MFP1", "substructure", "FP2"]

5 rank
Matriptase : Homo sapiens
      score: 3.560 on CHEMBL3018 based on 5 fingerprints
Fingerprint type         Ligand  Tanimoto Similarity  activity Valid fg
       FeatMFP1  CHEMBL2089124             0.700000       1.4
           MFP1  CHEMBL2089124             0.666667       1.4        *
         RDKit7  CHEMBL2089124             0.699449       1.4
        Pattern  CHEMBL2089121             0.765926    6124.0
        AP_bits  CHEMBL3402438             0.538776    6700.0        *
        TT_bits  CHEMBL2089124             0.487179       1.4
            FP2  CHEMBL2089124             0.725962       1.4        *
  hybridization  CHEMBL2089124             0.777982       1.4        *
   substructure  CHEMBL2089124             0.740741       1.4
          graph  CHEMBL2089121             0.750929    6124.0
        pubchem  CHEMBL2089124             0.785000       1.4
      cdk_maccs  CHEMBL2089121             0.850746    6124.0        *
   klekota_roth  CHEMBL2089124             0.620370       1.4
      *** ki ACTIVITY *** value prediction
      based on 5 locally validated fgps ---> 1947.147
["AP_bits", "hybridization", "MFP1", "cdk_maccs", "FP2"]

6 rank
Tryptase beta-1 : Homo sapiens
      score: 3.021 on CHEMBL2617 based on 4 fingerprints
Fingerprint type        Ligand  Tanimoto Similarity  activity Valid fg
       FeatMFP1  CHEMBL340547             0.724138      88.0
           MFP1  CHEMBL340547             0.666667      88.0        *
         RDKit7  CHEMBL341148             0.716385     250.0
        Pattern  CHEMBL311936             0.742029    6900.0
        AP_bits  CHEMBL211638             0.502558      11.0
        TT_bits  CHEMBL340547             0.481481      88.0
            FP2  CHEMBL341148             0.710407     250.0        *
  hybridization  CHEMBL421643             0.730634      41.0        *
   substructure  CHEMBL340547             0.913043      88.0        *
```

```
         graph  CHEMBL339703             0.778210      20.0
       pubchem  CHEMBL341148             0.831683     250.0
     cdk_maccs  CHEMBL340098             0.808824     470.0
  klekota_roth  CHEMBL340547             0.572650      88.0
     *** ki ACTIVITY *** value prediction
     based on 4 locally validated fgps ---> 156.257
["hybridization", "MFP1", "substructure", "FP2"]
```

MuSSel Prediction IC$_{50}$:

1 rank
Metacaspase : Trypanosoma brucei
score: 9.500 on CHEMBL1075193 based on 12 fingerprints

Fingerprint type	Ligand	Tanimoto Similarity	activity	Valid fg
FeatMFP1	CHEMBL1077318	0.793103	600.0	*
MFP1	CHEMBL1077318	0.761905	600.0	*
RDKit7	CHEMBL1082043	0.770912	1100.0	*
Pattern	CHEMBL1082042	0.750000	1400.0	
AP_bits	CHEMBL1082042	0.655864	1400.0	*
TT_bits	CHEMBL1082042	0.558140	1400.0	*
FP2	CHEMBL1082043	0.792627	1100.0	*
hybridization	CHEMBL1082043	0.831858	1100.0	*
substructure	CHEMBL1082042	0.916667	1400.0	*
graph	CHEMBL1082042	0.928571	1400.0	*
pubchem	CHEMBL1082042	0.879227	1400.0	*
cdk_maccs	CHEMBL1082043	0.897059	1100.0	*
klekota_roth	CHEMBL1082042	0.714286	1400.0	*

*** ic50 ACTIVITY *** value prediction
based on 12 locally validated fgps ---> 1425.881
["TT_bits", "substructure", "cdk_maccs", "AP_bits", "MFP1", "klekota_roth", "pubchem", "graph", "FP2", "FeatMFP1", "RDKit7", "hybridization"]

2 rank
Thrombin : Homo sapiens
score: 9.227 on CHEMBL204 based on 12 fingerprints

Fingerprint type	Ligand	Tanimoto Similarity	activity	Valid fg
FeatMFP1	CHEMBL179210	0.766667	11.0	*
MFP1	CHEMBL360684	0.666667	82000.0	*
RDKit7	CHEMBL366882	0.824427	95.0	*
Pattern	CHEMBL366882	0.921348	95.0	*
AP_bits	CHEMBL407142	0.652231	11200.0	*
TT_bits	CHEMBL366882	0.594059	95.0	*
FP2	CHEMBL366882	0.826667	95.0	*
hybridization	CHEMBL366882	0.756058	95.0	*
substructure	CHEMBL360684	0.913043	82000.0	*
graph	CHEMBL257898	0.760714	56.0	
pubchem	CHEMBL366882	0.892523	95.0	*
cdk_maccs	CHEMBL368894	0.805556	29.0	*
klekota_roth	CHEMBL289477	0.607692	42000.0	*

*** ic50 ACTIVITY *** value prediction
based on 12 locally validated fgps ---> 11129.924
["TT_bits", "substructure", "cdk_maccs", "Pattern", "FP2", "MFP1", "klekota_roth", "pubchem", "AP_bits", "FeatMFP1", "RDKit7", "hybridization"]

3 rank
Coagulation factor VII : Homo sapiens
score: 7.307 on CHEMBL3991 based on 10 fingerprints

Fingerprint type	Ligand	Tanimoto Similarity	activity	Valid fg
FeatMFP1	CHEMBL282830	0.718750	270.0	*
MFP1	CHEMBL1797527	0.555556	16000.0	*
RDKit7	CHEMBL27852	0.705432	200.0	*
Pattern	CHEMBL27852	0.847059	200.0	*
AP_bits	CHEMBL407142	0.652231	17800.0	*
TT_bits	CHEMBL27852	0.386792	200.0	
FP2	CHEMBL27852	0.729730	200.0	*
hybridization	CHEMBL27852	0.736089	200.0	*
substructure	CHEMBL339956	0.875000	3300.0	*
graph	CHEMBL27852	0.727941	200.0	

pubchem	CHEMBL282830	0.830918	270.0	*
cdk_maccs	CHEMBL1797527	0.746269	16000.0	
klekota_roth	CHEMBL1797527	0.655738	16000.0	*

*** ic50 ACTIVITY *** value prediction
based on 10 locally validated fgps ---> 3142.794
["substructure", "Pattern", "AP_bits", "MFP1", "klekota_roth", "pubchem", "FP2", "FeatMFP1", "RDKit7", "hybridization"]

4 rank
Coagulation factor X : Homo sapiens
score: 7.273 on CHEMBL244 based on 10 fingerprints

Fingerprint type	Ligand	Tanimoto Similarity	activity	Valid fg
FeatMFP1	CHEMBL282830	0.718750	270.0	*
MFP1	CHEMBL27852	0.545455	19.3	*
RDKit7	CHEMBL27852	0.705432	19.3	*
Pattern	CHEMBL27852	0.847059	19.3	*
AP_bits	CHEMBL407142	0.652231	3140.0	*
TT_bits	CHEMBL27852	0.386792	19.3	
FP2	CHEMBL27852	0.729730	19.3	*
hybridization	CHEMBL27852	0.736089	19.3	*
substructure	CHEMBL339956	0.875000	2700.0	*
graph	CHEMBL27852	0.727941	19.3	
pubchem	CHEMBL282830	0.830918	270.0	*
cdk_maccs	CHEMBL191264	0.792208	14300.0	
klekota_roth	CHEMBL41722	0.632812	11000.0	*

*** ic50 ACTIVITY *** value prediction
based on 10 locally validated fgps ---> 1156.327
["substructure", "Pattern", "AP_bits", "MFP1", "klekota_roth", "pubchem", "FP2", "FeatMFP1", "RDKit7", "hybridization"]

5 rank
Trypsin I : Homo sapiens
score: 6.579 on CHEMBL209 based on 9 fingerprints

Fingerprint type	Ligand	Tanimoto Similarity	activity	Valid fg
FeatMFP1	CHEMBL352928	0.709677	1.0	*
MFP1	CHEMBL352928	0.545455	1.0	*
RDKit7	CHEMBL352928	0.724138	1.0	*
Pattern	CHEMBL352928	0.870398	1.0	*
AP_bits	CHEMBL79245	0.581098	11000.0	*
TT_bits	CHEMBL352928	0.362832	1.0	
FP2	CHEMBL352928	0.735160	1.0	*
hybridization	CHEMBL83001	0.701183	3700.0	*
substructure	CHEMBL116297	0.875000	31600.0	*
graph	CHEMBL352928	0.709790	1.0	
pubchem	CHEMBL83001	0.836538	3700.0	*
cdk_maccs	CHEMBL169291	0.760000	2.0	
klekota_roth	CHEMBL163543	0.583942	115.0	

*** ic50 ACTIVITY *** value prediction
based on 9 locally validated fgps ---> 3323.134
["substructure", "Pattern", "AP_bits", "MFP1", "pubchem", "FP2", "FeatMFP1", "RDKit7", "hybridization"]

6 rank
Tissue-type plasminogen activator : Homo sapiens
score: 6.267 on CHEMBL1873 based on 9 fingerprints

Fingerprint type	Ligand	Tanimoto Similarity	activity	Valid fg
FeatMFP1	CHEMBL282830	0.718750	495.0	*
MFP1	CHEMBL27852	0.545455	93.0	*
RDKit7	CHEMBL27852	0.705432	93.0	*
Pattern	CHEMBL27852	0.847059	93.0	*

```
        AP_bits   CHEMBL27852            0.556497      93.0        *
        TT_bits   CHEMBL27852            0.386792      93.0
            FP2   CHEMBL27852            0.729730      93.0        *
  hybridization   CHEMBL27852            0.736089      93.0        *
   substructure  CHEMBL162461            0.833333    4010.0
          graph   CHEMBL27852            0.727941      93.0
        pubchem  CHEMBL282830            0.830918     495.0        *
      cdk_maccs   CHEMBL27852            0.716216      93.0
   klekota_roth  CHEMBL282830            0.596639     495.0        *
      *** ic50 ACTIVITY *** value prediction
      based on 9 locally validated fgps ---> 317.917
["Pattern", "AP_bits", "MFP1", "klekota_roth", "pubchem", "FP2",
"FeatMFP1", "RDKit7", "hybridization"]

7 rank
Plasminogen : Rattus norvegicus
      score: 6.267 on CHEMBL3204 based on 9 fingerprints
Fingerprint type        Ligand  Tanimoto Similarity  activity Valid fg
       FeatMFP1  CHEMBL282830            0.718750     415.0        *
           MFP1   CHEMBL27852            0.545455     251.0        *
         RDKit7   CHEMBL27852            0.705432     251.0        *
        Pattern   CHEMBL27852            0.847059     251.0        *
        AP_bits  CHEMBL436851            0.556978    1320.0        *
        TT_bits   CHEMBL27852            0.386792     251.0
            FP2   CHEMBL27852            0.729730     251.0        *
  hybridization   CHEMBL27852            0.736089     251.0        *
   substructure   CHEMBL27852            0.740741     251.0
          graph   CHEMBL27852            0.727941     251.0
        pubchem  CHEMBL282830            0.830918     415.0        *
      cdk_maccs   CHEMBL27852            0.716216     251.0
   klekota_roth  CHEMBL282830            0.596639     415.0        *
      *** ic50 ACTIVITY *** value prediction
      based on 9 locally validated fgps ---> 400.797
["Pattern", "AP_bits", "MFP1", "klekota_roth", "pubchem", "FP2",
"FeatMFP1", "RDKit7", "hybridization"]

8 rank
Urokinase-type plasminogen activator : Homo sapiens
      score: 6.267 on CHEMBL3286 based on 9 fingerprints
Fingerprint type        Ligand  Tanimoto Similarity  activity Valid fg
       FeatMFP1  CHEMBL282830            0.718750     600.0        *
           MFP1   CHEMBL27852            0.545455     335.0        *
         RDKit7   CHEMBL27852            0.705432     335.0        *
        Pattern   CHEMBL27852            0.847059     335.0        *
        AP_bits   CHEMBL27852            0.556497     335.0        *
        TT_bits   CHEMBL27852            0.386792     335.0
            FP2   CHEMBL27852            0.729730     335.0        *
  hybridization   CHEMBL27852            0.736089     335.0        *
   substructure   CHEMBL27852            0.740741     335.0
          graph   CHEMBL27852            0.727941     335.0
        pubchem  CHEMBL282830            0.830918     600.0        *
      cdk_maccs   CHEMBL27852            0.716216     335.0
   klekota_roth  CHEMBL282830            0.596639     600.0        *
      *** ic50 ACTIVITY *** value prediction
      based on 9 locally validated fgps ---> 495.620
["Pattern", "AP_bits", "MFP1", "klekota_roth", "pubchem", "FP2",
"FeatMFP1", "RDKit7", "hybridization"]

9 rank
Trypsin I : Bos taurus
```

```
      score: 5.746 on CHEMBL3769 based on 8 fingerprints
Fingerprint type        Ligand  Tanimoto Similarity  activity Valid fg
       FeatMFP1  CHEMBL176329            0.709677       5.8         *
           MFP1  CHEMBL176329            0.545455       5.8         *
         RDKit7  CHEMBL176329            0.730906       5.8         *
        Pattern  CHEMBL176385            0.860947     352.0         *
        AP_bits  CHEMBL176329            0.626628       5.8         *
        TT_bits  CHEMBL176329            0.450000       5.8
            FP2  CHEMBL176329            0.715556       5.8         *
  hybridization  CHEMBL176329            0.722222       5.8         *
   substructure  CHEMBL150497            0.833333     230.0
          graph  CHEMBL176016            0.703971   32000.0
        pubchem  CHEMBL176385            0.834951     352.0         *
      cdk_maccs  CHEMBL176329            0.763889       5.8
   klekota_roth  CHEMBL176329            0.549618       5.8
      *** ic50 ACTIVITY *** value prediction
      based on 8 locally validated fgps ---> 148.231
["Pattern", "AP_bits", "MFP1", "pubchem", "FP2", "FeatMFP1", "RDKit7",
"hybridization"]

10 rank
Lysosomal Pro-X carboxypeptidase : Homo sapiens
      score: 5.605 on CHEMBL2335 based on 8 fingerprints
Fingerprint type         Ligand  Tanimoto Similarity  activity Valid fg
       FeatMFP1  CHEMBL1256273            0.542857     850.0
           MFP1  CHEMBL1256277            0.454545     240.0
         RDKit7  CHEMBL1259114            0.742291     300.0         *
        Pattern  CHEMBL1256278            0.866856     570.0         *
        AP_bits  CHEMBL1256278            0.600291     570.0         *
        TT_bits  CHEMBL1259131            0.509804    5000.0         *
            FP2  CHEMBL1259114            0.753555     300.0         *
  hybridization  CHEMBL1259114            0.743760     300.0         *
   substructure  CHEMBL1256277            0.640000     240.0
          graph  CHEMBL1259114            0.773946     300.0         *
        pubchem  CHEMBL1259175            0.799107    4370.0
      cdk_maccs  CHEMBL1256277            0.684932     240.0
   klekota_roth  CHEMBL1256277            0.614815     240.0         *
      *** ic50 ACTIVITY *** value prediction
      based on 8 locally validated fgps ---> 2054.221
["TT_bits", "Pattern", "AP_bits", "klekota_roth", "graph", "FP2",
"RDKit7", "hybridization"]

11 rank
Tryptase beta-1 : Homo sapiens
      score: 4.476 on CHEMBL2617 based on 7 fingerprints
Fingerprint type        Ligand  Tanimoto Similarity  activity Valid fg
       FeatMFP1  CHEMBL178315            0.656250    9090.0
           MFP1  CHEMBL178315            0.576923    9090.0         *
         RDKit7  CHEMBL127185            0.705224       2.0         *
        Pattern  CHEMBL178315            0.809651    9090.0
        AP_bits  CHEMBL361835            0.500597   12400.0         *
        TT_bits  CHEMBL178315            0.544554    9090.0         *
            FP2  CHEMBL127185            0.675926       2.0         *
  hybridization  CHEMBL127185            0.708819       2.0         *
   substructure  CHEMBL127185            0.800000       2.0
          graph  CHEMBL127185            0.752852       2.0
        pubchem  CHEMBL127185            0.754902       2.0
      cdk_maccs  CHEMBL178315            0.783784    9090.0
   klekota_roth  CHEMBL178315            0.763780    9090.0         *
      *** ic50 ACTIVITY *** value prediction
```

```
     based on 7 locally validated fgps ---> 4628.473
["TT_bits", "AP_bits", "MFP1", "klekota_roth", "FP2", "RDKit7",
"hybridization"]
```

SwissTargetPrediction report:

Reference:
Gfeller D., Michielin O. & Zoete V.
Shaping the interaction landscape of bioactive molecules, *Bioinformatics* (2013) 29:3073-3079.

Query Molecule

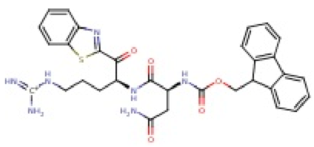

Frequency of Target Class

Target	Uniprot ID	Gene code	ChEMBL ID	Probability	# sim. cmpds (3D / 2D)	Target Class
Activation peptide fragment 1	P00734	F2	CHEMBL204		1464 / 332	Serine Protease
Alpha-trypsin chain 1 *(by homology)*	P07477	PRSS1	CHEMBL209		622 / 132	Serine Protease
Trypsin-3 *(by homology)*	P35030	PRSS3	CHEMBL4551		622 / 132	Serine Protease
Trypsin-2 *(by homology)*	P07478	PRSS2	CHEMBL3159		622 / 132	Serine Protease
Coagulation factor IXa heavy chain *(by homology)*	P00740	F9	CHEMBL2016		1257 / 152	Serine Protease
Factor X light chain	P00742	F10	CHEMBL244		1257 / 152	Serine Protease
Coagulation factor VII	P08709	F7	CHEMBL3991		1241 / 152	Serine Protease
Plasmin light chain B	P00747	PLG	CHEMBL1801		268 / 8	Serine Protease
Apolipoprotein(a) *(by homology)*	P08519	LPA			268 / 8	Serine Protease
Transmembrane protease serine 11D non-catalytic chain	O60235	TMPRSS11D	CHEMBL1795138		33 / 1	Enzyme
Transmembrane protease serine 11A *(by homology)*	Q6ZMR5	TMPRSS11A			33 / 1	Enzyme
Transmembrane protease serine 11F *(by homology)*	Q6ZWK6	TMPRSS11F			33 / 1	Enzyme
Transmembrane protease serine 11B *(by homology)*	Q86T26	TMPRSS11B			33 / 1	Enzyme
Transmembrane protease serine 11E *(by homology)*	Q9UL52	TMPRSS11E			33 / 1	Enzyme
Urokinase-type plasminogen activator long chain A	P00749	PLAU	CHEMBL3286		181 / 2	Serine Protease

Polypharmacology Browser 2 Prediction:

Targets predicted using NN(ECfp4) + NB(ECfp4). Save Table

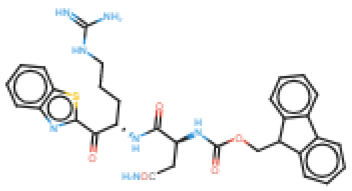

Query molecule

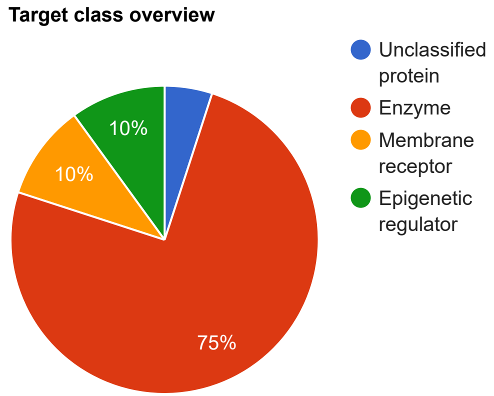

Rank	ChEMBL ID	Common name	Nearest neighbours
1	CHEMBL3018 (https://www.ebi.ac.uk/chembl/target/inspect/CHEMBL3018)	Matriptase	Show NN
2	CHEMBL209 (https://www.ebi.ac.uk/chembl/target/inspect/CHEMBL209)	Trypsin I	Show NN
3	CHEMBL1795139 (https://www.ebi.ac.uk/chembl/target/inspect/CHEMBL1795139)	Transmembrane protease serine 6	Show NN
4	CHEMBL204 (https://www.ebi.ac.uk/chembl/target/inspect/CHEMBL204)	Thrombin	Show NN
5	CHEMBL244 (https://www.ebi.ac.uk/chembl/target/inspect/CHEMBL244)	Coagulation factor X	Show NN
6	CHEMBL3991 (https://www.ebi.ac.uk/chembl/target/inspect/CHEMBL3991)	Coagulation factor VII	Show NN
7	CHEMBL2617 (https://www.ebi.ac.uk/chembl/target/inspect/CHEMBL2617)	Tryptase beta-1	Show NN
8	CHEMBL270 (https://www.ebi.ac.uk/chembl/target/inspect/CHEMBL270)	Mu opioid receptor	Show NN
9	CHEMBL325 (https://www.ebi.ac.uk/chembl/target/inspect/CHEMBL325)	Histone deacetylase 1	Show NN
10	CHEMBL2079849 (https://www.ebi.ac.uk/chembl/target/inspect/CHEMBL2079849)	Serine protease hepsin	Show NN
11	CHEMBL3351190 (https://www.ebi.ac.uk/chembl/target/inspect/CHEMBL3351190)	Hepatocyte growth factor activator	Show NN
12	CHEMBL3286 (https://www.ebi.ac.uk/chembl/target/inspect/CHEMBL3286)	Urokinase-type plasminogen activator	Show NN
13	CHEMBL2000 (https://www.ebi.ac.uk/chembl/target/inspect/CHEMBL2000)	Plasma kallikrein	Show NN
14	CHEMBL2820 (https://www.ebi.ac.uk/chembl/target/inspect/CHEMBL2820)	Coagulation factor XI	Show NN
15	CHEMBL4777 (https://www.ebi.ac.uk/chembl/target/inspect/CHEMBL4777)	Neuropeptide Y receptor type 1	Show NN
16	CHEMBL1801 (https://www.ebi.ac.uk/chembl/target/inspect/CHEMBL1801)	Plasminogen	Show NN
17	CHEMBL1865 (https://www.ebi.ac.uk/chembl/target/inspect/CHEMBL1865)	Histone deacetylase 6	Show NN
18	CHEMBL333 (https://www.ebi.ac.uk/chembl/target/inspect/CHEMBL333)	Matrix metalloproteinase-2	Show NN

19	CHEMBL4822 (https://www.ebi.ac.uk/chembl/target/inspect/CHEMBL4822)	Beta-secretase 1	Show NN
20	CHEMBL332 (https://www.ebi.ac.uk/chembl/target/inspect/CHEMBL332)	Matrix metalloproteinase-1	Show NN
