# Supplementary material for: Accelerating Drug Discovery by Early Protein Drug Target Prediction Based on a Multi-Fingerprint Similarity Search
Source: Molecules. 2019 Jun 14;24(12):2233. doi: 10.3390/molecules24122233 (PMC6631269; doi:10.3390/molecules24122233)
Supplement: Supplementary file 1 [file molecules-24-02233-s001.zip › File_S2.zip!/File_S2/Target_Prediction_compound#17.pdf]

MuSSel Prediction k_i:

```
1 rank
p53-binding protein Mdm-2 : Homo sapiens
      score: 6.243 on CHEMBL5023 based on 8 fingerprints
Fingerprint type         Ligand  Tanimoto Similarity  activity Valid fg
       FeatMFP1  CHEMBL3355423            0.687500   1238.00
           MFP1  CHEMBL3355430            0.606557      0.16        *
         RDKit7  CHEMBL3355430            0.917831      0.16        *
        Pattern  CHEMBL3355430            0.794145      0.16
        AP_bits  CHEMBL3355430            0.447471      0.16
        TT_bits  CHEMBL3355430            0.671533      0.16        *
            FP2  CHEMBL3355430            0.735426      0.16        *
  hybridization  CHEMBL3355430            0.777305      0.16        *
   substructure  CHEMBL3355430            0.777778      0.16
          graph  CHEMBL3355430            0.971429      0.16        *
        pubchem   CHEMBL409889            0.841530     39.00
      cdk_maccs  CHEMBL3355421            0.891892      1.70        *
   klekota_roth  CHEMBL3355430            0.670588      0.16        *
      *** ki ACTIVITY *** value prediction
      based on 8 locally validated fgps ---> 162.342
["TT_bits", "cdk_maccs", "graph", "MFP1", "klekota_roth", "FP2",
"RDKit7", "hybridization"]
```

MuSSel Prediction IC_{50}:

```
1 rank
p53-binding protein Mdm-2 : Homo sapiens
      score: 8.586 on CHEMBL5023 based on 11 fingerprints
Fingerprint type         Ligand  Tanimoto Similarity  activity Valid fg
       FeatMFP1  CHEMBL3693968             0.733333     100.0        *
           MFP1  CHEMBL3693972             0.612903     100.0        *
         RDKit7  CHEMBL3355430             0.917831       4.4        *
        Pattern  CHEMBL3693970             0.825922     100.0
        AP_bits  CHEMBL3703353             0.639013      80.1        *
        TT_bits  CHEMBL3355430             0.671533       4.4        *
            FP2  CHEMBL3693974             0.761062     100.0        *
  hybridization  CHEMBL3693976             0.779167     100.0        *
   substructure  CHEMBL3693969             0.791667     100.0
          graph  CHEMBL3355430             0.971429       4.4        *
        pubchem  CHEMBL3693982             0.844920     100.0        *
      cdk_maccs  CHEMBL3693982             0.931507     100.0        *
   klekota_roth  CHEMBL3693983             0.723529     100.0        *
      *** ic50 ACTIVITY *** value prediction
      based on 11 locally validated fgps ---> 289.600
["TT_bits", "cdk_maccs", "AP_bits", "MFP1", "klekota_roth", "pubchem",
"graph", "FP2", "FeatMFP1", "RDKit7", "hybridization"]

2 rank
Cholinesterase : Equus caballus
      score: 5.506 on CHEMBL5763 based on 7 fingerprints
Fingerprint type         Ligand  Tanimoto Similarity  activity Valid fg
       FeatMFP1  CHEMBL3633479             0.516129    2650.0
           MFP1  CHEMBL3235943             0.356164   27120.0
         RDKit7  CHEMBL3235941             0.827957   11760.0        *
        Pattern  CHEMBL3235948             0.874459   26300.0        *
        AP_bits  CHEMBL2204447             0.587997      49.7        *
        TT_bits  CHEMBL3235943             0.378531   27120.0
            FP2  CHEMBL3235952             0.583333   35860.0
  hybridization  CHEMBL2332534             0.676225   11740.0        *
   substructure  CHEMBL3415565             0.680000    2377.0
          graph  CHEMBL2380672             0.814815    3130.0        *
        pubchem  CHEMBL2380667             0.870968    9650.0        *
      cdk_maccs  CHEMBL2332531             0.853333    9450.0        *
   klekota_roth  CHEMBL2380672             0.549223    3130.0
      *** ic50 ACTIVITY *** value prediction
      based on 7 locally validated fgps ---> 13259.989
["cdk_maccs", "Pattern", "AP_bits", "pubchem", "graph", "RDKit7",
"hybridization"]

3 rank
Acetylcholinesterase : Homo sapiens
      score: 5.442 on CHEMBL220 based on 7 fingerprints
Fingerprint type         Ligand  Tanimoto Similarity  activity Valid fg
       FeatMFP1  CHEMBL1288823             0.529412   9860.00
           MFP1  CHEMBL1288825             0.347826  12200.00
         RDKit7  CHEMBL1288829             0.811321   4200.00        *
        Pattern  CHEMBL3115036             0.855035   6140.00        *
        AP_bits   CHEMBL575674             0.560129      5.96        *
        TT_bits  CHEMBL1288825             0.357955  12200.00
            FP2  CHEMBL1288823             0.575875   9860.00
  hybridization  CHEMBL2332534             0.676225  13640.00        *
   substructure  CHEMBL1288823             0.769231   9860.00
          graph  CHEMBL2380672             0.814815  29510.00        *
        pubchem  CHEMBL2380667             0.870968   5810.00        *
      cdk_maccs  CHEMBL2332531             0.853333   6960.00        *
```

```
   klekota_roth  CHEMBL2380672             0.549223  29510.00
      *** ic50 ACTIVITY *** value prediction
      based on 7 locally validated fgps ---> 11328.198
["cdk_maccs", "Pattern", "AP_bits", "pubchem", "graph", "RDKit7",
"hybridization"]
```

SwissTargetPrediction report:

Reference:
Gfeller D., Michielin O. & Zoete V.
Shaping the interaction landscape of bioactive molecules, *Bioinformatics* (2013) 29:3073-3079.

Query Molecule

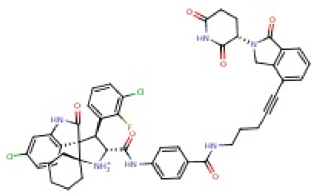

Frequency of Target Class

Target	Uniprot ID	Gene code	ChEMBL ID	Probability	# sim. cmpds (3D / 2D)	Target Class
Microtubule-associated protein tau	P10636	MAPT	CHEMBL1293224		4 / 2	Unclassified
E3 ubiquitin-protein ligase Mdm2	Q00987	MDM2	CHEMBL5023		0 / 27	Nuclear other
Cholinesterase *(by homology)*	P06276	BCHE	CHEMBL1914		18 / 22	Enzyme
Acetylcholinesterase	P22303	ACHE	CHEMBL220		18 / 22	Enzyme
Muscleblind-like protein 1	Q9NR56	MBNL1	CHEMBL1293317		2 / 3	Unclassified
Muscleblind-like protein 2 *(by homology)*	Q5VZF2	MBNL2			2 / 3	Unclassified
Muscleblind-like protein 3 *(by homology)*	Q9NUK0	MBNL3			2 / 3	Unclassified
Calcitonin receptor *(by homology)*	P30988	CALCR	CHEMBL1832		20 / 8	Membrane receptor
Calcitonin gene-related peptide type 1 receptor	Q16602	CALCRL	CHEMBL3798		21 / 14	Membrane receptor
D(2) dopamine receptor	P14416	DRD2	CHEMBL217		44 / 34	Membrane receptor
D(4) dopamine receptor	P21917	DRD4	CHEMBL219		24 / 30	Membrane receptor
D(3) dopamine receptor	P35462	DRD3	CHEMBL234		33 / 30	Membrane receptor
Gastrin/cholecystokinin type B receptor	P32239	CCKBR	CHEMBL298		8 / 25	Membrane receptor
Cathepsin L1 light chain	P07711	CTSL1	CHEMBL3837		7 / 22	Cysteine Protease
Cathepsin S	P25774	CTSS	CHEMBL2954		7 / 22	Cysteine Protease

Polypharmacology Browser 2 not predicted
