# Supplementary material for: Accelerating Drug Discovery by Early Protein Drug Target Prediction Based on a Multi-Fingerprint Similarity Search
Source: Molecules. 2019 Jun 14;24(12):2233. doi: 10.3390/molecules24122233 (PMC6631269; doi:10.3390/molecules24122233)
Supplement: Supplementary file 1 [file molecules-24-02233-s001.zip › File_S2.zip!/File_S2/Target_Prediction_compound#18.pdf]

MuSSel Prediction k_i:

1 rank
Vascular endothelial growth factor receptor 3 : Homo sapiens
score: 7.106 on CHEMBL1955 based on 9 fingerprints

Fingerprint type	Ligand	Tanimoto Similarity	activity	Valid fg
FeatMFP1	CHEMBL939	0.750000	6309.57	*
MFP1	CHEMBL939	0.688889	6309.57	*
RDKit7	CHEMBL939	0.805133	6309.57	*
Pattern	CHEMBL124660	0.861301	3162.28	*
AP_bits	CHEMBL215152	0.516266	1258.93	
TT_bits	CHEMBL939	0.635135	6309.57	*
FP2	CHEMBL939	0.782313	6309.57	*
hybridization	CHEMBL939	0.816471	6309.57	*
substructure	CHEMBL1825138	0.818182	1584.89	
graph	CHEMBL939	0.777778	6309.57	
pubchem	CHEMBL939	0.925764	6309.57	*
cdk_maccs	CHEMBL939	0.840580	6309.57	*
klekota_roth	CHEMBL939	0.600000	6309.57	

*** ki ACTIVITY *** value prediction
based on 9 locally validated fgps ---> 5792.373
["TT_bits", "cdk_maccs", "Pattern", "MFP1", "pubchem", "FP2", "FeatMFP1", "RDKit7", "hybridization"]

2 rank
Tyrosine-protein kinase LCK : Homo sapiens
score: 6.797 on CHEMBL258 based on 9 fingerprints

Fingerprint type	Ligand	Tanimoto Similarity	activity	Valid fg
FeatMFP1	CHEMBL939	0.750000	398.11	*
MFP1	CHEMBL939	0.688889	398.11	*
RDKit7	CHEMBL939	0.805133	398.11	*
Pattern	CHEMBL202721	0.856637	63.10	
AP_bits	CHEMBL202721	0.547865	63.10	*
TT_bits	CHEMBL939	0.635135	398.11	*
FP2	CHEMBL939	0.782313	398.11	*
hybridization	CHEMBL939	0.816471	398.11	*
substructure	CHEMBL230686	0.850000	29.40	
graph	CHEMBL939	0.777778	398.11	
pubchem	CHEMBL939	0.925764	398.11	*
cdk_maccs	CHEMBL24828	0.845070	39.81	*
klekota_roth	CHEMBL939	0.600000	398.11	

*** ki ACTIVITY *** value prediction
based on 9 locally validated fgps ---> 340.928
["TT_bits", "cdk_maccs", "AP_bits", "MFP1", "pubchem", "FP2", "FeatMFP1", "RDKit7", "hybridization"]

3 rank
Mitogen-activated protein kinase kinase kinase kinase 5 : Homo sapiens
score: 6.797 on CHEMBL4852 based on 9 fingerprints

Fingerprint type	Ligand	Tanimoto Similarity	activity	Valid fg
FeatMFP1	CHEMBL939	0.750000	1584.89	*
MFP1	CHEMBL939	0.688889	1584.89	*
RDKit7	CHEMBL939	0.805133	1584.89	*
Pattern	CHEMBL202721	0.856637	31.62	
AP_bits	CHEMBL202721	0.547865	31.62	*
TT_bits	CHEMBL939	0.635135	1584.89	*
FP2	CHEMBL939	0.782313	1584.89	*
hybridization	CHEMBL939	0.816471	1584.89	*
substructure	CHEMBL95692	0.850000	1584.89	
graph	CHEMBL939	0.777778	1584.89	
pubchem	CHEMBL939	0.925764	1584.89	*
cdk_maccs	CHEMBL24828	0.845070	316.23	*

```
   klekota_roth     CHEMBL939             0.600000   1584.89
      *** ki ACTIVITY *** value prediction
      based on 9 locally validated fgps ---> 1341.636
["TT_bits", "cdk_maccs", "AP_bits", "MFP1", "pubchem", "FP2", "FeatMFP1",
"RDKit7", "hybridization"]

4 rank
MAP kinase p38 alpha : Homo sapiens
      score: 6.249 on CHEMBL260 based on 8 fingerprints
Fingerprint type         Ligand  Tanimoto Similarity  activity Valid fg
       FeatMFP1      CHEMBL939             0.750000    501.19        *
           MFP1      CHEMBL939             0.688889    501.19        *
         RDKit7      CHEMBL939             0.805133    501.19        *
        Pattern      CHEMBL939             0.822785    501.19
        AP_bits  CHEMBL1256039             0.509091    885.00
        TT_bits      CHEMBL939             0.635135    501.19        *
            FP2      CHEMBL939             0.782313    501.19        *
  hybridization      CHEMBL939             0.816471    501.19        *
   substructure    CHEMBL95692             0.850000     36.00
          graph      CHEMBL939             0.777778    501.19
        pubchem      CHEMBL939             0.925764    501.19        *
      cdk_maccs    CHEMBL24828             0.845070   2511.89        *
   klekota_roth      CHEMBL939             0.600000    501.19
      *** ki ACTIVITY *** value prediction
      based on 8 locally validated fgps ---> 627.194
["TT_bits", "cdk_maccs", "MFP1", "pubchem", "FP2", "FeatMFP1", "RDKit7",
"hybridization"]

5 rank
Tyrosine-protein kinase receptor UFO : Homo sapiens
      score: 6.249 on CHEMBL4895 based on 8 fingerprints
Fingerprint type         Ligand  Tanimoto Similarity  activity Valid fg
       FeatMFP1      CHEMBL939             0.750000  1584.890        *
           MFP1      CHEMBL939             0.688889  1584.890        *
         RDKit7      CHEMBL939             0.805133  1584.890        *
        Pattern  CHEMBL1983268             0.824959    39.810
        AP_bits      CHEMBL939             0.507317  1584.890
        TT_bits      CHEMBL939             0.635135  1584.890        *
            FP2      CHEMBL939             0.782313  1584.890        *
  hybridization      CHEMBL939             0.816471  1584.890        *
   substructure  CHEMBL1825138             0.818182     5.012
          graph      CHEMBL939             0.777778  1584.890
        pubchem      CHEMBL939             0.925764  1584.890        *
      cdk_maccs    CHEMBL24828             0.845070   251.190        *
   klekota_roth      CHEMBL939             0.600000  1584.890
      *** ki ACTIVITY *** value prediction
      based on 8 locally validated fgps ---> 1501.312
["TT_bits", "cdk_maccs", "MFP1", "pubchem", "FP2", "FeatMFP1", "RDKit7",
"hybridization"]

6 rank
Mitogen-activated protein kinase kinase kinase kinase 4 : Homo sapiens
      score: 6.249 on CHEMBL6166 based on 8 fingerprints
Fingerprint type         Ligand  Tanimoto Similarity  activity Valid fg
       FeatMFP1      CHEMBL939             0.750000   1000.00        *
           MFP1      CHEMBL939             0.688889   1000.00        *
         RDKit7      CHEMBL939             0.805133   1000.00        *
        Pattern  CHEMBL1983268             0.824959   1995.26
        AP_bits   CHEMBL215152             0.516266   3162.28
        TT_bits      CHEMBL939             0.635135   1000.00        *
```

```
           FP2      CHEMBL939           0.782313   1000.00        *
  hybridization      CHEMBL939           0.816471   1000.00        *
   substructure    CHEMBL95692           0.850000    794.33
          graph      CHEMBL939           0.777778   1000.00
        pubchem      CHEMBL939           0.925764   1000.00        *
      cdk_maccs    CHEMBL24828           0.845070    158.49        *
   klekota_roth      CHEMBL939           0.600000   1000.00
     *** ki ACTIVITY *** value prediction
     based on 8 locally validated fgps ---> 947.266
["TT_bits", "cdk_maccs", "MFP1", "pubchem", "FP2", "FeatMFP1", "RDKit7",
"hybridization"]

7 rank
Homeodomain-interacting protein kinase 4 : Homo sapiens
     score: 6.244 on CHEMBL1075167 based on 8 fingerprints
Fingerprint type         Ligand  Tanimoto Similarity  activity Valid fg
       FeatMFP1      CHEMBL939           0.750000   1258.93        *
           MFP1      CHEMBL939           0.688889   1258.93        *
         RDKit7      CHEMBL939           0.805133   1258.93        *
        Pattern  CHEMBL1968705           0.829787   5011.87
        AP_bits      CHEMBL939           0.507317   1258.93
        TT_bits      CHEMBL939           0.635135   1258.93        *
            FP2      CHEMBL939           0.782313   1258.93        *
  hybridization      CHEMBL939           0.816471   1258.93        *
   substructure      CHEMBL939           0.750000   1258.93
          graph      CHEMBL939           0.777778   1258.93
        pubchem      CHEMBL939           0.925764   1258.93        *
      cdk_maccs      CHEMBL939           0.840580   1258.93        *
   klekota_roth      CHEMBL939           0.600000   1258.93
     *** ki ACTIVITY *** value prediction
     based on 8 locally validated fgps ---> 1258.930
["TT_bits", "cdk_maccs", "MFP1", "pubchem", "FP2", "FeatMFP1", "RDKit7",
"hybridization"]

8 rank
Receptor protein-tyrosine kinase erbB-2 : Homo sapiens
     score: 6.244 on CHEMBL1824 based on 8 fingerprints
Fingerprint type     Ligand  Tanimoto Similarity  activity Valid fg
       FeatMFP1  CHEMBL939           0.750000    25.120        *
           MFP1  CHEMBL939           0.688889    25.120        *
         RDKit7  CHEMBL939           0.805133    25.120        *
        Pattern  CHEMBL554           0.855025     1.585
        AP_bits  CHEMBL939           0.507317    25.120
        TT_bits  CHEMBL939           0.635135    25.120        *
            FP2  CHEMBL939           0.782313    25.120        *
  hybridization  CHEMBL939           0.816471    25.120        *
   substructure  CHEMBL554           0.809524     1.585
          graph  CHEMBL939           0.777778    25.120
        pubchem  CHEMBL939           0.925764    25.120        *
      cdk_maccs  CHEMBL939           0.840580    25.120        *
   klekota_roth  CHEMBL939           0.600000    25.120
     *** ki ACTIVITY *** value prediction
     based on 8 locally validated fgps ---> 23.760
["TT_bits", "cdk_maccs", "MFP1", "pubchem", "FP2", "FeatMFP1", "RDKit7",
"hybridization"]

9 rank
Epidermal growth factor receptor erbB1 : Homo sapiens
     score: 6.244 on CHEMBL203 based on 8 fingerprints
Fingerprint type         Ligand  Tanimoto Similarity  activity Valid fg
```

```
       FeatMFP1      CHEMBL939             0.750000      0.40        *
           MFP1      CHEMBL939             0.688889      0.40        *
         RDKit7      CHEMBL939             0.805133      0.40        *
        Pattern  CHEMBL1999749             0.825040      6.31
        AP_bits   CHEMBL215152             0.516266    501.19
        TT_bits      CHEMBL939             0.635135      0.40        *
            FP2      CHEMBL939             0.782313      0.40        *
  hybridization      CHEMBL939             0.816471      0.40        *
   substructure  CHEMBL1682560             0.761905      8.00
          graph      CHEMBL939             0.777778      0.40
        pubchem      CHEMBL939             0.925764      0.40        *
      cdk_maccs      CHEMBL939             0.840580      0.40        *
   klekota_roth      CHEMBL939             0.600000      0.40
      *** ki ACTIVITY *** value prediction
      based on 8 locally validated fgps ---> 0.400
["TT_bits", "cdk_maccs", "MFP1", "pubchem", "FP2", "FeatMFP1", "RDKit7",
"hybridization"]

10 rank
Serine/threonine-protein kinase PIM1 : Homo sapiens
      score: 6.244 on CHEMBL2147 based on 8 fingerprints
Fingerprint type         Ligand  Tanimoto Similarity  activity Valid fg
       FeatMFP1      CHEMBL939             0.750000   1995.26        *
           MFP1      CHEMBL939             0.688889   1995.26        *
         RDKit7      CHEMBL939             0.805133   1995.26        *
        Pattern      CHEMBL939             0.822785   1995.26
        AP_bits      CHEMBL939             0.507317   1995.26
        TT_bits      CHEMBL939             0.635135   1995.26        *
            FP2      CHEMBL939             0.782313   1995.26        *
  hybridization      CHEMBL939             0.816471   1995.26        *
   substructure  CHEMBL3634777             0.850000      3.40
          graph      CHEMBL939             0.777778   1995.26
        pubchem      CHEMBL939             0.925764   1995.26        *
      cdk_maccs      CHEMBL939             0.840580   1995.26        *
   klekota_roth      CHEMBL939             0.600000   1995.26
      *** ki ACTIVITY *** value prediction
      based on 8 locally validated fgps ---> 1995.260
["TT_bits", "cdk_maccs", "MFP1", "pubchem", "FP2", "FeatMFP1", "RDKit7",
"hybridization"]

11 rank
Tyrosine-protein kinase BLK : Homo sapiens
      score: 6.244 on CHEMBL2250 based on 8 fingerprints
Fingerprint type         Ligand  Tanimoto Similarity  activity Valid fg
       FeatMFP1      CHEMBL939             0.750000   1000.00        *
           MFP1      CHEMBL939             0.688889   1000.00        *
         RDKit7      CHEMBL939             0.805133   1000.00        *
        Pattern  CHEMBL1999749             0.825040     12.59
        AP_bits   CHEMBL215152             0.516266    630.96
        TT_bits      CHEMBL939             0.635135   1000.00        *
            FP2      CHEMBL939             0.782313   1000.00        *
  hybridization      CHEMBL939             0.816471   1000.00        *
   substructure  CHEMBL1825138             0.818182    630.96
          graph      CHEMBL939             0.777778   1000.00
        pubchem      CHEMBL939             0.925764   1000.00        *
      cdk_maccs      CHEMBL939             0.840580   1000.00        *
   klekota_roth      CHEMBL939             0.600000   1000.00
      *** ki ACTIVITY *** value prediction
      based on 8 locally validated fgps ---> 1000.000
```

```
["TT_bits", "cdk_maccs", "MFP1", "pubchem", "FP2", "FeatMFP1", "RDKit7",
"hybridization"]

12 rank
Phosphorylase kinase gamma subunit 2 : Homo sapiens
      score: 6.244 on CHEMBL2349 based on 8 fingerprints
Fingerprint type         Ligand  Tanimoto Similarity  activity Valid fg
       FeatMFP1      CHEMBL939             0.750000   3162.28        *
           MFP1      CHEMBL939             0.688889   3162.28        *
         RDKit7      CHEMBL939             0.805133   3162.28        *
        Pattern  CHEMBL1968705             0.829787    630.96
        AP_bits      CHEMBL939             0.507317   3162.28
        TT_bits      CHEMBL939             0.635135   3162.28        *
            FP2      CHEMBL939             0.782313   3162.28        *
  hybridization      CHEMBL939             0.816471   3162.28        *
   substructure  CHEMBL1825138             0.818182   3162.28
          graph      CHEMBL939             0.777778   3162.28
        pubchem      CHEMBL939             0.925764   3162.28        *
      cdk_maccs      CHEMBL939             0.840580   3162.28        *
   klekota_roth      CHEMBL939             0.600000   3162.28
      *** ki ACTIVITY *** value prediction
      based on 8 locally validated fgps ---> 3162.280
["TT_bits", "cdk_maccs", "MFP1", "pubchem", "FP2", "FeatMFP1", "RDKit7",
"hybridization"]

13 rank
Serine/threonine-protein kinase Chk2 : Homo sapiens
      score: 6.244 on CHEMBL2527 based on 8 fingerprints
Fingerprint type         Ligand  Tanimoto Similarity  activity Valid fg
       FeatMFP1      CHEMBL939             0.750000    630.96        *
           MFP1      CHEMBL939             0.688889    630.96        *
         RDKit7      CHEMBL939             0.805133    630.96        *
        Pattern  CHEMBL1968705             0.829787   1000.00
        AP_bits   CHEMBL215152             0.516266   1995.26
        TT_bits      CHEMBL939             0.635135    630.96        *
            FP2      CHEMBL939             0.782313    630.96        *
  hybridization      CHEMBL939             0.816471    630.96        *
   substructure   CHEMBL491473             0.772727   2511.89
          graph      CHEMBL939             0.777778    630.96
        pubchem      CHEMBL939             0.925764    630.96        *
      cdk_maccs      CHEMBL939             0.840580    630.96        *
   klekota_roth      CHEMBL939             0.600000    630.96
      *** ki ACTIVITY *** value prediction
      based on 8 locally validated fgps ---> 630.960
["TT_bits", "cdk_maccs", "MFP1", "pubchem", "FP2", "FeatMFP1", "RDKit7",
"hybridization"]

14 rank
Protein kinase C nu : Homo sapiens
      score: 6.244 on CHEMBL2595 based on 8 fingerprints
Fingerprint type         Ligand  Tanimoto Similarity  activity Valid fg
       FeatMFP1      CHEMBL939             0.750000   1258.93        *
           MFP1      CHEMBL939             0.688889   1258.93        *
         RDKit7      CHEMBL939             0.805133   1258.93        *
        Pattern  CHEMBL1968705             0.829787   1584.89
        AP_bits      CHEMBL939             0.507317   1258.93
        TT_bits      CHEMBL939             0.635135   1258.93        *
            FP2      CHEMBL939             0.782313   1258.93        *
  hybridization      CHEMBL939             0.816471   1258.93        *
   substructure    CHEMBL95692             0.850000   3162.28
```

```
          graph      CHEMBL939            0.777778   1258.93
        pubchem      CHEMBL939            0.925764   1258.93        *
      cdk_maccs      CHEMBL939            0.840580   1258.93        *
   klekota_roth      CHEMBL939            0.600000   1258.93
      *** ki ACTIVITY *** value prediction
      based on 8 locally validated fgps ---> 1258.930
["TT_bits", "cdk_maccs", "MFP1", "pubchem", "FP2", "FeatMFP1", "RDKit7",
"hybridization"]

15 rank
c-Jun N-terminal kinase 3 : Homo sapiens
      score: 6.244 on CHEMBL2637 based on 8 fingerprints
Fingerprint type     Ligand  Tanimoto Similarity  activity Valid fg
       FeatMFP1  CHEMBL939            0.750000    794.33        *
           MFP1  CHEMBL939            0.688889    794.33        *
         RDKit7  CHEMBL939            0.805133    794.33        *
        Pattern  CHEMBL939            0.822785    794.33
        AP_bits  CHEMBL939            0.507317    794.33
        TT_bits  CHEMBL939            0.635135    794.33        *
            FP2  CHEMBL939            0.782313    794.33        *
  hybridization  CHEMBL939            0.816471    794.33        *
   substructure  CHEMBL939            0.750000    794.33
          graph  CHEMBL939            0.777778    794.33
        pubchem  CHEMBL939            0.925764    794.33        *
      cdk_maccs  CHEMBL939            0.840580    794.33        *
   klekota_roth  CHEMBL939            0.600000    794.33
      *** ki ACTIVITY *** value prediction
      based on 8 locally validated fgps ---> 794.330
["TT_bits", "cdk_maccs", "MFP1", "pubchem", "FP2", "FeatMFP1", "RDKit7",
"hybridization"]

16 rank
CaM kinase II delta : Homo sapiens
      score: 6.244 on CHEMBL2801 based on 8 fingerprints
Fingerprint type         Ligand  Tanimoto Similarity  activity Valid fg
       FeatMFP1      CHEMBL939            0.750000   2511.89        *
           MFP1      CHEMBL939            0.688889   2511.89        *
         RDKit7      CHEMBL939            0.805133   2511.89        *
        Pattern  CHEMBL1968705            0.829787   1000.00
        AP_bits      CHEMBL939            0.507317   2511.89
        TT_bits      CHEMBL939            0.635135   2511.89        *
            FP2      CHEMBL939            0.782313   2511.89        *
  hybridization      CHEMBL939            0.816471   2511.89        *
   substructure  CHEMBL1965702            0.800000   1995.26
          graph      CHEMBL939            0.777778   2511.89
        pubchem      CHEMBL939            0.925764   2511.89        *
      cdk_maccs      CHEMBL939            0.840580   2511.89        *
   klekota_roth      CHEMBL939            0.600000   2511.89
      *** ki ACTIVITY *** value prediction
      based on 8 locally validated fgps ---> 2511.890
["TT_bits", "cdk_maccs", "MFP1", "pubchem", "FP2", "FeatMFP1", "RDKit7",
"hybridization"]

17 rank
Casein kinase I delta : Homo sapiens
      score: 6.244 on CHEMBL2828 based on 8 fingerprints
Fingerprint type        Ligand  Tanimoto Similarity  activity Valid fg
       FeatMFP1     CHEMBL939            0.750000   7943.28        *
           MFP1     CHEMBL939            0.688889   7943.28        *
         RDKit7     CHEMBL939            0.805133   7943.28        *
```

```
        Pattern     CHEMBL939             0.822785   7943.28
        AP_bits  CHEMBL215152             0.516266   5011.87
        TT_bits     CHEMBL939             0.635135   7943.28        *
            FP2     CHEMBL939             0.782313   7943.28        *
  hybridization     CHEMBL939             0.816471   7943.28        *
   substructure   CHEMBL95692             0.850000     19.95
          graph     CHEMBL939             0.777778   7943.28
        pubchem     CHEMBL939             0.925764   7943.28        *
      cdk_maccs     CHEMBL939             0.840580   7943.28        *
   klekota_roth     CHEMBL939             0.600000   7943.28
      *** ki ACTIVITY *** value prediction
      based on 8 locally validated fgps ---> 7943.280
["TT_bits", "cdk_maccs", "MFP1", "pubchem", "FP2", "FeatMFP1", "RDKit7",
"hybridization"]

18 rank
Receptor protein-tyrosine kinase erbB-4 : Homo sapiens
      score: 6.244 on CHEMBL3009 based on 8 fingerprints
Fingerprint type        Ligand  Tanimoto Similarity  activity Valid fg
       FeatMFP1     CHEMBL939             0.750000    158.49        *
           MFP1     CHEMBL939             0.688889    158.49        *
         RDKit7     CHEMBL939             0.805133    158.49        *
        Pattern     CHEMBL554             0.855025     25.12
        AP_bits  CHEMBL215152             0.516266    199.53
        TT_bits     CHEMBL939             0.635135    158.49        *
            FP2     CHEMBL939             0.782313    158.49        *
  hybridization     CHEMBL939             0.816471    158.49        *
   substructure     CHEMBL554             0.809524     25.12
          graph     CHEMBL939             0.777778    158.49
        pubchem     CHEMBL939             0.925764    158.49        *
      cdk_maccs     CHEMBL939             0.840580    158.49        *
   klekota_roth     CHEMBL939             0.600000    158.49
      *** ki ACTIVITY *** value prediction
      based on 8 locally validated fgps ---> 150.784
["TT_bits", "cdk_maccs", "MFP1", "pubchem", "FP2", "FeatMFP1", "RDKit7",
"hybridization"]

19 rank
Tyrosine-protein kinase HCK : Homo sapiens
      score: 6.244 on CHEMBL3234 based on 8 fingerprints
Fingerprint type     Ligand  Tanimoto Similarity  activity Valid fg
       FeatMFP1  CHEMBL939             0.750000   2511.89        *
           MFP1  CHEMBL939             0.688889   2511.89        *
         RDKit7  CHEMBL939             0.805133   2511.89        *
        Pattern  CHEMBL939             0.822785   2511.89
        AP_bits  CHEMBL939             0.507317   2511.89
        TT_bits  CHEMBL939             0.635135   2511.89        *
            FP2  CHEMBL939             0.782313   2511.89        *
  hybridization  CHEMBL939             0.816471   2511.89        *
   substructure  CHEMBL939             0.750000   2511.89
          graph  CHEMBL939             0.777778   2511.89
        pubchem  CHEMBL939             0.925764   2511.89        *
      cdk_maccs  CHEMBL939             0.840580   2511.89        *
   klekota_roth  CHEMBL939             0.600000   2511.89
      *** ki ACTIVITY *** value prediction
      based on 8 locally validated fgps ---> 2511.890
["TT_bits", "cdk_maccs", "MFP1", "pubchem", "FP2", "FeatMFP1", "RDKit7",
"hybridization"]

20 rank
```

```
Interleukin-1 receptor-associated kinase 1 : Homo sapiens
     score: 6.244 on CHEMBL3357 based on 8 fingerprints
Fingerprint type        Ligand  Tanimoto Similarity  activity Valid fg
       FeatMFP1      CHEMBL939             0.750000    316.23        *
           MFP1      CHEMBL939             0.688889    316.23        *
         RDKit7      CHEMBL939             0.805133    316.23        *
        Pattern      CHEMBL939             0.822785    316.23
        AP_bits      CHEMBL939             0.507317    316.23
        TT_bits      CHEMBL939             0.635135    316.23        *
            FP2      CHEMBL939             0.782313    316.23        *
  hybridization      CHEMBL939             0.816471    316.23        *
   substructure  CHEMBL1825138             0.818182    199.53
          graph      CHEMBL939             0.777778    316.23
        pubchem      CHEMBL939             0.925764    316.23        *
      cdk_maccs      CHEMBL939             0.840580    316.23        *
   klekota_roth      CHEMBL939             0.600000    316.23
     *** ki ACTIVITY *** value prediction
     based on 8 locally validated fgps ---> 316.230
["TT_bits", "cdk_maccs", "MFP1", "pubchem", "FP2", "FeatMFP1", "RDKit7",
"hybridization"]

21 rank
Interleukin-1 receptor-associated kinase 4 : Homo sapiens
     score: 6.244 on CHEMBL3778 based on 8 fingerprints
Fingerprint type        Ligand  Tanimoto Similarity  activity Valid fg
       FeatMFP1      CHEMBL939             0.750000   1000.00        *
           MFP1      CHEMBL939             0.688889   1000.00        *
         RDKit7      CHEMBL939             0.805133   1000.00        *
        Pattern      CHEMBL939             0.822785   1000.00
        AP_bits      CHEMBL939             0.507317   1000.00
        TT_bits      CHEMBL939             0.635135   1000.00        *
            FP2      CHEMBL939             0.782313   1000.00        *
  hybridization      CHEMBL939             0.816471   1000.00        *
   substructure  CHEMBL1996155             0.842105   3162.28
          graph      CHEMBL939             0.777778   1000.00
        pubchem      CHEMBL939             0.925764   1000.00        *
      cdk_maccs      CHEMBL939             0.840580   1000.00        *
   klekota_roth      CHEMBL939             0.600000   1000.00
     *** ki ACTIVITY *** value prediction
     based on 8 locally validated fgps ---> 1000.000
["TT_bits", "cdk_maccs", "MFP1", "pubchem", "FP2", "FeatMFP1", "RDKit7",
"hybridization"]

22 rank
CaM kinase II gamma : Homo sapiens
     score: 6.244 on CHEMBL3829 based on 8 fingerprints
Fingerprint type        Ligand  Tanimoto Similarity  activity Valid fg
       FeatMFP1      CHEMBL939             0.750000   3162.28        *
           MFP1      CHEMBL939             0.688889   3162.28        *
         RDKit7      CHEMBL939             0.805133   3162.28        *
        Pattern  CHEMBL1968705             0.829787   3162.28
        AP_bits      CHEMBL939             0.507317   3162.28
        TT_bits      CHEMBL939             0.635135   3162.28        *
            FP2      CHEMBL939             0.782313   3162.28        *
  hybridization      CHEMBL939             0.816471   3162.28        *
   substructure      CHEMBL939             0.750000   3162.28
          graph      CHEMBL939             0.777778   3162.28
        pubchem      CHEMBL939             0.925764   3162.28        *
      cdk_maccs      CHEMBL939             0.840580   3162.28        *
   klekota_roth      CHEMBL939             0.600000   3162.28
```

```
      *** ki ACTIVITY *** value prediction
      based on 8 locally validated fgps ---> 3162.280
["TT_bits", "cdk_maccs", "MFP1", "pubchem", "FP2", "FeatMFP1", "RDKit7",
"hybridization"]

23 rank
LIM domain kinase 1 : Homo sapiens
      score: 6.244 on CHEMBL3836 based on 8 fingerprints
Fingerprint type         Ligand  Tanimoto Similarity  activity Valid fg
       FeatMFP1      CHEMBL939            0.750000   3981.07         *
           MFP1      CHEMBL939            0.688889   3981.07         *
         RDKit7      CHEMBL939            0.805133   3981.07         *
        Pattern      CHEMBL939            0.822785   3981.07
        AP_bits      CHEMBL939            0.507317   3981.07
        TT_bits      CHEMBL939            0.635135   3981.07         *
            FP2      CHEMBL939            0.782313   3981.07         *
  hybridization      CHEMBL939            0.816471   3981.07         *
   substructure  CHEMBL1996155            0.842105   5011.87
          graph      CHEMBL939            0.777778   3981.07
        pubchem      CHEMBL939            0.925764   3981.07         *
      cdk_maccs      CHEMBL939            0.840580   3981.07         *
   klekota_roth      CHEMBL939            0.600000   3981.07
      *** ki ACTIVITY *** value prediction
      based on 8 locally validated fgps ---> 3981.070
["TT_bits", "cdk_maccs", "MFP1", "pubchem", "FP2", "FeatMFP1", "RDKit7",
"hybridization"]

24 rank
Tyrosine-protein kinase Lyn : Homo sapiens
      score: 6.244 on CHEMBL3905 based on 8 fingerprints
Fingerprint type         Ligand  Tanimoto Similarity  activity Valid fg
       FeatMFP1      CHEMBL939            0.750000    158.49         *
           MFP1      CHEMBL939            0.688889    158.49         *
         RDKit7      CHEMBL939            0.805133    158.49         *
        Pattern  CHEMBL1999749            0.825040     63.10
        AP_bits   CHEMBL215152            0.516266   1000.00
        TT_bits      CHEMBL939            0.635135    158.49         *
            FP2      CHEMBL939            0.782313    158.49         *
  hybridization      CHEMBL939            0.816471    158.49         *
   substructure   CHEMBL230686            0.850000     25.90
          graph      CHEMBL939            0.777778    158.49
        pubchem      CHEMBL939            0.925764    158.49         *
      cdk_maccs      CHEMBL939            0.840580    158.49         *
   klekota_roth      CHEMBL939            0.600000    158.49
      *** ki ACTIVITY *** value prediction
      based on 8 locally validated fgps ---> 158.490
["TT_bits", "cdk_maccs", "MFP1", "pubchem", "FP2", "FeatMFP1", "RDKit7",
"hybridization"]

25 rank
c-Jun N-terminal kinase 2 : Homo sapiens
      score: 6.244 on CHEMBL4179 based on 8 fingerprints
Fingerprint type        Ligand  Tanimoto Similarity  activity Valid fg
       FeatMFP1     CHEMBL939            0.750000   2511.89        *
           MFP1     CHEMBL939            0.688889   2511.89        *
         RDKit7     CHEMBL939            0.805133   2511.89        *
        Pattern     CHEMBL939            0.822785   2511.89
        AP_bits     CHEMBL939            0.507317   2511.89
        TT_bits     CHEMBL939            0.635135   2511.89        *
            FP2     CHEMBL939            0.782313   2511.89        *
```

```
  hybridization     CHEMBL939             0.816471   2511.89        *
   substructure  CHEMBL101311             0.761905   1584.89
          graph     CHEMBL939             0.777778   2511.89
        pubchem     CHEMBL939             0.925764   2511.89        *
      cdk_maccs     CHEMBL939             0.840580   2511.89        *
   klekota_roth     CHEMBL939             0.600000   2511.89
      *** ki ACTIVITY *** value prediction
      based on 8 locally validated fgps ---> 2511.890
["TT_bits", "cdk_maccs", "MFP1", "pubchem", "FP2", "FeatMFP1", "RDKit7",
"hybridization"]

26 rank
Serine/threonine-protein kinase 2 : Homo sapiens
      score: 6.244 on CHEMBL4202 based on 8 fingerprints
Fingerprint type         Ligand  Tanimoto Similarity  activity Valid fg
       FeatMFP1      CHEMBL939             0.750000    398.11        *
           MFP1      CHEMBL939             0.688889    398.11        *
         RDKit7      CHEMBL939             0.805133    398.11        *
        Pattern  CHEMBL1968705             0.829787   6309.57
        AP_bits      CHEMBL939             0.507317    398.11
        TT_bits      CHEMBL939             0.635135    398.11        *
            FP2      CHEMBL939             0.782313    398.11        *
  hybridization      CHEMBL939             0.816471    398.11        *
   substructure  CHEMBL1825138             0.818182     31.62
          graph      CHEMBL939             0.777778    398.11
        pubchem      CHEMBL939             0.925764    398.11        *
      cdk_maccs      CHEMBL939             0.840580    398.11        *
   klekota_roth      CHEMBL939             0.600000    398.11
      *** ki ACTIVITY *** value prediction
      based on 8 locally validated fgps ---> 398.110
["TT_bits", "cdk_maccs", "MFP1", "pubchem", "FP2", "FeatMFP1", "RDKit7",
"hybridization"]

27 rank
MAP kinase signal-integrating kinase 2 : Homo sapiens
      score: 6.244 on CHEMBL4204 based on 8 fingerprints
Fingerprint type         Ligand  Tanimoto Similarity  activity Valid fg
       FeatMFP1      CHEMBL939             0.750000    398.11        *
           MFP1      CHEMBL939             0.688889    398.11        *
         RDKit7      CHEMBL939             0.805133    398.11        *
        Pattern  CHEMBL1968705             0.829787   7943.28
        AP_bits      CHEMBL939             0.507317    398.11
        TT_bits      CHEMBL939             0.635135    398.11        *
            FP2      CHEMBL939             0.782313    398.11        *
  hybridization      CHEMBL939             0.816471    398.11        *
   substructure      CHEMBL939             0.750000    398.11
          graph      CHEMBL939             0.777778    398.11
        pubchem      CHEMBL939             0.925764    398.11        *
      cdk_maccs      CHEMBL939             0.840580    398.11        *
   klekota_roth      CHEMBL939             0.600000    398.11
      *** ki ACTIVITY *** value prediction
      based on 8 locally validated fgps ---> 398.110
["TT_bits", "cdk_maccs", "MFP1", "pubchem", "FP2", "FeatMFP1", "RDKit7",
"hybridization"]

28 rank
Tyrosine-protein kinase FRK : Homo sapiens
      score: 6.244 on CHEMBL4223 based on 8 fingerprints
Fingerprint type         Ligand  Tanimoto Similarity  activity Valid fg
       FeatMFP1      CHEMBL939             0.750000    251.19        *
```

```
         MFP1      CHEMBL939              0.688889    251.19         *
        RDKit7     CHEMBL939              0.805133    251.19         *
       Pattern  CHEMBL1973961             0.826014   1258.93
       AP_bits     CHEMBL939              0.507317    251.19
       TT_bits     CHEMBL939              0.635135    251.19         *
           FP2     CHEMBL939              0.782313    251.19         *
 hybridization     CHEMBL939              0.816471    251.19         *
  substructure  CHEMBL1825138             0.818182    199.53
         graph     CHEMBL939              0.777778    251.19
       pubchem     CHEMBL939              0.925764    251.19         *
     cdk_maccs     CHEMBL939              0.840580    251.19         *
  klekota_roth     CHEMBL939              0.600000    251.19
      *** ki ACTIVITY *** value prediction
      based on 8 locally validated fgps ---> 251.190
["TT_bits", "cdk_maccs", "MFP1", "pubchem", "FP2", "FeatMFP1", "RDKit7",
"hybridization"]

29 rank
Serine/threonine-protein kinase D2 : Homo sapiens
      score: 6.244 on CHEMBL4900 based on 8 fingerprints
Fingerprint type         Ligand  Tanimoto Similarity  activity Valid fg
      FeatMFP1     CHEMBL939              0.750000   2511.89         *
          MFP1     CHEMBL939              0.688889   2511.89         *
        RDKit7     CHEMBL939              0.805133   2511.89         *
       Pattern  CHEMBL1968705             0.829787   7943.28
       AP_bits     CHEMBL939              0.507317   2511.89
       TT_bits     CHEMBL939              0.635135   2511.89         *
           FP2     CHEMBL939              0.782313   2511.89         *
 hybridization     CHEMBL939              0.816471   2511.89         *
  substructure  CHEMBL1996155             0.842105    398.11
         graph     CHEMBL939              0.777778   2511.89
       pubchem     CHEMBL939              0.925764   2511.89         *
     cdk_maccs     CHEMBL939              0.840580   2511.89         *
  klekota_roth     CHEMBL939              0.600000   2511.89
      *** ki ACTIVITY *** value prediction
      based on 8 locally validated fgps ---> 2511.890
["TT_bits", "cdk_maccs", "MFP1", "pubchem", "FP2", "FeatMFP1", "RDKit7",
"hybridization"]

30 rank
Serine/threonine-protein kinase PIM3 : Homo sapiens
      score: 6.244 on CHEMBL5407 based on 8 fingerprints
Fingerprint type         Ligand  Tanimoto Similarity  activity Valid fg
      FeatMFP1     CHEMBL939              0.750000   1258.93         *
          MFP1     CHEMBL939              0.688889   1258.93         *
        RDKit7     CHEMBL939              0.805133   1258.93         *
       Pattern  CHEMBL1968705             0.829787   3981.07
       AP_bits     CHEMBL939              0.507317   1258.93
       TT_bits     CHEMBL939              0.635135   1258.93         *
           FP2     CHEMBL939              0.782313   1258.93         *
 hybridization     CHEMBL939              0.816471   1258.93         *
  substructure  CHEMBL3634777             0.850000      0.11
         graph     CHEMBL939              0.777778   1258.93
       pubchem     CHEMBL939              0.925764   1258.93         *
     cdk_maccs     CHEMBL939              0.840580   1258.93         *
  klekota_roth     CHEMBL939              0.600000   1258.93
      *** ki ACTIVITY *** value prediction
      based on 8 locally validated fgps ---> 1258.930
["TT_bits", "cdk_maccs", "MFP1", "pubchem", "FP2", "FeatMFP1", "RDKit7",
"hybridization"]
```

31 rank
Misshapen-like kinase 1 : Homo sapiens
score: 6.244 on CHEMBL5518 based on 8 fingerprints

Fingerprint type	Ligand	Tanimoto Similarity	activity	Valid fg
FeatMFP1	CHEMBL939	0.750000	5011.87	*
MFP1	CHEMBL939	0.688889	5011.87	*
RDKit7	CHEMBL939	0.805133	5011.87	*
Pattern	CHEMBL939	0.822785	5011.87	
AP_bits	CHEMBL939	0.507317	5011.87	
TT_bits	CHEMBL939	0.635135	5011.87	*
FP2	CHEMBL939	0.782313	5011.87	*
hybridization	CHEMBL939	0.816471	5011.87	*
substructure	CHEMBL95692	0.850000	3981.07	
graph	CHEMBL939	0.777778	5011.87	
pubchem	CHEMBL939	0.925764	5011.87	*
cdk_maccs	CHEMBL939	0.840580	5011.87	*
klekota_roth	CHEMBL939	0.600000	5011.87	

*** ki ACTIVITY *** value prediction
based on 8 locally validated fgps ---> 5011.870
["TT_bits", "cdk_maccs", "MFP1", "pubchem", "FP2", "FeatMFP1", "RDKit7", "hybridization"]

32 rank
BR serine/threonine-protein kinase 1 : Homo sapiens
score: 6.244 on CHEMBL5650 based on 8 fingerprints

Fingerprint type	Ligand	Tanimoto Similarity	activity	Valid fg
FeatMFP1	CHEMBL939	0.750000	3162.28	*
MFP1	CHEMBL939	0.688889	3162.28	*
RDKit7	CHEMBL939	0.805133	3162.28	*
Pattern	CHEMBL939	0.822785	3162.28	
AP_bits	CHEMBL939	0.507317	3162.28	
TT_bits	CHEMBL939	0.635135	3162.28	*
FP2	CHEMBL939	0.782313	3162.28	*
hybridization	CHEMBL939	0.816471	3162.28	*
substructure	CHEMBL939	0.750000	3162.28	
graph	CHEMBL939	0.777778	3162.28	
pubchem	CHEMBL939	0.925764	3162.28	*
cdk_maccs	CHEMBL939	0.840580	3162.28	*
klekota_roth	CHEMBL939	0.600000	3162.28	

*** ki ACTIVITY *** value prediction
based on 8 locally validated fgps ---> 3162.280
["TT_bits", "cdk_maccs", "MFP1", "pubchem", "FP2", "FeatMFP1", "RDKit7", "hybridization"]

33 rank
Serine/threonine-protein kinase SIK2 : Homo sapiens
score: 6.244 on CHEMBL5699 based on 8 fingerprints

Fingerprint type	Ligand	Tanimoto Similarity	activity	Valid fg
FeatMFP1	CHEMBL939	0.750000	316.23	*
MFP1	CHEMBL939	0.688889	316.23	*
RDKit7	CHEMBL939	0.805133	316.23	*
Pattern	CHEMBL939	0.822785	316.23	
AP_bits	CHEMBL939	0.507317	316.23	
TT_bits	CHEMBL939	0.635135	316.23	*
FP2	CHEMBL939	0.782313	316.23	*
hybridization	CHEMBL939	0.816471	316.23	*
substructure	CHEMBL939	0.750000	316.23	
graph	CHEMBL939	0.777778	316.23	
pubchem	CHEMBL939	0.925764	316.23	*

cdk_maccs	CHEMBL939	0.840580	316.23	*
klekota_roth	CHEMBL939	0.600000	316.23	

*** ki ACTIVITY *** value prediction
based on 8 locally validated fgps ---> 316.230
["TT_bits", "cdk_maccs", "MFP1", "pubchem", "FP2", "FeatMFP1", "RDKit7", "hybridization"]

34 rank
Serine/threonine-protein kinase Aurora-B : Homo sapiens
score: 3.640 on CHEMBL2185 based on 5 fingerprints

Fingerprint type	Ligand	Tanimoto Similarity	activity	Valid fg
FeatMFP1	CHEMBL24828	0.676471	2511.89	
MFP1	CHEMBL202721	0.540000	79.43	
RDKit7	CHEMBL202721	0.717813	79.43	
Pattern	CHEMBL386310	0.875415	1.00	*
AP_bits	CHEMBL386310	0.608054	1.00	*
TT_bits	CHEMBL217804	0.609756	2.00	*
FP2	CHEMBL202721	0.619048	79.43	
hybridization	CHEMBL554	0.701826	1995.26	*
substructure	CHEMBL1825138	0.818182	39.81	
graph	CHEMBL24828	0.668293	2511.89	
pubchem	CHEMBL24828	0.848739	2511.89	
cdk_maccs	CHEMBL24828	0.845070	2511.89	*
klekota_roth	CHEMBL491473	0.585366	1258.93	

*** ki ACTIVITY *** value prediction
based on 5 locally validated fgps ---> 902.132
["Pattern", "hybridization", "TT_bits", "cdk_maccs", "AP_bits"]

35 rank
Platelet-derived growth factor receptor alpha : Homo sapiens
score: 3.105 on CHEMBL2007 based on 4 fingerprints

Fingerprint type	Ligand	Tanimoto Similarity	activity	Valid fg
FeatMFP1	CHEMBL24828	0.676471	158.49	
MFP1	CHEMBL202721	0.540000	398.11	
RDKit7	CHEMBL202721	0.717813	398.11	
Pattern	CHEMBL124660	0.861301	63.10	*
AP_bits	CHEMBL202721	0.547865	398.11	*
TT_bits	CHEMBL202721	0.529412	398.11	
FP2	CHEMBL202721	0.619048	398.11	
hybridization	CHEMBL202721	0.685714	398.11	
substructure	CHEMBL24828	0.809524	158.49	
graph	CHEMBL24828	0.668293	158.49	
pubchem	CHEMBL124660	0.851064	63.10	*
cdk_maccs	CHEMBL24828	0.845070	158.49	*
klekota_roth	CHEMBL491473	0.585366	10.00	

*** ki ACTIVITY *** value prediction
based on 4 locally validated fgps ---> 170.700
["Pattern", "cdk_maccs", "pubchem", "AP_bits"]

MuSSel Prediction IC_{50}:

```
1 rank
Epidermal growth factor receptor erbB1 : Homo sapiens
      score: 10.901 on CHEMBL203 based on 13 fingerprints
Fingerprint type          Ligand  Tanimoto Similarity  activity Valid fg
       FeatMFP1    CHEMBL56936             0.812500      2.00        *
           MFP1      CHEMBL939             0.688889      0.10        *
         RDKit7   CHEMBL598799             0.915222      9.08        *
        Pattern   CHEMBL598799             0.926786      9.08        *
        AP_bits   CHEMBL598799             0.675595      9.08        *
        TT_bits   CHEMBL598799             0.760000      9.08        *
            FP2  CHEMBL2334003             0.855072     13.60        *
  hybridization   CHEMBL598799             0.841871      9.08        *
   substructure   CHEMBL247709             0.950000     48.00        *
          graph  CHEMBL3355883             0.911917     38.30        *
        pubchem  CHEMBL2334003             0.950893     13.60        *
      cdk_maccs   CHEMBL291514             0.911765     79.00        *
   klekota_roth   CHEMBL247709             0.700000     48.00        *
      *** ic50 ACTIVITY *** value prediction
      based on 13 locally validated fgps ---> 58.254
["TT_bits", "substructure", "cdk_maccs", "Pattern", "FP2", "MFP1",
"klekota_roth", "pubchem", "graph", "AP_bits", "FeatMFP1", "RDKit7",
"hybridization"]

2 rank
Receptor protein-tyrosine kinase erbB-2 : Homo sapiens
      score: 10.719 on CHEMBL1824 based on 13 fingerprints
Fingerprint type          Ligand  Tanimoto Similarity  activity Valid fg
       FeatMFP1  CHEMBL3814812             0.777778      12.1        *
           MFP1      CHEMBL939             0.688889     240.0        *
         RDKit7   CHEMBL598799             0.915222      10.1        *
        Pattern   CHEMBL598799             0.926786      10.1        *
        AP_bits   CHEMBL598799             0.675595      10.1        *
        TT_bits   CHEMBL598799             0.760000      10.1        *
            FP2   CHEMBL197640             0.836735       2.0        *
  hybridization   CHEMBL598799             0.841871      10.1        *
   substructure   CHEMBL247709             0.950000      41.0        *
          graph  CHEMBL2408050             0.835897     264.0        *
        pubchem      CHEMBL939             0.925764     240.0        *
      cdk_maccs   CHEMBL197640             0.884058       2.0        *
   klekota_roth   CHEMBL247709             0.700000      41.0        *
      *** ic50 ACTIVITY *** value prediction
      based on 13 locally validated fgps ---> 125.194
["TT_bits", "substructure", "cdk_maccs", "Pattern", "FP2", "MFP1",
"klekota_roth", "pubchem", "graph", "AP_bits", "FeatMFP1", "RDKit7",
"hybridization"]

3 rank
Vascular endothelial growth factor receptor 2 : Homo sapiens
      score: 10.262 on CHEMBL279 based on 13 fingerprints
Fingerprint type          Ligand  Tanimoto Similarity  activity Valid fg
       FeatMFP1  CHEMBL1202477             0.787879       9.0        *
           MFP1      CHEMBL939             0.688889    1900.0        *
         RDKit7      CHEMBL939             0.805133    1900.0        *
        Pattern  CHEMBL3758749             0.898067    1810.0        *
        AP_bits  CHEMBL3133754             0.603198    8568.0        *
        TT_bits   CHEMBL169797             0.643836    1110.0        *
            FP2  CHEMBL1744347             0.787671      20.0        *
  hybridization      CHEMBL939             0.816471    1900.0        *
   substructure   CHEMBL147180             0.947368      40.0        *
          graph  CHEMBL3706823             0.782609       1.0        *
```

```
        pubchem  CHEMBL1744347            0.934211      20.0        *
      cdk_maccs  CHEMBL1744347            0.900000      20.0        *
   klekota_roth  CHEMBL2448067            0.666667      30.0        *
      *** ic50 ACTIVITY *** value prediction
      based on 13 locally validated fgps ---> 1332.325
["TT_bits", "substructure", "cdk_maccs", "Pattern", "FP2", "MFP1",
"klekota_roth", "pubchem", "graph", "AP_bits", "FeatMFP1", "RDKit7",
"hybridization"]

4 rank
Tyrosine-protein kinase SRC : Homo sapiens
      score: 10.050 on CHEMBL267 based on 13 fingerprints
Fingerprint type        Ligand  Tanimoto Similarity  activity Valid fg
       FeatMFP1  CHEMBL128158            0.764706       1.1        *
           MFP1     CHEMBL939            0.688889    1100.0        *
         RDKit7     CHEMBL939            0.805133    1100.0        *
        Pattern  CHEMBL426148            0.879581     600.0        *
        AP_bits  CHEMBL201307            0.572005    3000.0        *
        TT_bits  CHEMBL169797            0.643836      37.0        *
            FP2     CHEMBL939            0.782313    1100.0        *
  hybridization     CHEMBL939            0.816471    1100.0        *
   substructure  CHEMBL217977            0.894737     540.0        *
          graph     CHEMBL939            0.777778    1100.0        *
        pubchem     CHEMBL939            0.925764    1100.0        *
      cdk_maccs  CHEMBL128158            0.898551       1.1        *
   klekota_roth     CHEMBL939            0.600000    1100.0        *
      *** ic50 ACTIVITY *** value prediction
      based on 13 locally validated fgps ---> 701.377
["TT_bits", "substructure", "cdk_maccs", "Pattern", "FP2", "MFP1",
"klekota_roth", "pubchem", "graph", "AP_bits", "FeatMFP1", "RDKit7",
"hybridization"]

5 rank
Tyrosine-protein kinase receptor RET : Homo sapiens
      score: 10.028 on CHEMBL2041 based on 13 fingerprints
Fingerprint type         Ligand  Tanimoto Similarity  activity Valid fg
       FeatMFP1  CHEMBL3797261            0.800000       5.0        *
           MFP1      CHEMBL939            0.688889    1700.0        *
         RDKit7      CHEMBL939            0.805133    1700.0        *
        Pattern  CHEMBL3800348            0.855615      29.0        *
        AP_bits      CHEMBL939            0.507317    1700.0        *
        TT_bits      CHEMBL939            0.635135    1700.0        *
            FP2      CHEMBL939            0.782313    1700.0        *
  hybridization      CHEMBL939            0.816471    1700.0        *
   substructure  CHEMBL3797261            0.857143       5.0        *
          graph      CHEMBL939            0.777778    1700.0        *
        pubchem      CHEMBL939            0.925764    1700.0        *
      cdk_maccs  CHEMBL3797261            0.914286       5.0        *
   klekota_roth  CHEMBL3799971            0.662338      11.0        *
      *** ic50 ACTIVITY *** value prediction
      based on 13 locally validated fgps ---> 740.419
["TT_bits", "substructure", "cdk_maccs", "Pattern", "FP2", "MFP1",
"klekota_roth", "pubchem", "graph", "AP_bits", "FeatMFP1", "RDKit7",
"hybridization"]

6 rank
Receptor protein-tyrosine kinase erbB-4 : Homo sapiens
      score: 9.883 on CHEMBL3009 based on 13 fingerprints
Fingerprint type         Ligand  Tanimoto Similarity  activity Valid fg
       FeatMFP1      CHEMBL939            0.750000     476.0        *
```

```
          MFP1      CHEMBL939             0.688889     476.0        *
        RDKit7      CHEMBL939             0.805133     476.0        *
       Pattern  CHEMBL3680375             0.891753      61.5        *
       AP_bits  CHEMBL1645462             0.519411     190.0        *
       TT_bits      CHEMBL939             0.635135     476.0        *
           FP2      CHEMBL939             0.782313     476.0        *
 hybridization      CHEMBL939             0.816471     476.0        *
  substructure  CHEMBL3828092             0.850000    1603.0        *
         graph      CHEMBL939             0.777778     476.0        *
       pubchem      CHEMBL939             0.925764     476.0        *
     cdk_maccs      CHEMBL939             0.840580     476.0        *
  klekota_roth      CHEMBL939             0.600000     476.0        *
     *** ic50 ACTIVITY *** value prediction
     based on 12 locally validated fgps ---> 352.929
["TT_bits", "cdk_maccs", "Pattern", "FP2", "MFP1", "klekota_roth",
"pubchem", "graph", "AP_bits", "FeatMFP1", "RDKit7", "hybridization"]

7 rank
Vascular endothelial growth factor receptor 1 : Homo sapiens
     score: 9.188 on CHEMBL1868 based on 12 fingerprints
Fingerprint type         Ligand  Tanimoto Similarity  activity Valid fg
      FeatMFP1  CHEMBL1202477             0.787879     550.0        *
          MFP1    CHEMBL24137             0.617021     200.0        *
        RDKit7  CHEMBL1744347             0.792329     600.0        *
       Pattern  CHEMBL2448066             0.872302      20.0        *
       AP_bits   CHEMBL453737             0.538462      45.0        *
       TT_bits  CHEMBL2448067             0.586667     200.0        *
           FP2  CHEMBL1744347             0.787671     600.0        *
 hybridization  CHEMBL1744347             0.757506     600.0        *
  substructure   CHEMBL147180             0.947368     300.0        *
         graph  CHEMBL1744347             0.766497     600.0
       pubchem  CHEMBL1744347             0.934211     600.0        *
     cdk_maccs  CHEMBL1744347             0.900000     600.0        *
  klekota_roth  CHEMBL2448067             0.666667     200.0        *
     *** ic50 ACTIVITY *** value prediction
     based on 12 locally validated fgps ---> 426.218
["TT_bits", "substructure", "cdk_maccs", "Pattern", "FP2", "MFP1",
"klekota_roth", "pubchem", "AP_bits", "FeatMFP1", "RDKit7",
"hybridization"]

8 rank
Fibroblast growth factor receptor 1 : Homo sapiens
     score: 9.165 on CHEMBL3650 based on 12 fingerprints
Fingerprint type         Ligand  Tanimoto Similarity  activity Valid fg
      FeatMFP1  CHEMBL1202477             0.787879    2000.0        *
          MFP1    CHEMBL24137             0.617021    6700.0        *
        RDKit7  CHEMBL1744347             0.792329    2100.0        *
       Pattern  CHEMBL2448066             0.872302     900.0        *
       AP_bits  CHEMBL3691340             0.515843      11.5        *
       TT_bits  CHEMBL2419762             0.586667      63.0        *
           FP2  CHEMBL1744347             0.787671    2100.0        *
 hybridization  CHEMBL1744347             0.757506    2100.0        *
  substructure   CHEMBL147180             0.947368    9400.0        *
         graph  CHEMBL1744347             0.766497    2100.0
       pubchem  CHEMBL1744347             0.934211    2100.0        *
     cdk_maccs  CHEMBL1744347             0.900000    2100.0        *
  klekota_roth  CHEMBL2448067             0.666667    4200.0        *
     *** ic50 ACTIVITY *** value prediction
     based on 12 locally validated fgps ---> 4262.451
```

```
["TT_bits", "substructure", "cdk_maccs", "Pattern", "FP2", "MFP1",
"klekota_roth", "pubchem", "AP_bits", "FeatMFP1", "RDKit7",
"hybridization"]

9 rank
Serine/threonine-protein kinase B-raf : Homo sapiens
      score: 9.154 on CHEMBL5145 based on 12 fingerprints
Fingerprint type         Ligand  Tanimoto Similarity  activity Valid fg
       FeatMFP1      CHEMBL939             0.750000   30000.0        *
           MFP1      CHEMBL939             0.688889   30000.0        *
         RDKit7      CHEMBL939             0.805133   30000.0        *
        Pattern  CHEMBL3133753             0.912892      31.0        *
        AP_bits  CHEMBL3133753             0.618911      31.0        *
        TT_bits      CHEMBL939             0.635135   30000.0        *
            FP2      CHEMBL939             0.782313   30000.0        *
  hybridization      CHEMBL939             0.816471   30000.0        *
   substructure  CHEMBL1079785             0.809524       8.7
          graph      CHEMBL939             0.777778   30000.0        *
        pubchem      CHEMBL939             0.925764   30000.0        *
      cdk_maccs      CHEMBL939             0.840580   30000.0        *
   klekota_roth      CHEMBL939             0.600000   30000.0        *
      *** ic50 ACTIVITY *** value prediction
      based on 12 locally validated fgps ---> 16597.534
["TT_bits", "cdk_maccs", "Pattern", "FP2", "MFP1", "klekota_roth",
"pubchem", "graph", "AP_bits", "FeatMFP1", "RDKit7", "hybridization"]

10 rank
Ephrin type-B receptor 4 : Homo sapiens
      score: 9.036 on CHEMBL5147 based on 12 fingerprints
Fingerprint type         Ligand  Tanimoto Similarity  activity Valid fg
       FeatMFP1      CHEMBL939             0.750000    1000.0        *
           MFP1      CHEMBL939             0.688889    1000.0        *
         RDKit7      CHEMBL939             0.805133    1000.0        *
        Pattern   CHEMBL288441             0.823430       5.5
        AP_bits      CHEMBL939             0.507317    1000.0        *
        TT_bits      CHEMBL939             0.635135    1000.0        *
            FP2      CHEMBL939             0.782313    1000.0        *
  hybridization      CHEMBL939             0.816471    1000.0        *
   substructure  CHEMBL3544983             0.900000      50.0        *
          graph      CHEMBL939             0.777778    1000.0        *
        pubchem      CHEMBL939             0.925764    1000.0        *
      cdk_maccs   CHEMBL288441             0.847222       5.5        *
   klekota_roth      CHEMBL939             0.600000    1000.0        *
      *** ic50 ACTIVITY *** value prediction
      based on 12 locally validated fgps ---> 704.900
["TT_bits", "substructure", "cdk_maccs", "AP_bits", "MFP1",
"klekota_roth", "pubchem", "graph", "FP2", "FeatMFP1", "RDKit7",
"hybridization"]

11 rank
Tyrosine-protein kinase ABL : Homo sapiens
      score: 9.022 on CHEMBL1862 based on 12 fingerprints
Fingerprint type         Ligand  Tanimoto Similarity  activity Valid fg
       FeatMFP1      CHEMBL939             0.750000    1200.0        *
           MFP1      CHEMBL939             0.688889    1200.0        *
         RDKit7      CHEMBL939             0.805133    1200.0        *
        Pattern  CHEMBL1630109             0.886010    5300.0        *
        AP_bits      CHEMBL939             0.507317    1200.0        *
        TT_bits      CHEMBL939             0.635135    1200.0        *
            FP2      CHEMBL939             0.782313    1200.0        *
```

```
  hybridization      CHEMBL939             0.816471    1200.0        *
   substructure   CHEMBL601719             0.818182      24.0
          graph      CHEMBL939             0.777778    1200.0        *
        pubchem      CHEMBL939             0.925764    1200.0        *
      cdk_maccs   CHEMBL288441             0.847222       0.5        *
   klekota_roth      CHEMBL939             0.600000    1200.0        *
      *** ic50 ACTIVITY *** value prediction
      based on 12 locally validated fgps ---> 1769.360
["TT_bits", "cdk_maccs", "Pattern", "FP2", "MFP1", "klekota_roth",
"pubchem", "graph", "AP_bits", "FeatMFP1", "RDKit7", "hybridization"]

12 rank
Tyrosine kinase non-receptor protein 2 : Homo sapiens
      score: 8.986 on CHEMBL4599 based on 12 fingerprints
Fingerprint type         Ligand  Tanimoto Similarity  activity Valid fg
       FeatMFP1      CHEMBL939             0.750000    2300.0        *
           MFP1      CHEMBL939             0.688889    2300.0        *
         RDKit7      CHEMBL939             0.805133    2300.0        *
        Pattern  CHEMBL1983268             0.824959      70.0
        AP_bits      CHEMBL939             0.507317    2300.0        *
        TT_bits      CHEMBL939             0.635135    2300.0        *
            FP2      CHEMBL939             0.782313    2300.0        *
  hybridization      CHEMBL939             0.816471    2300.0        *
   substructure   CHEMBL247468             0.850000      98.0        *
          graph      CHEMBL939             0.777778    2300.0        *
        pubchem      CHEMBL939             0.925764    2300.0        *
      cdk_maccs   CHEMBL288441             0.847222       2.7        *
   klekota_roth      CHEMBL939             0.600000    2300.0        *
      *** ic50 ACTIVITY *** value prediction
      based on 11 locally validated fgps ---> 1919.949
["TT_bits", "cdk_maccs", "AP_bits", "MFP1", "klekota_roth", "pubchem",
"graph", "FP2", "FeatMFP1", "RDKit7", "hybridization"]

13 rank
ATP-binding cassette sub-family G member 2 : Homo sapiens
      score: 8.986 on CHEMBL5393 based on 12 fingerprints
Fingerprint type         Ligand  Tanimoto Similarity  activity Valid fg
       FeatMFP1      CHEMBL939             0.750000     400.0        *
           MFP1      CHEMBL939             0.688889     400.0        *
         RDKit7      CHEMBL939             0.805133     400.0        *
        Pattern  CHEMBL3809832             0.856373    1710.0        *
        AP_bits      CHEMBL939             0.507317     400.0        *
        TT_bits      CHEMBL939             0.635135     400.0        *
            FP2      CHEMBL939             0.782313     400.0        *
  hybridization      CHEMBL939             0.816471     400.0        *
   substructure      CHEMBL939             0.750000     400.0
          graph      CHEMBL939             0.777778     400.0        *
        pubchem      CHEMBL939             0.925764     400.0        *
      cdk_maccs      CHEMBL939             0.840580     400.0        *
   klekota_roth      CHEMBL939             0.600000     400.0        *
      *** ic50 ACTIVITY *** value prediction
      based on 12 locally validated fgps ---> 4402.171
["TT_bits", "cdk_maccs", "Pattern", "FP2", "MFP1", "klekota_roth",
"pubchem", "graph", "AP_bits", "FeatMFP1", "RDKit7", "hybridization"]

14 rank
Platelet-derived growth factor receptor beta : Homo sapiens
      score: 8.899 on CHEMBL1913 based on 12 fingerprints
Fingerprint type         Ligand  Tanimoto Similarity  activity Valid fg
       FeatMFP1   CHEMBL593274             0.742857    3600.0        *
```

```
           MFP1   CHEMBL593274            0.588235    3600.0        *
         RDKit7   CHEMBL301018            0.750727    3000.0        *
        Pattern  CHEMBL1630109            0.886010    2500.0        *
        AP_bits   CHEMBL604893            0.609078    7700.0        *
        TT_bits  CHEMBL1630109            0.587500    2500.0        *
            FP2   CHEMBL301018            0.710145    3000.0        *
  hybridization  CHEMBL1630113            0.747788    3900.0        *
   substructure   CHEMBL593909            0.900000    9200.0        *
          graph  CHEMBL1630113            0.734694    3900.0
        pubchem   CHEMBL301018            0.910714    3000.0        *
      cdk_maccs    CHEMBL24828            0.845070     477.0        *
   klekota_roth   CHEMBL593909            0.620690    9200.0        *
      *** ic50 ACTIVITY *** value prediction
      based on 12 locally validated fgps ---> 4335.226
["TT_bits", "substructure", "cdk_maccs", "Pattern", "FP2", "MFP1",
"klekota_roth", "pubchem", "AP_bits", "FeatMFP1", "RDKit7",
"hybridization"]

15 rank
Serine/threonine-protein kinase Aurora-A : Homo sapiens
      score: 8.875 on CHEMBL4722 based on 12 fingerprints
Fingerprint type        Ligand  Tanimoto Similarity  activity Valid fg
       FeatMFP1  CHEMBL593274            0.742857     27.00        *
           MFP1  CHEMBL593274            0.588235     27.00        *
         RDKit7  CHEMBL301018            0.750727   6500.00        *
        Pattern  CHEMBL382667            0.880750      0.15        *
        AP_bits  CHEMBL604893            0.609078    690.00        *
        TT_bits  CHEMBL217804            0.609756    220.00        *
            FP2  CHEMBL301018            0.710145   6500.00        *
  hybridization  CHEMBL604893            0.700787    690.00        *
   substructure  CHEMBL593909            0.900000     85.00        *
          graph  CHEMBL301018            0.713514   6500.00
        pubchem  CHEMBL301018            0.910714   6500.00        *
      cdk_maccs  CHEMBL203945            0.851351      0.80        *
   klekota_roth  CHEMBL593909            0.620690     85.00        *
      *** ic50 ACTIVITY *** value prediction
      based on 12 locally validated fgps ---> 4089.889
["TT_bits", "substructure", "cdk_maccs", "Pattern", "FP2", "MFP1",
"klekota_roth", "pubchem", "AP_bits", "FeatMFP1", "RDKit7",
"hybridization"]

16 rank
Serine/threonine-protein kinase Aurora-B : Homo sapiens
      score: 8.835 on CHEMBL2185 based on 12 fingerprints
Fingerprint type         Ligand  Tanimoto Similarity  activity Valid fg
       FeatMFP1   CHEMBL593274            0.742857      78.0        *
           MFP1   CHEMBL593274            0.588235      78.0        *
         RDKit7   CHEMBL301018            0.750727    7400.0        *
        Pattern  CHEMBL3685239            0.873684       6.5        *
        AP_bits   CHEMBL604893            0.609078    4800.0        *
        TT_bits   CHEMBL217804            0.609756       2.0        *
            FP2   CHEMBL301018            0.710145    7400.0        *
  hybridization   CHEMBL604893            0.700787    4800.0        *
   substructure   CHEMBL593909            0.900000      90.0        *
          graph   CHEMBL301018            0.713514    7400.0
        pubchem   CHEMBL301018            0.910714    7400.0        *
      cdk_maccs   CHEMBL593909            0.818182      90.0        *
   klekota_roth   CHEMBL593909            0.620690      90.0        *
      *** ic50 ACTIVITY *** value prediction
      based on 12 locally validated fgps ---> 1930.037
```

```
["TT_bits", "substructure", "cdk_maccs", "Pattern", "FP2", "MFP1",
"klekota_roth", "pubchem", "AP_bits", "FeatMFP1", "RDKit7",
"hybridization"]

17 rank
Vascular endothelial growth factor receptor 3 : Homo sapiens
      score: 8.714 on CHEMBL1955 based on 12 fingerprints
Fingerprint type          Ligand  Tanimoto Similarity  activity Valid fg
       FeatMFP1   CHEMBL593274             0.742857     470.0        *
           MFP1   CHEMBL593274             0.588235     470.0        *
         RDKit7   CHEMBL301018             0.750727    2400.0        *
        Pattern  CHEMBL1934327             0.851590       9.6        *
        AP_bits   CHEMBL593274             0.569602     470.0        *
        TT_bits   CHEMBL301018             0.541667    2400.0        *
            FP2   CHEMBL301018             0.710145    2400.0        *
  hybridization   CHEMBL593274             0.682879     470.0        *
   substructure   CHEMBL593909             0.900000    3000.0        *
          graph   CHEMBL301018             0.713514    2400.0
        pubchem   CHEMBL301018             0.910714    2400.0        *
      cdk_maccs    CHEMBL24828             0.845070     233.0        *
   klekota_roth   CHEMBL593909             0.620690    3000.0        *
      *** ic50 ACTIVITY *** value prediction
      based on 12 locally validated fgps ---> 1711.813
["TT_bits", "substructure", "cdk_maccs", "Pattern", "FP2", "MFP1",
"klekota_roth", "pubchem", "AP_bits", "FeatMFP1", "RDKit7",
"hybridization"]

18 rank
Tyrosine-protein kinase HCK : Homo sapiens
      score: 8.136 on CHEMBL3234 based on 11 fingerprints
Fingerprint type         Ligand  Tanimoto Similarity  activity Valid fg
       FeatMFP1     CHEMBL939             0.750000     110.0        *
           MFP1     CHEMBL939             0.688889     110.0        *
         RDKit7     CHEMBL939             0.805133     110.0        *
        Pattern  CHEMBL288441             0.823430       3.2
        AP_bits     CHEMBL939             0.507317     110.0        *
        TT_bits     CHEMBL939             0.635135     110.0        *
            FP2     CHEMBL939             0.782313     110.0        *
  hybridization     CHEMBL939             0.816471     110.0        *
   substructure  CHEMBL288441             0.772727       3.2
          graph     CHEMBL939             0.777778     110.0        *
        pubchem     CHEMBL939             0.925764     110.0        *
      cdk_maccs  CHEMBL288441             0.847222       3.2        *
   klekota_roth     CHEMBL939             0.600000     110.0        *
      *** ic50 ACTIVITY *** value prediction
      based on 11 locally validated fgps ---> 180.410
["TT_bits", "cdk_maccs", "AP_bits", "MFP1", "klekota_roth", "pubchem",
"graph", "FP2", "FeatMFP1", "RDKit7", "hybridization"]

19 rank
Cruzipain : Trypanosoma cruzi
      score: 8.129 on CHEMBL3563 based on 11 fingerprints
Fingerprint type      Ligand  Tanimoto Similarity  activity Valid fg
       FeatMFP1  CHEMBL939             0.750000   88000.0        *
           MFP1  CHEMBL939             0.688889   88000.0        *
         RDKit7  CHEMBL939             0.805133   88000.0        *
        Pattern  CHEMBL939             0.822785   88000.0
        AP_bits  CHEMBL939             0.507317   88000.0        *
        TT_bits  CHEMBL939             0.635135   88000.0        *
            FP2  CHEMBL939             0.782313   88000.0        *
```

```
  hybridization  CHEMBL939            0.816471   88000.0        *
   substructure  CHEMBL939            0.750000   88000.0
          graph  CHEMBL939            0.777778   88000.0        *
        pubchem  CHEMBL939            0.925764   88000.0        *
      cdk_maccs  CHEMBL939            0.840580   88000.0        *
   klekota_roth  CHEMBL939            0.600000   88000.0        *
      *** ic50 ACTIVITY *** value prediction
      based on 11 locally validated fgps ---> 88000.000
["TT_bits", "cdk_maccs", "AP_bits", "MFP1", "klekota_roth", "pubchem",
"graph", "FP2", "FeatMFP1", "RDKit7", "hybridization"]

20 rank
Tyrosine-protein kinase BRK : Homo sapiens
      score: 8.129 on CHEMBL4601 based on 11 fingerprints
Fingerprint type         Ligand  Tanimoto Similarity  activity Valid fg
       FeatMFP1      CHEMBL939            0.750000     980.0        *
           MFP1      CHEMBL939            0.688889     980.0        *
         RDKit7      CHEMBL939            0.805133     980.0        *
        Pattern  CHEMBL1983268            0.824959     195.0
        AP_bits      CHEMBL939            0.507317     980.0        *
        TT_bits      CHEMBL939            0.635135     980.0        *
            FP2      CHEMBL939            0.782313     980.0        *
  hybridization      CHEMBL939            0.816471     980.0        *
   substructure      CHEMBL939            0.750000     980.0
          graph      CHEMBL939            0.777778     980.0        *
        pubchem      CHEMBL939            0.925764     980.0        *
      cdk_maccs      CHEMBL939            0.840580     980.0        *
   klekota_roth      CHEMBL939            0.600000     980.0        *
      *** ic50 ACTIVITY *** value prediction
      based on 11 locally validated fgps ---> 945.203
["TT_bits", "cdk_maccs", "AP_bits", "MFP1", "klekota_roth", "pubchem",
"graph", "FP2", "FeatMFP1", "RDKit7", "hybridization"]

21 rank
Beta-secretase 1 : Homo sapiens
      score: 8.129 on CHEMBL4822 based on 11 fingerprints
Fingerprint type         Ligand  Tanimoto Similarity  activity Valid fg
       FeatMFP1      CHEMBL939            0.750000   20000.0        *
           MFP1      CHEMBL939            0.688889   20000.0        *
         RDKit7      CHEMBL939            0.805133   20000.0        *
        Pattern      CHEMBL939            0.822785   20000.0
        AP_bits      CHEMBL939            0.507317   20000.0        *
        TT_bits      CHEMBL939            0.635135   20000.0        *
            FP2      CHEMBL939            0.782313   20000.0        *
  hybridization      CHEMBL939            0.816471   20000.0        *
   substructure  CHEMBL2047223            0.842105    1700.0
          graph      CHEMBL939            0.777778   20000.0        *
        pubchem      CHEMBL939            0.925764   20000.0        *
      cdk_maccs      CHEMBL939            0.840580   20000.0        *
   klekota_roth      CHEMBL939            0.600000   20000.0        *
      *** ic50 ACTIVITY *** value prediction
      based on 11 locally validated fgps ---> 19175.609
["TT_bits", "cdk_maccs", "AP_bits", "MFP1", "klekota_roth", "pubchem",
"graph", "FP2", "FeatMFP1", "RDKit7", "hybridization"]

22 rank
Serine/threonine-protein kinase RIPK2 : Homo sapiens
      score: 8.129 on CHEMBL5014 based on 11 fingerprints
Fingerprint type     Ligand  Tanimoto Similarity  activity Valid fg
       FeatMFP1  CHEMBL939            0.750000       3.8        *
```

```
          MFP1  CHEMBL939            0.688889      3.8        *
        RDKit7  CHEMBL939            0.805133      3.8        *
       Pattern  CHEMBL939            0.822785      3.8
       AP_bits  CHEMBL939            0.507317      3.8        *
       TT_bits  CHEMBL939            0.635135      3.8        *
           FP2  CHEMBL939            0.782313      3.8        *
 hybridization  CHEMBL939            0.816471      3.8        *
  substructure  CHEMBL939            0.750000      3.8
         graph  CHEMBL939            0.777778      3.8        *
       pubchem  CHEMBL939            0.925764      3.8        *
     cdk_maccs  CHEMBL939            0.840580      3.8        *
  klekota_roth  CHEMBL939            0.600000      3.8        *
     *** ic50 ACTIVITY *** value prediction
     based on 11 locally validated fgps ---> 5.709
["TT_bits", "cdk_maccs", "AP_bits", "MFP1", "klekota_roth", "pubchem",
"graph", "FP2", "FeatMFP1", "RDKit7", "hybridization"]

23 rank
HERG : Homo sapiens
     score: 8.005 on CHEMBL240 based on 11 fingerprints
Fingerprint type         Ligand  Tanimoto Similarity  activity Valid fg
      FeatMFP1  CHEMBL3623278            0.727273  17000.0        *
          MFP1  CHEMBL3623278            0.583333  17000.0        *
        RDKit7   CHEMBL193544            0.703361   5000.0        *
       Pattern      CHEMBL554            0.855025   1000.0        *
       AP_bits  CHEMBL1645462            0.519411   4000.0        *
       TT_bits      CHEMBL554            0.533333   1000.0        *
           FP2   CHEMBL193544            0.703030   5000.0        *
 hybridization   CHEMBL193544            0.709812   5000.0        *
  substructure  CHEMBL1916532            0.947368   3000.0        *
         graph   CHEMBL193544            0.705314   5000.0
       pubchem   CHEMBL193544            0.858333   5000.0        *
     cdk_maccs  CHEMBL1916533            0.864865   4000.0        *
  klekota_roth  CHEMBL3359238            0.511905   1400.0
     *** ic50 ACTIVITY *** value prediction
     based on 11 locally validated fgps ---> 6768.192
["TT_bits", "substructure", "cdk_maccs", "Pattern", "FP2", "MFP1",
"pubchem", "AP_bits", "FeatMFP1", "RDKit7", "hybridization"]

24 rank
Tyrosine-protein kinase TIE-2 : Homo sapiens
     score: 7.922 on CHEMBL4128 based on 11 fingerprints
Fingerprint type        Ligand  Tanimoto Similarity  activity Valid fg
      FeatMFP1  CHEMBL593274            0.742857      37.0        *
          MFP1  CHEMBL593274            0.588235      37.0        *
        RDKit7  CHEMBL301018            0.750727    8300.0        *
       Pattern  CHEMBL593971            0.829889    4000.0
       AP_bits  CHEMBL604893            0.609078     510.0        *
       TT_bits  CHEMBL301018            0.541667    8300.0        *
           FP2  CHEMBL301018            0.710145    8300.0        *
 hybridization  CHEMBL604893            0.700787     510.0        *
  substructure  CHEMBL593909            0.900000     615.0        *
         graph  CHEMBL301018            0.713514    8300.0
       pubchem  CHEMBL301018            0.910714    8300.0        *
     cdk_maccs  CHEMBL288441            0.847222    1191.0        *
  klekota_roth  CHEMBL593909            0.620690     615.0        *
     *** ic50 ACTIVITY *** value prediction
     based on 11 locally validated fgps ---> 2424.601
["TT_bits", "substructure", "cdk_maccs", "AP_bits", "MFP1",
"klekota_roth", "pubchem", "FP2", "FeatMFP1", "RDKit7", "hybridization"]
```

```
25 rank
Ephrin type-A receptor 2 : Homo sapiens
      score: 7.549 on CHEMBL2068 based on 10 fingerprints
Fingerprint type         Ligand  Tanimoto Similarity  activity Valid fg
       FeatMFP1  CHEMBL3322983            0.705882     300.0        *
           MFP1  CHEMBL3322981            0.541667     620.0        *
         RDKit7  CHEMBL3322973            0.772433    1470.0        *
        Pattern  CHEMBL3322972            0.846816    1190.0        *
        AP_bits  CHEMBL1090360            0.461438     370.0
        TT_bits  CHEMBL3322978            0.531646     580.0        *
            FP2  CHEMBL3322983            0.724359     300.0        *
  hybridization  CHEMBL3322983            0.760000     300.0        *
   substructure  CHEMBL3544983            0.900000      50.0        *
          graph  CHEMBL3322973            0.738693    1470.0
        pubchem  CHEMBL3322973            0.908696    1470.0        *
      cdk_maccs  CHEMBL3322987            0.857143     160.0        *
   klekota_roth  CHEMBL3322987            0.455556     160.0
      *** ic50 ACTIVITY *** value prediction
      based on 10 locally validated fgps ---> 627.315
["TT_bits", "substructure", "cdk_maccs", "Pattern", "MFP1", "pubchem",
"FP2", "FeatMFP1", "RDKit7", "hybridization"]

26 rank
Insulin receptor : Homo sapiens
      score: 7.203 on CHEMBL1981 based on 10 fingerprints
Fingerprint type         Ligand  Tanimoto Similarity  activity Valid fg
       FeatMFP1  CHEMBL2063926            0.685714   10000.0        *
           MFP1   CHEMBL301018            0.547619    2600.0        *
         RDKit7   CHEMBL301018            0.750727    2600.0        *
        Pattern  CHEMBL1630109            0.886010    3600.0        *
        AP_bits   CHEMBL502652            0.527043     100.0        *
        TT_bits  CHEMBL1630109            0.587500    3600.0        *
            FP2   CHEMBL301018            0.710145    2600.0        *
  hybridization  CHEMBL1630113            0.747788    5500.0        *
   substructure  CHEMBL3823045            0.850000      26.0        *
          graph  CHEMBL1630113            0.734694    5500.0
        pubchem   CHEMBL301018            0.910714    2600.0        *
      cdk_maccs  CHEMBL3785951            0.794872      93.0
   klekota_roth  CHEMBL3263965            0.526316   11805.0
      *** ic50 ACTIVITY *** value prediction
      based on 9 locally validated fgps ---> 4380.344
["TT_bits", "Pattern", "FP2", "MFP1", "pubchem", "AP_bits", "FeatMFP1",
"RDKit7", "hybridization"]

27 rank
Tyrosine-protein kinase LCK : Homo sapiens
      score: 7.025 on CHEMBL258 based on 10 fingerprints
Fingerprint type        Ligand  Tanimoto Similarity  activity Valid fg
       FeatMFP1  CHEMBL217092            0.694444       4.0        *
           MFP1  CHEMBL541586            0.536585   10000.0        *
         RDKit7  CHEMBL540068            0.752505    5000.0        *
        Pattern  CHEMBL288441            0.823430       1.3
        AP_bits  CHEMBL453737            0.538462       4.0        *
        TT_bits  CHEMBL541586            0.571429   10000.0        *
            FP2  CHEMBL540068            0.669118    5000.0        *
  hybridization  CHEMBL540068            0.667488    5000.0        *
   substructure  CHEMBL247468            0.850000       9.0        *
          graph  CHEMBL540068            0.705556    5000.0
        pubchem  CHEMBL540068            0.897321    5000.0        *
```

```
      cdk_maccs  CHEMBL288441            0.847222       1.3        *
   klekota_roth  CHEMBL288441            0.532609       1.3
      *** ic50 ACTIVITY *** value prediction
      based on 9 locally validated fgps ---> 4594.651
["TT_bits", "cdk_maccs", "AP_bits", "MFP1", "pubchem", "FP2", "FeatMFP1",
"RDKit7", "hybridization"]

28 rank
Cyclin-dependent kinase 2 : Homo sapiens
      score: 6.537 on CHEMBL301 based on 9 fingerprints
Fingerprint type         Ligand  Tanimoto Similarity  activity Valid fg
       FeatMFP1   CHEMBL217092            0.694444   10000.0        *
           MFP1      CHEMBL554            0.490909   11000.0
         RDKit7  CHEMBL1630110            0.694683     850.0        *
        Pattern  CHEMBL1630109            0.886010     920.0        *
        AP_bits   CHEMBL201307            0.572005   62000.0        *
        TT_bits  CHEMBL1630109            0.587500     920.0        *
            FP2  CHEMBL1630116            0.640000    1400.0        *
  hybridization  CHEMBL1630113            0.747788    1900.0        *
   substructure  CHEMBL2163984            0.850000     700.0        *
          graph  CHEMBL1630113            0.734694    1900.0
        pubchem   CHEMBL127907            0.829694    1000.0
      cdk_maccs   CHEMBL186396            0.864865     812.0        *
   klekota_roth   CHEMBL201307            0.480769   62000.0
      *** ic50 ACTIVITY *** value prediction
      based on 8 locally validated fgps ---> 7359.761
["TT_bits", "cdk_maccs", "Pattern", "AP_bits", "FP2", "FeatMFP1",
"RDKit7", "hybridization"]

29 rank
Stem cell growth factor receptor : Homo sapiens
      score: 6.036 on CHEMBL1936 based on 8 fingerprints
Fingerprint type         Ligand  Tanimoto Similarity  activity Valid fg
       FeatMFP1   CHEMBL217092            0.694444    200.00        *
           MFP1  CHEMBL3594105            0.491803     38.20
         RDKit7    CHEMBL24828            0.708850    343.00        *
        Pattern   CHEMBL330863            0.864177     21.00        *
        AP_bits  CHEMBL3764279            0.555280    162.50        *
        TT_bits  CHEMBL3628796            0.481481      3.00
            FP2    CHEMBL24828            0.617284    343.00
  hybridization    CHEMBL24828            0.654348    343.00        *
   substructure  CHEMBL1945173            0.850000   1100.00        *
          graph    CHEMBL24828            0.668293    343.00
        pubchem   CHEMBL102346            0.862069     50.00        *
      cdk_maccs   CHEMBL288441            0.847222   6313.00        *
   klekota_roth  CHEMBL3823520            0.566667      9.36
      *** ic50 ACTIVITY *** value prediction
      based on 7 locally validated fgps ---> 1090.081
["cdk_maccs", "Pattern", "pubchem", "AP_bits", "FeatMFP1", "RDKit7",
"hybridization"]

30 rank
Tyrosine-protein kinase receptor FLT3 : Homo sapiens
      score: 5.851 on CHEMBL1974 based on 8 fingerprints
Fingerprint type         Ligand  Tanimoto Similarity  activity Valid fg
       FeatMFP1  CHEMBL3545311            0.615385      2.10
           MFP1  CHEMBL3594105            0.491803    134.00
         RDKit7   CHEMBL598797            0.708257   1500.00        *
        Pattern   CHEMBL330863            0.864177    128.00        *
        AP_bits  CHEMBL1940111            0.561080     10.00        *
```

```
        TT_bits   CHEMBL598797             0.551282   1500.00        *
            FP2   CHEMBL598797             0.580838   1500.00
  hybridization   CHEMBL598797             0.639413   1500.00        *
   substructure  CHEMBL1915641             0.850000     61.00        *
          graph   CHEMBL598797             0.661458   1500.00
        pubchem   CHEMBL102346             0.862069    230.00        *
      cdk_maccs  CHEMBL3355065             0.814286     50.00        *
   klekota_roth  CHEMBL3823520             0.566667      8.91
     *** ic50 ACTIVITY *** value prediction
     based on 7 locally validated fgps ---> 638.262
["TT_bits", "cdk_maccs", "Pattern", "pubchem", "AP_bits", "RDKit7",
"hybridization"]

31 rank
MAP kinase p38 alpha : Homo sapiens
     score: 5.775 on CHEMBL260 based on 8 fingerprints
Fingerprint type         Ligand  Tanimoto Similarity  activity Valid fg
       FeatMFP1     CHEMBL7917             0.612903     560.0
           MFP1     CHEMBL7917             0.500000     560.0
         RDKit7     CHEMBL7917             0.752505     560.0        *
        Pattern   CHEMBL188114             0.860269      30.0        *
        AP_bits   CHEMBL188167             0.529183      50.0        *
        TT_bits     CHEMBL7917             0.549296     560.0        *
            FP2     CHEMBL7917             0.669118     560.0        *
  hybridization     CHEMBL7917             0.667488     560.0        *
   substructure    CHEMBL95692             0.850000      19.0        *
          graph     CHEMBL7917             0.705556     560.0
        pubchem     CHEMBL7917             0.897321     560.0        *
      cdk_maccs  CHEMBL1946341             0.767123    2800.0
   klekota_roth   CHEMBL215289             0.456311    1300.0
     *** ic50 ACTIVITY *** value prediction
     based on 7 locally validated fgps ---> 489.892
["TT_bits", "Pattern", "AP_bits", "pubchem", "FP2", "RDKit7",
"hybridization"]

32 rank
Histone deacetylase 1 : Homo sapiens
     score: 5.762 on CHEMBL325 based on 8 fingerprints
Fingerprint type         Ligand  Tanimoto Similarity  activity Valid fg
       FeatMFP1  CHEMBL3700343             0.657895     330.0
           MFP1  CHEMBL3700343             0.537037     330.0        *
         RDKit7  CHEMBL3633769             0.725184       3.5        *
        Pattern  CHEMBL1630109             0.886010      35.0        *
        AP_bits  CHEMBL3700343             0.626844     330.0        *
        TT_bits  CHEMBL1630109             0.587500      35.0        *
            FP2  CHEMBL1630110             0.619318     650.0
  hybridization  CHEMBL1630107             0.742152     610.0        *
   substructure   CHEMBL216300             0.695652       9.0
          graph  CHEMBL1630107             0.720812     610.0
        pubchem  CHEMBL3633769             0.832653       3.5        *
      cdk_maccs  CHEMBL3700343             0.824324     330.0        *
   klekota_roth  CHEMBL3700343             0.494737     330.0
     *** ic50 ACTIVITY *** value prediction
     based on 8 locally validated fgps ---> 274.996
["TT_bits", "cdk_maccs", "Pattern", "MFP1", "pubchem", "AP_bits",
"RDKit7", "hybridization"]

33 rank
Macrophage colony stimulating factor receptor : Homo sapiens
     score: 5.726 on CHEMBL1844 based on 8 fingerprints
```

Fingerprint type	Ligand	Tanimoto Similarity	activity	Valid fg
FeatMFP1	CHEMBL24828	0.676471	1600.0	
MFP1	CHEMBL481231	0.527273	7.0	*
RDKit7	CHEMBL24828	0.708850	1600.0	*
Pattern	CHEMBL125898	0.862779	1400.0	*
AP_bits	CHEMBL1940111	0.561080	400.0	*
TT_bits	CHEMBL1940267	0.487500	300.0	
FP2	CHEMBL553908	0.669118	1500.0	*
hybridization	CHEMBL553908	0.667488	1500.0	*
substructure	CHEMBL24828	0.809524	1600.0	
graph	CHEMBL553908	0.705556	1500.0	
pubchem	CHEMBL553908	0.883929	1500.0	*
cdk_maccs	CHEMBL24828	0.845070	1600.0	*
klekota_roth	CHEMBL481231	0.580645	7.0	

*** ic50 ACTIVITY *** value prediction
based on 8 locally validated fgps ---> 1230.512
["cdk_maccs", "Pattern", "AP_bits", "MFP1", "pubchem", "FP2", "RDKit7", "hybridization"]

34 rank
C-C chemokine receptor type 4 : Homo sapiens
score: 5.426 on CHEMBL2414 based on 7 fingerprints

Fingerprint type	Ligand	Tanimoto Similarity	activity	Valid fg
FeatMFP1	CHEMBL488691	0.757576	150.0	*
MFP1	CHEMBL488691	0.480000	150.0	
RDKit7	CHEMBL488690	0.688946	92.0	*
Pattern	CHEMBL488855	0.881416	130.0	*
AP_bits	CHEMBL488855	0.496372	130.0	*
TT_bits	CHEMBL488704	0.395833	220.0	
FP2	CHEMBL488690	0.604938	92.0	
hybridization	CHEMBL488690	0.579798	92.0	
substructure	CHEMBL365155	0.894737	2000.0	*
graph	CHEMBL495736	0.648649	87.0	
pubchem	CHEMBL487665	0.882609	550.0	*
cdk_maccs	CHEMBL496151	0.824324	48.0	*
klekota_roth	CHEMBL488691	0.500000	150.0	

*** ic50 ACTIVITY *** value prediction
based on 7 locally validated fgps ---> 300.844
["substructure", "Pattern", "pubchem", "AP_bits", "FeatMFP1", "RDKit7", "cdk_maccs"]

35 rank
Ephrin type-B receptor 2 : Homo sapiens
score: 4.791 on CHEMBL3290 based on 7 fingerprints

Fingerprint type	Ligand	Tanimoto Similarity	activity	Valid fg
FeatMFP1	CHEMBL288441	0.694444	8.5	*
MFP1	CHEMBL150315	0.477273	8500.0	
RDKit7	CHEMBL150315	0.712135	8500.0	*
Pattern	CHEMBL288441	0.823430	8.5	
AP_bits	CHEMBL288441	0.474648	8.5	*
TT_bits	CHEMBL127907	0.527778	1200.0	*
FP2	CHEMBL150315	0.641379	8500.0	*
hybridization	CHEMBL150315	0.611241	8500.0	
substructure	CHEMBL288441	0.772727	8.5	
graph	CHEMBL97771	0.666667	86000.0	
pubchem	CHEMBL150315	0.893805	8500.0	*
cdk_maccs	CHEMBL288441	0.847222	8.5	*
klekota_roth	CHEMBL288441	0.532609	8.5	

*** ic50 ACTIVITY *** value prediction
based on 7 locally validated fgps ---> 9424.287

```
["TT_bits", "AP_bits", "pubchem", "FP2", "FeatMFP1", "RDKit7",
"cdk_maccs"]

36 rank
Tyrosine-protein kinase CSK : Homo sapiens
     score: 4.774 on CHEMBL2634 based on 7 fingerprints
Fingerprint type         Ligand  Tanimoto Similarity  activity Valid fg
       FeatMFP1   CHEMBL369360             0.727273    7300.0        *
           MFP1   CHEMBL369360             0.571429    7300.0        *
         RDKit7   CHEMBL169186             0.696327    1700.0        *
        Pattern   CHEMBL201307             0.839607   21000.0
        AP_bits   CHEMBL201307             0.572005   21000.0        *
        TT_bits   CHEMBL201307             0.521739   21000.0        *
            FP2   CHEMBL369360             0.588889    7300.0
  hybridization   CHEMBL369360             0.626894    7300.0
   substructure   CHEMBL169186             0.809524    1700.0
          graph  CHEMBL1614725             0.668224    4700.0
        pubchem   CHEMBL169186             0.838057    1700.0        *
      cdk_maccs   CHEMBL288441             0.847222      63.0        *
   klekota_roth   CHEMBL169186             0.558140    1700.0
     *** ic50 ACTIVITY *** value prediction
     based on 7 locally validated fgps ---> 7459.712
["TT_bits", "AP_bits", "MFP1", "pubchem", "FeatMFP1", "RDKit7",
"cdk_maccs"]
```

SwissTargetPrediction report:

Reference:
Gfeller D., Michielin O. & Zoete V.
Shaping the interaction landscape of bioactive molecules, *Bioinformatics* (2013) 29:3073-3079.

Query Molecule

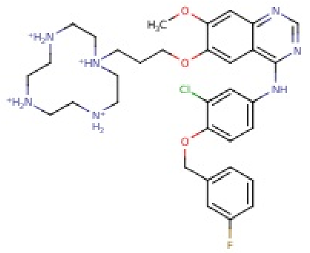

Frequency of Target Class

Target	Uniprot ID	Gene code	ChEMBL ID	Probability	# sim. cmpds (3D / 2D)	Target Class
Epidermal growth factor receptor	P00533	EGFR	CHEMBL203		27 / 755	Tyr Kinase
Receptor tyrosine-protein kinase erbB-2	P04626	ERBB2	CHEMBL1824		27 / 755	Tyr Kinase
Receptor tyrosine-protein kinase erbB-3 *(by homology)*	P21860	ERBB3	CHEMBL5838		27 / 755	Tyr Kinase
ERBB4 intracellular domain *(by homology)*	Q15303	ERBB4	CHEMBL3009		27 / 755	Tyr Kinase
Vascular endothelial growth factor receptor 1	P17948	FLT1	CHEMBL1868		24 / 216	Tyr Kinase
Vascular endothelial growth factor receptor 3 *(by homology)*	P35916	FLT4	CHEMBL1955		24 / 216	Tyr Kinase
Vascular endothelial growth factor receptor 2	P35968	KDR	CHEMBL279		24 / 216	Tyr Kinase
Serine/threonine-protein kinase Chk2	O96017	CHEK2	CHEMBL2527		5 / 17	Ser_Thr Kinase
Tyrosine-protein kinase ABL1	P00519	ABL1	CHEMBL1862		3 / 27	Tyr Kinase
Hepatocyte growth factor receptor	P08581	MET	CHEMBL3717		5 / 14	Tyr Kinase
Abelson tyrosine-protein kinase 2	P42684	ABL2	CHEMBL4014		3 / 27	Tyr Kinase
Mitogen-activated protein kinase 8 *(by homology)*	P45983	MAPK8	CHEMBL2276		6 / 27	Ser_Thr Kinase
Mitogen-activated protein kinase 9	P45984	MAPK9	CHEMBL4179		6 / 27	Ser_Thr Kinase
Mitogen-activated protein kinase 10	P53779	MAPK10	CHEMBL2637		6 / 27	Ser_Thr Kinase
Mitogen-activated protein kinase 14	Q16539	MAPK14	CHEMBL260		6 / 27	Ser_Thr Kinase

Polypharmacology Browser 2 Prediction:

Targets predicted using NN(ECfp4) + NB(ECfp4). Save Table

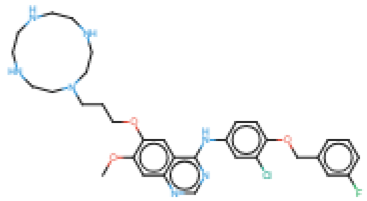

Query molecule

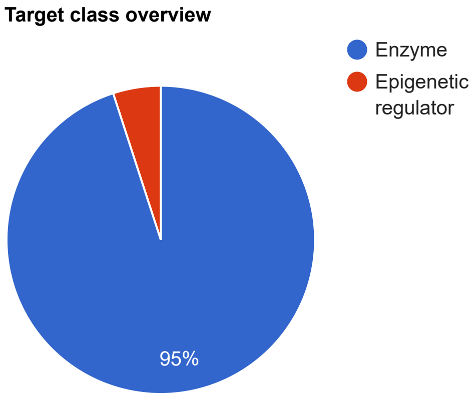

Rank	ChEMBL ID	Common name	Nearest neighbours
1	CHEMBL203 (https://www.ebi.ac.uk/chembl/target/inspect/CHEMBL203)	Epidermal growth factor receptor erbB1	Show NN
2	CHEMBL1824 (https://www.ebi.ac.uk/chembl/target/inspect/CHEMBL1824)	Receptor protein-tyrosine kinase erbB-2	Show NN
3	CHEMBL1862 (https://www.ebi.ac.uk/chembl/target/inspect/CHEMBL1862)	Tyrosine-protein kinase ABL	Show NN
4	CHEMBL1981 (https://www.ebi.ac.uk/chembl/target/inspect/CHEMBL1981)	Insulin receptor	Show NN
5	CHEMBL1913 (https://www.ebi.ac.uk/chembl/target/inspect/CHEMBL1913)	Platelet-derived growth factor receptor beta	Show NN
6	CHEMBL279 (https://www.ebi.ac.uk/chembl/target/inspect/CHEMBL279)	Vascular endothelial growth factor receptor 2	Show NN
7	CHEMBL267 (https://www.ebi.ac.uk/chembl/target/inspect/CHEMBL267)	Tyrosine-protein kinase SRC	Show NN
8	CHEMBL258 (https://www.ebi.ac.uk/chembl/target/inspect/CHEMBL258)	Tyrosine-protein kinase LCK	Show NN
9	CHEMBL1868 (https://www.ebi.ac.uk/chembl/target/inspect/CHEMBL1868)	Vascular endothelial growth factor receptor 1	Show NN
10	CHEMBL3009 (https://www.ebi.ac.uk/chembl/target/inspect/CHEMBL3009)	Receptor protein-tyrosine kinase erbB-4	Show NN
11	CHEMBL1974 (https://www.ebi.ac.uk/chembl/target/inspect/CHEMBL1974)	Tyrosine-protein kinase receptor FLT3	Show NN
12	CHEMBL2185 (https://www.ebi.ac.uk/chembl/target/inspect/CHEMBL2185)	Serine/threonine-protein kinase Aurora-B	Show NN
13	CHEMBL1955 (https://www.ebi.ac.uk/chembl/target/inspect/CHEMBL1955)	Vascular endothelial growth factor receptor 3	Show NN
14	CHEMBL1844 (https://www.ebi.ac.uk/chembl/target/inspect/CHEMBL1844)	Macrophage colony stimulating factor receptor	Show NN
15	CHEMBL3650 (https://www.ebi.ac.uk/chembl/target/inspect/CHEMBL3650)	Fibroblast growth factor receptor 1	Show NN
16	CHEMBL1936 (https://www.ebi.ac.uk/chembl/target/inspect/CHEMBL1936)	Stem cell growth factor receptor	Show NN
17	CHEMBL3024 (https://www.ebi.ac.uk/chembl/target/inspect/CHEMBL3024)	Serine/threonine-protein kinase PLK1	Show NN
18	CHEMBL2041 (https://www.ebi.ac.uk/chembl/target/inspect/CHEMBL2041)	Tyrosine-protein kinase receptor RET	Show NN

19	CHEMBL325 (https://www.ebi.ac.uk/chembl/target/inspect/CHEMBL325)	Histone deacetylase 1	Show NN
20	CHEMBL2007 (https://www.ebi.ac.uk/chembl/target/inspect/CHEMBL2007)	Platelet-derived growth factor receptor alpha	Show NN
