# Supplementary material for: Accelerating Drug Discovery by Early Protein Drug Target Prediction Based on a Multi-Fingerprint Similarity Search
Source: Molecules. 2019 Jun 14;24(12):2233. doi: 10.3390/molecules24122233 (PMC6631269; doi:10.3390/molecules24122233)
Supplement: Supplementary file 1 [file molecules-24-02233-s001.zip › File_S2.zip!/File_S2/Target_Prediction_compound#19.pdf]

MuSSel not predicted

SwissTargetPrediction report:

Reference:
Gfeller D., Michielin O. & Zoete V.
Shaping the interaction landscape of bioactive molecules, *Bioinformatics* (2013) 29:3073-3079.

Query Molecule

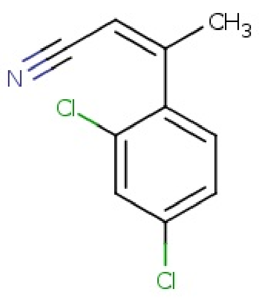

Frequency of Target Class

Target	Uniprot ID	Gene code	ChEMBL ID	Probability	# sim. cmpds (3D / 2D)	Target Class
Sodium-dependent noradrenaline transporter *(by homology)*	P23975	SLC6A2	CHEMBL222		2 / 2	Transporter
Sodium-dependent dopamine transporter	Q01959	SLC6A3	CHEMBL238		2 / 2	Transporter
Sodium-dependent serotonin transporter	P31645	SLC6A4	CHEMBL228		2 / 1	Transporter
Sodium- and chloride-dependent glycine transporter 1 *(by homology)*	P48067	SLC6A9	CHEMBL2337		2 / 1	Transporter
Sodium-dependent proline transporter *(by homology)*	Q99884	SLC6A7			2 / 1	Transporter
Sodium- and chloride-dependent neutral and basic amino acid transporter B(0+) *(by homology)*	Q9UN76	SLC6A14			2 / 1	Transporter
Sodium- and chloride-dependent glycine transporter 2 *(by homology)*	Q9Y345	SLC6A5	CHEMBL3060		2 / 1	Transporter
Alpha-2A adrenergic receptor	P08913	ADRA2A	CHEMBL1867		1 / 1	Membrane receptor
Alpha-2C adrenergic receptor	P18825	ADRA2C	CHEMBL1916		1 / 1	Membrane receptor
Adenosine receptor A3	P33765	ADORA3	CHEMBL256		1 / 1	Membrane receptor
Alpha-2B adrenergic receptor *(by homology)*	P18089	ADRA2B	CHEMBL1942		1 / 1	Membrane receptor
Amine oxidase [flavin-containing] A	P21397	MAOA	CHEMBL1951		2 / 2	Enzyme
Amine oxidase [flavin-containing] B	P27338	MAOB	CHEMBL2039		2 / 2	Enzyme
Microtubule-associated protein tau	P10636	MAPT	CHEMBL1293224		5 / 2	Unclassified
Indoleamine 2,3-dioxygenase 1	P14902	IDO1	CHEMBL4685		7 / 0	Enzyme

Polypharmacology Browser 2 not predicted
