# Supplementary material for: Accelerating Drug Discovery by Early Protein Drug Target Prediction Based on a Multi-Fingerprint Similarity Search
Source: Molecules. 2019 Jun 14;24(12):2233. doi: 10.3390/molecules24122233 (PMC6631269; doi:10.3390/molecules24122233)
Supplement: Supplementary file 1 [file molecules-24-02233-s001.zip › File_S2.zip!/File_S2/Target_Prediction_compound#2.pdf]

MuSSel Prediction IC_{50}:

```
1 rank
Heat shock protein HSP 90-alpha : Homo sapiens
      score: 4.912 on CHEMBL3880 based on 7 fingerprints
Fingerprint type         Ligand  Tanimoto Similarity  activity Valid fg
       FeatMFP1  CHEMBL3653872             0.730769    1490.0        *
           MFP1  CHEMBL3656930             0.625000     340.0        *
         RDKit7  CHEMBL3653943             0.532567     290.0
        Pattern  CHEMBL2237431             0.790155   17300.0
        AP_bits  CHEMBL3653862             0.448363     140.0
        TT_bits  CHEMBL3653862             0.561404     140.0        *
            FP2  CHEMBL3653943             0.661157     290.0        *
  hybridization  CHEMBL3653857             0.652582     270.0        *
   substructure  CHEMBL3653882             0.818182     280.0
          graph  CHEMBL3653943             0.737500     290.0
        pubchem  CHEMBL3653857             0.752688     270.0
      cdk_maccs  CHEMBL3653855             0.836735     150.0        *
   klekota_roth  CHEMBL3653883             0.844828     120.0        *
      *** ic50 ACTIVITY *** value prediction
      based on 7 locally validated fgps ---> 315.768
["TT_bits", "klekota_roth", "MFP1", "FP2", "FeatMFP1", "cdk_maccs",
"hybridization"]
```

SwissTargetPrediction report:

Reference:
Gfeller D., Michielin O. & Zoete V.
Shaping the interaction landscape of bioactive molecules, *Bioinformatics* (2013) 29:3073-3079.

Query Molecule

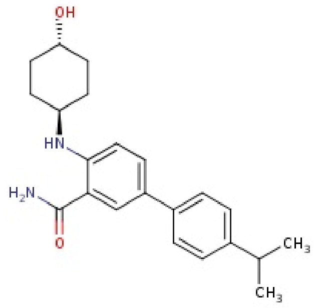

Frequency of Target Class

Target	Uniprot ID	Gene code	ChEMBL ID	Probability	# sim. cmpds (3D / 2D)	Target Class
Proteinase-activated receptor 1	P25116	F2R	CHEMBL3974		17 / 1	Membrane receptor
Mitogen-activated protein kinase 8 *(by homology)*	P45983	MAPK8	CHEMBL2276		232 / 42	Ser_Thr Kinase
Mitogen-activated protein kinase 9 *(by homology)*	P45984	MAPK9	CHEMBL4179		232 / 42	Ser_Thr Kinase
Mitogen-activated protein kinase 10 *(by homology)*	P53779	MAPK10	CHEMBL2637		232 / 42	Ser_Thr Kinase
Mitogen-activated protein kinase 11 *(by homology)*	Q15759	MAPK11	CHEMBL3961		240 / 42	Ser_Thr Kinase
Mitogen-activated protein kinase 14	Q16539	MAPK14	CHEMBL260		240 / 42	Ser_Thr Kinase
Cannabinoid receptor 1	P21554	CNR1	CHEMBL218		331 / 6	Membrane receptor
Cannabinoid receptor 2	P34972	CNR2	CHEMBL253		202 / 6	Membrane receptor
C-C chemokine receptor type 1	P32246	CCR1	CHEMBL2413		133 / 9	Membrane receptor
C-C chemokine receptor type 2	P41597	CCR2	CHEMBL4015		133 / 9	Membrane receptor
C-C chemokine receptor type 3 *(by homology)*	P51677	CCR3	CHEMBL3473		133 / 9	Membrane receptor
C-C chemokine receptor type 5 *(by homology)*	P51681	CCR5	CHEMBL274		133 / 9	Membrane receptor
Histamine H3 receptor	Q9Y5N1	HRH3	CHEMBL264		28 / 4	Membrane receptor
Mu-type opioid receptor	P35372	OPRM1	CHEMBL233		105 / 53	Membrane receptor
Delta-type opioid receptor	P41143	OPRD1	CHEMBL236		105 / 53	Membrane receptor

Polypharmacology Browser 2 Prediction:

Targets predicted using NN(ECfp4) + NB(ECfp4). Save Table

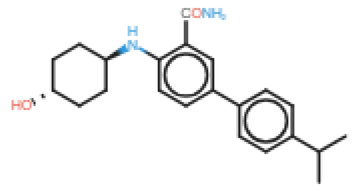

Query molecule

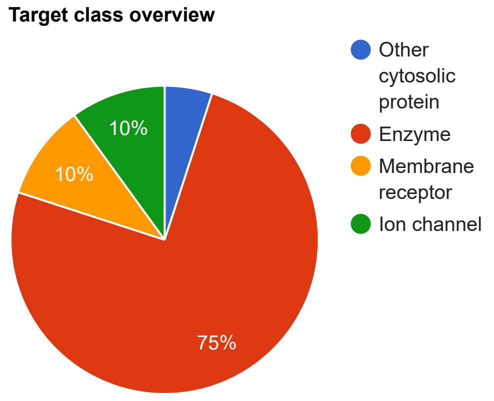

Rank	ChEMBL ID	Common name	Nearest neighbours
1	CHEMBL3880 (https://www.ebi.ac.uk/chembl/target/inspect/CHEMBL3880)	Heat shock protein HSP 90-alpha	Show NN
2	CHEMBL2971 (https://www.ebi.ac.uk/chembl/target/inspect/CHEMBL2971)	Tyrosine-protein kinase JAK2	Show NN
3	CHEMBL2835 (https://www.ebi.ac.uk/chembl/target/inspect/CHEMBL2835)	Tyrosine-protein kinase JAK1	Show NN
4	CHEMBL2599 (https://www.ebi.ac.uk/chembl/target/inspect/CHEMBL2599)	Tyrosine-protein kinase SYK	Show NN
5	CHEMBL240 (https://www.ebi.ac.uk/chembl/target/inspect/CHEMBL240)	HERG	Show NN
6	CHEMBL4235 (https://www.ebi.ac.uk/chembl/target/inspect/CHEMBL4235)	11-beta-hydroxysteroid dehydrogenase 1	Show NN
7	CHEMBL260 (https://www.ebi.ac.uk/chembl/target/inspect/CHEMBL260)	MAP kinase p38 alpha	Show NN
8	CHEMBL2148 (https://www.ebi.ac.uk/chembl/target/inspect/CHEMBL2148)	Tyrosine-protein kinase JAK3	Show NN
9	CHEMBL2276 (https://www.ebi.ac.uk/chembl/target/inspect/CHEMBL2276)	c-Jun N-terminal kinase 1	Show NN
10	CHEMBL1974 (https://www.ebi.ac.uk/chembl/target/inspect/CHEMBL1974)	Tyrosine-protein kinase receptor FLT3	Show NN
11	CHEMBL4247 (https://www.ebi.ac.uk/chembl/target/inspect/CHEMBL4247)	ALK tyrosine kinase receptor	Show NN
12	CHEMBL4794 (https://www.ebi.ac.uk/chembl/target/inspect/CHEMBL4794)	Vanilloid receptor	Show NN
13	CHEMBL1075104 (https://www.ebi.ac.uk/chembl/target/inspect/CHEMBL1075104)	Leucine-rich repeat serine/threonine-protein kinase 2	Show NN
14	CHEMBL284 (https://www.ebi.ac.uk/chembl/target/inspect/CHEMBL284)	Dipeptidyl peptidase IV	Show NN
15	CHEMBL253 (https://www.ebi.ac.uk/chembl/target/inspect/CHEMBL253)	Cannabinoid CB2 receptor	Show NN
16	CHEMBL1991 (https://www.ebi.ac.uk/chembl/target/inspect/CHEMBL1991)	Inhibitor of nuclear factor kappa B kinase beta subunit	Show NN
17	CHEMBL213 (https://www.ebi.ac.uk/chembl/target/inspect/CHEMBL213)	Beta-1 adrenergic receptor	Show NN

18	CHEMBL262 (https://www.ebi.ac.uk/chembl/target/inspect/CHEMBL262)	Glycogen synthase kinase-3 beta	Show NN
19	CHEMBL2147 (https://www.ebi.ac.uk/chembl/target/inspect/CHEMBL2147)	Serine/threonine-protein kinase PIM1	Show NN
20	CHEMBL5314 (https://www.ebi.ac.uk/chembl/target/inspect/CHEMBL5314)	Tyrosine-protein kinase receptor TYRO3	Show NN
