# Supplementary material for: Accelerating Drug Discovery by Early Protein Drug Target Prediction Based on a Multi-Fingerprint Similarity Search
Source: Molecules. 2019 Jun 14;24(12):2233. doi: 10.3390/molecules24122233 (PMC6631269; doi:10.3390/molecules24122233)
Supplement: Supplementary file 1 [file molecules-24-02233-s001.zip › File_S2.zip!/File_S2/Target_Prediction_compound#20.pdf]

MuSSel not predicted

SwissTargetPrediction report:

Reference:
Gfeller D., Michielin O. & Zoete V.
Shaping the interaction landscape of bioactive molecules, *Bioinformatics* (2013) 29:3073-3079.

Query Molecule

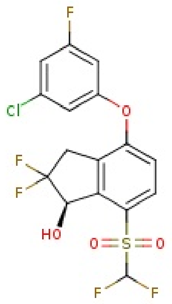

Frequency of Target Class

Target	Uniprot ID	Gene code	ChEMBL ID	Probability	# sim. cmpds (3D / 2D)	Target Class
Peroxisome proliferator-activated receptor gamma *(by homology)*	P37231	PPARG	CHEMBL235		0 / 16	Transcription Factor
Peroxisome proliferator-activated receptor delta	Q03181	PPARD	CHEMBL3979		0 / 16	Transcription Factor
Peroxisome proliferator-activated receptor alpha	Q07869	PPARA	CHEMBL239		0 / 16	Transcription Factor
Microtubule-associated protein tau	P10636	MAPT	CHEMBL1293224		2 / 0	Unclassified
Tyrosine-protein phosphatase non-receptor type 1	P18031	PTPN1	CHEMBL335		1 / 0	Tyr Phosphatase
Tyrosine-protein phosphatase non-receptor type 2 *(by homology)*	P17706	PTPN2	CHEMBL3807		1 / 0	Tyr Phosphatase
Adenosine receptor A1	P30542	ADORA1	CHEMBL226		1 / 0	Membrane receptor
Adenosine receptor A2a *(by homology)*	P29274	ADORA2A	CHEMBL251		1 / 0	Membrane receptor
Adenosine receptor A2b *(by homology)*	P29275	ADORA2B	CHEMBL255		1 / 0	Membrane receptor
Sodium channel protein type 9 subunit alpha	Q15858	SCN9A	CHEMBL4296		1 / 0	Ion channel
Sodium channel protein type 1 subunit alpha *(by homology)*	P35498	SCN1A	CHEMBL1845		1 / 0	Ion channel
Sodium channel protein type 2 subunit alpha *(by homology)*	Q99250	SCN2A	CHEMBL4187		1 / 0	Ion channel
Sodium channel protein type 3 subunit alpha *(by homology)*	Q9NY46	SCN3A	CHEMBL5163		1 / 0	Ion channel
Sodium channel protein type 8 subunit alpha *(by homology)*	Q9UQD0	SCN8A	CHEMBL5202		1 / 0	Ion channel
Sodium-dependent noradrenaline transporter	P23975	SLC6A2	CHEMBL222		1 / 0	Transporter

Polypharmacology Browser 2 Prediction:

Targets predicted using NN(ECfp4) + NB(ECfp4). Save Table

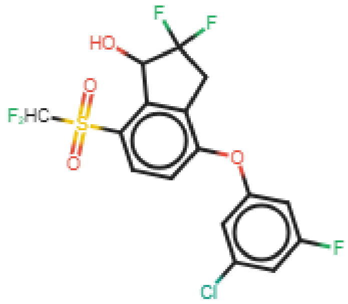

Query molecule

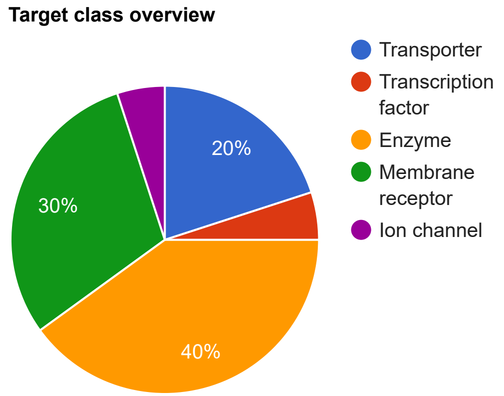

Rank	ChEMBL ID	Common name	Nearest neighbours
1	CHEMBL205 (https://www.ebi.ac.uk/chembl/target/inspect/CHEMBL205)	Carbonic anhydrase II	Show NN
2	CHEMBL3371 (https://www.ebi.ac.uk/chembl/target/inspect/CHEMBL3371)	Serotonin 6 (5-HT6) receptor	Show NN
3	CHEMBL228 (https://www.ebi.ac.uk/chembl/target/inspect/CHEMBL228)	Serotonin transporter	Show NN
4	CHEMBL261 (https://www.ebi.ac.uk/chembl/target/inspect/CHEMBL261)	Carbonic anhydrase I	Show NN
5	CHEMBL313 (https://www.ebi.ac.uk/chembl/target/inspect/CHEMBL313)	Serotonin transporter	Show NN
6	CHEMBL240 (https://www.ebi.ac.uk/chembl/target/inspect/CHEMBL240)	HERG	Show NN
7	CHEMBL222 (https://www.ebi.ac.uk/chembl/target/inspect/CHEMBL222)	Norepinephrine transporter	Show NN
8	CHEMBL340 (https://www.ebi.ac.uk/chembl/target/inspect/CHEMBL340)	Cytochrome P450 3A4	Show NN
9	CHEMBL238 (https://www.ebi.ac.uk/chembl/target/inspect/CHEMBL238)	Dopamine transporter	Show NN
10	CHEMBL230 (https://www.ebi.ac.uk/chembl/target/inspect/CHEMBL230)	Cyclooxygenase-2	Show NN
11	CHEMBL5071 (https://www.ebi.ac.uk/chembl/target/inspect/CHEMBL5071)	G protein-coupled receptor 44	Show NN
12	CHEMBL224 (https://www.ebi.ac.uk/chembl/target/inspect/CHEMBL224)	Serotonin 2a (5-HT2a) receptor	Show NN
13	CHEMBL1871 (https://www.ebi.ac.uk/chembl/target/inspect/CHEMBL1871)	Androgen Receptor	Show NN
14	CHEMBL2564 (https://www.ebi.ac.uk/chembl/target/inspect/CHEMBL2564)	Metabotropic glutamate receptor 5	Show NN
15	CHEMBL3594 (https://www.ebi.ac.uk/chembl/target/inspect/CHEMBL3594)	Carbonic anhydrase IX	Show NN
16	CHEMBL2069 (https://www.ebi.ac.uk/chembl/target/inspect/CHEMBL2069)	Thromboxane A2 receptor	Show NN
17	CHEMBL280 (https://www.ebi.ac.uk/chembl/target/inspect/CHEMBL280)	Matrix metalloproteinase 13	Show NN
18	CHEMBL253 (https://www.ebi.ac.uk/chembl/target/inspect/CHEMBL253)	Cannabinoid CB2 receptor	Show NN

19	CHEMBL221 (https://www.ebi.ac.uk/chembl/target/inspect/CHEMBL221)	Cyclooxygenase-1	Show NN
20	CHEMBL4068 (https://www.ebi.ac.uk/chembl/target/inspect/CHEMBL4068)	Chymase	Show NN
