# Supplementary material for: Accelerating Drug Discovery by Early Protein Drug Target Prediction Based on a Multi-Fingerprint Similarity Search
Source: Molecules. 2019 Jun 14;24(12):2233. doi: 10.3390/molecules24122233 (PMC6631269; doi:10.3390/molecules24122233)
Supplement: Supplementary file 1 [file molecules-24-02233-s001.zip › File_S2.zip!/File_S2/Target_Prediction_compound#21.pdf]

MuSSel not predicted

SwissTargetPrediction report:

Reference:
Gfeller D., Michielin O. & Zoete V.
Shaping the interaction landscape of bioactive molecules, *Bioinformatics* (2013) 29:3073-3079.

Query Molecule

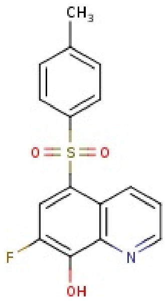

Frequency of Target Class

Target	Uniprot ID	Gene code	ChEMBL ID	Probability	# sim. cmpds (3D / 2D)	Target Class
Dual specificity tyrosine-phosphorylation-regulated kinase 1A *(by homology)*	Q13627	DYRK1A	CHEMBL2292		90 / 1	Ser_Thr_Tyr Kinase
Prostaglandin G/H synthase 1 *(by homology)*	P23219	PTGS1	CHEMBL221		80 / 21	Enzyme
Prostaglandin G/H synthase 2	P35354	PTGS2	CHEMBL230		80 / 21	Enzyme
Microtubule-associated protein tau	P10636	MAPT	CHEMBL1293224		710 / 1	Unclassified
Thymidylate synthase	P04818	TYMS	CHEMBL1952		49 / 3	Enzyme
Tyrosine-protein kinase Fyn *(by homology)*	P06241	FYN	CHEMBL1841		48 / 4	Tyr Kinase
Proto-oncogene tyrosine-protein kinase Src	P12931	SRC	CHEMBL267		48 / 4	Tyr Kinase
Tyrosine-protein kinase FRK *(by homology)*	P42685	FRK	CHEMBL4223		48 / 4	Tyr Kinase
Tyrosine-protein kinase Yes *(by homology)*	P07947	YES1	CHEMBL2073		48 / 4	Tyr Kinase
Tyrosine-protein kinase Fgr *(by homology)*	P09769	FGR	CHEMBL4454		48 / 4	Tyr Kinase
Poly [ADP-ribose] polymerase 1	P09874	PARP1	CHEMBL3105		50 / 2	Enzyme
Tyrosyl-DNA phosphodiesterase 1	Q9NUW8	TDP1	CHEMBL1075138		172 / 1	Enzyme
PEX	P08253	MMP2	CHEMBL333		15 / 1	Metallo Protease
67 kDa matrix metalloproteinase-9 *(by homology)*	P14780	MMP9	CHEMBL321		15 / 1	Metallo Protease
FAD-linked sulfhydryl oxidase ALR	P55789	GFER	CHEMBL1741189		87 / 1	Enzyme

Polypharmacology Browser 2 Prediction:

Targets predicted using NN(ECfp4) + NB(ECfp4). Save Table

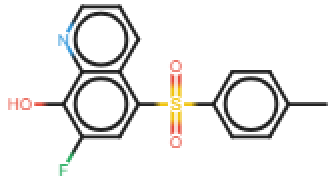

Query molecule

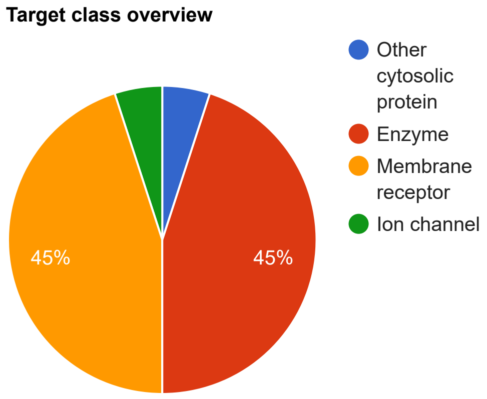

Rank	ChEMBL ID	Common name	Nearest neighbours
1	CHEMBL3371 (https://www.ebi.ac.uk/chembl/target/inspect/CHEMBL3371)	Serotonin 6 (5-HT6) receptor	Show NN
2	CHEMBL333 (https://www.ebi.ac.uk/chembl/target/inspect/CHEMBL333)	Matrix metalloproteinase-2	Show NN
3	CHEMBL5071 (https://www.ebi.ac.uk/chembl/target/inspect/CHEMBL5071)	G protein-coupled receptor 44	Show NN
4	CHEMBL218 (https://www.ebi.ac.uk/chembl/target/inspect/CHEMBL218)	Cannabinoid CB1 receptor	Show NN
5	CHEMBL230 (https://www.ebi.ac.uk/chembl/target/inspect/CHEMBL230)	Cyclooxygenase-2	Show NN
6	CHEMBL205 (https://www.ebi.ac.uk/chembl/target/inspect/CHEMBL205)	Carbonic anhydrase II	Show NN
7	CHEMBL253 (https://www.ebi.ac.uk/chembl/target/inspect/CHEMBL253)	Cannabinoid CB2 receptor	Show NN
8	CHEMBL2564 (https://www.ebi.ac.uk/chembl/target/inspect/CHEMBL2564)	Metabotropic glutamate receptor 5	Show NN
9	CHEMBL224 (https://www.ebi.ac.uk/chembl/target/inspect/CHEMBL224)	Serotonin 2a (5-HT2a) receptor	Show NN
10	CHEMBL225 (https://www.ebi.ac.uk/chembl/target/inspect/CHEMBL225)	Serotonin 2c (5-HT2c) receptor	Show NN
11	CHEMBL4235 (https://www.ebi.ac.uk/chembl/target/inspect/CHEMBL4235)	11-beta-hydroxysteroid dehydrogenase 1	Show NN
12	CHEMBL279 (https://www.ebi.ac.uk/chembl/target/inspect/CHEMBL279)	Vascular endothelial growth factor receptor 2	Show NN
13	CHEMBL240 (https://www.ebi.ac.uk/chembl/target/inspect/CHEMBL240)	HERG	Show NN
14	CHEMBL254 (https://www.ebi.ac.uk/chembl/target/inspect/CHEMBL254)	Phosphodiesterase 4A	Show NN
15	CHEMBL4005 (https://www.ebi.ac.uk/chembl/target/inspect/CHEMBL4005)	PI3-kinase p110-alpha subunit	Show NN

16	CHEMBL1900 (https://www.ebi.ac.uk/chembl/target/inspect/CHEMBL1900)	Aldose reductase	Show NN
17	CHEMBL3155 (https://www.ebi.ac.uk/chembl/target/inspect/CHEMBL3155)	Serotonin 7 (5-HT7) receptor	Show NN
18	CHEMBL4777 (https://www.ebi.ac.uk/chembl/target/inspect/CHEMBL4777)	Neuropeptide Y receptor type 1	Show NN
19	CHEMBL4361 (https://www.ebi.ac.uk/chembl/target/inspect/CHEMBL4361)	Induced myeloid leukemia cell differentiation protein Mcl-1	Show NN
20	CHEMBL3717 (https://www.ebi.ac.uk/chembl/target/inspect/CHEMBL3717)	Hepatocyte growth factor receptor	Show NN
