# Supplementary material for: Accelerating Drug Discovery by Early Protein Drug Target Prediction Based on a Multi-Fingerprint Similarity Search
Source: Molecules. 2019 Jun 14;24(12):2233. doi: 10.3390/molecules24122233 (PMC6631269; doi:10.3390/molecules24122233)
Supplement: Supplementary file 1 [file molecules-24-02233-s001.zip › File_S2.zip!/File_S2/Target_Prediction_compound#22.pdf]

MuSSel not predicted

SwissTargetPrediction report:

Reference:
Gfeller D., Michielin O. & Zoete V.
Shaping the interaction landscape of bioactive molecules, *Bioinformatics* (2013) 29:3073-3079.

Query Molecule

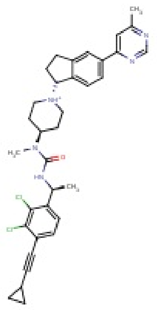

Frequency of Target Class

Target	Uniprot ID	Gene code	ChEMBL ID	Probability	# sim. cmpds (3D / 2D)	Target Class
C-C chemokine receptor type 1 *(by homology)*	P32246	CCR1	CHEMBL2413		560 / 47	Membrane receptor
C-C chemokine receptor type 2 *(by homology)*	P41597	CCR2	CHEMBL4015		571 / 47	Membrane receptor
C-C chemokine receptor type 3 *(by homology)*	P51677	CCR3	CHEMBL3473		560 / 47	Membrane receptor
C-C chemokine receptor type 5	P51681	CCR5	CHEMBL274		571 / 47	Membrane receptor
C-C chemokine receptor-like 2 *(by homology)*	C9JP23	CCRL2			532 / 47	Membrane receptor
Potassium voltage-gated channel subfamily H member 2	Q12809	KCNH2	CHEMBL240		327 / 13	Ion channel
Potassium voltage-gated channel subfamily H member 6 *(by homology)*	Q9H252	KCNH6			327 / 13	Ion channel
Potassium voltage-gated channel subfamily H member 7 *(by homology)*	Q9NS40	KCNH7			327 / 13	Ion channel
D(2) dopamine receptor	P14416	DRD2	CHEMBL217		1112 / 2	Membrane receptor
D(3) dopamine receptor	P35462	DRD3	CHEMBL234		748 / 1	Membrane receptor
D(4) dopamine receptor	P21917	DRD4	CHEMBL219		430 / 1	Membrane receptor
RANTES(4-68)	P13501	CCL5	CHEMBL1275217		21 / 4	Unclassified
C-C motif chemokine 16 *(by homology)*	O15467	CCL16			21 / 4	Unclassified
MIP-1-alpha(4-69) *(by homology)*	P10147	CCL3			21 / 4	Unclassified
MIP-1-beta(3-69) *(by homology)*	P13236	CCL4			21 / 4	Unclassified

Polypharmacology Browser 2 not predicted
