# Supplementary material for: Accelerating Drug Discovery by Early Protein Drug Target Prediction Based on a Multi-Fingerprint Similarity Search
Source: Molecules. 2019 Jun 14;24(12):2233. doi: 10.3390/molecules24122233 (PMC6631269; doi:10.3390/molecules24122233)
Supplement: Supplementary file 1 [file molecules-24-02233-s001.zip › File_S2.zip!/File_S2/Target_Prediction_compound#23.pdf]

MuSSel not predicted

SwissTargetPrediction report:

Reference:
Gfeller D., Michielin O. & Zoete V.
Shaping the interaction landscape of bioactive molecules, *Bioinformatics* (2013) 29:3073-3079.

Query Molecule

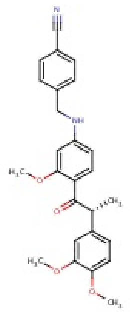

Frequency of Target Class

Target	Uniprot ID	Gene code	ChEMBL ID	Probability	# sim. cmpds (3D / 2D)	Target Class
Cholinesterase *(by homology)*	P06276	BCHE	CHEMBL1914		92 / 30	Enzyme
Acetylcholinesterase	P22303	ACHE	CHEMBL220		92 / 30	Enzyme
Arachidonate 5-lipoxygenase	P09917	ALOX5	CHEMBL215		19 / 15	Enzyme
Arachidonate 15-lipoxygenase	P16050	ALOX15	CHEMBL2903		18 / 15	Enzyme
Arachidonate 12-lipoxygenase, 12S-type *(by homology)*	P18054	ALOX12	CHEMBL3687		18 / 15	Enzyme
Arachidonate 15-lipoxygenase B *(by homology)*	O15296	ALOX15B	CHEMBL2457		18 / 15	Enzyme
Epidermis-type lipoxygenase 3 *(by homology)*	Q9BYJ1	ALOXE3			18 / 15	Enzyme
Microtubule-associated protein tau	P10636	MAPT	CHEMBL1293224		1170 / 43	Unclassified
Arachidonate 12-lipoxygenase, 12R-type *(by homology)*	O75342	ALOX12B			17 / 1	Enzyme
FAD-linked sulfhydryl oxidase ALR	P55789	GFER	CHEMBL1741189		60 / 9	Enzyme
Heat shock protein HSP 90-alpha	P07900	HSP90AA1	CHEMBL3880		29 / 1	Cytosolic other
Heat shock protein HSP 90-beta	P08238	HSP90AB1	CHEMBL4303		29 / 1	Cytosolic other
Endoplasmin *(by homology)*	P14625	HSP90B1	CHEMBL1075323		29 / 1	Unclassified
Muscleblind-like protein 1	Q9NR56	MBNL1	CHEMBL1293317		426 / 19	Unclassified
Muscleblind-like protein 2 *(by homology)*	Q5VZF2	MBNL2			426 / 19	Unclassified

Polypharmacology Browser 2 not predicted
