# Supplementary material for: Accelerating Drug Discovery by Early Protein Drug Target Prediction Based on a Multi-Fingerprint Similarity Search
Source: Molecules. 2019 Jun 14;24(12):2233. doi: 10.3390/molecules24122233 (PMC6631269; doi:10.3390/molecules24122233)
Supplement: Supplementary file 1 [file molecules-24-02233-s001.zip › File_S2.zip!/File_S2/Target_Prediction_compound#24.pdf]

MuSSel Prediction IC_{50}:

```
1 rank
HERG : Homo sapiens
     score: 5.867 on CHEMBL240 based on 8 fingerprints
Fingerprint type         Ligand  Tanimoto Similarity  activity Valid fg
       FeatMFP1   CHEMBL549438            0.740741  50118.72        *
           MFP1  CHEMBL2069398            0.564103   6000.00        *
         RDKit7  CHEMBL2041188            0.511613  23000.00
        Pattern   CHEMBL258176            0.877510  25118.86        *
        AP_bits  CHEMBL2043004            0.512195    220.00        *
        TT_bits   CHEMBL575475            0.363636   2658.23
            FP2  CHEMBL2041188            0.537736  23000.00
  hybridization   CHEMBL100566            0.471223   8200.00
   substructure   CHEMBL214983            0.875000   4000.00        *
          graph   CHEMBL485161            0.672515  50000.00
        pubchem  CHEMBL2325987            0.846995  26957.00        *
      cdk_maccs   CHEMBL214983            0.842105   4000.00        *
   klekota_roth   CHEMBL552023            0.608696   3100.00        *
     *** ic50 ACTIVITY *** value prediction
     based on 8 locally validated fgps ---> 14170.092
["substructure", "Pattern", "MFP1", "pubchem", "AP_bits", "FeatMFP1",
"klekota_roth", "cdk_maccs"]
```

MuSSel Prediction k_i:

```
1 rank
Orexin receptor 2 : Homo sapiens
     score: 3.177 on CHEMBL4792 based on 4 fingerprints
Fingerprint type         Ligand  Tanimoto Similarity  activity Valid fg
       FeatMFP1  CHEMBL3646199             0.730769    716.00
           MFP1  CHEMBL3646199             0.564103    716.00
         RDKit7  CHEMBL3670574             0.494475     72.00
        Pattern  CHEMBL3099889             0.874747      4.80        *
        AP_bits  CHEMBL3669443             0.567929     35.00        *
        TT_bits  CHEMBL3670618             0.316327     28.00
            FP2  CHEMBL3670574             0.571429     72.00
  hybridization  CHEMBL3670574             0.522772     72.00
   substructure  CHEMBL2435413             0.800000     12.88
          graph  CHEMBL3663562             0.660606    934.00
        pubchem  CHEMBL2435401             0.897727     20.89        *
      cdk_maccs  CHEMBL3646199             0.836364    716.00        *
   klekota_roth  CHEMBL1083358             0.500000      0.17
     *** ki ACTIVITY *** value prediction
     based on 4 locally validated fgps ---> 219.194
["Pattern", "cdk_maccs", "pubchem", "AP_bits"]

2 rank
Orexin receptor 1 : Homo sapiens
     score: 3.177 on CHEMBL5113 based on 4 fingerprints
Fingerprint type         Ligand  Tanimoto Similarity  activity Valid fg
       FeatMFP1  CHEMBL3646199             0.730769  10000.00
           MFP1  CHEMBL3646199             0.564103  10000.00
         RDKit7  CHEMBL3670574             0.494475   3841.00
        Pattern  CHEMBL3099889             0.874747    833.00        *
        AP_bits  CHEMBL3669443             0.567929    365.00        *
        TT_bits  CHEMBL3670618             0.316327    143.00
            FP2  CHEMBL3670574             0.571429   3841.00
  hybridization  CHEMBL3670574             0.522772   3841.00
   substructure  CHEMBL2435413             0.800000     58.88
          graph  CHEMBL3663562             0.660606     77.00
        pubchem  CHEMBL2435401             0.897727   1445.44        *
      cdk_maccs  CHEMBL3646199             0.836364  10000.00        *
   klekota_roth  CHEMBL1083358             0.500000      1.80
     *** ki ACTIVITY *** value prediction
     based on 4 locally validated fgps ---> 3000.118
["Pattern", "cdk_maccs", "pubchem", "AP_bits"]
```

SwissTargetPrediction report:

Reference:
Gfeller D., Michielin O. & Zoete V.
Shaping the interaction landscape of bioactive molecules, *Bioinformatics* (2013) 29:3073-3079.

Query Molecule

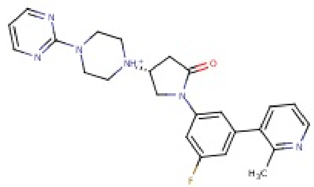

Frequency of Target Class

Target	Uniprot ID	Gene code	ChEMBL ID	Probability	# sim. cmpds (3D / 2D)	Target Class
cGMP-specific 3',5'-cyclic phosphodiesterase	O76074	PDE5A	CHEMBL1827		143 / 2	Enzyme
Dual 3',5'-cyclic-AMP and -GMP phosphodiesterase 11A	Q9HCR9	PDE11A	CHEMBL2717		125 / 2	Enzyme
D(2) dopamine receptor	P14416	DRD2	CHEMBL217		2174 / 33	Membrane receptor
Dipeptidyl peptidase 4 membrane form	P27487	DPP4	CHEMBL284		592 / 22	Serine Protease
Seprase *(by homology)*	Q12884	FAP	CHEMBL4683		592 / 22	Serine Protease
D(4) dopamine receptor	P21917	DRD4	CHEMBL219		685 / 11	Membrane receptor
D(3) dopamine receptor	P35462	DRD3	CHEMBL234		1132 / 15	Membrane receptor
Alpha-1D adrenergic receptor	P25100	ADRA1D	CHEMBL223		426 / 7	Membrane receptor
Alpha-1A adrenergic receptor	P35348	ADRA1A	CHEMBL229		487 / 16	Membrane receptor
Alpha-1B adrenergic receptor	P35368	ADRA1B	CHEMBL232		426 / 7	Membrane receptor
Neuronal acetylcholine receptor subunit alpha-7 *(by homology)*	P36544	CHRNA7	CHEMBL2492		141 / 24	Ion channel
CHRNA7-FAM7A fusion protein *(by homology)*	Q494W8	CHRFAM7A			141 / 24	Ion channel
Egl nine homolog 2	Q96KS0	EGLN2	CHEMBL3028		10 / 35	Enzyme
Egl nine homolog 1	Q9GZT9	EGLN1	CHEMBL5697		10 / 35	Enzyme
Potassium voltage-gated channel subfamily H member 2	Q12809	KCNH2	CHEMBL240		518 / 34	Ion channel

Polypharmacology Browser 2 Prediction:

Targets predicted using NN(ECfp4) + NB(ECfp4). Save Table

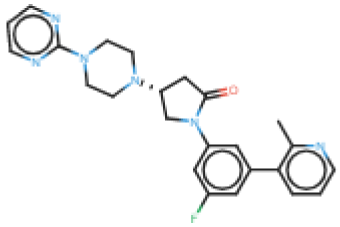

Query molecule

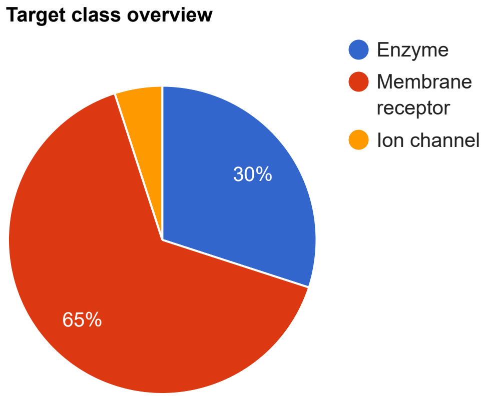

Rank	ChEMBL ID	Common name	Nearest neighbours
1	CHEMBL284 (https://www.ebi.ac.uk/chembl/target/inspect/CHEMBL284)	Dipeptidyl peptidase IV	Show NN
2	CHEMBL3227 (https://www.ebi.ac.uk/chembl/target/inspect/CHEMBL3227)	Metabotropic glutamate receptor 5	Show NN
3	CHEMBL4005 (https://www.ebi.ac.uk/chembl/target/inspect/CHEMBL4005)	PI3-kinase p110-alpha subunit	Show NN
4	CHEMBL217 (https://www.ebi.ac.uk/chembl/target/inspect/CHEMBL217)	Dopamine D2 receptor	Show NN
5	CHEMBL234 (https://www.ebi.ac.uk/chembl/target/inspect/CHEMBL234)	Dopamine D3 receptor	Show NN
6	CHEMBL2842 (https://www.ebi.ac.uk/chembl/target/inspect/CHEMBL2842)	Serine/threonine-protein kinase mTOR	Show NN
7	CHEMBL240 (https://www.ebi.ac.uk/chembl/target/inspect/CHEMBL240)	HERG	Show NN
8	CHEMBL224 (https://www.ebi.ac.uk/chembl/target/inspect/CHEMBL224)	Serotonin 2a (5-HT2a) receptor	Show NN
9	CHEMBL4409 (https://www.ebi.ac.uk/chembl/target/inspect/CHEMBL4409)	Phosphodiesterase 10A	Show NN
10	CHEMBL214 (https://www.ebi.ac.uk/chembl/target/inspect/CHEMBL214)	Serotonin 1a (5-HT1a) receptor	Show NN
11	CHEMBL3371 (https://www.ebi.ac.uk/chembl/target/inspect/CHEMBL3371)	Serotonin 6 (5-HT6) receptor	Show NN
12	CHEMBL339 (https://www.ebi.ac.uk/chembl/target/inspect/CHEMBL339)	Dopamine D2 receptor	Show NN
13	CHEMBL219 (https://www.ebi.ac.uk/chembl/target/inspect/CHEMBL219)	Dopamine D4 receptor	Show NN
14	CHEMBL225 (https://www.ebi.ac.uk/chembl/target/inspect/CHEMBL225)	Serotonin 2c (5-HT2c) receptor	Show NN
15	CHEMBL279 (https://www.ebi.ac.uk/chembl/target/inspect/CHEMBL279)	Vascular endothelial growth factor receptor 2	Show NN
16	CHEMBL273 (https://www.ebi.ac.uk/chembl/target/inspect/CHEMBL273)	Serotonin 1a (5-HT1a) receptor	Show NN
17	CHEMBL4657 (https://www.ebi.ac.uk/chembl/target/inspect/CHEMBL4657)	Dipeptidyl peptidase VIII	Show NN
18	CHEMBL5652 (https://www.ebi.ac.uk/chembl/target/inspect/CHEMBL5652)	Glucose-dependent insulinotropic receptor	Show NN

19	CHEMBL2564 (https://www.ebi.ac.uk/chembl/target/inspect/CHEMBL2564)	Metabotropic glutamate receptor 5	Show NN
20	CHEMBL4616 (https://www.ebi.ac.uk/chembl/target/inspect/CHEMBL4616)	Ghrelin receptor	Show NN
