# Supplementary material for: Accelerating Drug Discovery by Early Protein Drug Target Prediction Based on a Multi-Fingerprint Similarity Search
Source: Molecules. 2019 Jun 14;24(12):2233. doi: 10.3390/molecules24122233 (PMC6631269; doi:10.3390/molecules24122233)
Supplement: Supplementary file 1 [file molecules-24-02233-s001.zip › File_S2.zip!/File_S2/Target_Prediction_compound#25.pdf]

MuSSel not predicted

SwissTargetPrediction report:

Reference:
Gfeller D., Michielin O. & Zoete V.
Shaping the interaction landscape of bioactive molecules, *Bioinformatics* (2013) 29:3073-3079.

Query Molecule

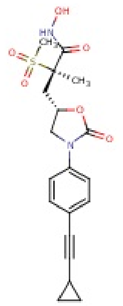

Frequency of Target Class

Target	Uniprot ID	Gene code	ChEMBL ID	Probability	# sim. cmpds (3D / 2D)	Target Class
22 kDa interstitial collagenase *(by homology)*	P03956	MMP1	CHEMBL332		334 / 9	Metallo Protease
PEX	P08253	MMP2	CHEMBL333		292 / 9	Metallo Protease
Stromelysin-1 *(by homology)*	P08254	MMP3	CHEMBL283		334 / 9	Metallo Protease
Stromelysin-2 *(by homology)*	P09238	MMP10	CHEMBL4270		334 / 9	Metallo Protease
67 kDa matrix metalloproteinase-9	P14780	MMP9	CHEMBL321		292 / 9	Metallo Protease
Neutrophil collagenase	P22894	MMP8	CHEMBL4588		287 / 2	Metallo Protease
Matrix metalloproteinase-27 *(by homology)*	Q9H306	MMP27			334 / 9	Metallo Protease
Macrophage metalloelastase *(by homology)*	P39900	MMP12	CHEMBL4393		295 / 9	Metallo Protease
Collagenase 3	P45452	MMP13	CHEMBL280		296 / 9	Metallo Protease
Matrix metalloproteinase-20 *(by homology)*	O60882	MMP20	CHEMBL1938226		185 / 9	Enzyme
Neuronal acetylcholine receptor subunit alpha-7 *(by homology)*	P36544	CHRNA7	CHEMBL2492		11 / 10	Ion channel
CHRNA7-FAM7A fusion protein *(by homology)*	Q494W8	CHRFAM7A			11 / 10	Ion channel
Metabotropic glutamate receptor 3 *(by homology)*	Q14832	GRM3	CHEMBL2888		1 / 29	Membrane receptor
Muscarinic acetylcholine receptor M2	P08172	CHRM2	CHEMBL211		11 / 6	Membrane receptor
Muscarinic acetylcholine receptor M4 *(by homology)*	P08173	CHRM4	CHEMBL1821		12 / 6	Membrane receptor

Polypharmacology Browser 2 not predicted
