# Supplementary material for: Accelerating Drug Discovery by Early Protein Drug Target Prediction Based on a Multi-Fingerprint Similarity Search
Source: Molecules. 2019 Jun 14;24(12):2233. doi: 10.3390/molecules24122233 (PMC6631269; doi:10.3390/molecules24122233)
Supplement: Supplementary file 1 [file molecules-24-02233-s001.zip › File_S2.zip!/File_S2/Target_Prediction_compound#26.pdf]

MuSSel not predicted

SwissTargetPrediction report:

Reference:
Gfeller D., Michielin O. & Zoete V.
Shaping the interaction landscape of bioactive molecules, *Bioinformatics* (2013) 29:3073-3079.

Query Molecule

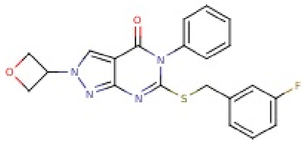

Frequency of Target Class

Target	Uniprot ID	Gene code	ChEMBL ID	Probability	# sim. cmpds (3D / 2D)	Target Class
Mitogen-activated protein kinase 8 *(by homology)*	P45983	MAPK8	CHEMBL2276		312 / 0	Ser_Thr Kinase
Mitogen-activated protein kinase 9 *(by homology)*	P45984	MAPK9	CHEMBL4179		312 / 0	Ser_Thr Kinase
Mitogen-activated protein kinase 10 *(by homology)*	P53779	MAPK10	CHEMBL2637		312 / 0	Ser_Thr Kinase
Mitogen-activated protein kinase 11 *(by homology)*	Q15759	MAPK11	CHEMBL3961		314 / 0	Ser_Thr Kinase
Mitogen-activated protein kinase 14	Q16539	MAPK14	CHEMBL260		314 / 0	Ser_Thr Kinase
Phosphatidylinositol 4,5-bisphosphate 3-kinase catalytic subunit delta isoform	O00329	PIK3CD	CHEMBL3130		386 / 0	Enzyme
Phosphatidylinositol 4,5-bisphosphate 3-kinase catalytic subunit alpha isoform *(by homology)*	P42336	PIK3CA	CHEMBL4005		388 / 0	Enzyme
Phosphatidylinositol 4,5-bisphosphate 3-kinase catalytic subunit beta isoform	P42338	PIK3CB	CHEMBL3145		386 / 0	Enzyme
DNA-dependent protein kinase catalytic subunit	P78527	PRKDC	CHEMBL3142		158 / 0	Ser_Thr Kinase
Muscleblind-like protein 1	Q9NR56	MBNL1	CHEMBL1293317		491 / 0	Unclassified
Muscleblind-like protein 2 *(by homology)*	Q5VZF2	MBNL2			491 / 0	Unclassified
Muscleblind-like protein 3 *(by homology)*	Q9NUK0	MBNL3			491 / 0	Unclassified
Microtubule-associated protein tau	P10636	MAPT	CHEMBL1293224		554 / 0	Unclassified

tau						
Neuropeptide Y receptor type 5	Q15761	NPY5R	CHEMBL4561		60 / 0	Membrane receptor
Complex	P30281/P24385/P11802/P30279	CCND3/CCND1/CDK4/CCND2	CHEMBL2095942		10 / 0	Cytosolic other /Ser_Thr Kinase

Polypharmacology Browser 2 Prediction:

Targets predicted using NN(ECfp4) + NB(ECfp4). Save Table

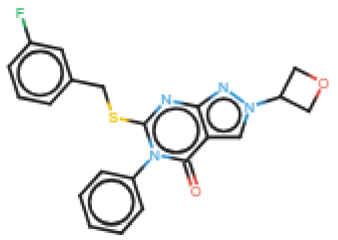

Query molecule

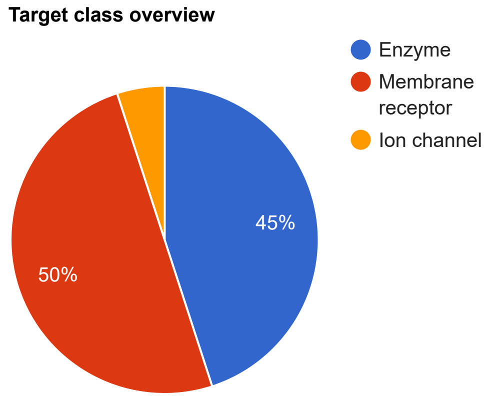

Rank	ChEMBL ID	Common name	Nearest neighbours
1	CHEMBL217 (https://www.ebi.ac.uk/chembl/target/inspect/CHEMBL217)	Dopamine D2 receptor	Show NN
2	CHEMBL3717 (https://www.ebi.ac.uk/chembl/target/inspect/CHEMBL3717)	Hepatocyte growth factor receptor	Show NN
3	CHEMBL240 (https://www.ebi.ac.uk/chembl/target/inspect/CHEMBL240)	HERG	Show NN
4	CHEMBL251 (https://www.ebi.ac.uk/chembl/target/inspect/CHEMBL251)	Adenosine A2a receptor	Show NN
5	CHEMBL234 (https://www.ebi.ac.uk/chembl/target/inspect/CHEMBL234)	Dopamine D3 receptor	Show NN
6	CHEMBL224 (https://www.ebi.ac.uk/chembl/target/inspect/CHEMBL224)	Serotonin 2a (5-HT2a) receptor	Show NN
7	CHEMBL4005 (https://www.ebi.ac.uk/chembl/target/inspect/CHEMBL4005)	PI3-kinase p110-alpha subunit	Show NN
8	CHEMBL3130 (https://www.ebi.ac.uk/chembl/target/inspect/CHEMBL3130)	PI3-kinase p110-delta subunit	Show NN
9	CHEMBL344 (https://www.ebi.ac.uk/chembl/target/inspect/CHEMBL344)	Melanin-concentrating hormone receptor 1	Show NN
10	CHEMBL226 (https://www.ebi.ac.uk/chembl/target/inspect/CHEMBL226)	Adenosine A1 receptor	Show NN
11	CHEMBL260 (https://www.ebi.ac.uk/chembl/target/inspect/CHEMBL260)	MAP kinase p38 alpha	Show NN
12	CHEMBL4409 (https://www.ebi.ac.uk/chembl/target/inspect/CHEMBL4409)	Phosphodiesterase 10A	Show NN
13	CHEMBL204 (https://www.ebi.ac.uk/chembl/target/inspect/CHEMBL204)	Thrombin	Show NN
14	CHEMBL3267 (https://www.ebi.ac.uk/chembl/target/inspect/CHEMBL3267)	PI3-kinase p110-gamma subunit	Show NN
15	CHEMBL225 (https://www.ebi.ac.uk/chembl/target/inspect/CHEMBL225)	Serotonin 2c (5-HT2c) receptor	Show NN
16	CHEMBL5071 (https://www.ebi.ac.uk/chembl/target/inspect/CHEMBL5071)	G protein-coupled receptor 44	Show NN
17	CHEMBL279 (https://www.ebi.ac.uk/chembl/target/inspect/CHEMBL279)	Vascular endothelial growth factor receptor 2	Show NN
18	CHEMBL287 (https://www.ebi.ac.uk/chembl/target/inspect/CHEMBL287)	Sigma opioid receptor	Show NN

19	CHEMBL214 (https://www.ebi.ac.uk/chembl/target/inspect/CHEMBL214)	Serotonin 1a (5-HT1a) receptor	Show NN
20	CHEMBL4282 (https://www.ebi.ac.uk/chembl/target/inspect/CHEMBL4282)	Serine/threonine-protein kinase AKT	Show NN
