# Supplementary material for: Accelerating Drug Discovery by Early Protein Drug Target Prediction Based on a Multi-Fingerprint Similarity Search
Source: Molecules. 2019 Jun 14;24(12):2233. doi: 10.3390/molecules24122233 (PMC6631269; doi:10.3390/molecules24122233)
Supplement: Supplementary file 1 [file molecules-24-02233-s001.zip › File_S2.zip!/File_S2/Target_Prediction_compound#27.pdf]

MuSSel not predicted

SwissTargetPrediction report:

Reference:
Gfeller D., Michielin O. & Zoete V.
Shaping the interaction landscape of bioactive molecules, *Bioinformatics* (2013) 29:3073-3079.

Query Molecule

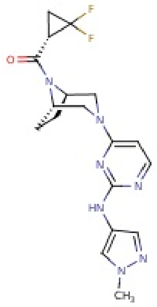

Frequency of Target Class

Target	Uniprot ID	Gene code	ChEMBL ID	Probability	# sim. cmpds (3D / 2D)	Target Class
Aurora kinase A	O14965	AURKA	CHEMBL4722		103 / 36	Ser_Thr Kinase
Aurora kinase B	Q96GD4	AURKB	CHEMBL2185		103 / 36	Ser_Thr Kinase
Aurora kinase C *(by homology)*	Q9UQB9	AURKC	CHEMBL3935		103 / 36	Ser_Thr Kinase
Cyclin-dependent kinase 1 *(by homology)*	P06493	CDK1	CHEMBL308		143 / 4	Ser_Thr Kinase
Cyclin-dependent kinase 4	P11802	CDK4	CHEMBL331		138 / 4	Ser_Thr Kinase
Cyclin-dependent kinase 2	P24941	CDK2	CHEMBL301		143 / 4	Ser_Thr Kinase
Cyclin-dependent kinase 3 *(by homology)*	Q00526	CDK3	CHEMBL4442		143 / 4	Ser_Thr Kinase
Cyclin-dependent kinase 6 *(by homology)*	Q00534	CDK6	CHEMBL2508		138 / 4	Ser_Thr Kinase
Vascular endothelial growth factor receptor 1 *(by homology)*	P17948	FLT1	CHEMBL1868		168 / 30	Tyr Kinase
Vascular endothelial growth factor receptor 3 *(by homology)*	P35916	FLT4	CHEMBL1955		168 / 30	Tyr Kinase
Vascular endothelial growth factor receptor 2	P35968	KDR	CHEMBL279		168 / 30	Tyr Kinase
Inhibitor of nuclear factor kappa-B kinase subunit epsilon	Q14164	IKBKE	CHEMBL3529		8 / 21	Ser_Thr Kinase
Serine/threonine-protein kinase TBK1	Q9UHD2	TBK1	CHEMBL5408		8 / 21	Ser_Thr Kinase
MAP/microtubule affinity-regulating kinase 3	P27448	MARK3	CHEMBL5600		4 / 1	Ser_Thr Kinase
Serine/threonine-protein kinase MARK2 *(by homology)*	Q7KZI7	MARK2	CHEMBL3831		4 / 1	Ser_Thr Kinase

Polypharmacology Browser 2 Prediction:

Targets predicted using NN(ECfp4) + NB(ECfp4). Save Table

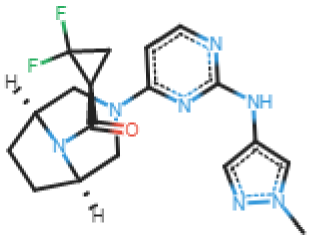

Query molecule

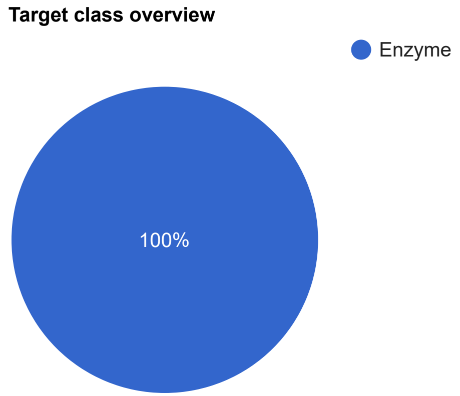

Rank	ChEMBL ID	Common name	Nearest neighbours
1	CHEMBL279 (https://www.ebi.ac.uk/chembl/target/inspect/CHEMBL279)	Vascular endothelial growth factor receptor 2	Show NN
2	CHEMBL1957 (https://www.ebi.ac.uk/chembl/target/inspect/CHEMBL1957)	Insulin-like growth factor I receptor	Show NN
3	CHEMBL4247 (https://www.ebi.ac.uk/chembl/target/inspect/CHEMBL4247)	ALK tyrosine kinase receptor	Show NN
4	CHEMBL2185 (https://www.ebi.ac.uk/chembl/target/inspect/CHEMBL2185)	Serine/threonine-protein kinase Aurora-B	Show NN
5	CHEMBL2637 (https://www.ebi.ac.uk/chembl/target/inspect/CHEMBL2637)	c-Jun N-terminal kinase 3	Show NN
6	CHEMBL262 (https://www.ebi.ac.uk/chembl/target/inspect/CHEMBL262)	Glycogen synthase kinase-3 beta	Show NN
7	CHEMBL2147 (https://www.ebi.ac.uk/chembl/target/inspect/CHEMBL2147)	Serine/threonine-protein kinase PIM1	Show NN
8	CHEMBL301 (https://www.ebi.ac.uk/chembl/target/inspect/CHEMBL301)	Cyclin-dependent kinase 2	Show NN
9	CHEMBL2148 (https://www.ebi.ac.uk/chembl/target/inspect/CHEMBL2148)	Tyrosine-protein kinase JAK3	Show NN
10	CHEMBL4005 (https://www.ebi.ac.uk/chembl/target/inspect/CHEMBL4005)	PI3-kinase p110-alpha subunit	Show NN
11	CHEMBL2842 (https://www.ebi.ac.uk/chembl/target/inspect/CHEMBL2842)	Serine/threonine-protein kinase mTOR	Show NN
12	CHEMBL3778 (https://www.ebi.ac.uk/chembl/target/inspect/CHEMBL3778)	Interleukin-1 receptor-associated kinase 4	Show NN
13	CHEMBL2835 (https://www.ebi.ac.uk/chembl/target/inspect/CHEMBL2835)	Tyrosine-protein kinase JAK1	Show NN
14	CHEMBL1862 (https://www.ebi.ac.uk/chembl/target/inspect/CHEMBL1862)	Tyrosine-protein kinase ABL	Show NN
15	CHEMBL2971 (https://www.ebi.ac.uk/chembl/target/inspect/CHEMBL2971)	Tyrosine-protein kinase JAK2	Show NN
16	CHEMBL3717 (https://www.ebi.ac.uk/chembl/target/inspect/CHEMBL3717)	Hepatocyte growth factor receptor	Show NN
17	CHEMBL4179 (https://www.ebi.ac.uk/chembl/target/inspect/CHEMBL4179)	c-Jun N-terminal kinase 2	Show NN
18	CHEMBL1974 (https://www.ebi.ac.uk/chembl/target/inspect/CHEMBL1974)	Tyrosine-protein kinase receptor FLT3	Show NN

19	CHEMBL2276 (https://www.ebi.ac.uk/chembl/target/inspect/CHEMBL2276)	c-Jun N-terminal kinase 1	Show NN
20	CHEMBL4722 (https://www.ebi.ac.uk/chembl/target/inspect/CHEMBL4722)	Serine/threonine-protein kinase Aurora-A	Show NN
