# Supplementary material for: Accelerating Drug Discovery by Early Protein Drug Target Prediction Based on a Multi-Fingerprint Similarity Search
Source: Molecules. 2019 Jun 14;24(12):2233. doi: 10.3390/molecules24122233 (PMC6631269; doi:10.3390/molecules24122233)
Supplement: Supplementary file 1 [file molecules-24-02233-s001.zip › File_S2.zip!/File_S2/Target_Prediction_compound#28.pdf]

MuSSel not predicted

SwissTargetPrediction report:

Reference:
Gfeller D., Michielin O. & Zoete V.
Shaping the interaction landscape of bioactive molecules, *Bioinformatics* (2013) 29:3073-3079.

Query Molecule

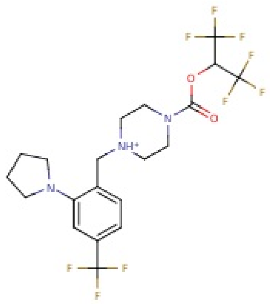

Frequency of Target Class

Target	Uniprot ID	Gene code	ChEMBL ID	Probability	# sim. cmpds (3D / 2D)	Target Class
Microtubule-associated protein tau	P10636	MAPT	CHEMBL1293224		15 / 16	Unclassified
Muscleblind-like protein 1	Q9NR56	MBNL1	CHEMBL1293317		16 / 27	Unclassified
Muscleblind-like protein 2 *(by homology)*	Q5VZF2	MBNL2			16 / 27	Unclassified
Muscleblind-like protein 3 *(by homology)*	Q9NUK0	MBNL3			16 / 27	Unclassified
Sodium- and chloride-dependent glycine transporter 1	P48067	SLC6A9	CHEMBL2337		6 / 34	Transporter
Sodium- and chloride-dependent glycine transporter 2	Q9Y345	SLC6A5	CHEMBL3060		6 / 34	Transporter
Sodium-dependent proline transporter *(by homology)*	Q99884	SLC6A7			6 / 34	Transporter
Sodium- and chloride-dependent neutral and basic amino acid transporter B(0+) *(by homology)*	Q9UN76	SLC6A14			6 / 34	Transporter
Tyrosyl-DNA phosphodiesterase 1	Q9NUW8	TDP1	CHEMBL1075138		4 / 3	Enzyme
Caspase-3 subunit p12	P42574	CASP3	CHEMBL2334		5 / 2	Cysteine Protease
Caspase-7 subunit p20	P55210	CASP7	CHEMBL3468		5 / 2	Cysteine Protease
Caspase-6 subunit p18	P55212	CASP6	CHEMBL3308		5 / 2	Cysteine Protease
Transient receptor potential cation channel subfamily V member 1	Q8NER1	TRPV1	CHEMBL4794		33 / 1	Ion channel
Cannabinoid receptor 1	P21554	CNR1	CHEMBL218		11 / 5	Membrane receptor
D(2) dopamine receptor *(by homology)*	P14416	DRD2	CHEMBL217		4 / 25	Membrane receptor

Polypharmacology Browser 2 Prediction:

Targets predicted using NN(ECfp4) + NB(ECfp4). Save Table

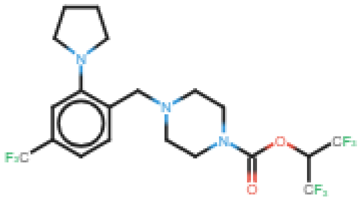

Query molecule

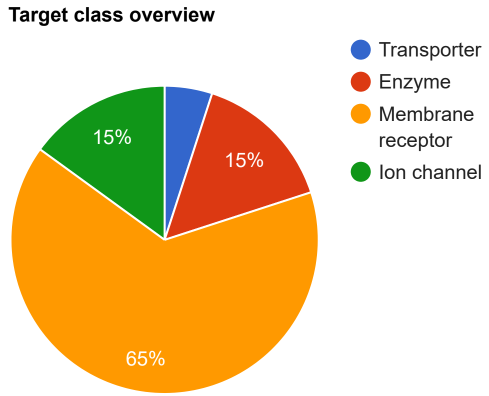

Rank	ChEMBL ID	Common name	Nearest neighbours
1	CHEMBL4794 (https://www.ebi.ac.uk/chembl/target/inspect/CHEMBL4794)	Vanilloid receptor	Show NN
2	CHEMBL240 (https://www.ebi.ac.uk/chembl/target/inspect/CHEMBL240)	HERG	Show NN
3	CHEMBL218 (https://www.ebi.ac.uk/chembl/target/inspect/CHEMBL218)	Cannabinoid CB1 receptor	Show NN
4	CHEMBL3229 (https://www.ebi.ac.uk/chembl/target/inspect/CHEMBL3229)	Anandamide amidohydrolase	Show NN
5	CHEMBL249 (https://www.ebi.ac.uk/chembl/target/inspect/CHEMBL249)	Neurokinin 1 receptor	Show NN
6	CHEMBL253 (https://www.ebi.ac.uk/chembl/target/inspect/CHEMBL253)	Cannabinoid CB2 receptor	Show NN
7	CHEMBL273 (https://www.ebi.ac.uk/chembl/target/inspect/CHEMBL273)	Serotonin 1a (5-HT1a) receptor	Show NN
8	CHEMBL284 (https://www.ebi.ac.uk/chembl/target/inspect/CHEMBL284)	Dipeptidyl peptidase IV	Show NN
9	CHEMBL264 (https://www.ebi.ac.uk/chembl/target/inspect/CHEMBL264)	Histamine H3 receptor	Show NN
10	CHEMBL344 (https://www.ebi.ac.uk/chembl/target/inspect/CHEMBL344)	Melanin-concentrating hormone receptor 1	Show NN
11	CHEMBL259 (https://www.ebi.ac.uk/chembl/target/inspect/CHEMBL259)	Melanocortin receptor 4	Show NN
12	CHEMBL3572 (https://www.ebi.ac.uk/chembl/target/inspect/CHEMBL3572)	Cholesteryl ester transfer protein	Show NN
13	CHEMBL2337 (https://www.ebi.ac.uk/chembl/target/inspect/CHEMBL2337)	Glycine transporter 1	Show NN
14	CHEMBL339 (https://www.ebi.ac.uk/chembl/target/inspect/CHEMBL339)	Dopamine D2 receptor	Show NN
15	CHEMBL340 (https://www.ebi.ac.uk/chembl/target/inspect/CHEMBL340)	Cytochrome P450 3A4	Show NN
16	CHEMBL4018 (https://www.ebi.ac.uk/chembl/target/inspect/CHEMBL4018)	Neuropeptide Y receptor type 2	Show NN
17	CHEMBL217 (https://www.ebi.ac.uk/chembl/target/inspect/CHEMBL217)	Dopamine D2 receptor	Show NN
18	CHEMBL224 (https://www.ebi.ac.uk/chembl/target/inspect/CHEMBL224)	Serotonin 2a (5-HT2a) receptor	Show NN

19	CHEMBL225 (https://www.ebi.ac.uk/chembl/target/inspect/CHEMBL225)	Serotonin 2c (5-HT2c) receptor	Show NN
20	CHEMBL5071 (https://www.ebi.ac.uk/chembl/target/inspect/CHEMBL5071)	G protein-coupled receptor 44	Show NN
