# Supplementary material for: Accelerating Drug Discovery by Early Protein Drug Target Prediction Based on a Multi-Fingerprint Similarity Search
Source: Molecules. 2019 Jun 14;24(12):2233. doi: 10.3390/molecules24122233 (PMC6631269; doi:10.3390/molecules24122233)
Supplement: Supplementary file 1 [file molecules-24-02233-s001.zip › File_S2.zip!/File_S2/Target_Prediction_compound#29.pdf]

MuSSel not predicted

SwissTargetPrediction report:

Reference:
Gfeller D., Michielin O. & Zoete V.
Shaping the interaction landscape of bioactive molecules, *Bioinformatics* (2013) 29:3073-3079.

Query Molecule

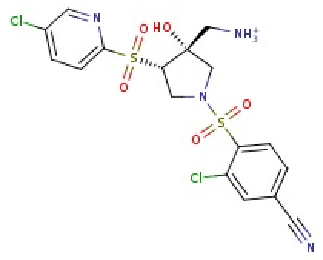

Frequency of Target Class

Target	Uniprot ID	Gene code	ChEMBL ID	Probability	# sim. cmpds (3D / 2D)	Target Class
Microtubule-associated protein tau	P10636	MAPT	CHEMBL1293224		201 / 2	Unclassified
Coagulation factor IXa heavy chain *(by homology)*	P00740	F9	CHEMBL2016		34 / 1	Serine Protease
Factor X light chain	P00742	F10	CHEMBL244		34 / 1	Serine Protease
Coagulation factor VII *(by homology)*	P08709	F7	CHEMBL3991		34 / 1	Serine Protease
Methionine aminopeptidase 2	P50579	METAP2	CHEMBL3922		82 / 0	Metallo Protease
Dual specificity mitogen-activated protein kinase kinase 2 *(by homology)*	P36507	MAP2K2	CHEMBL2964		102 / 0	Ser_Thr_Tyr Kinase
Dual specificity mitogen-activated protein kinase kinase 1	Q02750	MAP2K1	CHEMBL3587		102 / 0	Ser_Thr_Tyr Kinase
Dual specificity mitogen-activated protein kinase kinase 5 *(by homology)*	Q13163	MAP2K5	CHEMBL4948		104 / 0	Ser_Thr_Tyr Kinase
Vascular endothelial growth factor receptor 1 *(by homology)*	P17948	FLT1	CHEMBL1868		114 / 0	Tyr Kinase
Vascular endothelial growth factor receptor 3 *(by homology)*	P35916	FLT4	CHEMBL1955		114 / 0	Tyr Kinase
Vascular endothelial growth factor receptor 2	P35968	KDR	CHEMBL279		114 / 0	Tyr Kinase
Methionine aminopeptidase 1	P53582	METAP1	CHEMBL2474		15 / 0	Metallo Protease
Methionine aminopeptidase 1D, mitochondrial *(by homology)*	Q6UB28	METAP1D			15 / 0	Metallo Protease
Beta-2 adrenergic receptor *(by homology)*	P07550	ADRB2	CHEMBL210		66 / 0	Membrane receptor
Beta-1 adrenergic receptor	P08588	ADRB1	CHEMBL213		61 / 0	Membrane receptor

Polypharmacology Browser 2 not predicted
