# Supplementary material for: Accelerating Drug Discovery by Early Protein Drug Target Prediction Based on a Multi-Fingerprint Similarity Search
Source: Molecules. 2019 Jun 14;24(12):2233. doi: 10.3390/molecules24122233 (PMC6631269; doi:10.3390/molecules24122233)
Supplement: Supplementary file 1 [file molecules-24-02233-s001.zip › File_S2.zip!/File_S2/Target_Prediction_compound#3.pdf]

MuSSel Prediction k_i:

```
1 rank
Sigma-1 receptor : Cavia porcellus
     score: 6.867 on CHEMBL4153 based on 9 fingerprints
Fingerprint type         Ligand  Tanimoto Similarity  activity Valid fg
      FeatMFP1   CHEMBL244449             0.739130    175.00
          MFP1  CHEMBL1645207             0.600000      0.71        *
        RDKit7  CHEMBL1780001             0.741107    107.00
       Pattern  CHEMBL1780001             0.902857    107.00        *
       AP_bits   CHEMBL574235             0.564955     37.00        *
       TT_bits  CHEMBL1171865             0.677419      9.00        *
           FP2  CHEMBL1171865             0.699115      9.00
 hybridization  CHEMBL1779993             0.712644    538.00        *
  substructure  CHEMBL1645202             1.000000      0.59        *
         graph  CHEMBL1672034             0.698113     13.60
       pubchem  CHEMBL1645207             0.858333      0.71        *
     cdk_maccs  CHEMBL1645207             0.840000      0.71        *
  klekota_roth  CHEMBL1780001             0.710843    107.00        *
     *** ki ACTIVITY *** value prediction
     based on 9 locally validated fgps ---> 115.852
["TT_bits", "substructure", "Pattern", "MFP1", "klekota_roth", "pubchem",
"AP_bits", "cdk_maccs", "hybridization"]

2 rank
Sigma opioid receptor : Rattus norvegicus
     score: 4.465 on CHEMBL3602 based on 6 fingerprints
Fingerprint type         Ligand  Tanimoto Similarity  activity Valid fg
      FeatMFP1   CHEMBL281594             0.600000     116.0
          MFP1   CHEMBL144662             0.527778    8460.0
        RDKit7   CHEMBL344348             0.658977    4360.0
       Pattern   CHEMBL140030             0.917137    9890.0        *
       AP_bits  CHEMBL1162973             0.552553      13.6        *
       TT_bits   CHEMBL144662             0.552239    8460.0        *
           FP2   CHEMBL344348             0.616667    4360.0
 hybridization   CHEMBL344348             0.581921    4360.0
  substructure  CHEMBL2346998             0.937500      37.5        *
         graph  CHEMBL3740764             0.663636     614.0
       pubchem   CHEMBL344348             0.846774    4360.0
     cdk_maccs   CHEMBL366388             0.843137      30.0        *
  klekota_roth  CHEMBL1162973             0.662651      13.6        *
     *** ki ACTIVITY *** value prediction
     based on 6 locally validated fgps ---> 4135.774
["TT_bits", "substructure", "klekota_roth", "AP_bits", "Pattern",
"cdk_maccs"]

3 rank
Sigma opioid receptor : Homo sapiens
     score: 3.730 on CHEMBL287 based on 5 fingerprints
Fingerprint type         Ligand  Tanimoto Similarity  activity Valid fg
      FeatMFP1   CHEMBL312766             0.652174      0.52
          MFP1  CHEMBL1645202             0.542857      0.59
        RDKit7   CHEMBL344348             0.658977    148.00
       Pattern   CHEMBL140030             0.917137     95.30        *
       AP_bits    CHEMBL75608             0.581081      0.61        *
       TT_bits   CHEMBL144662             0.552239     12.70        *
           FP2   CHEMBL344348             0.616667    148.00
 hybridization   CHEMBL344348             0.581921    148.00
  substructure  CHEMBL1645202             1.000000      0.59        *
         graph    CHEMBL80968             0.676471     12.00
       pubchem   CHEMBL344348             0.846774    148.00
     cdk_maccs    CHEMBL61479             0.821429     17.00
```

```
   klekota_roth    CHEMBL75608             0.679487      0.61        *
      *** ki ACTIVITY *** value prediction
      based on 5 locally validated fgps ---> 36.244
["Pattern", "klekota_roth", "TT_bits", "substructure", "AP_bits"]

4 rank
Dopamine D2 receptor : Rattus norvegicus
      score: 2.943 on CHEMBL339 based on 4 fingerprints
Fingerprint type         Ligand  Tanimoto Similarity  activity Valid fg
       FeatMFP1    CHEMBL70540             0.652174  11993.00
           MFP1   CHEMBL127216             0.444444    194.98
         RDKit7   CHEMBL420972             0.555345   5540.00
        Pattern   CHEMBL391272             0.815789   1170.00
        AP_bits    CHEMBL77220             0.558824   3670.00        *
        TT_bits    CHEMBL75444             0.333333    479.00
            FP2   CHEMBL544099             0.578947   4610.00
  hybridization   CHEMBL420604             0.511568   4680.00
   substructure  CHEMBL1672302             0.875000      6.90        *
          graph   CHEMBL142668             0.707965    407.38
        pubchem    CHEMBL81330             0.786765    160.00
      cdk_maccs    CHEMBL81330             0.830189    160.00        *
   klekota_roth    CHEMBL75878             0.679012   4260.00        *
      *** ki ACTIVITY *** value prediction
      based on 4 locally validated fgps ---> 1323.175
["AP_bits", "klekota_roth", "substructure", "cdk_maccs"]

5 rank
Serotonin 1a (5-HT1a) receptor : Rattus norvegicus
      score: 2.933 on CHEMBL273 based on 4 fingerprints
Fingerprint type        Ligand  Tanimoto Similarity  activity Valid fg
       FeatMFP1  CHEMBL314997             0.600000    24.300
           MFP1  CHEMBL127216             0.444444     3.802
         RDKit7  CHEMBL542436             0.538182    54.100
        Pattern  CHEMBL543219             0.782759    37.000
        AP_bits   CHEMBL53325             0.552553    28.600        *
        TT_bits   CHEMBL28110             0.352113    64.000
            FP2  CHEMBL542436             0.578125    54.100
  hybridization  CHEMBL542436             0.490338    54.100
   substructure  CHEMBL281923             0.875000    17.000        *
          graph  CHEMBL485728             0.663462   183.000
        pubchem   CHEMBL81330             0.786765   120.000
      cdk_maccs  CHEMBL366388             0.843137    17.000        *
   klekota_roth   CHEMBL53325             0.662651    28.600        *
      *** ki ACTIVITY *** value prediction
      based on 4 locally validated fgps ---> 35.575
["AP_bits", "klekota_roth", "substructure", "cdk_maccs"]
```

SwissTargetPrediction report:

Reference:
Gfeller D., Michielin O. & Zoete V.
Shaping the interaction landscape of bioactive molecules, *Bioinformatics* (2013) 29:3073-3079.

Query Molecule

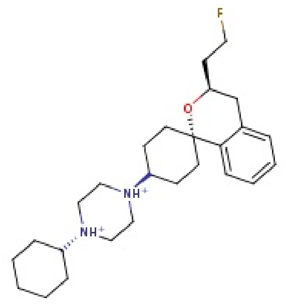

Frequency of Target Class

Target	Uniprot ID	Gene code	ChEMBL ID	Probability	# sim. cmpds (3D / 2D)	Target Class
Sigma non-opioid intracellular receptor 1	Q99720	SIGMAR1	CHEMBL287		205 / 73	Membrane receptor
Sodium-dependent noradrenaline transporter *(by homology)*	P23975	SLC6A2	CHEMBL222		508 / 227	Transporter
Sodium-dependent serotonin transporter *(by homology)*	P31645	SLC6A4	CHEMBL228		520 / 215	Transporter
Sodium-dependent dopamine transporter	Q01959	SLC6A3	CHEMBL238		508 / 227	Transporter
D(2) dopamine receptor *(by homology)*	P14416	DRD2	CHEMBL217		427 / 83	Membrane receptor
Sodium- and chloride-dependent glycine transporter 1 *(by homology)*	P48067	SLC6A9	CHEMBL2337		241 / 110	Transporter
Sodium- and chloride-dependent glycine transporter 2 *(by homology)*	Q9Y345	SLC6A5	CHEMBL3060		241 / 110	Transporter
Sodium-dependent proline transporter *(by homology)*	Q99884	SLC6A7			241 / 110	Transporter
Sodium- and chloride-dependent neutral and basic amino acid transporter B(0+) *(by homology)*	Q9UN76	SLC6A14			241 / 110	Transporter
D(3) dopamine receptor *(by homology)*	P35462	DRD3	CHEMBL234		280 / 26	Membrane receptor
D(4) dopamine receptor	P21917	DRD4	CHEMBL219		204 / 15	Membrane receptor
5-hydroxytryptamine receptor 2A *(by homology)*	P28223	HTR2A	CHEMBL224		187 / 36	Membrane receptor
5-hydroxytryptamine receptor 2C	P28335	HTR2C	CHEMBL225		135 / 31	Membrane receptor
5-hydroxytryptamine receptor 2B	P41595	HTR2B	CHEMBL1833		135 / 31	Membrane receptor
5-hydroxytryptamine receptor 1A	P08908	HTR1A	CHEMBL214		212 / 23	Membrane receptor

Polypharmacology Browser 2 Prediction:

Targets predicted using NN(ECfp4) + NB(ECfp4). Save Table

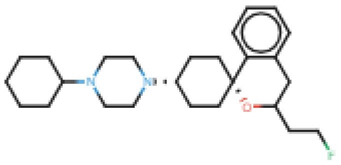

Query molecule

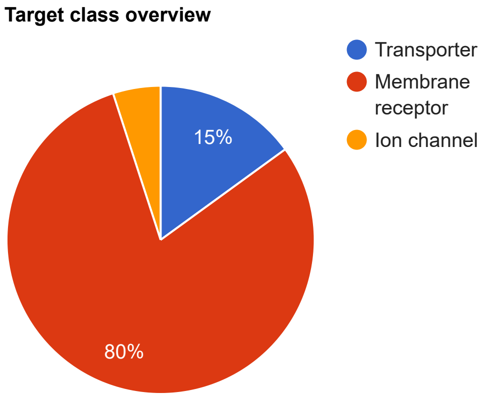

Rank	ChEMBL ID	Common name	Nearest neighbours
1	CHEMBL3602 (https://www.ebi.ac.uk/chembl/target/inspect/CHEMBL3602)	Sigma opioid receptor	Show NN
2	CHEMBL287 (https://www.ebi.ac.uk/chembl/target/inspect/CHEMBL287)	Sigma opioid receptor	Show NN
3	CHEMBL339 (https://www.ebi.ac.uk/chembl/target/inspect/CHEMBL339)	Dopamine D2 receptor	Show NN
4	CHEMBL217 (https://www.ebi.ac.uk/chembl/target/inspect/CHEMBL217)	Dopamine D2 receptor	Show NN
5	CHEMBL237 (https://www.ebi.ac.uk/chembl/target/inspect/CHEMBL237)	Kappa opioid receptor	Show NN
6	CHEMBL233 (https://www.ebi.ac.uk/chembl/target/inspect/CHEMBL233)	Mu opioid receptor	Show NN
7	CHEMBL264 (https://www.ebi.ac.uk/chembl/target/inspect/CHEMBL264)	Histamine H3 receptor	Show NN
8	CHEMBL240 (https://www.ebi.ac.uk/chembl/target/inspect/CHEMBL240)	HERG	Show NN
9	CHEMBL236 (https://www.ebi.ac.uk/chembl/target/inspect/CHEMBL236)	Delta opioid receptor	Show NN
10	CHEMBL214 (https://www.ebi.ac.uk/chembl/target/inspect/CHEMBL214)	Serotonin 1a (5-HT1a) receptor	Show NN
11	CHEMBL4767 (https://www.ebi.ac.uk/chembl/target/inspect/CHEMBL4767)	Vesicular acetylcholine transporter	Show NN
12	CHEMBL216 (https://www.ebi.ac.uk/chembl/target/inspect/CHEMBL216)	Muscarinic acetylcholine receptor M1	Show NN
13	CHEMBL273 (https://www.ebi.ac.uk/chembl/target/inspect/CHEMBL273)	Serotonin 1a (5-HT1a) receptor	Show NN
14	CHEMBL224 (https://www.ebi.ac.uk/chembl/target/inspect/CHEMBL224)	Serotonin 2a (5-HT2a) receptor	Show NN
15	CHEMBL2014 (https://www.ebi.ac.uk/chembl/target/inspect/CHEMBL2014)	Nociceptin receptor	Show NN
16	CHEMBL228 (https://www.ebi.ac.uk/chembl/target/inspect/CHEMBL228)	Serotonin transporter	Show NN
17	CHEMBL211 (https://www.ebi.ac.uk/chembl/target/inspect/CHEMBL211)	Muscarinic acetylcholine receptor M2	Show NN
18	CHEMBL245 (https://www.ebi.ac.uk/chembl/target/inspect/CHEMBL245)	Muscarinic acetylcholine receptor M3	Show NN

19	CHEMBL313 (https://www.ebi.ac.uk/chembl/target/inspect/CHEMBL313)	Serotonin transporter	Show NN
20	CHEMBL234 (https://www.ebi.ac.uk/chembl/target/inspect/CHEMBL234)	Dopamine D3 receptor	Show NN
