# Supplementary material for: Accelerating Drug Discovery by Early Protein Drug Target Prediction Based on a Multi-Fingerprint Similarity Search
Source: Molecules. 2019 Jun 14;24(12):2233. doi: 10.3390/molecules24122233 (PMC6631269; doi:10.3390/molecules24122233)
Supplement: Supplementary file 1 [file molecules-24-02233-s001.zip › File_S2.zip!/File_S2/Target_Prediction_compound#30.pdf]

MuSSel not predicted

SwissTargetPrediction report:

Reference:
Gfeller D., Michielin O. & Zoete V.
Shaping the interaction landscape of bioactive molecules, *Bioinformatics* (2013) 29:3073-3079.

Query Molecule

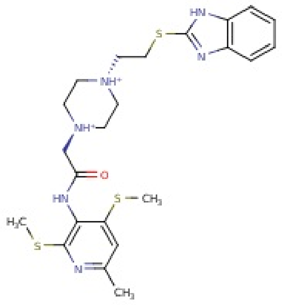

Frequency of Target Class

Target	Uniprot ID	Gene code	ChEMBL ID	Probability	# sim. cmpds (3D / 2D)	Target Class
Microtubule-associated protein tau	P10636	MAPT	CHEMBL1293224		1150 / 13	Unclassified
Muscleblind-like protein 1	Q9NR56	MBNL1	CHEMBL1293317		504 / 10	Unclassified
Muscleblind-like protein 2 *(by homology)*	Q5VZF2	MBNL2			504 / 10	Unclassified
Muscleblind-like protein 3 *(by homology)*	Q9NUK0	MBNL3			504 / 10	Unclassified
Signal transducer and activator of transcription 3	P40763	STAT3	CHEMBL4026		11 / 1	Transcription Factor
Signal transducer and activator of transcription 1-alpha/beta *(by homology)*	P42224	STAT1	CHEMBL6101		11 / 1	Unclassified
Signal transducer and activator of transcription 2 *(by homology)*	P52630	STAT2			11 / 1	Transcription Factor
Signal transducer and activator of transcription 4 *(by homology)*	Q14765	STAT4			11 / 1	Transcription Factor
Orexin receptor type 1	O43613	HCRTR1	CHEMBL5113		29 / 12	Membrane receptor
Orexin receptor type 2	O43614	HCRTR2	CHEMBL4792		29 / 12	Membrane receptor
D(4) dopamine receptor	P21917	DRD4	CHEMBL219		721 / 2	Membrane receptor
D(2) dopamine receptor	P14416	DRD2	CHEMBL217		1865 / 1	Membrane receptor
D(3) dopamine receptor	P35462	DRD3	CHEMBL234		1124 / 1	Membrane receptor
E3 ubiquitin-protein ligase XIAP	P98170	XIAP	CHEMBL4198		212 / 2	Cytosolic other
Baculoviral IAP repeat-containing protein 3 *(by homology)*	Q13489	BIRC3	CHEMBL5335		213 / 2	Enzyme

Polypharmacology Browser 2 Prediction:

Targets predicted using NN(ECfp4) + NB(ECfp4). Save Table

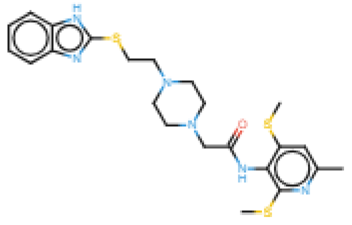

Query molecule

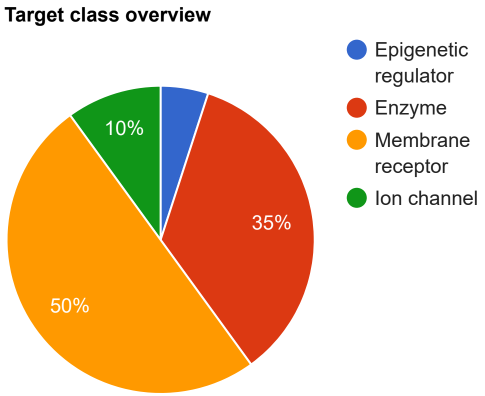

Rank	ChEMBL ID	Common name	Nearest neighbours
1	CHEMBL219 (https://www.ebi.ac.uk/chembl/target/inspect/CHEMBL219)	Dopamine D4 receptor	Show NN
2	CHEMBL240 (https://www.ebi.ac.uk/chembl/target/inspect/CHEMBL240)	HERG	Show NN
3	CHEMBL217 (https://www.ebi.ac.uk/chembl/target/inspect/CHEMBL217)	Dopamine D2 receptor	Show NN
4	CHEMBL273 (https://www.ebi.ac.uk/chembl/target/inspect/CHEMBL273)	Serotonin 1a (5-HT1a) receptor	Show NN
5	CHEMBL279 (https://www.ebi.ac.uk/chembl/target/inspect/CHEMBL279)	Vascular endothelial growth factor receptor 2	Show NN
6	CHEMBL325 (https://www.ebi.ac.uk/chembl/target/inspect/CHEMBL325)	Histone deacetylase 1	Show NN
7	CHEMBL4641 (https://www.ebi.ac.uk/chembl/target/inspect/CHEMBL4641)	Voltage-gated T-type calcium channel alpha-1G subunit	Show NN
8	CHEMBL1868 (https://www.ebi.ac.uk/chembl/target/inspect/CHEMBL1868)	Vascular endothelial growth factor receptor 1	Show NN
9	CHEMBL251 (https://www.ebi.ac.uk/chembl/target/inspect/CHEMBL251)	Adenosine A2a receptor	Show NN
10	CHEMBL249 (https://www.ebi.ac.uk/chembl/target/inspect/CHEMBL249)	Neurokinin 1 receptor	Show NN
11	CHEMBL234 (https://www.ebi.ac.uk/chembl/target/inspect/CHEMBL234)	Dopamine D3 receptor	Show NN
12	CHEMBL4722 (https://www.ebi.ac.uk/chembl/target/inspect/CHEMBL4722)	Serine/threonine-protein kinase Aurora-A	Show NN
13	CHEMBL2185 (https://www.ebi.ac.uk/chembl/target/inspect/CHEMBL2185)	Serine/threonine-protein kinase Aurora-B	Show NN
14	CHEMBL3371 (https://www.ebi.ac.uk/chembl/target/inspect/CHEMBL3371)	Serotonin 6 (5-HT6) receptor	Show NN
15	CHEMBL1833 (https://www.ebi.ac.uk/chembl/target/inspect/CHEMBL1833)	Serotonin 2b (5-HT2b) receptor	Show NN

16	CHEMBL205 (https://www.ebi.ac.uk/chembl/target/inspect/CHEMBL205)	Carbonic anhydrase II	Show NN
17	CHEMBL261 (https://www.ebi.ac.uk/chembl/target/inspect/CHEMBL261)	Carbonic anhydrase I	Show NN
18	CHEMBL214 (https://www.ebi.ac.uk/chembl/target/inspect/CHEMBL214)	Serotonin 1a (5-HT1a) receptor	Show NN
19	CHEMBL233 (https://www.ebi.ac.uk/chembl/target/inspect/CHEMBL233)	Mu opioid receptor	Show NN
20	CHEMBL340 (https://www.ebi.ac.uk/chembl/target/inspect/CHEMBL340)	Cytochrome P450 3A4	Show NN
