# Supplementary material for: Accelerating Drug Discovery by Early Protein Drug Target Prediction Based on a Multi-Fingerprint Similarity Search
Source: Molecules. 2019 Jun 14;24(12):2233. doi: 10.3390/molecules24122233 (PMC6631269; doi:10.3390/molecules24122233)
Supplement: Supplementary file 1 [file molecules-24-02233-s001.zip › File_S2.zip!/File_S2/Target_Prediction_compound#31.pdf]

MuSSel not predicted

SwissTargetPrediction report:

Reference:
Gfeller D., Michielin O. & Zoete V.
Shaping the interaction landscape of bioactive molecules, *Bioinformatics* (2013) 29:3073-3079.

Query Molecule

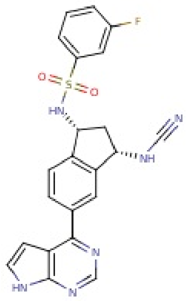

Frequency of Target Class

Target	Uniprot ID	Gene code	ChEMBL ID	Probability	# sim. cmpds (3D / 2D)	Target Class
B1 bradykinin receptor	P46663	BDKRB1	CHEMBL4308		68 / 6	Membrane receptor
Inhibitor of nuclear factor kappa-B kinase subunit beta	O14920	IKBKB	CHEMBL1991		138 / 11	Ser_Thr Kinase
Inhibitor of nuclear factor kappa-B kinase subunit alpha	O15111	CHUK	CHEMBL3476		138 / 11	Ser_Thr Kinase
Adenosine receptor A3	P33765	ADORA3	CHEMBL256		285 / 14	Membrane receptor
Adenosine receptor A2a	P29274	ADORA2A	CHEMBL251		498 / 23	Membrane receptor
Adenosine receptor A2b	P29275	ADORA2B	CHEMBL255		439 / 23	Membrane receptor
Adenosine receptor A1	P30542	ADORA1	CHEMBL226		615 / 25	Membrane receptor
Potassium voltage-gated channel subfamily H member 2	Q12809	KCNH2	CHEMBL240		162 / 1	Ion channel
Potassium voltage-gated channel subfamily H member 6 *(by homology)*	Q9H252	KCNH6			162 / 1	Ion channel
Potassium voltage-gated channel subfamily H member 7 *(by homology)*	Q9NS40	KCNH7			162 / 1	Ion channel
5-hydroxytryptamine receptor 2A *(by homology)*	P28223	HTR2A	CHEMBL224		270 / 1	Membrane receptor
Activation peptide fragment 1	P00734	F2	CHEMBL204		203 / 5	Serine Protease
Factor X light chain	P00742	F10	CHEMBL244		355 / 11	Serine Protease
Coagulation factor VII *(by homology)*	P08709	F7	CHEMBL3991		352 / 11	Serine Protease
Coagulation factor IXa heavy chain *(by homology)*	P00740	F9	CHEMBL2016		355 / 11	Serine Protease

Polypharmacology Browser 2 not predicted
