# Supplementary material for: Accelerating Drug Discovery by Early Protein Drug Target Prediction Based on a Multi-Fingerprint Similarity Search
Source: Molecules. 2019 Jun 14;24(12):2233. doi: 10.3390/molecules24122233 (PMC6631269; doi:10.3390/molecules24122233)
Supplement: Supplementary file 1 [file molecules-24-02233-s001.zip › File_S2.zip!/File_S2/Target_Prediction_compound#32.pdf]

MuSSel Prediction IC$_{50}$:

1 rank
Proteasome Macropain subunit MB1 : Homo sapiens
score: 8.696 on CHEMBL4662 based on 11 fingerprints

Fingerprint type	Ligand	Tanimoto Similarity	activity	Valid fg
FeatMFP1	CHEMBL3218825	0.761905	22.0	*
MFP1	CHEMBL3218825	0.757576	22.0	*
RDKit7	CHEMBL3218825	0.777778	22.0	*
Pattern	CHEMBL3218821	0.944056	25.0	*
AP_bits	CHEMBL3218825	0.651050	22.0	*
TT_bits	CHEMBL3218825	0.676923	22.0	*
FP2	CHEMBL3218825	0.754545	22.0	*
hybridization	CHEMBL3218825	0.677507	22.0	*
substructure	CHEMBL3218825	1.000000	22.0	*
graph	CHEMBL3218824	0.700000	5.4	
pubchem	CHEMBL3218825	0.904000	22.0	*
cdk_maccs	CHEMBL3218820	0.793103	50.0	
klekota_roth	CHEMBL3218841	0.790476	23.0	*

*** ic50 ACTIVITY *** value prediction
based on 11 locally validated fgps ---> 23.477
["TT_bits", "substructure", "Pattern", "FP2", "MFP1", "klekota_roth", "pubchem", "AP_bits", "FeatMFP1", "RDKit7", "hybridization"]

2 rank
Peptidase 1 : Dermatophagoides pteronyssinus
score: 7.327 on CHEMBL3351204 based on 10 fingerprints

Fingerprint type	Ligand	Tanimoto Similarity	activity	Valid fg
FeatMFP1	CHEMBL3679689	0.700000	7.85	*
MFP1	CHEMBL3360281	0.647059	9.00	*
RDKit7	CHEMBL3360284	0.682278	8.00	*
Pattern	CHEMBL3679689	0.939815	7.85	*
AP_bits	CHEMBL3360281	0.549834	9.00	*
TT_bits	CHEMBL3359775	0.514706	12.00	*
FP2	CHEMBL3360284	0.810526	8.00	*
hybridization	CHEMBL3360281	0.551913	9.00	
substructure	CHEMBL3359774	0.928571	9167.00	*
graph	CHEMBL3679690	0.678571	6.30	
pubchem	CHEMBL3360284	0.902439	8.00	*
cdk_maccs	CHEMBL3359775	0.696429	12.00	
klekota_roth	CHEMBL3360281	0.652174	9.00	*

*** ic50 ACTIVITY *** value prediction
based on 10 locally validated fgps ---> 466.790
["TT_bits", "substructure", "Pattern", "FP2", "MFP1", "klekota_roth", "pubchem", "AP_bits", "FeatMFP1", "RDKit7"]

3 rank
Cathepsin B : Homo sapiens
score: 7.228 on CHEMBL4072 based on 10 fingerprints

Fingerprint type	Ligand	Tanimoto Similarity	activity	Valid fg
FeatMFP1	CHEMBL3360294	0.681818	511.0	*
MFP1	CHEMBL3360281	0.647059	512.0	*
RDKit7	CHEMBL3360284	0.682278	242.0	*
Pattern	CHEMBL3360281	0.914414	512.0	*
AP_bits	CHEMBL3360281	0.549834	512.0	*
TT_bits	CHEMBL3359775	0.514706	50.0	*
FP2	CHEMBL3360284	0.810526	242.0	*
hybridization	CHEMBL3360281	0.551913	512.0	
substructure	CHEMBL3359776	0.866667	378.0	*
graph	CHEMBL3359780	0.676056	628.0	
pubchem	CHEMBL3360284	0.902439	242.0	*
cdk_maccs	CHEMBL3359775	0.696429	50.0	

```
   klekota_roth   CHEMBL133016             0.658120   59000.0        *
      *** ic50 ACTIVITY *** value prediction
      based on 10 locally validated fgps ---> 4293.936
["TT_bits", "substructure", "Pattern", "FP2", "MFP1", "klekota_roth",
"pubchem", "AP_bits", "FeatMFP1", "RDKit7"]

4 rank
ADAM17 : Homo sapiens
      score: 6.864 on CHEMBL3706 based on 9 fingerprints
Fingerprint type        Ligand  Tanimoto Similarity  activity Valid fg
       FeatMFP1  CHEMBL358770             0.809524      9.00        *
           MFP1  CHEMBL358770             0.540541      9.00        *
         RDKit7  CHEMBL358770             0.621590      9.00
        Pattern  CHEMBL144001             0.900452      0.57        *
        AP_bits  CHEMBL144206             0.566007      2.80        *
        TT_bits  CHEMBL314024             0.381579   2200.00
            FP2  CHEMBL358770             0.747664      9.00        *
  hybridization  CHEMBL358770             0.517045      9.00
   substructure  CHEMBL144169             0.928571      3.30        *
          graph   CHEMBL67311             0.711864   3592.00
        pubchem  CHEMBL358770             0.941176      9.00        *
      cdk_maccs  CHEMBL144001             0.816667      0.57        *
   klekota_roth   CHEMBL16520             0.613445     20.00        *
      *** ic50 ACTIVITY *** value prediction
      based on 9 locally validated fgps ---> 5.780
["substructure", "Pattern", "AP_bits", "MFP1", "pubchem", "FP2",
"FeatMFP1", "klekota_roth", "cdk_maccs"]

5 rank
Matrix metalloproteinase-2 : Homo sapiens
      score: 6.668 on CHEMBL333 based on 9 fingerprints
Fingerprint type         Ligand  Tanimoto Similarity  activity Valid fg
       FeatMFP1  CHEMBL1090647             0.761905      0.70        *
           MFP1    CHEMBL11306             0.571429      0.70        *
         RDKit7   CHEMBL347673             0.527950    100.00
        Pattern   CHEMBL152531             0.947368     15.00        *
        AP_bits   CHEMBL492599             0.569044    320.00        *
        TT_bits  CHEMBL3355723             0.465753     12.50
            FP2   CHEMBL347673             0.635514    100.00        *
  hybridization  CHEMBL3827934             0.569492    100.00
   substructure   CHEMBL196647             0.857143    197.00        *
          graph  CHEMBL3827831             0.773913      0.20        *
        pubchem  CHEMBL3355723             0.918033     12.50        *
      cdk_maccs   CHEMBL152267             0.793103     25.70
   klekota_roth   CHEMBL153914             0.634146      0.38        *
      *** ic50 ACTIVITY *** value prediction
      based on 9 locally validated fgps ---> 84.434
["substructure", "Pattern", "AP_bits", "MFP1", "pubchem", "graph", "FP2",
"FeatMFP1", "klekota_roth"]

6 rank
Matrix metalloproteinase-1 : Homo sapiens
      score: 6.140 on CHEMBL332 based on 8 fingerprints
Fingerprint type         Ligand  Tanimoto Similarity  activity Valid fg
       FeatMFP1  CHEMBL1090647             0.761905     196.0        *
           MFP1    CHEMBL96654             0.666667      30.0        *
         RDKit7   CHEMBL133303             0.529114     500.0
        Pattern   CHEMBL286698             0.925059     110.0        *
        AP_bits   CHEMBL162128             0.573668     488.0        *
        TT_bits    CHEMBL57551             0.483871     147.0
```

```
           FP2   CHEMBL144568            0.625954       2.2
  hybridization    CHEMBL96654           0.502732      30.0
   substructure   CHEMBL196647           0.857143   10000.0        *
          graph   CHEMBL315898           0.686957      26.0
        pubchem    CHEMBL19623           0.883333      92.0        *
      cdk_maccs    CHEMBL96654           0.839286      30.0        *
   klekota_roth    CHEMBL94155           0.633028    7000.0        *
      *** ic50 ACTIVITY *** value prediction
      based on 8 locally validated fgps ---> 2389.582
["substructure", "Pattern", "MFP1", "pubchem", "AP_bits", "FeatMFP1",
"klekota_roth", "cdk_maccs"]

7 rank
Matrix metalloproteinase 3 : Homo sapiens
      score: 5.922 on CHEMBL283 based on 8 fingerprints
Fingerprint type         Ligand  Tanimoto Similarity  activity Valid fg
       FeatMFP1   CHEMBL154040           0.761905    120.00        *
           MFP1    CHEMBL11306           0.571429     43.00        *
         RDKit7   CHEMBL133303           0.529114  10800.00
        Pattern   CHEMBL152531           0.947368     21.88        *
        AP_bits   CHEMBL162128           0.573668   2340.00        *
        TT_bits   CHEMBL133303           0.454545  10800.00
            FP2    CHEMBL74670           0.692982      9.10        *
  hybridization   CHEMBL154040           0.535714    120.00
   substructure  CHEMBL3617400           0.857143    450.00        *
          graph   CHEMBL420259           0.690476    665.00
        pubchem    CHEMBL19623           0.883333     17.00        *
      cdk_maccs   CHEMBL152267           0.793103    570.00
   klekota_roth   CHEMBL153914           0.634146      9.00        *
      *** ic50 ACTIVITY *** value prediction
      based on 8 locally validated fgps ---> 1553.075
["substructure", "Pattern", "AP_bits", "MFP1", "pubchem", "FP2",
"FeatMFP1", "klekota_roth"]

8 rank
Matrix metalloproteinase 9 : Homo sapiens
      score: 5.922 on CHEMBL321 based on 8 fingerprints
Fingerprint type         Ligand  Tanimoto Similarity  activity Valid fg
       FeatMFP1  CHEMBL1090647           0.761905      8.60        *
           MFP1    CHEMBL11306           0.571429      0.38        *
         RDKit7   CHEMBL162128           0.519016     49.00
        Pattern  CHEMBL3355723           0.933486     31.50        *
        AP_bits   CHEMBL162128           0.573668     49.00        *
        TT_bits    CHEMBL57551           0.483871     80.00
            FP2    CHEMBL74670           0.692982      4.30        *
  hybridization   CHEMBL117225           0.494624    840.00
   substructure   CHEMBL196647           0.857143    983.00        *
          graph   CHEMBL550961           0.717742      0.60
        pubchem  CHEMBL3355723           0.918033     31.50        *
      cdk_maccs  CHEMBL3355722           0.779661     19.50
   klekota_roth    CHEMBL16520           0.613445     12.00        *
      *** ic50 ACTIVITY *** value prediction
      based on 8 locally validated fgps ---> 91.630
["substructure", "Pattern", "AP_bits", "MFP1", "pubchem", "FP2",
"FeatMFP1", "klekota_roth"]

9 rank
Histone deacetylase 6 : Homo sapiens
      score: 5.861 on CHEMBL1865 based on 8 fingerprints
Fingerprint type         Ligand  Tanimoto Similarity  activity Valid fg
```

```
       FeatMFP1  CHEMBL2431863             0.842105      79.0        *
           MFP1  CHEMBL2431863             0.580645      79.0        *
         RDKit7  CHEMBL3356933             0.551867     139.0
        Pattern   CHEMBL227170             0.871332       9.0        *
        AP_bits   CHEMBL570041             0.547546    4900.0        *
        TT_bits  CHEMBL2333341             0.509804       7.0        *
            FP2   CHEMBL481718             0.678899     170.0        *
  hybridization  CHEMBL3356933             0.486486     139.0
   substructure  CHEMBL3309286             0.928571     130.0        *
          graph  CHEMBL3415628             0.741667      18.0
        pubchem   CHEMBL481718             0.901639     170.0        *
      cdk_maccs  CHEMBL3693774             0.716667       7.0
   klekota_roth   CHEMBL402363             0.554688   12000.0
      *** ic50 ACTIVITY *** value prediction
      based on 8 locally validated fgps ---> 4825.007
["TT_bits", "substructure", "Pattern", "AP_bits", "MFP1", "pubchem",
"FP2", "FeatMFP1"]

10 rank
Cathepsin L : Homo sapiens
      score: 5.781 on CHEMBL3837 based on 8 fingerprints
Fingerprint type        Ligand  Tanimoto Similarity  activity Valid fg
       FeatMFP1  CHEMBL123571             0.727273   4300.00        *
           MFP1  CHEMBL123571             0.567568   4300.00        *
         RDKit7  CHEMBL122513             0.582960     42.00
        Pattern  CHEMBL377285             0.864745    342.00        *
        AP_bits  CHEMBL258148             0.524922  34000.00        *
        TT_bits  CHEMBL550246             0.423729    251.19
            FP2  CHEMBL122513             0.572816     42.00
  hybridization  CHEMBL518284             0.463277     10.00
   substructure   CHEMBL51314             0.857143    500.00        *
          graph  CHEMBL123571             0.620438   4300.00
        pubchem  CHEMBL200884             0.842975     55.00        *
      cdk_maccs  CHEMBL123571             0.803571   4300.00        *
   klekota_roth  CHEMBL125553             0.593220     23.00        *
      *** ic50 ACTIVITY *** value prediction
      based on 8 locally validated fgps ---> 3324.267
["substructure", "Pattern", "MFP1", "pubchem", "AP_bits", "FeatMFP1",
"klekota_roth", "cdk_maccs"]

11 rank
Histone deacetylase 8 : Homo sapiens
      score: 5.740 on CHEMBL3192 based on 8 fingerprints
Fingerprint type         Ligand  Tanimoto Similarity  activity Valid fg
       FeatMFP1  CHEMBL1083439             0.750000     608.0        *
           MFP1  CHEMBL2333341             0.551724    1030.0        *
         RDKit7  CHEMBL3356933             0.551867      23.0
        Pattern   CHEMBL227170             0.871332    2060.0        *
        AP_bits   CHEMBL572044             0.547619    9700.0        *
        TT_bits  CHEMBL2333341             0.509804    1030.0        *
            FP2   CHEMBL481718             0.678899    2340.0        *
  hybridization  CHEMBL3356933             0.486486      23.0
   substructure  CHEMBL3309286             0.928571      59.0        *
          graph  CHEMBL3415628             0.741667      76.0
        pubchem   CHEMBL481718             0.901639    2340.0        *
      cdk_maccs   CHEMBL589246             0.700000   12170.0
   klekota_roth   CHEMBL402363             0.554688    3700.0
      *** ic50 ACTIVITY *** value prediction
      based on 8 locally validated fgps ---> 1575.219
```

```
["TT_bits", "substructure", "Pattern", "AP_bits", "MFP1", "pubchem",
"FP2", "FeatMFP1"]

12 rank
Histone deacetylase 2 : Homo sapiens
      score: 5.727 on CHEMBL1937 based on 8 fingerprints
Fingerprint type          Ligand  Tanimoto Similarity  activity Valid fg
       FeatMFP1  CHEMBL3098687             0.750000     956.0         *
           MFP1  CHEMBL2333341             0.551724     381.0         *
         RDKit7  CHEMBL3356933             0.551867    1770.0
        Pattern   CHEMBL227170             0.871332     205.0         *
        AP_bits  CHEMBL2012815             0.535022     290.0         *
        TT_bits  CHEMBL2333341             0.509804     381.0         *
            FP2   CHEMBL481718             0.678899    5540.0         *
  hybridization  CHEMBL3356933             0.486486    1770.0
   substructure  CHEMBL3309286             0.928571     100.0         *
          graph   CHEMBL140525             0.714286     200.0
        pubchem   CHEMBL481718             0.901639    5540.0         *
      cdk_maccs  CHEMBL3309290             0.687500     100.0
   klekota_roth  CHEMBL2063396             0.563492    1400.0
      *** ic50 ACTIVITY *** value prediction
      based on 8 locally validated fgps ---> 1959.524
["TT_bits", "substructure", "Pattern", "AP_bits", "MFP1", "pubchem",
"FP2", "FeatMFP1"]

13 rank
Histone deacetylase 1 : Homo sapiens
      score: 5.398 on CHEMBL325 based on 7 fingerprints
Fingerprint type          Ligand  Tanimoto Similarity  activity Valid fg
       FeatMFP1   CHEMBL246391             0.842105      93.0         *
           MFP1   CHEMBL246391             0.656250      93.0         *
         RDKit7   CHEMBL235702             0.616519     560.0
        Pattern   CHEMBL227170             0.871332      36.9         *
        AP_bits   CHEMBL570041             0.547546     200.0         *
        TT_bits   CHEMBL246391             0.466667      93.0
            FP2   CHEMBL246391             0.637255      93.0         *
  hybridization   CHEMBL246391             0.557576      93.0
   substructure  CHEMBL3309286             0.928571      43.0         *
          graph  CHEMBL3415628             0.741667    6020.0
        pubchem   CHEMBL246391             0.915254      93.0         *
      cdk_maccs   CHEMBL246391             0.741379      93.0
   klekota_roth  CHEMBL2063396             0.563492     680.0
      *** ic50 ACTIVITY *** value prediction
      based on 7 locally validated fgps ---> 8910.543
["substructure", "Pattern", "AP_bits", "MFP1", "pubchem", "FP2",
"FeatMFP1"]

14 rank
Immunoglobulin epsilon Fc receptor : Homo sapiens
      score: 5.254 on CHEMBL2940 based on 7 fingerprints
Fingerprint type         Ligand  Tanimoto Similarity  activity Valid fg
       FeatMFP1   CHEMBL96654             0.761905    1900.0         *
           MFP1   CHEMBL96654             0.666667    1900.0         *
         RDKit7  CHEMBL420077             0.465278     200.0
        Pattern  CHEMBL325608             0.914352     210.0         *
        AP_bits  CHEMBL112228             0.556114    3700.0         *
        TT_bits   CHEMBL94155             0.459016    5000.0
            FP2   CHEMBL94368             0.586207    1600.0
  hybridization   CHEMBL96654             0.502732    1900.0
   substructure   CHEMBL11306             0.714286    1000.0
```

```
          graph  CHEMBL330625             0.601449    5000.0
        pubchem   CHEMBL96654             0.882353    1900.0        *
      cdk_maccs   CHEMBL96654             0.839286    1900.0        *
   klekota_roth   CHEMBL94155             0.633028    5000.0        *
      *** ic50 ACTIVITY *** value prediction
      based on 7 locally validated fgps ---> 3785.606
["Pattern", "MFP1", "pubchem", "AP_bits", "FeatMFP1", "klekota_roth",
"cdk_maccs"]

15 rank
Aminopeptidase N : Sus scrofa
      score: 5.182 on CHEMBL2590 based on 7 fingerprints
Fingerprint type          Ligand  Tanimoto Similarity  activity Valid fg
       FeatMFP1  CHEMBL2036146             0.761905    4100.0        *
           MFP1  CHEMBL2036144             0.666667   21000.0        *
         RDKit7   CHEMBL597936             0.551646    6700.0
        Pattern   CHEMBL597332             0.855580    4300.0        *
        AP_bits   CHEMBL599356             0.512712   33100.0        *
        TT_bits   CHEMBL594961             0.473684   65100.0
            FP2   CHEMBL575029             0.601695    4300.0
  hybridization   CHEMBL594961             0.629508   65100.0
   substructure   CHEMBL593542             0.800000   36000.0
          graph   CHEMBL594848             0.651515   18500.0
        pubchem   CHEMBL594961             0.905983   65100.0        *
      cdk_maccs   CHEMBL594961             0.821429   65100.0        *
   klekota_roth   CHEMBL594961             0.657407   65100.0        *
      *** ic50 ACTIVITY *** value prediction
      based on 7 locally validated fgps ---> 34298.205
["Pattern", "MFP1", "pubchem", "AP_bits", "FeatMFP1", "klekota_roth",
"cdk_maccs"]

16 rank
Matrix metalloproteinase 14 : Homo sapiens
      score: 5.102 on CHEMBL3869 based on 7 fingerprints
Fingerprint type          Ligand  Tanimoto Similarity  activity Valid fg
       FeatMFP1  CHEMBL1090647             0.761905      34.0        *
           MFP1    CHEMBL20154             0.540541       1.9        *
         RDKit7   CHEMBL117225             0.456816    1900.0
        Pattern   CHEMBL117225             0.876333    1900.0        *
        AP_bits   CHEMBL492599             0.569044    1100.0        *
        TT_bits   CHEMBL267603             0.439024   80000.0
            FP2    CHEMBL19843             0.575221      56.0
  hybridization   CHEMBL117225             0.494624    1900.0
   substructure   CHEMBL196647             0.857143    1490.0        *
          graph  CHEMBL1392507             0.620968   26700.0
        pubchem    CHEMBL19623             0.883333     105.0        *
      cdk_maccs   CHEMBL117225             0.770492    1900.0
   klekota_roth    CHEMBL16520             0.613445       1.9        *
      *** ic50 ACTIVITY *** value prediction
      based on 7 locally validated fgps ---> 421.389
["substructure", "Pattern", "MFP1", "pubchem", "AP_bits", "FeatMFP1",
"klekota_roth"]

17 rank
Matrix metalloproteinase 13 : Homo sapiens
      score: 5.008 on CHEMBL280 based on 7 fingerprints
Fingerprint type          Ligand  Tanimoto Similarity  activity Valid fg
       FeatMFP1  CHEMBL2048512             0.681818      6.40        *
           MFP1  CHEMBL2048512             0.555556      6.40        *
         RDKit7   CHEMBL117225             0.456816     73.00
```

```
        Pattern   CHEMBL516206             0.920561      0.10         *
        AP_bits   CHEMBL250266             0.550960   2300.00         *
        TT_bits  CHEMBL1796289             0.372881  39810.72
            FP2   CHEMBL278366             0.552632   1590.00
  hybridization   CHEMBL117225             0.494624     73.00
   substructure  CHEMBL3617400             0.857143      4.60         *
          graph  CHEMBL2048512             0.652174      6.40
        pubchem   CHEMBL117225             0.830769     73.00         *
      cdk_maccs   CHEMBL117225             0.770492     73.00
   klekota_roth   CHEMBL117225             0.611111     73.00         *
      *** ic50 ACTIVITY *** value prediction
      based on 7 locally validated fgps ---> 272.868
["substructure", "Pattern", "MFP1", "pubchem", "AP_bits", "FeatMFP1",
"klekota_roth"]

18 rank
Neprilysin : Rattus norvegicus
      score: 4.979 on CHEMBL3369 based on 7 fingerprints
Fingerprint type        Ligand  Tanimoto Similarity  activity Valid fg
       FeatMFP1   CHEMBL16791             0.714286     600.0        *
           MFP1   CHEMBL16046             0.588235       2.0        *
         RDKit7  CHEMBL314626             0.418704    1270.0
        Pattern  CHEMBL358338             0.863341      28.0        *
        AP_bits   CHEMBL23598             0.503145     800.0        *
        TT_bits  CHEMBL146594             0.435484       3.0
            FP2   CHEMBL16975             0.642202       1.2        *
  hybridization   CHEMBL16975             0.500000       1.2
   substructure   CHEMBL16467             0.714286     800.0
          graph   CHEMBL16467             0.666667     800.0
        pubchem   CHEMBL16568             0.842975     750.0        *
      cdk_maccs   CHEMBL16568             0.824561     750.0        *
   klekota_roth   CHEMBL16975             0.547170       1.2
      *** ic50 ACTIVITY *** value prediction
      based on 7 locally validated fgps ---> 393.619
["Pattern", "AP_bits", "MFP1", "pubchem", "FP2", "FeatMFP1", "cdk_maccs"]
```

MuSSel Prediction k_i:

```
1 rank
Matrix metalloproteinase 8 : Homo sapiens
      score: 5.439 on CHEMBL4588 based on 7 fingerprints
Fingerprint type        Ligand  Tanimoto Similarity  activity Valid fg
       FeatMFP1  CHEMBL103480             0.800000     380.0        *
           MFP1   CHEMBL71800             0.647059       3.6        *
         RDKit7  CHEMBL324522             0.559840   55000.0
        Pattern   CHEMBL70406             0.941176      13.0        *
        AP_bits   CHEMBL70406             0.548061      13.0        *
        TT_bits   CHEMBL71800             0.477612       3.6
            FP2  CHEMBL324522             0.770000   55000.0        *
  hybridization  CHEMBL324522             0.555556   55000.0
   substructure  CHEMBL102373             0.846154    1100.0
          graph  CHEMBL324522             0.722689   55000.0
        pubchem  CHEMBL430838             0.890756      30.0        *
      cdk_maccs  CHEMBL117503             0.842105   25000.0        *
   klekota_roth  CHEMBL334065             0.657658   50000.0
      *** ki ACTIVITY *** value prediction
      based on 7 locally validated fgps ---> 9645.068
["Pattern", "AP_bits", "MFP1", "pubchem", "FP2", "FeatMFP1", "cdk_maccs"]

2 rank
Matrix metalloproteinase-2 : Homo sapiens
      score: 3.743 on CHEMBL333 based on 5 fingerprints
Fingerprint type        Ligand  Tanimoto Similarity  activity Valid fg
       FeatMFP1  CHEMBL415074             0.761905      0.10        *
           MFP1   CHEMBL72337             0.628571     18.00        *
         RDKit7  CHEMBL295798             0.471090     91.00
        Pattern   CHEMBL70406             0.941176     25.00        *
        AP_bits   CHEMBL70406             0.548061     25.00        *
        TT_bits   CHEMBL72337             0.455882     18.00
            FP2   CHEMBL77057             0.573913      0.02
  hybridization  CHEMBL308228             0.503958      1.90
   substructure   CHEMBL74040             0.733333      0.01
          graph   CHEMBL77057             0.682759      0.02
        pubchem   CHEMBL70406             0.862903     25.00        *
      cdk_maccs   CHEMBL72337             0.824561     18.00
   klekota_roth   CHEMBL72337             0.572581     18.00
      *** ki ACTIVITY *** value prediction
      based on 5 locally validated fgps ---> 15.267
["FeatMFP1", "Pattern", "MFP1", "pubchem", "AP_bits"]

3 rank
Matrix metalloproteinase 3 : Homo sapiens
      score: 3.130 on CHEMBL283 based on 4 fingerprints
Fingerprint type        Ligand  Tanimoto Similarity  activity Valid fg
       FeatMFP1  CHEMBL415074             0.761905      5.44        *
           MFP1  CHEMBL430838             0.583333     91.20
         RDKit7  CHEMBL112864             0.482465    120.00
        Pattern  CHEMBL269334             0.926773     15.00        *
        AP_bits  CHEMBL112864             0.550544    120.00        *
        TT_bits  CHEMBL430838             0.439394     91.20
            FP2   CHEMBL77057             0.573913      5.87
  hybridization  CHEMBL167232             0.468672    168.00
   substructure  CHEMBL313207             0.733333     16.00
          graph   CHEMBL77057             0.682759      5.87
        pubchem  CHEMBL430838             0.890756     91.20        *
      cdk_maccs   CHEMBL77754             0.810345     25.30
   klekota_roth   CHEMBL16520             0.613445    310.00
      *** ki ACTIVITY *** value prediction
```

based on 4 locally validated fgps ---> 72.839
["FeatMFP1", "Pattern", "pubchem", "AP_bits"]

4 rank
Matrix metalloproteinase-1 : Homo sapiens
score: 3.119 on CHEMBL332 based on 4 fingerprints

Fingerprint type	Ligand	Tanimoto Similarity	activity	Valid fg
FeatMFP1	CHEMBL415074	0.761905	5120.0	*
MFP1	CHEMBL430838	0.583333	9600.0	
RDKit7	CHEMBL112864	0.482465	1090.0	
Pattern	CHEMBL167232	0.915332	282.0	*
AP_bits	CHEMBL112864	0.550544	1090.0	*
TT_bits	CHEMBL430838	0.439394	9600.0	
FP2	CHEMBL77057	0.573913	313.0	
hybridization	CHEMBL167232	0.468672	282.0	
substructure	CHEMBL313207	0.733333	350.0	
graph	CHEMBL77057	0.682759	313.0	
pubchem	CHEMBL430838	0.890756	9600.0	*
cdk_maccs	CHEMBL77754	0.810345	7.8	
klekota_roth	CHEMBL16520	0.613445	2.0	

*** ki ACTIVITY *** value prediction
based on 4 locally validated fgps ---> 3223.905
["FeatMFP1", "Pattern", "pubchem", "AP_bits"]

5 rank
Matrix metalloproteinase 9 : Homo sapiens
score: 3.009 on CHEMBL321 based on 4 fingerprints

Fingerprint type	Ligand	Tanimoto Similarity	activity	Valid fg
FeatMFP1	CHEMBL71800	0.727273	3.0	
MFP1	CHEMBL71800	0.647059	3.0	*
RDKit7	CHEMBL367959	0.523873	5500.0	
Pattern	CHEMBL70406	0.941176	6.3	*
AP_bits	CHEMBL70406	0.548061	6.3	*
TT_bits	CHEMBL71800	0.477612	3.0	
FP2	CHEMBL367959	0.609524	5500.0	
hybridization	CHEMBL69024	0.514905	1.5	
substructure	CHEMBL171466	0.785714	150.0	
graph	CHEMBL381189	0.666667	6600.0	
pubchem	CHEMBL369184	0.872881	900.0	*
cdk_maccs	CHEMBL71800	0.824561	3.0	
klekota_roth	CHEMBL16520	0.613445	2.0	

*** ki ACTIVITY *** value prediction
based on 4 locally validated fgps ---> 135.367
["Pattern", "MFP1", "pubchem", "AP_bits"]

6 rank
Cathepsin K : Homo sapiens
score: 3.001 on CHEMBL268 based on 4 fingerprints

Fingerprint type	Ligand	Tanimoto Similarity	activity	Valid fg
FeatMFP1	CHEMBL99288	0.583333	110.0	
MFP1	CHEMBL371466	0.529412	1500.0	
RDKit7	CHEMBL96987	0.606258	20.0	
Pattern	CHEMBL2316590	0.874172	11.5	*
AP_bits	CHEMBL97402	0.538188	111.0	*
TT_bits	CHEMBL200235	0.491525	300.0	
FP2	CHEMBL607169	0.730769	10.1	*
hybridization	CHEMBL96987	0.486957	20.0	
substructure	CHEMBL198612	0.812500	246.0	
graph	CHEMBL3605412	0.608333	114.0	
pubchem	CHEMBL371466	0.858333	1500.0	*

```
    cdk_maccs   CHEMBL100482             0.693548      30.0
 klekota_roth  CHEMBL1651359             0.530973       2.9
    *** ki ACTIVITY *** value prediction
    based on 4 locally validated fgps ---> 1844.626
["Pattern", "FP2", "pubchem", "AP_bits"]
```

SwissTargetPrediction report:

Reference:
Gfeller D., Michielin O. & Zoete V.
Shaping the interaction landscape of bioactive molecules, *Bioinformatics* (2013) 29:3073-3079.

Query Molecule

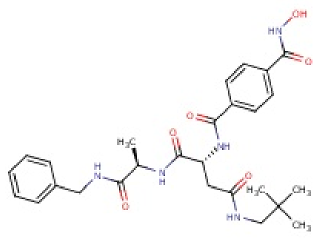

Frequency of Target Class

Target	Uniprot ID	Gene code	ChEMBL ID	Probability	# sim. cmpds (3D / 2D)	Target Class
Cathepsin L2 *(by homology)*	O60911	CTSL2	CHEMBL3272		77 / 150	Cysteine Protease
Cathepsin L1 light chain	P07711	CTSL1	CHEMBL3837		77 / 150	Cysteine Protease
Cathepsin S	P25774	CTSS	CHEMBL2954		77 / 150	Cysteine Protease
Cathepsin K	P43235	CTSK	CHEMBL268		77 / 150	Cysteine Protease
Microtubule-associated protein tau	P10636	MAPT	CHEMBL1293224		200 / 16	Unclassified
Disintegrin and metalloproteinase domain-containing protein 17	P78536	ADAM17	CHEMBL3706		236 / 39	Metallo Protease
22 kDa interstitial collagenase	P03956	MMP1	CHEMBL332		752 / 277	Metallo Protease
Stromelysin-1	P08254	MMP3	CHEMBL283		752 / 277	Metallo Protease
Stromelysin-2 *(by homology)*	P09238	MMP10	CHEMBL4270		752 / 277	Metallo Protease
Neutrophil collagenase *(by homology)*	P22894	MMP8	CHEMBL4588		684 / 273	Metallo Protease
Macrophage metalloelastase *(by homology)*	P39900	MMP12	CHEMBL4393		696 / 241	Metallo Protease
Collagenase 3 *(by homology)*	P45452	MMP13	CHEMBL280		697 / 241	Metallo Protease
Matrix metalloproteinase-27 *(by homology)*	Q9H306	MMP27			752 / 277	Metallo Protease
Histone deacetylase 1 *(by homology)*	Q13547	HDAC1	CHEMBL325		174 / 42	Enzyme
Histone deacetylase 2	Q92769	HDAC2	CHEMBL1937		174 / 42	Enzyme

Polypharmacology Browser 2 Prediction:

Targets predicted using NN(ECfp4) + NB(ECfp4). Save Table

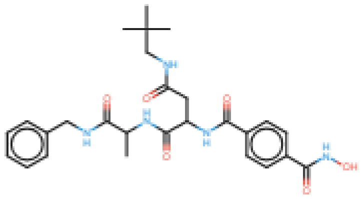

Query molecule

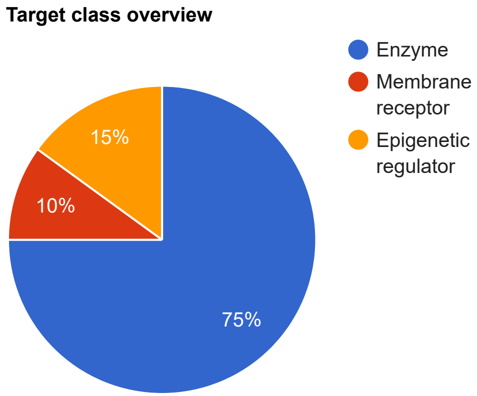

Rank	ChEMBL ID	Common name	Nearest neighbours
1	CHEMBL4662 (https://www.ebi.ac.uk/chembl/target/inspect/CHEMBL4662)	Proteasome Macropain subunit MB1	Show NN
2	CHEMBL325 (https://www.ebi.ac.uk/chembl/target/inspect/CHEMBL325)	Histone deacetylase 1	Show NN
3	CHEMBL4822 (https://www.ebi.ac.uk/chembl/target/inspect/CHEMBL4822)	Beta-secretase 1	Show NN
4	CHEMBL321 (https://www.ebi.ac.uk/chembl/target/inspect/CHEMBL321)	Matrix metalloproteinase 9	Show NN
5	CHEMBL333 (https://www.ebi.ac.uk/chembl/target/inspect/CHEMBL333)	Matrix metalloproteinase-2	Show NN
6	CHEMBL4072 (https://www.ebi.ac.uk/chembl/target/inspect/CHEMBL4072)	Cathepsin B	Show NN
7	CHEMBL332 (https://www.ebi.ac.uk/chembl/target/inspect/CHEMBL332)	Matrix metalloproteinase-1	Show NN
8	CHEMBL2954 (https://www.ebi.ac.uk/chembl/target/inspect/CHEMBL2954)	Cathepsin S	Show NN
9	CHEMBL1865 (https://www.ebi.ac.uk/chembl/target/inspect/CHEMBL1865)	Histone deacetylase 6	Show NN
10	CHEMBL205 (https://www.ebi.ac.uk/chembl/target/inspect/CHEMBL205)	Carbonic anhydrase II	Show NN
11	CHEMBL3192 (https://www.ebi.ac.uk/chembl/target/inspect/CHEMBL3192)	Histone deacetylase 8	Show NN
12	CHEMBL4588 (https://www.ebi.ac.uk/chembl/target/inspect/CHEMBL4588)	Matrix metalloproteinase 8	Show NN
13	CHEMBL261 (https://www.ebi.ac.uk/chembl/target/inspect/CHEMBL261)	Carbonic anhydrase I	Show NN
14	CHEMBL233 (https://www.ebi.ac.uk/chembl/target/inspect/CHEMBL233)	Mu opioid receptor	Show NN
15	CHEMBL3837 (https://www.ebi.ac.uk/chembl/target/inspect/CHEMBL3837)	Cathepsin L	Show NN
16	CHEMBL268 (https://www.ebi.ac.uk/chembl/target/inspect/CHEMBL268)	Cathepsin K	Show NN
17	CHEMBL3286 (https://www.ebi.ac.uk/chembl/target/inspect/CHEMBL3286)	Urokinase-type plasminogen activator	Show NN
18	CHEMBL204 (https://www.ebi.ac.uk/chembl/target/inspect/CHEMBL204)	Thrombin	Show NN

19	CHEMBL236 (https://www.ebi.ac.uk/chembl/target/inspect/CHEMBL236)	Delta opioid receptor	Show NN
20	CHEMBL4801 (https://www.ebi.ac.uk/chembl/target/inspect/CHEMBL4801)	Caspase-1	Show NN
