# Supplementary material for: Accelerating Drug Discovery by Early Protein Drug Target Prediction Based on a Multi-Fingerprint Similarity Search
Source: Molecules. 2019 Jun 14;24(12):2233. doi: 10.3390/molecules24122233 (PMC6631269; doi:10.3390/molecules24122233)
Supplement: Supplementary file 1 [file molecules-24-02233-s001.zip › File_S2.zip!/File_S2/Target_Prediction_compound#33.pdf]

MuSSel Prediction IC$_{50}$:

```
1 rank
Acetylcholinesterase : Electrophorus electricus
      score: 6.335 on CHEMBL4078 based on 9 fingerprints
Fingerprint type         Ligand  Tanimoto Similarity  activity Valid fg
       FeatMFP1   CHEMBL470867            0.727273    330.00        *
           MFP1   CHEMBL470867            0.575758    330.00        *
         RDKit7   CHEMBL258893            0.596059   3780.00
        Pattern   CHEMBL258893            0.893987   3780.00        *
        AP_bits  CHEMBL1270850            0.610592   1800.00        *
        TT_bits   CHEMBL258893            0.590909   3780.00        *
            FP2   CHEMBL258893            0.640449   3780.00        *
  hybridization   CHEMBL258893            0.661157   3780.00        *
   substructure  CHEMBL3793762            0.842105    465.96
          graph  CHEMBL3819248            0.814159    131.00        *
        pubchem   CHEMBL374981            0.819512      2.73
      cdk_maccs  CHEMBL1271342            0.820896   1740.00        *
   klekota_roth  CHEMBL1271342            0.563380   1740.00
      *** ic50 ACTIVITY *** value prediction
      based on 9 locally validated fgps ---> 2034.977
["TT_bits", "Pattern", "AP_bits", "MFP1", "graph", "FP2", "FeatMFP1",
"cdk_maccs", "hybridization"]

2 rank
Acetylcholinesterase : Homo sapiens
      score: 5.542 on CHEMBL220 based on 8 fingerprints
Fingerprint type         Ligand  Tanimoto Similarity  activity Valid fg
       FeatMFP1   CHEMBL470867            0.727273    540.00        *
           MFP1   CHEMBL507903            0.588235      7.70        *
         RDKit7   CHEMBL258893            0.596059   1540.00
        Pattern   CHEMBL258893            0.893987   1540.00        *
        AP_bits    CHEMBL12089            0.603226     40.00        *
        TT_bits   CHEMBL258893            0.590909   1540.00        *
            FP2   CHEMBL258893            0.640449   1540.00        *
  hybridization   CHEMBL258893            0.661157   1540.00        *
   substructure  CHEMBL3335056            0.842105   6700.00
          graph  CHEMBL3233992            0.836364  61400.00        *
        pubchem   CHEMBL374981            0.819512      5.08
      cdk_maccs  CHEMBL3407582            0.780822   8400.00
   klekota_roth   CHEMBL491526            0.560000   2370.00
      *** ic50 ACTIVITY *** value prediction
      based on 8 locally validated fgps ---> 8912.199
["TT_bits", "Pattern", "AP_bits", "MFP1", "graph", "FP2", "FeatMFP1",
"hybridization"]

3 rank
Reverse transcriptase : Human immunodeficiency virus 1
      score: 4.855 on CHEMBL2366516 based on 7 fingerprints
Fingerprint type         Ligand  Tanimoto Similarity  activity Valid fg
       FeatMFP1   CHEMBL485509            0.482759   4677.35
           MFP1   CHEMBL292540            0.529412   5500.00        *
         RDKit7     CHEMBL8443            0.650138     49.00
        Pattern     CHEMBL8443            0.887122     49.00        *
        AP_bits     CHEMBL8443            0.596215     49.00        *
        TT_bits     CHEMBL8443            0.645161     49.00        *
            FP2     CHEMBL8443            0.678571     49.00        *
  hybridization     CHEMBL8443            0.709821     49.00        *
   substructure   CHEMBL292540            0.684211   5500.00
          graph     CHEMBL8443            0.809091     49.00        *
        pubchem  CHEMBL2407273            0.737374   3850.00
      cdk_maccs  CHEMBL3338519            0.698630  25770.00
```

```
   klekota_roth  CHEMBL3338519             0.412500  25770.00
      *** ic50 ACTIVITY *** value prediction
      based on 7 locally validated fgps ---> 1454.398
["TT_bits", "Pattern", "AP_bits", "MFP1", "graph", "FP2",
"hybridization"]

4 rank
Cholinesterase : Equus caballus
      score: 4.739 on CHEMBL5763 based on 7 fingerprints
Fingerprint type         Ligand  Tanimoto Similarity  activity Valid fg
       FeatMFP1  CHEMBL3323054             0.666667    5220.0
           MFP1  CHEMBL1186488             0.531250   19340.0        *
         RDKit7   CHEMBL258893             0.596059    6330.0
        Pattern   CHEMBL258893             0.893987    6330.0        *
        AP_bits    CHEMBL12089             0.603226   18200.0        *
        TT_bits   CHEMBL258893             0.590909    6330.0        *
            FP2   CHEMBL258893             0.640449    6330.0        *
  hybridization   CHEMBL258893             0.661157    6330.0        *
   substructure  CHEMBL2323351             0.833333   71700.0
          graph  CHEMBL3819247             0.818182    9820.0        *
        pubchem  CHEMBL3819248             0.819095    4249.0
      cdk_maccs  CHEMBL1164080             0.782609    1310.0
   klekota_roth  CHEMBL1186488             0.565217   19340.0
      *** ic50 ACTIVITY *** value prediction
      based on 7 locally validated fgps ---> 10339.888
["TT_bits", "Pattern", "AP_bits", "MFP1", "graph", "FP2",
"hybridization"]

5 rank
Butyrylcholinesterase : Homo sapiens
      score: 4.674 on CHEMBL1914 based on 7 fingerprints
Fingerprint type         Ligand  Tanimoto Similarity  activity Valid fg
       FeatMFP1  CHEMBL3335057             0.695652  33200.00        *
           MFP1   CHEMBL507903             0.588235   3000.00        *
         RDKit7   CHEMBL258893             0.596059  10340.00
        Pattern   CHEMBL258893             0.893987  10340.00        *
        AP_bits    CHEMBL12089             0.603226  18970.00        *
        TT_bits   CHEMBL258893             0.590909  10340.00        *
            FP2   CHEMBL258893             0.640449  10340.00        *
  hybridization   CHEMBL258893             0.661157  10340.00        *
   substructure  CHEMBL3335056             0.842105  14100.00
          graph    CHEMBL12089             0.747899  18970.00
        pubchem   CHEMBL374981             0.819512      1.38
      cdk_maccs  CHEMBL3407582             0.780822   5900.00
   klekota_roth   CHEMBL491526             0.560000  88800.00
      *** ic50 ACTIVITY *** value prediction
      based on 7 locally validated fgps ---> 10781.231
["TT_bits", "Pattern", "AP_bits", "MFP1", "FP2", "FeatMFP1",
"hybridization"]
```

SwissTargetPrediction report:

Reference:
Gfeller D., Michielin O. & Zoete V.
Shaping the interaction landscape of bioactive molecules, *Bioinformatics* (2013) 29:3073-3079.

Query Molecule

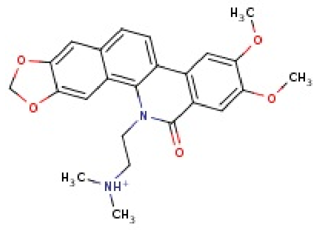

Frequency of Target Class

Target	Uniprot ID	Gene code	ChEMBL ID	Probability	# sim. cmpds (3D / 2D)	Target Class
DNA topoisomerase 1	P11387	TOP1	CHEMBL1781		21 / 15	Enzyme
DNA topoisomerase I, mitochondrial *(by homology)*	Q969P6	TOP1MT			21 / 15	Enzyme
Cyclin-dependent kinase 1	P06493	CDK1	CHEMBL308		226 / 5	Ser_Thr Kinase
Cyclin-dependent kinase 4	P11802	CDK4	CHEMBL331		221 / 5	Ser_Thr Kinase
Cyclin-dependent kinase 2	P24941	CDK2	CHEMBL301		226 / 5	Ser_Thr Kinase
Cyclin-dependent kinase 3 *(by homology)*	Q00526	CDK3	CHEMBL4442		226 / 5	Ser_Thr Kinase
Cyclin-dependent kinase 6 *(by homology)*	Q00534	CDK6	CHEMBL2508		221 / 5	Ser_Thr Kinase
Aurora kinase A	O14965	AURKA	CHEMBL4722		201 / 2	Ser_Thr Kinase
Aurora kinase B *(by homology)*	Q96GD4	AURKB	CHEMBL2185		201 / 2	Ser_Thr Kinase
Aurora kinase C *(by homology)*	Q9UQB9	AURKC	CHEMBL3935		201 / 2	Ser_Thr Kinase
Cholinesterase	P06276	BCHE	CHEMBL1914		124 / 7	Enzyme
Acetylcholinesterase	P22303	ACHE	CHEMBL220		124 / 7	Enzyme
Cytochrome P450 19A1	P11511	CYP19A1	CHEMBL1978		31 / 10	Enzyme
Adenosine receptor A1 *(by homology)*	P30542	ADORA1	CHEMBL226		170 / 1	Membrane receptor
Protein kinase C eta type	P24723	PRKCH	CHEMBL3616		60 / 2	Ser_Thr Kinase

Polypharmacology Browser 2 Prediction:

Targets predicted using NN(ECfp4) + NB(ECfp4). Save Table

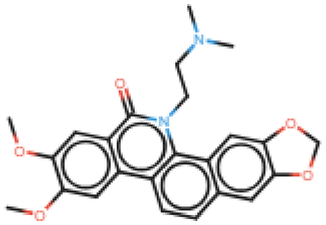

Query molecule

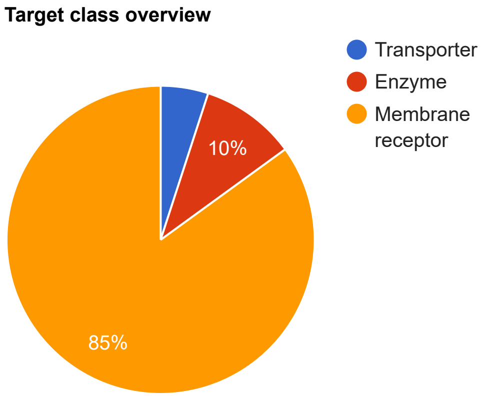

Rank	ChEMBL ID	Common name	Nearest neighbours
1	CHEMBL214 (https://www.ebi.ac.uk/chembl/target/inspect/CHEMBL214)	Serotonin 1a (5-HT1a) receptor	Show NN
2	CHEMBL273 (https://www.ebi.ac.uk/chembl/target/inspect/CHEMBL273)	Serotonin 1a (5-HT1a) receptor	Show NN
3	CHEMBL339 (https://www.ebi.ac.uk/chembl/target/inspect/CHEMBL339)	Dopamine D2 receptor	Show NN
4	CHEMBL217 (https://www.ebi.ac.uk/chembl/target/inspect/CHEMBL217)	Dopamine D2 receptor	Show NN
5	CHEMBL1827 (https://www.ebi.ac.uk/chembl/target/inspect/CHEMBL1827)	Phosphodiesterase 5A	Show NN
6	CHEMBL253 (https://www.ebi.ac.uk/chembl/target/inspect/CHEMBL253)	Cannabinoid CB2 receptor	Show NN
7	CHEMBL319 (https://www.ebi.ac.uk/chembl/target/inspect/CHEMBL319)	Alpha-1a adrenergic receptor	Show NN
8	CHEMBL326 (https://www.ebi.ac.uk/chembl/target/inspect/CHEMBL326)	Alpha-1d adrenergic receptor	Show NN
9	CHEMBL225 (https://www.ebi.ac.uk/chembl/target/inspect/CHEMBL225)	Serotonin 2c (5-HT2c) receptor	Show NN
10	CHEMBL224 (https://www.ebi.ac.uk/chembl/target/inspect/CHEMBL224)	Serotonin 2a (5-HT2a) receptor	Show NN
11	CHEMBL220 (https://www.ebi.ac.uk/chembl/target/inspect/CHEMBL220)	Acetylcholinesterase	Show NN
12	CHEMBL2056 (https://www.ebi.ac.uk/chembl/target/inspect/CHEMBL2056)	Dopamine D1 receptor	Show NN
13	CHEMBL4302 (https://www.ebi.ac.uk/chembl/target/inspect/CHEMBL4302)	P-glycoprotein 1	Show NN
14	CHEMBL234 (https://www.ebi.ac.uk/chembl/target/inspect/CHEMBL234)	Dopamine D3 receptor	Show NN
15	CHEMBL3155 (https://www.ebi.ac.uk/chembl/target/inspect/CHEMBL3155)	Serotonin 7 (5-HT7) receptor	Show NN
16	CHEMBL232 (https://www.ebi.ac.uk/chembl/target/inspect/CHEMBL232)	Alpha-1b adrenergic receptor	Show NN
17	CHEMBL229 (https://www.ebi.ac.uk/chembl/target/inspect/CHEMBL229)	Alpha-1a adrenergic receptor	Show NN
18	CHEMBL3371 (https://www.ebi.ac.uk/chembl/target/inspect/CHEMBL3371)	Serotonin 6 (5-HT6) receptor	Show NN

19	CHEMBL287 (https://www.ebi.ac.uk/chembl/target/inspect/CHEMBL287)	Sigma opioid receptor	Show NN
20	CHEMBL223 (https://www.ebi.ac.uk/chembl/target/inspect/CHEMBL223)	Alpha-1d adrenergic receptor	Show NN
