# Supplementary material for: Accelerating Drug Discovery by Early Protein Drug Target Prediction Based on a Multi-Fingerprint Similarity Search
Source: Molecules. 2019 Jun 14;24(12):2233. doi: 10.3390/molecules24122233 (PMC6631269; doi:10.3390/molecules24122233)
Supplement: Supplementary file 1 [file molecules-24-02233-s001.zip › File_S2.zip!/File_S2/Target_Prediction_compound#34.pdf]

MuSSel not predicted

SwissTargetPrediction report:

Reference:
Gfeller D., Michielin O. & Zoete V.
Shaping the interaction landscape of bioactive molecules, *Bioinformatics* (2013) 29:3073-3079.

Query Molecule

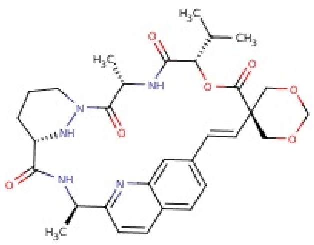

Frequency of Target Class

Target	Uniprot ID	Gene code	ChEMBL ID	Probability	# sim. cmpds (3D / 2D)	Target Class
Caspase-3 subunit p12	P42574	CASP3	CHEMBL2334		70 / 1	Cysteine Protease
Caspase-7 subunit p20 *(by homology)*	P55210	CASP7	CHEMBL3468		70 / 1	Cysteine Protease
Caspase-6 subunit p18 *(by homology)*	P55212	CASP6	CHEMBL3308		70 / 1	Cysteine Protease
Caspase-1 subunit p20	P29466	CASP1	CHEMBL4801		95 / 12	Cysteine Protease
Caspase-8 subunit p18	Q14790	CASP8	CHEMBL3776		64 / 6	Cysteine Protease
Caspase-4 *(by homology)*	P49662	CASP4	CHEMBL2226		95 / 12	Cysteine Protease
Caspase-5 subunit p10 *(by homology)*	P51878	CASP5	CHEMBL3131		95 / 12	Cysteine Protease
Inactive caspase-12 *(by homology)*	Q6UXS9	CASP12			95 / 12	Cysteine Protease
Cathepsin L2 *(by homology)*	O60911	CTSL2	CHEMBL3272		222 / 8	Cysteine Protease
Cathepsin L1 light chain	P07711	CTSL1	CHEMBL3837		222 / 8	Cysteine Protease
Cathepsin S	P25774	CTSS	CHEMBL2954		221 / 8	Cysteine Protease
Cathepsin K	P43235	CTSK	CHEMBL268		221 / 8	Cysteine Protease
Cathepsin B	P07858	CTSB	CHEMBL4072		53 / 1	Cysteine Protease
Microtubule-associated protein tau	P10636	MAPT	CHEMBL1293224		665 / 1	Unclassified
Renin	P00797	REN	CHEMBL286		360 / 57	Aspartic Protease

Polypharmacology Browser 2 Prediction:

Targets predicted using NN(ECfp4) + NB(ECfp4). Save Table

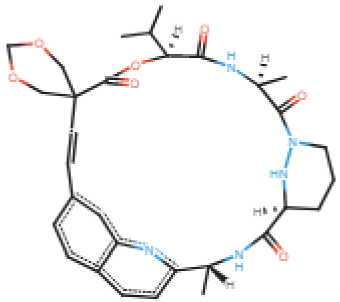

Query molecule

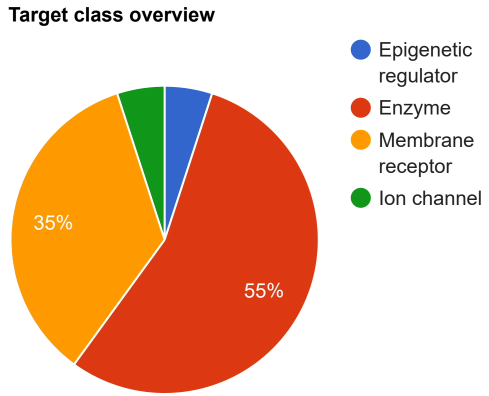

Rank	ChEMBL ID	Common name	Nearest neighbours
1	CHEMBL240 (https://www.ebi.ac.uk/chembl/target/inspect/CHEMBL240)	HERG	Show NN
2	CHEMBL214 (https://www.ebi.ac.uk/chembl/target/inspect/CHEMBL214)	Serotonin 1a (5-HT1a) receptor	Show NN
3	CHEMBL4822 (https://www.ebi.ac.uk/chembl/target/inspect/CHEMBL4822)	Beta-secretase 1	Show NN
4	CHEMBL268 (https://www.ebi.ac.uk/chembl/target/inspect/CHEMBL268)	Cathepsin K	Show NN
5	CHEMBL274 (https://www.ebi.ac.uk/chembl/target/inspect/CHEMBL274)	C-C chemokine receptor type 5	Show NN
6	CHEMBL340 (https://www.ebi.ac.uk/chembl/target/inspect/CHEMBL340)	Cytochrome P450 3A4	Show NN
7	CHEMBL253 (https://www.ebi.ac.uk/chembl/target/inspect/CHEMBL253)	Cannabinoid CB2 receptor	Show NN
8	CHEMBL325 (https://www.ebi.ac.uk/chembl/target/inspect/CHEMBL325)	Histone deacetylase 1	Show NN
9	CHEMBL4072 (https://www.ebi.ac.uk/chembl/target/inspect/CHEMBL4072)	Cathepsin B	Show NN
10	CHEMBL2954 (https://www.ebi.ac.uk/chembl/target/inspect/CHEMBL2954)	Cathepsin S	Show NN
11	CHEMBL3105 (https://www.ebi.ac.uk/chembl/target/inspect/CHEMBL3105)	Poly [ADP-ribose] polymerase-1	Show NN
12	CHEMBL4409 (https://www.ebi.ac.uk/chembl/target/inspect/CHEMBL4409)	Phosphodiesterase 10A	Show NN
13	CHEMBL260 (https://www.ebi.ac.uk/chembl/target/inspect/CHEMBL260)	MAP kinase p38 alpha	Show NN
14	CHEMBL262 (https://www.ebi.ac.uk/chembl/target/inspect/CHEMBL262)	Glycogen synthase kinase-3 beta	Show NN
15	CHEMBL4005 (https://www.ebi.ac.uk/chembl/target/inspect/CHEMBL4005)	PI3-kinase p110-alpha subunit	Show NN
16	CHEMBL3837 (https://www.ebi.ac.uk/chembl/target/inspect/CHEMBL3837)	Cathepsin L	Show NN
17	CHEMBL344 (https://www.ebi.ac.uk/chembl/target/inspect/CHEMBL344)	Melanin-concentrating hormone receptor 1	Show NN
18	CHEMBL273 (https://www.ebi.ac.uk/chembl/target/inspect/CHEMBL273)	Serotonin 1a (5-HT1a) receptor	Show NN

19	CHEMBL3996 (https://www.ebi.ac.uk/chembl/target/inspect/CHEMBL3996)	Oxytocin receptor	Show NN
20	CHEMBL233 (https://www.ebi.ac.uk/chembl/target/inspect/CHEMBL233)	Mu opioid receptor	Show NN
