# Supplementary material for: Accelerating Drug Discovery by Early Protein Drug Target Prediction Based on a Multi-Fingerprint Similarity Search
Source: Molecules. 2019 Jun 14;24(12):2233. doi: 10.3390/molecules24122233 (PMC6631269; doi:10.3390/molecules24122233)
Supplement: Supplementary file 1 [file molecules-24-02233-s001.zip › File_S2.zip!/File_S2/Target_Prediction_compound#35.pdf]

MuSSel Prediction IC_{50}:

1 rank
Vascular endothelial growth factor receptor 2 : Homo sapiens
score: 6.544 on CHEMBL279 based on 9 fingerprints

Fingerprint type	Ligand	Tanimoto Similarity	activity	Valid fg
FeatMFP1	CHEMBL3754556	0.680000	86.67	*
MFP1	CHEMBL3746595	0.560976	4.00	*
RDKit7	CHEMBL612137	0.516364	40.00	
Pattern	CHEMBL470483	0.881696	470.00	*
AP_bits	CHEMBL1242977	0.576497	3270.00	*
TT_bits	CHEMBL272670	0.454545	19.95	
FP2	CHEMBL272670	0.633987	19.95	*
hybridization	CHEMBL182283	0.658046	630.00	*
substructure	CHEMBL577742	0.842105	13.00	
graph	CHEMBL183504	0.841808	2040.00	*
pubchem	CHEMBL3814321	0.871287	120.00	*
cdk_maccs	CHEMBL600398	0.839286	860.00	*
klekota_roth	CHEMBL243916	0.555556	2.60	

*** ic50 ACTIVITY *** value prediction
based on 8 locally validated fgps ---> 645.383
["Pattern", "AP_bits", "MFP1", "pubchem", "graph", "FP2", "cdk_maccs", "hybridization"]

2 rank
MAP kinase signal-integrating kinase 2 : Homo sapiens
score: 5.958 on CHEMBL4204 based on 8 fingerprints

Fingerprint type	Ligand	Tanimoto Similarity	activity	Valid fg
FeatMFP1	CHEMBL3805074	0.636364	22.0	
MFP1	CHEMBL3805074	0.636364	22.0	*
RDKit7	CHEMBL3805094	0.706952	760.0	*
Pattern	CHEMBL3805094	0.855422	760.0	*
AP_bits	CHEMBL3805094	0.444444	760.0	
TT_bits	CHEMBL3805094	0.597015	760.0	*
FP2	CHEMBL3805094	0.774194	760.0	*
hybridization	CHEMBL3805094	0.630653	760.0	*
substructure	CHEMBL3804952	0.761905	8.0	
graph	CHEMBL3805094	0.826347	760.0	*
pubchem	CHEMBL3805094	0.931034	760.0	*
cdk_maccs	CHEMBL3698947	0.700000	11.0	
klekota_roth	CHEMBL3805094	0.493151	760.0	

*** ic50 ACTIVITY *** value prediction
based on 8 locally validated fgps ---> 531.260
["TT_bits", "Pattern", "MFP1", "pubchem", "graph", "FP2", "RDKit7", "hybridization"]

3 rank
Epidermal growth factor receptor erbB1 : Homo sapiens
score: 5.022 on CHEMBL203 based on 7 fingerprints

Fingerprint type	Ligand	Tanimoto Similarity	activity	Valid fg
FeatMFP1	CHEMBL3754556	0.680000	71.82	*
MFP1	CHEMBL1928946	0.600000	108.00	*
RDKit7	CHEMBL1928956	0.506826	627.00	
Pattern	CHEMBL387187	0.905192	21.00	*
AP_bits	CHEMBL3814846	0.514943	3.96	*
TT_bits	CHEMBL1928946	0.477612	108.00	
FP2	CHEMBL1928956	0.639785	627.00	*
hybridization	CHEMBL247228	0.476804	10000.00	
substructure	CHEMBL2335018	0.833333	12800.00	
graph	CHEMBL1928954	0.692308	1671.00	
pubchem	CHEMBL14627	0.833333	74.00	*
cdk_maccs	CHEMBL1928956	0.849057	627.00	*

```
   klekota_roth  CHEMBL3774451             0.404255    582.17
      *** ic50 ACTIVITY *** value prediction
      based on 6 locally validated fgps ---> 576.366
["Pattern", "AP_bits", "MFP1", "pubchem", "FP2", "cdk_maccs"]
```

MuSSel Prediction k_i:

```
1 rank
Leucine-rich repeat serine/threonine-protein kinase 2 : Homo sapiens
      score: 3.810 on CHEMBL1075104 based on 5 fingerprints
Fingerprint type         Ligand  Tanimoto Similarity  activity Valid fg
       FeatMFP1  CHEMBL1992581             0.615385  1584.890
           MFP1   CHEMBL574738             0.590909  1000.000        *
         RDKit7   CHEMBL574738             0.602683  1000.000
        Pattern  CHEMBL3641887             0.873391     5.200        *
        AP_bits  CHEMBL1988076             0.441667  2511.890
        TT_bits   CHEMBL574738             0.567164  1000.000        *
            FP2   CHEMBL574738             0.614458  1000.000
  hybridization   CHEMBL574738             0.504902  1000.000
   substructure  CHEMBL1983111             0.882353     0.631        *
          graph   CHEMBL574738             0.733333  1000.000
        pubchem   CHEMBL574738             0.896040  1000.000        *
      cdk_maccs  CHEMBL3641833             0.762712     5.200
   klekota_roth  CHEMBL1992581             0.450980  1584.890
      *** ki ACTIVITY *** value prediction
      based on 5 locally validated fgps ---> 600.887
["Pattern", "TT_bits", "MFP1", "substructure", "pubchem"]

2 rank
Tyrosine-protein kinase ABL : Homo sapiens
      score: 3.483 on CHEMBL1862 based on 5 fingerprints
Fingerprint type         Ligand  Tanimoto Similarity  activity Valid fg
       FeatMFP1  CHEMBL1992581             0.615385    199.53
           MFP1   CHEMBL574738             0.590909     25.12        *
         RDKit7   CHEMBL574738             0.602683     25.12
        Pattern  CHEMBL1992306             0.855580    630.96
        AP_bits  CHEMBL1992306             0.546539    630.96        *
        TT_bits   CHEMBL574738             0.567164     25.12        *
            FP2   CHEMBL574738             0.614458     25.12
  hybridization   CHEMBL574738             0.504902     25.12
   substructure  CHEMBL1983111             0.882353    251.19        *
          graph   CHEMBL574738             0.733333     25.12
        pubchem   CHEMBL574738             0.896040     25.12        *
      cdk_maccs     CHEMBL1336             0.750000    158.49
   klekota_roth  CHEMBL1973178             0.460000    199.53
      *** ki ACTIVITY *** value prediction
      based on 5 locally validated fgps ---> 166.683
["AP_bits", "TT_bits", "MFP1", "substructure", "pubchem"]

3 rank
Tyrosine-protein kinase receptor RET : Homo sapiens
      score: 3.483 on CHEMBL2041 based on 5 fingerprints
Fingerprint type         Ligand  Tanimoto Similarity  activity Valid fg
       FeatMFP1  CHEMBL1992581             0.615385   199.530
           MFP1   CHEMBL574738             0.590909     6.310        *
         RDKit7   CHEMBL574738             0.602683     6.310
        Pattern  CHEMBL1992306             0.855580     7.943
        AP_bits  CHEMBL1992306             0.546539     7.943        *
        TT_bits   CHEMBL574738             0.567164     6.310        *
            FP2   CHEMBL574738             0.614458     6.310
  hybridization   CHEMBL574738             0.504902     6.310
   substructure  CHEMBL1983111             0.882353     3.162        *
          graph   CHEMBL574738             0.733333     6.310
        pubchem   CHEMBL574738             0.896040     6.310        *
      cdk_maccs  CHEMBL1982660             0.754717   100.000
   klekota_roth  CHEMBL1992581             0.450980   199.530
      *** ki ACTIVITY *** value prediction
```

```
      based on 5 locally validated fgps ---> 6.007
["AP_bits", "TT_bits", "MFP1", "substructure", "pubchem"]

4 rank
Tyrosine-protein kinase LCK : Homo sapiens
      score: 3.483 on CHEMBL258 based on 5 fingerprints
Fingerprint type         Ligand  Tanimoto Similarity  activity Valid fg
       FeatMFP1  CHEMBL1992581             0.615385   3162.28
           MFP1   CHEMBL574738             0.590909     15.85        *
         RDKit7   CHEMBL574738             0.602683     15.85
        Pattern  CHEMBL1992306             0.855580    251.19
        AP_bits  CHEMBL1992306             0.546539    251.19        *
        TT_bits   CHEMBL574738             0.567164     15.85        *
            FP2   CHEMBL574738             0.614458     15.85
  hybridization   CHEMBL574738             0.504902     15.85
   substructure  CHEMBL1983111             0.882353    199.53        *
          graph   CHEMBL574738             0.733333     15.85
        pubchem   CHEMBL574738             0.896040     15.85        *
      cdk_maccs  CHEMBL2170582             0.803279    116.00
   klekota_roth  CHEMBL1973178             0.460000     31.62
      *** ki ACTIVITY *** value prediction
      based on 5 locally validated fgps ---> 99.654
["AP_bits", "TT_bits", "MFP1", "substructure", "pubchem"]

5 rank
Tyrosine-protein kinase JAK2 : Homo sapiens
      score: 3.483 on CHEMBL2971 based on 5 fingerprints
Fingerprint type         Ligand  Tanimoto Similarity  activity Valid fg
       FeatMFP1  CHEMBL2420821             0.576923   310.000
           MFP1   CHEMBL574738             0.590909  1000.000        *
         RDKit7   CHEMBL574738             0.602683  1000.000
        Pattern  CHEMBL1992306             0.855580   630.960
        AP_bits  CHEMBL1992306             0.546539   630.960        *
        TT_bits   CHEMBL574738             0.567164  1000.000        *
            FP2   CHEMBL574738             0.614458  1000.000
  hybridization   CHEMBL574738             0.504902  1000.000
   substructure  CHEMBL1983111             0.882353     3.981        *
          graph   CHEMBL574738             0.733333  1000.000
        pubchem   CHEMBL574738             0.896040  1000.000        *
      cdk_maccs  CHEMBL2170582             0.803279   130.000
   klekota_roth  CHEMBL2002723             0.431818  1995.260
      *** ki ACTIVITY *** value prediction
      based on 5 locally validated fgps ---> 727.890
["AP_bits", "TT_bits", "MFP1", "substructure", "pubchem"]

6 rank
Serine/threonine-protein kinase Aurora-A : Homo sapiens
      score: 3.483 on CHEMBL4722 based on 5 fingerprints
Fingerprint type         Ligand  Tanimoto Similarity  activity Valid fg
       FeatMFP1  CHEMBL1976455             0.576923  1995.260
           MFP1   CHEMBL574738             0.590909  1584.890        *
         RDKit7   CHEMBL574738             0.602683  1584.890
        Pattern  CHEMBL1992306             0.855580   501.190
        AP_bits  CHEMBL1992306             0.546539   501.190        *
        TT_bits   CHEMBL574738             0.567164  1584.890        *
            FP2   CHEMBL574738             0.614458  1584.890
  hybridization   CHEMBL574738             0.504902  1584.890
   substructure  CHEMBL1983111             0.882353     3.981        *
          graph   CHEMBL574738             0.733333  1584.890
        pubchem   CHEMBL574738             0.896040  1584.890        *
```

```
       cdk_maccs     CHEMBL1336             0.750000   794.330
   klekota_roth  CHEMBL2002723             0.431818  1584.890
      *** ki ACTIVITY *** value prediction
      based on 5 locally validated fgps ---> 904.998
["AP_bits", "TT_bits", "MFP1", "substructure", "pubchem"]

7 rank
Neurotrophic tyrosine kinase receptor type 2 : Homo sapiens
      score: 3.483 on CHEMBL4898 based on 5 fingerprints
Fingerprint type         Ligand  Tanimoto Similarity  activity Valid fg
       FeatMFP1  CHEMBL1992581             0.615385    251.19
           MFP1   CHEMBL574738             0.590909    251.19        *
         RDKit7   CHEMBL574738             0.602683    251.19
        Pattern  CHEMBL1992306             0.855580    125.89
        AP_bits  CHEMBL1992306             0.546539    125.89        *
        TT_bits   CHEMBL574738             0.567164    251.19        *
            FP2   CHEMBL574738             0.614458    251.19
  hybridization   CHEMBL574738             0.504902    251.19
   substructure  CHEMBL1983111             0.882353     31.62        *
          graph   CHEMBL574738             0.733333    251.19
        pubchem   CHEMBL574738             0.896040    251.19        *
      cdk_maccs     CHEMBL1336             0.750000    630.96
   klekota_roth  CHEMBL1992581             0.450980    251.19
      *** ki ACTIVITY *** value prediction
      based on 5 locally validated fgps ---> 182.216
["AP_bits", "TT_bits", "MFP1", "substructure", "pubchem"]

8 rank
Serine/threonine-protein kinase TAO1 : Homo sapiens
      score: 3.483 on CHEMBL5261 based on 5 fingerprints
Fingerprint type         Ligand  Tanimoto Similarity  activity Valid fg
       FeatMFP1  CHEMBL1992306             0.571429     79.43
           MFP1   CHEMBL574738             0.590909    501.19        *
         RDKit7   CHEMBL574738             0.602683    501.19
        Pattern  CHEMBL1992306             0.855580     79.43
        AP_bits  CHEMBL1992306             0.546539     79.43        *
        TT_bits   CHEMBL574738             0.567164    501.19        *
            FP2   CHEMBL574738             0.614458    501.19
  hybridization   CHEMBL574738             0.504902    501.19
   substructure  CHEMBL1983111             0.882353    100.00        *
          graph   CHEMBL574738             0.733333    501.19
        pubchem   CHEMBL574738             0.896040    501.19        *
      cdk_maccs     CHEMBL1336             0.750000    398.11
   klekota_roth  CHEMBL2002723             0.431818   5011.87
      *** ki ACTIVITY *** value prediction
      based on 5 locally validated fgps ---> 336.600
["AP_bits", "TT_bits", "MFP1", "substructure", "pubchem"]

9 rank
Tyrosine-protein kinase receptor TYRO3 : Homo sapiens
      score: 3.483 on CHEMBL5314 based on 5 fingerprints
Fingerprint type         Ligand  Tanimoto Similarity  activity Valid fg
       FeatMFP1  CHEMBL1986756             0.629630    251.19
           MFP1   CHEMBL574738             0.590909   1584.89        *
         RDKit7   CHEMBL574738             0.602683   1584.89
        Pattern  CHEMBL1992306             0.855580    316.23
        AP_bits  CHEMBL1992306             0.546539    316.23        *
        TT_bits   CHEMBL574738             0.567164   1584.89        *
            FP2   CHEMBL574738             0.614458   1584.89
  hybridization   CHEMBL574738             0.504902   1584.89
```

```
   substructure  CHEMBL1983111             0.882353    125.89        *
          graph   CHEMBL574738             0.733333   1584.89
        pubchem   CHEMBL574738             0.896040   1584.89        *
      cdk_maccs  CHEMBL1992306             0.740741    316.23
   klekota_roth  CHEMBL2002723             0.431818   1258.93
     *** ki ACTIVITY *** value prediction
     based on 5 locally validated fgps ---> 1039.358
["AP_bits", "TT_bits", "MFP1", "substructure", "pubchem"]

10 rank
Proto-oncogene tyrosine-protein kinase ROS : Homo sapiens
     score: 3.483 on CHEMBL5568 based on 5 fingerprints
Fingerprint type         Ligand  Tanimoto Similarity  activity Valid fg
       FeatMFP1  CHEMBL1992306             0.571429   1000.00
           MFP1   CHEMBL574738             0.590909   1995.26        *
         RDKit7   CHEMBL574738             0.602683   1995.26
        Pattern  CHEMBL1992306             0.855580   1000.00
        AP_bits  CHEMBL1992306             0.546539   1000.00        *
        TT_bits   CHEMBL574738             0.567164   1995.26        *
            FP2   CHEMBL574738             0.614458   1995.26
  hybridization   CHEMBL574738             0.504902   1995.26
   substructure  CHEMBL1983111             0.882353     31.62        *
          graph   CHEMBL574738             0.733333   1995.26
        pubchem   CHEMBL574738             0.896040   1995.26        *
      cdk_maccs  CHEMBL1982660             0.754717     63.10
   klekota_roth  CHEMBL1992306             0.413793   1000.00
     *** ki ACTIVITY *** value prediction
     based on 5 locally validated fgps ---> 1403.480
["AP_bits", "TT_bits", "MFP1", "substructure", "pubchem"]

11 rank
NT-3 growth factor receptor : Homo sapiens
     score: 3.483 on CHEMBL5608 based on 5 fingerprints
Fingerprint type         Ligand  Tanimoto Similarity  activity Valid fg
       FeatMFP1  CHEMBL1992581             0.615385    630.96
           MFP1   CHEMBL574738             0.590909   1000.00        *
         RDKit7   CHEMBL574738             0.602683   1000.00
        Pattern  CHEMBL1992306             0.855580     15.85
        AP_bits  CHEMBL1992306             0.546539     15.85        *
        TT_bits   CHEMBL574738             0.567164   1000.00        *
            FP2   CHEMBL574738             0.614458   1000.00
  hybridization   CHEMBL574738             0.504902   1000.00
   substructure  CHEMBL1983111             0.882353     39.81        *
          graph   CHEMBL574738             0.733333   1000.00
        pubchem   CHEMBL574738             0.896040   1000.00        *
      cdk_maccs     CHEMBL1336             0.750000    398.11
   klekota_roth  CHEMBL1992581             0.450980    630.96
     *** ki ACTIVITY *** value prediction
     based on 5 locally validated fgps ---> 611.132
["AP_bits", "TT_bits", "MFP1", "substructure", "pubchem"]

12 rank
Mitogen-activated protein kinase kinase kinase kinase 4 : Homo sapiens
     score: 3.483 on CHEMBL6166 based on 5 fingerprints
Fingerprint type         Ligand  Tanimoto Similarity  activity Valid fg
       FeatMFP1  CHEMBL1992581             0.615385   1000.00
           MFP1   CHEMBL574738             0.590909    158.49        *
         RDKit7   CHEMBL574738             0.602683    158.49
        Pattern  CHEMBL1992306             0.855580  10000.00
        AP_bits  CHEMBL1992306             0.546539  10000.00        *
```

```
        TT_bits   CHEMBL574738             0.567164    158.49        *
            FP2   CHEMBL574738             0.614458    158.49
  hybridization   CHEMBL574738             0.504902    158.49
   substructure  CHEMBL1983111             0.882353    100.00        *
          graph   CHEMBL574738             0.733333    158.49
        pubchem   CHEMBL574738             0.896040    158.49        *
      cdk_maccs  CHEMBL1982660             0.754717     63.10
   klekota_roth  CHEMBL1992581             0.450980   1000.00
     *** ki ACTIVITY *** value prediction
     based on 5 locally validated fgps ---> 2115.094
["AP_bits", "TT_bits", "MFP1", "substructure", "pubchem"]

13 rank
Macrophage colony stimulating factor receptor : Homo sapiens
     score: 2.936 on CHEMBL1844 based on 4 fingerprints
Fingerprint type         Ligand  Tanimoto Similarity  activity Valid fg
       FeatMFP1  CHEMBL1992581             0.615385  1584.890
           MFP1   CHEMBL574738             0.590909    50.120        *
         RDKit7   CHEMBL574738             0.602683    50.120
        Pattern  CHEMBL1988838             0.828753   316.230
        AP_bits     CHEMBL1336             0.507795   158.490
        TT_bits   CHEMBL574738             0.567164    50.120        *
            FP2   CHEMBL574738             0.614458    50.120
  hybridization   CHEMBL574738             0.504902    50.120
   substructure  CHEMBL1983111             0.882353    63.100        *
          graph   CHEMBL574738             0.733333    50.120
        pubchem   CHEMBL574738             0.896040    50.120        *
      cdk_maccs     CHEMBL1336             0.750000   158.490
   klekota_roth  CHEMBL1973178             0.460000     5.012
     *** ki ACTIVITY *** value prediction
     based on 4 locally validated fgps ---> 53.365
["TT_bits", "MFP1", "substructure", "pubchem"]

14 rank
Vascular endothelial growth factor receptor 1 : Homo sapiens
     score: 2.936 on CHEMBL1868 based on 4 fingerprints
Fingerprint type         Ligand  Tanimoto Similarity  activity Valid fg
       FeatMFP1  CHEMBL1992581             0.615385   316.230
           MFP1   CHEMBL574738             0.590909    31.620        *
         RDKit7   CHEMBL574738             0.602683    31.620
        Pattern  CHEMBL1988838             0.828753    79.430
        AP_bits     CHEMBL1336             0.507795   316.230
        TT_bits   CHEMBL574738             0.567164    31.620        *
            FP2   CHEMBL574738             0.614458    31.620
  hybridization   CHEMBL574738             0.504902    31.620
   substructure  CHEMBL1983111             0.882353    10.000        *
          graph   CHEMBL574738             0.733333    31.620
        pubchem   CHEMBL574738             0.896040    31.620        *
      cdk_maccs  CHEMBL1982660             0.754717   125.890
   klekota_roth  CHEMBL1973178             0.460000     0.631
     *** ki ACTIVITY *** value prediction
     based on 4 locally validated fgps ---> 26.215
["TT_bits", "MFP1", "substructure", "pubchem"]

15 rank
Platelet-derived growth factor receptor beta : Homo sapiens
     score: 2.936 on CHEMBL1913 based on 4 fingerprints
Fingerprint type         Ligand  Tanimoto Similarity  activity Valid fg
       FeatMFP1  CHEMBL1992581             0.615385  1995.260
           MFP1   CHEMBL574738             0.590909     7.943        *
```

```
         RDKit7   CHEMBL574738             0.602683     7.943
        Pattern   CHEMBL574738             0.827381     7.943
        AP_bits     CHEMBL1336             0.507795     3.981
        TT_bits   CHEMBL574738             0.567164     7.943         *
            FP2   CHEMBL574738             0.614458     7.943
  hybridization   CHEMBL574738             0.504902     7.943
   substructure  CHEMBL1983111             0.882353    63.100         *
          graph   CHEMBL574738             0.733333     7.943
        pubchem   CHEMBL574738             0.896040     7.943         *
      cdk_maccs     CHEMBL1336             0.750000     3.981
   klekota_roth  CHEMBL1992581             0.450980  1995.260
     *** ki ACTIVITY *** value prediction
     based on 4 locally validated fgps ---> 21.732
["TT_bits", "MFP1", "substructure", "pubchem"]

16 rank
Stem cell growth factor receptor : Homo sapiens
     score: 2.936 on CHEMBL1936 based on 4 fingerprints
Fingerprint type         Ligand  Tanimoto Similarity  activity Valid fg
       FeatMFP1  CHEMBL2000934             0.629630   199.530
           MFP1   CHEMBL574738             0.590909  1584.890         *
         RDKit7   CHEMBL574738             0.602683  1584.890
        Pattern   CHEMBL574738             0.827381  1584.890
        AP_bits  CHEMBL2004438             0.492857   158.490
        TT_bits   CHEMBL574738             0.567164  1584.890         *
            FP2   CHEMBL574738             0.614458  1584.890
  hybridization   CHEMBL574738             0.504902  1584.890
   substructure  CHEMBL1983111             0.882353   251.190         *
          graph   CHEMBL574738             0.733333  1584.890
        pubchem   CHEMBL574738             0.896040  1584.890         *
      cdk_maccs  CHEMBL1971943             0.649123   316.230
   klekota_roth  CHEMBL1973178             0.460000     1.585
     *** ki ACTIVITY *** value prediction
     based on 4 locally validated fgps ---> 1251.465
["TT_bits", "MFP1", "substructure", "pubchem"]

17 rank
Platelet-derived growth factor receptor alpha : Homo sapiens
     score: 2.936 on CHEMBL2007 based on 4 fingerprints
Fingerprint type         Ligand  Tanimoto Similarity  activity Valid fg
       FeatMFP1   CHEMBL574738             0.558824     79.43
           MFP1   CHEMBL574738             0.590909     79.43         *
         RDKit7   CHEMBL574738             0.602683     79.43
        Pattern   CHEMBL574738             0.827381     79.43
        AP_bits     CHEMBL1336             0.507795   1000.00
        TT_bits   CHEMBL574738             0.567164     79.43         *
            FP2   CHEMBL574738             0.614458     79.43
  hybridization   CHEMBL574738             0.504902     79.43
   substructure  CHEMBL1983111             0.882353    100.00         *
          graph   CHEMBL574738             0.733333     79.43
        pubchem   CHEMBL574738             0.896040     79.43         *
      cdk_maccs     CHEMBL1336             0.750000   1000.00
   klekota_roth     CHEMBL1336             0.387097   1000.00
     *** ki ACTIVITY *** value prediction
     based on 4 locally validated fgps ---> 84.573
["TT_bits", "MFP1", "substructure", "pubchem"]

18 rank
Death-associated protein kinase 3 : Homo sapiens
     score: 2.936 on CHEMBL2468 based on 4 fingerprints
```

```
Fingerprint type         Ligand  Tanimoto Similarity  activity Valid fg
       FeatMFP1   CHEMBL574738            0.558824   3981.07
           MFP1   CHEMBL574738            0.590909   3981.07         *
         RDKit7   CHEMBL574738            0.602683   3981.07
        Pattern  CHEMBL1988838            0.828753    316.23
        AP_bits   CHEMBL411903            0.476695    794.33
        TT_bits   CHEMBL574738            0.567164   3981.07         *
            FP2   CHEMBL574738            0.614458   3981.07
  hybridization   CHEMBL574738            0.504902   3981.07
   substructure  CHEMBL1983111            0.882353    251.19         *
          graph   CHEMBL574738            0.733333   3981.07
        pubchem   CHEMBL574738            0.896040   3981.07         *
      cdk_maccs   CHEMBL475251            0.698413     79.43
   klekota_roth  CHEMBL1084546            0.388060   1000.00
      *** ki ACTIVITY *** value prediction
      based on 4 locally validated fgps ---> 3048.600
["TT_bits", "MFP1", "substructure", "pubchem"]

19 rank
Vascular endothelial growth factor receptor 2 : Homo sapiens
      score: 2.936 on CHEMBL279 based on 4 fingerprints
Fingerprint type         Ligand  Tanimoto Similarity  activity Valid fg
       FeatMFP1  CHEMBL1254007            0.620690     0.024
           MFP1   CHEMBL574738            0.590909     2.512         *
         RDKit7   CHEMBL574738            0.602683     2.512
        Pattern   CHEMBL400512            0.845011     8.000
        AP_bits   CHEMBL394826            0.509009     9.000
        TT_bits   CHEMBL574738            0.567164     2.512         *
            FP2   CHEMBL574738            0.614458     2.512
  hybridization   CHEMBL574738            0.504902     2.512
   substructure  CHEMBL1983111            0.882353    15.850         *
          graph   CHEMBL574738            0.733333     2.512
        pubchem   CHEMBL574738            0.896040     2.512         *
      cdk_maccs   CHEMBL248978            0.811321    23.000
   klekota_roth   CHEMBL236205            0.489796    80.000
      *** ki ACTIVITY *** value prediction
      based on 4 locally validated fgps ---> 5.846
["TT_bits", "MFP1", "substructure", "pubchem"]

20 rank
Nerve growth factor receptor Trk-A : Homo sapiens
      score: 2.936 on CHEMBL2815 based on 4 fingerprints
Fingerprint type         Ligand  Tanimoto Similarity  activity Valid fg
       FeatMFP1  CHEMBL1992581            0.615385  10000.00
           MFP1   CHEMBL574738            0.590909   1584.89         *
         RDKit7   CHEMBL574738            0.602683   1584.89
        Pattern  CHEMBL1988838            0.828753    125.89
        AP_bits     CHEMBL1336            0.507795   1584.89
        TT_bits   CHEMBL574738            0.567164   1584.89         *
            FP2   CHEMBL574738            0.614458   1584.89
  hybridization   CHEMBL574738            0.504902   1584.89
   substructure  CHEMBL1983111            0.882353    100.00         *
          graph   CHEMBL574738            0.733333   1584.89
        pubchem   CHEMBL574738            0.896040   1584.89         *
      cdk_maccs     CHEMBL1336            0.750000   1584.89
   klekota_roth  CHEMBL1992581            0.450980  10000.00
      *** ki ACTIVITY *** value prediction
      based on 4 locally validated fgps ---> 1213.668
["TT_bits", "MFP1", "substructure", "pubchem"]
```

21 rank
Rho-associated protein kinase 2 : Homo sapiens
score: 2.936 on CHEMBL2973 based on 4 fingerprints

Fingerprint type	Ligand	Tanimoto Similarity	activity	Valid fg
FeatMFP1	CHEMBL574738	0.558824	501.19	
MFP1	CHEMBL574738	0.590909	501.19	*
RDKit7	CHEMBL574738	0.602683	501.19	
Pattern	CHEMBL1997554	0.831111	501.19	
AP_bits	CHEMBL1981410	0.506466	1584.89	
TT_bits	CHEMBL574738	0.567164	501.19	*
FP2	CHEMBL574738	0.614458	501.19	
hybridization	CHEMBL574738	0.504902	501.19	
substructure	CHEMBL1983111	0.882353	158.49	*
graph	CHEMBL574738	0.733333	501.19	
pubchem	CHEMBL574738	0.896040	501.19	*
cdk_maccs	CHEMBL1997554	0.673077	501.19	
klekota_roth	CHEMBL1984363	0.418182	158.49	

*** ki ACTIVITY *** value prediction
based on 4 locally validated fgps ---> 415.515
["TT_bits", "MFP1", "substructure", "pubchem"]

22 rank
Fibroblast growth factor receptor 1 : Homo sapiens
score: 2.936 on CHEMBL3650 based on 4 fingerprints

Fingerprint type	Ligand	Tanimoto Similarity	activity	Valid fg
FeatMFP1	CHEMBL1992581	0.615385	794.33	
MFP1	CHEMBL574738	0.590909	501.19	*
RDKit7	CHEMBL574738	0.602683	501.19	
Pattern	CHEMBL1988838	0.828753	199.53	
AP_bits	CHEMBL1987910	0.478365	1258.93	
TT_bits	CHEMBL574738	0.567164	501.19	*
FP2	CHEMBL574738	0.614458	501.19	
hybridization	CHEMBL574738	0.504902	501.19	
substructure	CHEMBL1983111	0.882353	19.95	*
graph	CHEMBL574738	0.733333	501.19	
pubchem	CHEMBL574738	0.896040	501.19	*
cdk_maccs	CHEMBL1982660	0.754717	3981.07	
klekota_roth	CHEMBL1992581	0.450980	794.33	

*** ki ACTIVITY *** value prediction
based on 4 locally validated fgps ---> 380.880
["TT_bits", "MFP1", "substructure", "pubchem"]

23 rank
cAMP-dependent protein kinase alpha-catalytic subunit : Homo sapiens
score: 2.936 on CHEMBL4101 based on 4 fingerprints

Fingerprint type	Ligand	Tanimoto Similarity	activity	Valid fg
FeatMFP1	CHEMBL3580669	0.592593	3000.00	
MFP1	CHEMBL574738	0.590909	199.53	*
RDKit7	CHEMBL574738	0.602683	199.53	
Pattern	CHEMBL1997554	0.831111	199.53	
AP_bits	CHEMBL1983393	0.445623	794.33	
TT_bits	CHEMBL574738	0.567164	199.53	*
FP2	CHEMBL574738	0.614458	199.53	
hybridization	CHEMBL574738	0.504902	199.53	
substructure	CHEMBL1983111	0.882353	125.89	*
graph	CHEMBL574738	0.733333	199.53	
pubchem	CHEMBL574738	0.896040	199.53	*
cdk_maccs	CHEMBL475251	0.698413	251.19	
klekota_roth	CHEMBL2002723	0.431818	1258.93	

*** ki ACTIVITY *** value prediction

based on 4 locally validated fgps ---> 181.120
["TT_bits", "MFP1", "substructure", "pubchem"]

24 rank
Dual specificity protein kinase CLK4 : Homo sapiens
score: 2.936 on CHEMBL4203 based on 4 fingerprints

Fingerprint type	Ligand	Tanimoto Similarity	activity	Valid fg
FeatMFP1	CHEMBL1986756	0.629630	63.10	
MFP1	CHEMBL574738	0.590909	316.23	*
RDKit7	CHEMBL574738	0.602683	316.23	
Pattern	CHEMBL1988838	0.828753	251.19	
AP_bits	CHEMBL1981410	0.506466	2511.89	
TT_bits	CHEMBL574738	0.567164	316.23	*
FP2	CHEMBL574738	0.614458	316.23	
hybridization	CHEMBL574738	0.504902	316.23	
substructure	CHEMBL1983111	0.882353	199.53	*
graph	CHEMBL574738	0.733333	316.23	
pubchem	CHEMBL574738	0.896040	316.23	*
cdk_maccs	CHEMBL1979176	0.754717	15.85	
klekota_roth	CHEMBL1992581	0.450980	251.19	

*** ki ACTIVITY *** value prediction
based on 4 locally validated fgps ---> 287.055
["TT_bits", "MFP1", "substructure", "pubchem"]

25 rank
Dual specificity protein kinase CLK2 : Homo sapiens
score: 2.936 on CHEMBL4225 based on 4 fingerprints

Fingerprint type	Ligand	Tanimoto Similarity	activity	Valid fg
FeatMFP1	CHEMBL1992581	0.615385	2511.89	
MFP1	CHEMBL574738	0.590909	3162.28	*
RDKit7	CHEMBL574738	0.602683	3162.28	
Pattern	CHEMBL1988838	0.828753	398.11	
AP_bits	CHEMBL1981410	0.506466	5011.87	
TT_bits	CHEMBL574738	0.567164	3162.28	*
FP2	CHEMBL574738	0.614458	3162.28	
hybridization	CHEMBL574738	0.504902	3162.28	
substructure	CHEMBL1983111	0.882353	630.96	*
graph	CHEMBL574738	0.733333	3162.28	
pubchem	CHEMBL574738	0.896040	3162.28	*
cdk_maccs	CHEMBL1979176	0.754717	1995.26	
klekota_roth	CHEMBL1992581	0.450980	2511.89	

*** ki ACTIVITY *** value prediction
based on 4 locally validated fgps ---> 2529.450
["TT_bits", "MFP1", "substructure", "pubchem"]

26 rank
Maternal embryonic leucine zipper kinase : Homo sapiens
score: 2.936 on CHEMBL4578 based on 4 fingerprints

Fingerprint type	Ligand	Tanimoto Similarity	activity	Valid fg
FeatMFP1	CHEMBL574738	0.558824	125.89	
MFP1	CHEMBL574738	0.590909	125.89	*
RDKit7	CHEMBL574738	0.602683	125.89	
Pattern	CHEMBL1988838	0.828753	1258.93	
AP_bits	CHEMBL1981410	0.506466	630.96	
TT_bits	CHEMBL574738	0.567164	125.89	*
FP2	CHEMBL574738	0.614458	125.89	
hybridization	CHEMBL574738	0.504902	125.89	
substructure	CHEMBL1983111	0.882353	199.53	*
graph	CHEMBL574738	0.733333	125.89	
pubchem	CHEMBL574738	0.896040	125.89	*

```
       cdk_maccs  CHEMBL2001239             0.672131    630.96
    klekota_roth  CHEMBL1084546             0.388060     79.43
      *** ki ACTIVITY *** value prediction
      based on 4 locally validated fgps ---> 144.300
["TT_bits", "MFP1", "substructure", "pubchem"]

27 rank
Mitogen-activated protein kinase kinase kinase kinase 2 : Homo sapiens
      score: 2.936 on CHEMBL5330 based on 4 fingerprints
Fingerprint type         Ligand  Tanimoto Similarity  activity Valid fg
        FeatMFP1  CHEMBL1992581             0.615385    251.19
            MFP1   CHEMBL574738             0.590909     79.43         *
          RDKit7   CHEMBL574738             0.602683     79.43
         Pattern  CHEMBL1988838             0.828753    398.11
         AP_bits  CHEMBL1981410             0.506466   1584.89
         TT_bits   CHEMBL574738             0.567164     79.43         *
             FP2   CHEMBL574738             0.614458     79.43
   hybridization   CHEMBL574738             0.504902     79.43
    substructure  CHEMBL1983111             0.882353     39.81         *
           graph   CHEMBL574738             0.733333     79.43
         pubchem   CHEMBL574738             0.896040     79.43         *
       cdk_maccs  CHEMBL1979176             0.754717   3162.28
    klekota_roth  CHEMBL1992581             0.450980    251.19
      *** ki ACTIVITY *** value prediction
      based on 4 locally validated fgps ---> 69.525
["TT_bits", "MFP1", "substructure", "pubchem"]

28 rank
Leukocyte tyrosine kinase receptor : Homo sapiens
      score: 2.936 on CHEMBL5627 based on 4 fingerprints
Fingerprint type         Ligand  Tanimoto Similarity  activity Valid fg
        FeatMFP1  CHEMBL1992581             0.615385   1995.26
            MFP1   CHEMBL574738             0.590909    630.96         *
          RDKit7   CHEMBL574738             0.602683    630.96
         Pattern   CHEMBL574738             0.827381    630.96
         AP_bits  CHEMBL1965033             0.478571    794.33
         TT_bits   CHEMBL574738             0.567164    630.96         *
             FP2   CHEMBL574738             0.614458    630.96
   hybridization   CHEMBL574738             0.504902    630.96
    substructure  CHEMBL1983111             0.882353     39.81         *
           graph   CHEMBL574738             0.733333    630.96
         pubchem   CHEMBL574738             0.896040    630.96         *
       cdk_maccs   CHEMBL574738             0.638889    630.96
    klekota_roth  CHEMBL1992581             0.450980   1995.26
      *** ki ACTIVITY *** value prediction
      based on 4 locally validated fgps ---> 483.173
["TT_bits", "MFP1", "substructure", "pubchem"]

29 rank
Tyrosine-protein kinase FYN : Homo sapiens
      score: 2.601 on CHEMBL1841 based on 4 fingerprints
Fingerprint type         Ligand  Tanimoto Similarity  activity Valid fg
        FeatMFP1  CHEMBL1992581             0.615385   1584.89
            MFP1   CHEMBL574738             0.590909    158.49         *
          RDKit7   CHEMBL574738             0.602683    158.49
         Pattern  CHEMBL1992306             0.855580    251.19
         AP_bits  CHEMBL1992306             0.546539    251.19         *
         TT_bits   CHEMBL574738             0.567164    158.49         *
             FP2   CHEMBL574738             0.614458    158.49
   hybridization   CHEMBL574738             0.504902    158.49
```

```
   substructure  CHEMBL1084546             0.764706   1995.26
          graph   CHEMBL574738             0.733333    158.49
        pubchem   CHEMBL574738             0.896040    158.49        *
      cdk_maccs  CHEMBL1992306             0.740741    251.19
   klekota_roth  CHEMBL1973178             0.460000     79.43
      *** ki ACTIVITY *** value prediction
      based on 4 locally validated fgps ---> 181.665
["AP_bits", "TT_bits", "MFP1", "pubchem"]
```

SwissTargetPrediction report:

Reference:
Gfeller D., Michielin O. & Zoete V.
Shaping the interaction landscape of bioactive molecules, *Bioinformatics* (2013) 29:3073-3079.

Query Molecule

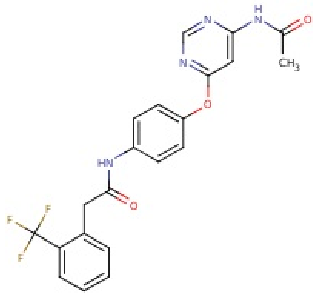

Frequency of Target Class

Target	Uniprot ID	Gene code	ChEMBL ID	Probability	# sim. cmpds (3D / 2D)	Target Class
Vascular endothelial growth factor receptor 1	P17948	FLT1	CHEMBL1868		287 / 90	Tyr Kinase
Vascular endothelial growth factor receptor 3	P35916	FLT4	CHEMBL1955		283 / 90	Tyr Kinase
Vascular endothelial growth factor receptor 2 *(by homology)*	P35968	KDR	CHEMBL279		283 / 90	Tyr Kinase
Macrophage colony-stimulating factor 1 receptor	P07333	CSF1R	CHEMBL1844		69 / 26	Tyr Kinase
Mast/stem cell growth factor receptor Kit	P10721	KIT	CHEMBL1936		69 / 26	Tyr Kinase
Receptor-type tyrosine-protein kinase FLT3	P36888	FLT3	CHEMBL1974		30 / 26	Tyr Kinase
Platelet-derived growth factor receptor beta	P09619	PDGFRB	CHEMBL1913		21 / 36	Tyr Kinase
Platelet-derived growth factor receptor alpha *(by homology)*	P16234	PDGFRA	CHEMBL2007		21 / 36	Tyr Kinase
Serine/threonine-protein kinase PLK1	P53350	PLK1	CHEMBL3024		60 / 1	Ser_Thr Kinase
Serine/threonine-protein kinase PLK3	Q9H4B4	PLK3	CHEMBL4897		60 / 1	Ser_Thr Kinase
Serine/threonine-protein kinase PLK2 *(by homology)*	Q9NYY3	PLK2	CHEMBL5938		60 / 1	Ser_Thr Kinase
RAF proto-oncogene serine/threonine-protein kinase	P04049	RAF1	CHEMBL1906		144 / 9	Ser_Thr_Tyr Kinase
Serine/threonine-protein kinase B-raf	P15056	BRAF	CHEMBL5145		144 / 9	Ser_Thr_Tyr Kinase
Serine/threonine-protein kinase A-Raf *(by homology)*	P10398	ARAF	CHEMBL1169596		144 / 9	Ser_Thr Kinase
Focal adhesion kinase 1	Q05397	PTK2	CHEMBL2695		44 / 2	Tyr Kinase

Polypharmacology Browser 2 Prediction:

Targets predicted using NN(ECfp4) + NB(ECfp4). Save Table

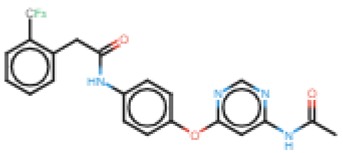

Query molecule

Target class overview

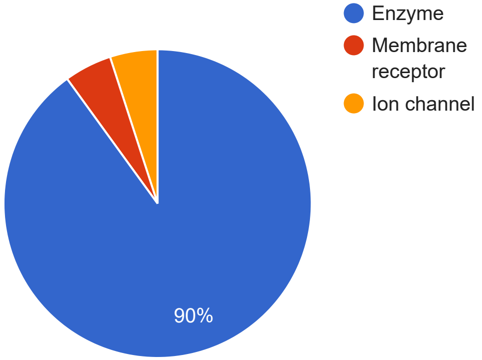

Rank	ChEMBL ID	Common name	Nearest neighbours
1	CHEMBL279 (https://www.ebi.ac.uk/chembl/target/inspect/CHEMBL279)	Vascular endothelial growth factor receptor 2	Show NN
2	CHEMBL203 (https://www.ebi.ac.uk/chembl/target/inspect/CHEMBL203)	Epidermal growth factor receptor erbB1	Show NN
3	CHEMBL2276 (https://www.ebi.ac.uk/chembl/target/inspect/CHEMBL2276)	c-Jun N-terminal kinase 1	Show NN
4	CHEMBL2637 (https://www.ebi.ac.uk/chembl/target/inspect/CHEMBL2637)	c-Jun N-terminal kinase 3	Show NN
5	CHEMBL1824 (https://www.ebi.ac.uk/chembl/target/inspect/CHEMBL1824)	Receptor protein-tyrosine kinase erbB-2	Show NN
6	CHEMBL1974 (https://www.ebi.ac.uk/chembl/target/inspect/CHEMBL1974)	Tyrosine-protein kinase receptor FLT3	Show NN
7	CHEMBL1844 (https://www.ebi.ac.uk/chembl/target/inspect/CHEMBL1844)	Macrophage colony stimulating factor receptor	Show NN
8	CHEMBL3717 (https://www.ebi.ac.uk/chembl/target/inspect/CHEMBL3717)	Hepatocyte growth factor receptor	Show NN
9	CHEMBL5102 (https://www.ebi.ac.uk/chembl/target/inspect/CHEMBL5102)	Vanilloid receptor	Show NN
10	CHEMBL4722 (https://www.ebi.ac.uk/chembl/target/inspect/CHEMBL4722)	Serine/threonine-protein kinase Aurora-A	Show NN
11	CHEMBL258 (https://www.ebi.ac.uk/chembl/target/inspect/CHEMBL258)	Tyrosine-protein kinase LCK	Show NN
12	CHEMBL260 (https://www.ebi.ac.uk/chembl/target/inspect/CHEMBL260)	MAP kinase p38 alpha	Show NN
13	CHEMBL1868 (https://www.ebi.ac.uk/chembl/target/inspect/CHEMBL1868)	Vascular endothelial growth factor receptor 1	Show NN
14	CHEMBL256 (https://www.ebi.ac.uk/chembl/target/inspect/CHEMBL256)	Adenosine A3 receptor	Show NN
15	CHEMBL267 (https://www.ebi.ac.uk/chembl/target/inspect/CHEMBL267)	Tyrosine-protein kinase SRC	Show NN
16	CHEMBL1955 (https://www.ebi.ac.uk/chembl/target/inspect/CHEMBL1955)	Vascular endothelial growth factor receptor 3	Show NN
17	CHEMBL2815 (https://www.ebi.ac.uk/chembl/target/inspect/CHEMBL2815)	Nerve growth factor receptor Trk-A	Show NN
18	CHEMBL261 (https://www.ebi.ac.uk/chembl/target/inspect/CHEMBL261)	Carbonic anhydrase I	Show NN

19	CHEMBL205 (https://www.ebi.ac.uk/chembl/target/inspect/CHEMBL205)	Carbonic anhydrase II	Show NN
20	CHEMBL3229 (https://www.ebi.ac.uk/chembl/target/inspect/CHEMBL3229)	Anandamide amidohydrolase	Show NN
