# Supplementary material for: Accelerating Drug Discovery by Early Protein Drug Target Prediction Based on a Multi-Fingerprint Similarity Search
Source: Molecules. 2019 Jun 14;24(12):2233. doi: 10.3390/molecules24122233 (PMC6631269; doi:10.3390/molecules24122233)
Supplement: Supplementary file 1 [file molecules-24-02233-s001.zip › File_S2.zip!/File_S2/Target_Prediction_compound#36.pdf]

MuSSel not predicted:

SwissTargetPrediction report:

Reference:
Gfeller D., Michielin O. & Zoete V.
Shaping the interaction landscape of bioactive molecules, *Bioinformatics* (2013) 29:3073-3079.

Query Molecule

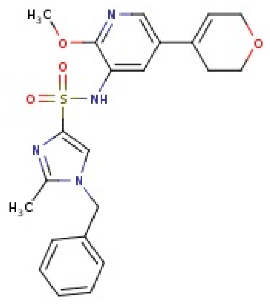

Frequency of Target Class

Target	Uniprot ID	Gene code	ChEMBL ID	Probability	# sim. cmpds (3D / 2D)	Target Class
Vasopressin V2 receptor *(by homology)*	P30518	AVPR2	CHEMBL1790		56 / 2	Membrane receptor
Oxytocin receptor	P30559	OXTR	CHEMBL2049		87 / 2	Membrane receptor
Vasopressin V1a receptor	P37288	AVPR1A	CHEMBL1889		52 / 2	Membrane receptor
Vasopressin V1b receptor	P47901	AVPR1B	CHEMBL1921		46 / 2	Membrane receptor
Pepsin A-5	P0DJD9	PGA5	CHEMBL3295		6 / 1	Aspartic Protease
Cathepsin E form I	P14091	CTSE	CHEMBL3092		6 / 1	Aspartic Protease
Pepsin A-4 *(by homology)*	P0DJD7	PGA4			6 / 1	Aspartic Protease
Pepsin A-3 *(by homology)*	P0DJD8	PGA3			6 / 1	Aspartic Protease
Gastricsin *(by homology)*	P20142	PGC	CHEMBL2136		6 / 1	Aspartic Protease
Mitogen-activated protein kinase 8 *(by homology)*	P45983	MAPK8	CHEMBL2276		401 / 0	Ser_Thr Kinase
Mitogen-activated protein kinase 9 *(by homology)*	P45984	MAPK9	CHEMBL4179		401 / 0	Ser_Thr Kinase
Mitogen-activated protein kinase 10 *(by homology)*	P53779	MAPK10	CHEMBL2637		401 / 0	Ser_Thr Kinase
Mitogen-activated protein kinase 11 *(by homology)*	Q15759	MAPK11	CHEMBL3961		413 / 0	Ser_Thr Kinase
Mitogen-activated protein kinase 14	Q16539	MAPK14	CHEMBL260		413 / 0	Ser_Thr Kinase
Platelet-activating factor receptor	P25105	PTAFR	CHEMBL250		42 / 0	Membrane receptor

Polypharmacology Browser 2 Prediction:

Targets predicted using NN(ECfp4) + NB(ECfp4). Save Table

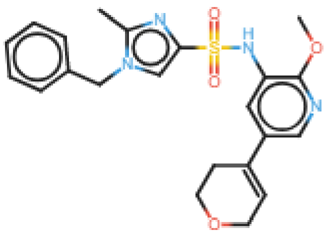

Query molecule

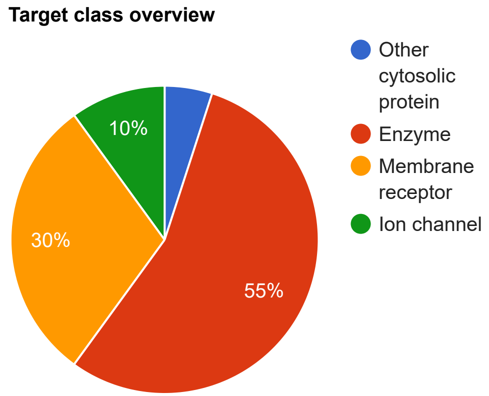

Rank	ChEMBL ID	Common name	Nearest neighbours
1	CHEMBL4005 (https://www.ebi.ac.uk/chembl/target/inspect/CHEMBL4005)	PI3-kinase p110-alpha subunit	Show NN
2	CHEMBL2842 (https://www.ebi.ac.uk/chembl/target/inspect/CHEMBL2842)	Serine/threonine-protein kinase mTOR	Show NN
3	CHEMBL3130 (https://www.ebi.ac.uk/chembl/target/inspect/CHEMBL3130)	PI3-kinase p110-delta subunit	Show NN
4	CHEMBL3145 (https://www.ebi.ac.uk/chembl/target/inspect/CHEMBL3145)	PI3-kinase p110-beta subunit	Show NN
5	CHEMBL3267 (https://www.ebi.ac.uk/chembl/target/inspect/CHEMBL3267)	PI3-kinase p110-gamma subunit	Show NN
6	CHEMBL3371 (https://www.ebi.ac.uk/chembl/target/inspect/CHEMBL3371)	Serotonin 6 (5-HT6) receptor	Show NN
7	CHEMBL4409 (https://www.ebi.ac.uk/chembl/target/inspect/CHEMBL4409)	Phosphodiesterase 10A	Show NN
8	CHEMBL3667 (https://www.ebi.ac.uk/chembl/target/inspect/CHEMBL3667)	PI4-kinase alpha subunit	Show NN
9	CHEMBL4296 (https://www.ebi.ac.uk/chembl/target/inspect/CHEMBL4296)	Sodium channel protein type IX alpha subunit	Show NN
10	CHEMBL217 (https://www.ebi.ac.uk/chembl/target/inspect/CHEMBL217)	Dopamine D2 receptor	Show NN
11	CHEMBL6140 (https://www.ebi.ac.uk/chembl/target/inspect/CHEMBL6140)	cAMP and cAMP-inhibited cGMP 3',5'-cyclic phosphodiesterase 10A	Show NN
12	CHEMBL3717 (https://www.ebi.ac.uk/chembl/target/inspect/CHEMBL3717)	Hepatocyte growth factor receptor	Show NN
13	CHEMBL218 (https://www.ebi.ac.uk/chembl/target/inspect/CHEMBL218)	Cannabinoid CB1 receptor	Show NN

14	CHEMBL203 (https://www.ebi.ac.uk/chembl/target/inspect/CHEMBL203)	Epidermal growth factor receptor erbB1	Show NN
15	CHEMBL240 (https://www.ebi.ac.uk/chembl/target/inspect/CHEMBL240)	HERG	Show NN
16	CHEMBL4550 (https://www.ebi.ac.uk/chembl/target/inspect/CHEMBL4550)	5-lipoxygenase activating protein	Show NN
17	CHEMBL5071 (https://www.ebi.ac.uk/chembl/target/inspect/CHEMBL5071)	G protein-coupled receptor 44	Show NN
18	CHEMBL253 (https://www.ebi.ac.uk/chembl/target/inspect/CHEMBL253)	Cannabinoid CB2 receptor	Show NN
19	CHEMBL2599 (https://www.ebi.ac.uk/chembl/target/inspect/CHEMBL2599)	Tyrosine-protein kinase SYK	Show NN
20	CHEMBL234 (https://www.ebi.ac.uk/chembl/target/inspect/CHEMBL234)	Dopamine D3 receptor	Show NN
