# Supplementary material for: Accelerating Drug Discovery by Early Protein Drug Target Prediction Based on a Multi-Fingerprint Similarity Search
Source: Molecules. 2019 Jun 14;24(12):2233. doi: 10.3390/molecules24122233 (PMC6631269; doi:10.3390/molecules24122233)
Supplement: Supplementary file 1 [file molecules-24-02233-s001.zip › File_S2.zip!/File_S2/Target_Prediction_compound#4.pdf]

MuSSel Prediction IC_{50}:

```
1 rank
Transient receptor potential cation channel subfamily V member 4 : Homo
sapiens
      score: 9.515 on CHEMBL3119 based on 12 fingerprints
Fingerprint type         Ligand  Tanimoto Similarity  activity Valid fg
       FeatMFP1  CHEMBL3218112             0.863636      12.9        *
           MFP1  CHEMBL3218118             0.756098       3.8        *
         RDKit7  CHEMBL3218118             0.764706       3.8        *
        Pattern  CHEMBL3218118             0.886756       3.8        *
        AP_bits  CHEMBL3218118             0.621134       3.8        *
        TT_bits  CHEMBL3218118             0.605634       3.8        *
            FP2  CHEMBL3218118             0.833333       3.8        *
  hybridization  CHEMBL3218118             0.720207       3.8        *
   substructure  CHEMBL3218116             0.894737       3.4        *
          graph  CHEMBL3218118             0.683258       3.8
        pubchem  CHEMBL3218112             0.918750      12.9        *
      cdk_maccs  CHEMBL3218116             0.875000       3.4        *
   klekota_roth  CHEMBL3218118             0.775281       3.8        *
      *** ic50 ACTIVITY *** value prediction
      based on 12 locally validated fgps ---> 8.257
["TT_bits", "substructure", "cdk_maccs", "Pattern", "FP2", "MFP1",
"klekota_roth", "pubchem", "AP_bits", "FeatMFP1", "RDKit7",
"hybridization"]

2 rank
Transient receptor potential cation channel subfamily M member 8 : Homo
sapiens
      score: 9.508 on CHEMBL1075319 based on 12 fingerprints
Fingerprint type         Ligand  Tanimoto Similarity  activity Valid fg
       FeatMFP1  CHEMBL3218114             0.863636    4800.0        *
           MFP1  CHEMBL3218118             0.756098    9580.0        *
         RDKit7  CHEMBL3218118             0.764706    9580.0        *
        Pattern  CHEMBL3218118             0.886756    9580.0        *
        AP_bits  CHEMBL3218118             0.621134    9580.0        *
        TT_bits  CHEMBL3218118             0.605634    9580.0        *
            FP2  CHEMBL3218118             0.833333    9580.0        *
  hybridization  CHEMBL3218118             0.720207    9580.0        *
   substructure  CHEMBL3218118             0.894737    9580.0        *
          graph  CHEMBL3218118             0.683258    9580.0
        pubchem  CHEMBL3218118             0.911950    9580.0        *
      cdk_maccs  CHEMBL3218118             0.875000    9580.0        *
   klekota_roth  CHEMBL3218118             0.775281    9580.0        *
      *** ic50 ACTIVITY *** value prediction
      based on 12 locally validated fgps ---> 8262.064
["TT_bits", "substructure", "cdk_maccs", "Pattern", "FP2", "MFP1",
"klekota_roth", "pubchem", "AP_bits", "FeatMFP1", "RDKit7",
"hybridization"]

3 rank
HERG : Homo sapiens
      score: 9.508 on CHEMBL240 based on 12 fingerprints
Fingerprint type         Ligand  Tanimoto Similarity  activity Valid fg
       FeatMFP1  CHEMBL3218118             0.863636    9670.0        *
           MFP1  CHEMBL3218118             0.756098    9670.0        *
         RDKit7  CHEMBL3218118             0.764706    9670.0        *
        Pattern  CHEMBL3218118             0.886756    9670.0        *
        AP_bits  CHEMBL3218118             0.621134    9670.0        *
        TT_bits  CHEMBL3218118             0.605634    9670.0        *
            FP2  CHEMBL3218118             0.833333    9670.0        *
  hybridization  CHEMBL3218118             0.720207    9670.0        *
```

```
   substructure  CHEMBL3218118             0.894737    9670.0        *
          graph  CHEMBL3218118             0.683258    9670.0
        pubchem  CHEMBL3218118             0.911950    9670.0        *
      cdk_maccs  CHEMBL3218118             0.875000    9670.0        *
   klekota_roth  CHEMBL3218118             0.775281    9670.0        *
      *** ic50 ACTIVITY *** value prediction
      based on 12 locally validated fgps ---> 9670.000
["TT_bits", "substructure", "cdk_maccs", "Pattern", "FP2", "MFP1",
"klekota_roth", "pubchem", "AP_bits", "FeatMFP1", "RDKit7",
"hybridization"]

4 rank
Transient receptor potential cation channel subfamily V member 4 : Rattus
norvegicus
      score: 9.508 on CHEMBL2775 based on 12 fingerprints
Fingerprint type         Ligand  Tanimoto Similarity  activity Valid fg
       FeatMFP1  CHEMBL3218114             0.863636    1150.0        *
           MFP1  CHEMBL3218118             0.756098      34.1        *
         RDKit7  CHEMBL3218118             0.764706      34.1        *
        Pattern  CHEMBL3218118             0.886756      34.1        *
        AP_bits  CHEMBL3218118             0.621134      34.1        *
        TT_bits  CHEMBL3218118             0.605634      34.1        *
            FP2  CHEMBL3218118             0.833333      34.1        *
  hybridization  CHEMBL3218118             0.720207      34.1        *
   substructure  CHEMBL3218118             0.894737      34.1        *
          graph  CHEMBL3218118             0.683258      34.1
        pubchem  CHEMBL3218118             0.911950      34.1        *
      cdk_maccs  CHEMBL3218118             0.875000      34.1        *
   klekota_roth  CHEMBL3218118             0.775281      34.1        *
      *** ic50 ACTIVITY *** value prediction
      based on 12 locally validated fgps ---> 341.775
["TT_bits", "substructure", "cdk_maccs", "Pattern", "FP2", "MFP1",
"klekota_roth", "pubchem", "AP_bits", "FeatMFP1", "RDKit7",
"hybridization"]
```

SwissTargetPrediction report:

Reference:
Gfeller D., Michielin O. & Zoete V.
Shaping the interaction landscape of bioactive molecules, *Bioinformatics* (2013) 29:3073-3079.

Query Molecule

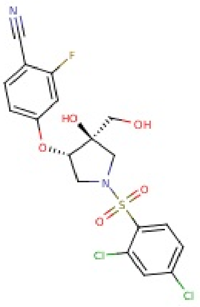

Frequency of Target Class

Target	Uniprot ID	Gene code	ChEMBL ID	Probability	# sim. cmpds (3D / 2D)	Target Class
5-hydroxytryptamine receptor 1A	P08908	HTR1A	CHEMBL214		26 / 31	Membrane receptor
5-hydroxytryptamine receptor 1B *(by homology)*	P28222	HTR1B	CHEMBL1898		29 / 32	Membrane receptor
Potassium voltage-gated channel subfamily H member 2	Q12809	KCNH2	CHEMBL240		23 / 10	Ion channel
Potassium voltage-gated channel subfamily H member 6 *(by homology)*	Q9H252	KCNH6			23 / 10	Ion channel
Potassium voltage-gated channel subfamily H member 7 *(by homology)*	Q9NS40	KCNH7			23 / 10	Ion channel
22 kDa interstitial collagenase *(by homology)*	P03956	MMP1	CHEMBL332		135 / 79	Metallo Protease
Stromelysin-1	P08254	MMP3	CHEMBL283		135 / 79	Metallo Protease
Macrophage metalloelastase *(by homology)*	P39900	MMP12	CHEMBL4393		126 / 79	Metallo Protease
Collagenase 3	P45452	MMP13	CHEMBL280		126 / 79	Metallo Protease
Stromelysin-2 *(by homology)*	P09238	MMP10	CHEMBL4270		135 / 79	Metallo Protease
Matrix metalloproteinase-27 *(by homology)*	Q9H306	MMP27			135 / 79	Metallo Protease
Androgen receptor	P10275	AR	CHEMBL1871		33 / 1	Transcription Factor
5-hydroxytryptamine receptor 7	P34969	HTR7	CHEMBL3155		2 / 36	Membrane receptor
Microtubule-associated protein tau	P10636	MAPT	CHEMBL1293224		105 / 1	Unclassified
PEX	P08253	MMP2	CHEMBL333		71 / 55	Metallo Protease

Polypharmacology Browser 2 not predicted
