# Supplementary material for: Accelerating Drug Discovery by Early Protein Drug Target Prediction Based on a Multi-Fingerprint Similarity Search
Source: Molecules. 2019 Jun 14;24(12):2233. doi: 10.3390/molecules24122233 (PMC6631269; doi:10.3390/molecules24122233)
Supplement: Supplementary file 1 [file molecules-24-02233-s001.zip › File_S2.zip!/File_S2/Target_Prediction_compound#5.pdf]

MuSSel Prediction k_i:

```
1 rank
Dopamine D2 receptor : Rattus norvegicus
      score: 4.894 on CHEMBL339 based on 6 fingerprints
Fingerprint type         Ligand  Tanimoto Similarity   activity Valid fg
       FeatMFP1  CHEMBL1096620             0.666667   1800.000
           MFP1   CHEMBL337149             0.687500      0.035        *
         RDKit7   CHEMBL337149             0.890785      0.035        *
        Pattern   CHEMBL444149             0.604149  17158.000
        AP_bits   CHEMBL313744             0.427068      1.400
        TT_bits   CHEMBL337149             0.457746      0.035
            FP2   CHEMBL337149             0.774194      0.035        *
  hybridization   CHEMBL337149             0.763975      0.035        *
   substructure   CHEMBL134400             0.869565      0.023        *
          graph   CHEMBL134400             0.785714      0.023
        pubchem   CHEMBL137309             0.818713      0.043
      cdk_maccs   CHEMBL335606             0.907895      0.087        *
   klekota_roth   CHEMBL135917             0.641379      0.029
      *** ki ACTIVITY *** value prediction
      based on 6 locally validated fgps ---> 0.043
["RDKit7", "substructure", "MFP1", "FP2", "cdk_maccs", "hybridization"]

2 rank
Dopamine D2 receptor : Homo sapiens
      score: 4.739 on CHEMBL217 based on 6 fingerprints
Fingerprint type         Ligand  Tanimoto Similarity  activity Valid fg
       FeatMFP1  CHEMBL2165130             0.676471   235.000
           MFP1   CHEMBL267932             0.645833     0.133        *
         RDKit7   CHEMBL267932             0.797782     0.133        *
        Pattern  CHEMBL3265044             0.863111    12.960        *
        AP_bits  CHEMBL3265048             0.688824    23.780        *
        TT_bits    CHEMBL92649             0.423313     0.660
            FP2   CHEMBL267932             0.690323     0.133
  hybridization    CHEMBL92649             0.683849     0.660
   substructure   CHEMBL267930             0.869565     0.060        *
          graph   CHEMBL267932             0.727273     0.133
        pubchem   CHEMBL267932             0.795322     0.133
      cdk_maccs   CHEMBL320173             0.873418     3.100        *
   klekota_roth   CHEMBL267930             0.612676     0.060
      *** ki ACTIVITY *** value prediction
      based on 6 locally validated fgps ---> 7.279
["substructure", "Pattern", "MFP1", "AP_bits", "RDKit7", "cdk_maccs"]

3 rank
Dopamine D3 receptor : Homo sapiens
      score: 3.788 on CHEMBL234 based on 5 fingerprints
Fingerprint type         Ligand  Tanimoto Similarity  activity Valid fg
       FeatMFP1     CHEMBL1112             0.636364     1.000
           MFP1   CHEMBL267932             0.645833     0.265        *
         RDKit7   CHEMBL267932             0.797782     0.265        *
        Pattern  CHEMBL1909065             0.723330     9.336
        AP_bits  CHEMBL1916550             0.617234    12.000        *
        TT_bits   CHEMBL267932             0.365517     0.265
            FP2   CHEMBL267932             0.690323     0.265
  hybridization  CHEMBL1813589             0.598940   427.000
   substructure   CHEMBL267930             0.869565     0.120        *
          graph   CHEMBL267932             0.727273     0.265
        pubchem   CHEMBL267932             0.795322     0.265
      cdk_maccs   CHEMBL267932             0.857143     0.265        *
   klekota_roth   CHEMBL267930             0.612676     0.120
      *** ki ACTIVITY *** value prediction
```

```
      based on 5 locally validated fgps ---> 1.593
["AP_bits", "RDKit7", "MFP1", "substructure", "cdk_maccs"]

4 rank
Nociceptin receptor : Homo sapiens
      score: 3.220 on CHEMBL2014 based on 4 fingerprints
Fingerprint type         Ligand  Tanimoto Similarity  activity Valid fg
       FeatMFP1   CHEMBL283432             0.666667       8.5
           MFP1  CHEMBL2088036             0.509434      12.9
         RDKit7    CHEMBL26483             0.774554      15.0         *
        Pattern  CHEMBL3810104             0.878706      38.0         *
        AP_bits  CHEMBL3808398             0.683515      27.0         *
        TT_bits    CHEMBL26483             0.375000      15.0
            FP2    CHEMBL46516             0.635220     500.0
  hybridization    CHEMBL26987             0.630237       7.2
   substructure    CHEMBL26483             0.791667      15.0
          graph  CHEMBL2088036             0.746835      12.9
        pubchem  CHEMBL2088036             0.752809      12.9
      cdk_maccs   CHEMBL516077             0.883117      19.5         *
   klekota_roth  CHEMBL2088036             0.629139      12.9
      *** ki ACTIVITY *** value prediction
      based on 4 locally validated fgps ---> 25.183
["Pattern", "RDKit7", "cdk_maccs", "AP_bits"]

5 rank
Mu opioid receptor : Homo sapiens
      score: 3.220 on CHEMBL233 based on 4 fingerprints
Fingerprint type         Ligand  Tanimoto Similarity  activity Valid fg
       FeatMFP1   CHEMBL283432             0.666667       7.5
           MFP1   CHEMBL516077             0.464286     683.0
         RDKit7    CHEMBL26483             0.774554      61.0         *
        Pattern  CHEMBL3810104             0.878706      15.0         *
        AP_bits  CHEMBL3808398             0.683515      15.0         *
        TT_bits    CHEMBL26483             0.375000      61.0
            FP2    CHEMBL26483             0.626374      61.0
  hybridization    CHEMBL26987             0.630237       3.3
   substructure    CHEMBL26483             0.791667      61.0
          graph   CHEMBL511649             0.716129      14.0
        pubchem    CHEMBL26483             0.735450      61.0
      cdk_maccs   CHEMBL516077             0.883117     683.0         *
   klekota_roth   CHEMBL516077             0.596154     683.0
      *** ki ACTIVITY *** value prediction
      based on 4 locally validated fgps ---> 198.087
["Pattern", "RDKit7", "cdk_maccs", "AP_bits"]

6 rank
Kappa opioid receptor : Homo sapiens
      score: 3.220 on CHEMBL237 based on 4 fingerprints
Fingerprint type         Ligand  Tanimoto Similarity  activity Valid fg
       FeatMFP1   CHEMBL283432             0.666667      17.5
           MFP1   CHEMBL516077             0.464286    2274.0
         RDKit7    CHEMBL26483             0.774554      28.8         *
        Pattern  CHEMBL3810104             0.878706      22.0         *
        AP_bits  CHEMBL3808398             0.683515      84.0         *
        TT_bits    CHEMBL26483             0.375000      28.8
            FP2    CHEMBL26483             0.626374      28.8
  hybridization    CHEMBL26987             0.630237      16.4
   substructure    CHEMBL26483             0.791667      28.8
          graph   CHEMBL511649             0.716129      56.0
        pubchem    CHEMBL26483             0.735450      28.8
```

cdk_maccs	CHEMBL516077	0.883117	2274.0	*
klekota_roth	CHEMBL516077	0.596154	2274.0	

*** ki ACTIVITY *** value prediction
based on 4 locally validated fgps ---> 550.986
["Pattern", "RDKit7", "cdk_maccs", "AP_bits"]

7 rank
Delta opioid receptor : Homo sapiens
score: 3.206 on CHEMBL236 based on 4 fingerprints

Fingerprint type	Ligand	Tanimoto Similarity	activity	Valid fg
FeatMFP1	CHEMBL283432	0.666667	240.0	
MFP1	CHEMBL391631	0.444444	320.0	
RDKit7	CHEMBL26483	0.774554	510.0	*
Pattern	CHEMBL3752798	0.899816	0.6	*
AP_bits	CHEMBL3616885	0.685634	7.0	*
TT_bits	CHEMBL26483	0.375000	510.0	
FP2	CHEMBL26483	0.626374	510.0	
hybridization	CHEMBL26987	0.630237	285.0	
substructure	CHEMBL26483	0.791667	510.0	
graph	CHEMBL511649	0.716129	795.0	
pubchem	CHEMBL26483	0.735450	510.0	
cdk_maccs	CHEMBL516024	0.846154	28990.0	*
klekota_roth	CHEMBL516024	0.529032	28990.0	

*** ki ACTIVITY *** value prediction
based on 4 locally validated fgps ---> 7390.652
["Pattern", "RDKit7", "cdk_maccs", "AP_bits"]

SwissTargetPrediction report:

Reference:
Gfeller D., Michielin O. & Zoete V.
Shaping the interaction landscape of bioactive molecules, *Bioinformatics* (2013) 29:3073-3079.

Query Molecule

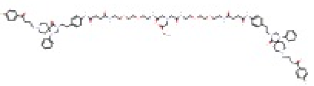

Frequency of Target Class

Target	Uniprot ID	Gene code	ChEMBL ID	Probability	# sim. cmpds (3D / 2D)	Target Class
D(2) dopamine receptor *(by homology)*	P14416	DRD2	CHEMBL217		0 / 81	Membrane receptor
D(3) dopamine receptor *(by homology)*	P35462	DRD3	CHEMBL234		0 / 38	Membrane receptor
D(1A) dopamine receptor	P21728	DRD1	CHEMBL2056		0 / 4	Membrane receptor
D(4) dopamine receptor	P21917	DRD4	CHEMBL219		0 / 26	Membrane receptor
Alpha-1D adrenergic receptor *(by homology)*	P25100	ADRA1D	CHEMBL223		0 / 14	Membrane receptor
5-hydroxytryptamine receptor 2A	P28223	HTR2A	CHEMBL224		0 / 40	Membrane receptor
5-hydroxytryptamine receptor 2C	P28335	HTR2C	CHEMBL225		0 / 16	Membrane receptor
5-hydroxytryptamine receptor 7	P34969	HTR7	CHEMBL3155		0 / 1	Membrane receptor
Alpha-1A adrenergic receptor	P35348	ADRA1A	CHEMBL229		0 / 22	Membrane receptor
Alpha-1B adrenergic receptor *(by homology)*	P35368	ADRA1B	CHEMBL232		0 / 14	Membrane receptor
5-hydroxytryptamine receptor 2B *(by homology)*	P41595	HTR2B	CHEMBL1833		0 / 16	Membrane receptor
5-hydroxytryptamine receptor 6	P50406	HTR6	CHEMBL3371		0 / 3	Membrane receptor
5-hydroxytryptamine receptor 1D *(by homology)*	P28221	HTR1D	CHEMBL1983		0 / 1	Membrane receptor
5-hydroxytryptamine receptor 1B *(by homology)*	P28222	HTR1B	CHEMBL1898		0 / 26	Membrane receptor
D(1B) dopamine receptor *(by homology)*	P21918	DRD5	CHEMBL1850		0 / 3	Membrane receptor

Polypharmacology Browser 2 Prediction:

Targets predicted using NN(ECfp4) + NB(ECfp4). Save Table

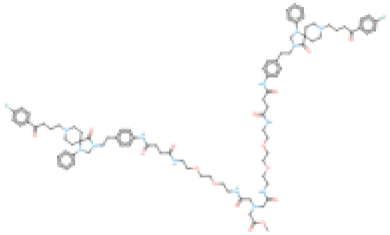

Query molecule

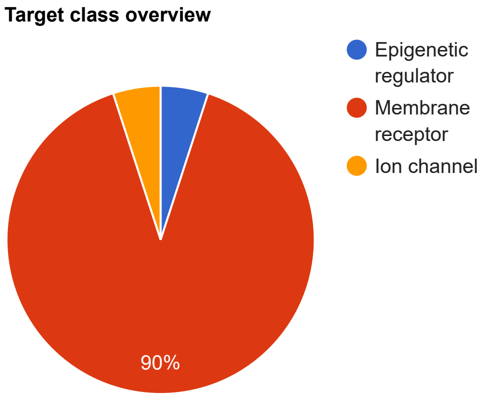

Rank	ChEMBL ID	Common name	Nearest neighbours
1	CHEMBL217 (https://www.ebi.ac.uk/chembl/target/inspect/CHEMBL217)	Dopamine D2 receptor	Show NN
2	CHEMBL339 (https://www.ebi.ac.uk/chembl/target/inspect/CHEMBL339)	Dopamine D2 receptor	Show NN
3	CHEMBL234 (https://www.ebi.ac.uk/chembl/target/inspect/CHEMBL234)	Dopamine D3 receptor	Show NN
4	CHEMBL219 (https://www.ebi.ac.uk/chembl/target/inspect/CHEMBL219)	Dopamine D4 receptor	Show NN
5	CHEMBL273 (https://www.ebi.ac.uk/chembl/target/inspect/CHEMBL273)	Serotonin 1a (5-HT1a) receptor	Show NN
6	CHEMBL233 (https://www.ebi.ac.uk/chembl/target/inspect/CHEMBL233)	Mu opioid receptor	Show NN
7	CHEMBL237 (https://www.ebi.ac.uk/chembl/target/inspect/CHEMBL237)	Kappa opioid receptor	Show NN
8	CHEMBL344 (https://www.ebi.ac.uk/chembl/target/inspect/CHEMBL344)	Melanin-concentrating hormone receptor 1	Show NN
9	CHEMBL214 (https://www.ebi.ac.uk/chembl/target/inspect/CHEMBL214)	Serotonin 1a (5-HT1a) receptor	Show NN
10	CHEMBL240 (https://www.ebi.ac.uk/chembl/target/inspect/CHEMBL240)	HERG	Show NN
11	CHEMBL236 (https://www.ebi.ac.uk/chembl/target/inspect/CHEMBL236)	Delta opioid receptor	Show NN
12	CHEMBL324 (https://www.ebi.ac.uk/chembl/target/inspect/CHEMBL324)	Serotonin 2c (5-HT2c) receptor	Show NN
13	CHEMBL322 (https://www.ebi.ac.uk/chembl/target/inspect/CHEMBL322)	Serotonin 2a (5-HT2a) receptor	Show NN
14	CHEMBL264 (https://www.ebi.ac.uk/chembl/target/inspect/CHEMBL264)	Histamine H3 receptor	Show NN
15	CHEMBL287 (https://www.ebi.ac.uk/chembl/target/inspect/CHEMBL287)	Sigma opioid receptor	Show NN
16	CHEMBL2056 (https://www.ebi.ac.uk/chembl/target/inspect/CHEMBL2056)	Dopamine D1 receptor	Show NN
17	CHEMBL224 (https://www.ebi.ac.uk/chembl/target/inspect/CHEMBL224)	Serotonin 2a (5-HT2a) receptor	Show NN
18	CHEMBL231 (https://www.ebi.ac.uk/chembl/target/inspect/CHEMBL231)	Histamine H1 receptor	Show NN

19	CHEMBL2014 (https://www.ebi.ac.uk/chembl/target/inspect/CHEMBL2014)	Nociceptin receptor	Show NN
20	CHEMBL325 (https://www.ebi.ac.uk/chembl/target/inspect/CHEMBL325)	Histone deacetylase 1	Show NN
