# Supplementary material for: Accelerating Drug Discovery by Early Protein Drug Target Prediction Based on a Multi-Fingerprint Similarity Search
Source: Molecules. 2019 Jun 14;24(12):2233. doi: 10.3390/molecules24122233 (PMC6631269; doi:10.3390/molecules24122233)
Supplement: Supplementary file 1 [file molecules-24-02233-s001.zip › File_S2.zip!/File_S2/Target_Prediction_compound#6.pdf]

MuSSel Prediction k_i:

1 rank

C-C chemokine receptor type 5 : Homo sapiens

score: 8.948 on CHEMBL274 based on 10 fingerprints

Fingerprint type	Ligand	Tanimoto Similarity	activity	Valid fg
FeatMFP1	CHEMBL425618	0.931034	0.24	*
MFP1	CHEMBL425618	0.883721	0.24	*
RDKit7	CHEMBL425618	0.884321	0.24	*
Pattern	CHEMBL425618	0.947099	0.24	*
AP_bits	CHEMBL425618	0.899791	0.24	*
TT_bits	CHEMBL425618	0.851351	0.24	*
FP2	CHEMBL425618	0.718121	0.24	*
hybridization	CHEMBL425618	0.657005	0.24	
substructure	CHEMBL425618	0.954545	0.24	*
graph	CHEMBL425618	0.643617	0.24	
pubchem	CHEMBL425618	0.819277	0.24	
cdk_maccs	CHEMBL425618	0.970149	0.24	*
klekota_roth	CHEMBL425618	0.908163	0.24	*

*** ki ACTIVITY *** value prediction

based on 10 locally validated fgps ---> 30.142

["TT_bits", "substructure", "Pattern", "FP2", "MFP1", "klekota_roth", "AP_bits", "FeatMFP1", "RDKit7", "cdk_maccs"]

MuSSel Prediction IC$_{50}$:

```
1 rank
HERG : Homo sapiens
      score: 9.696 on CHEMBL240 based on 11 fingerprints
Fingerprint type         Ligand  Tanimoto Similarity  activity Valid fg
       FeatMFP1  CHEMBL1201187            0.931034  39810.72        *
           MFP1  CHEMBL1201187            0.883721  39810.72        *
         RDKit7  CHEMBL1201187            0.884321  39810.72        *
        Pattern  CHEMBL3084350            0.951724  10000.00        *
        AP_bits  CHEMBL1201187            0.899791  39810.72        *
        TT_bits  CHEMBL1201187            0.851351  39810.72        *
            FP2  CHEMBL1201187            0.718121  39810.72        *
  hybridization  CHEMBL1201187            0.741546  39810.72        *
   substructure  CHEMBL1834797            0.956522  25000.00        *
          graph  CHEMBL1201187            0.739362  39810.72
        pubchem  CHEMBL1201187            0.825301  39810.72
      cdk_maccs  CHEMBL1201187            0.970149  39810.72        *
   klekota_roth  CHEMBL1201187            0.908163  39810.72        *
      *** ic50 ACTIVITY *** value prediction
      based on 11 locally validated fgps ---> 25074.453
["TT_bits", "substructure", "cdk_maccs", "Pattern", "FP2", "MFP1",
"klekota_roth", "AP_bits", "FeatMFP1", "RDKit7", "hybridization"]

2 rank
C-X-C chemokine receptor type 5 : Homo sapiens
      score: 9.690 on CHEMBL1075315 based on 11 fingerprints
Fingerprint type         Ligand  Tanimoto Similarity  activity Valid fg
       FeatMFP1  CHEMBL1201187            0.931034    0.0024        *
           MFP1  CHEMBL1201187            0.883721    0.0024        *
         RDKit7  CHEMBL1201187            0.884321    0.0024        *
        Pattern  CHEMBL1201187            0.947099    0.0024        *
        AP_bits  CHEMBL1201187            0.899791    0.0024        *
        TT_bits  CHEMBL1201187            0.851351    0.0024        *
            FP2  CHEMBL1201187            0.718121    0.0024        *
  hybridization  CHEMBL1201187            0.741546    0.0024        *
   substructure  CHEMBL1201187            0.954545    0.0024        *
          graph  CHEMBL1201187            0.739362    0.0024
        pubchem  CHEMBL1201187            0.825301    0.0024
      cdk_maccs  CHEMBL1201187            0.970149    0.0024        *
   klekota_roth  CHEMBL1201187            0.908163    0.0024        *
      *** ic50 ACTIVITY *** value prediction
      based on 11 locally validated fgps ---> 0.002
["TT_bits", "substructure", "cdk_maccs", "Pattern", "FP2", "MFP1",
"klekota_roth", "AP_bits", "FeatMFP1", "RDKit7", "hybridization"]

3 rank
Multidrug and toxin extrusion protein 1 : Homo sapiens
      score: 9.690 on CHEMBL1743126 based on 11 fingerprints
Fingerprint type         Ligand  Tanimoto Similarity  activity Valid fg
       FeatMFP1  CHEMBL1201187            0.931034   17300.0        *
           MFP1  CHEMBL1201187            0.883721   17300.0        *
         RDKit7  CHEMBL1201187            0.884321   17300.0        *
        Pattern  CHEMBL1201187            0.947099   17300.0        *
        AP_bits  CHEMBL1201187            0.899791   17300.0        *
        TT_bits  CHEMBL1201187            0.851351   17300.0        *
            FP2  CHEMBL1201187            0.718121   17300.0        *
  hybridization  CHEMBL1201187            0.741546   17300.0        *
   substructure  CHEMBL1201187            0.954545   17300.0        *
          graph  CHEMBL1201187            0.739362   17300.0
        pubchem  CHEMBL1201187            0.825301   17300.0
      cdk_maccs  CHEMBL1201187            0.970149   17300.0        *
```

```
   klekota_roth  CHEMBL1201187             0.908163   17300.0        *
      *** ic50 ACTIVITY *** value prediction
      based on 11 locally validated fgps ---> 17300.000
["TT_bits", "substructure", "cdk_maccs", "Pattern", "FP2", "MFP1",
"klekota_roth", "AP_bits", "FeatMFP1", "RDKit7", "hybridization"]

4 rank
Voltage-gated potassium channel, IKs; KCNQ1(Kv7.1)/KCNE1(MinK) : Homo
sapiens
      score: 9.690 on CHEMBL2221347 based on 11 fingerprints
Fingerprint type         Ligand  Tanimoto Similarity  activity Valid fg
       FeatMFP1  CHEMBL1201187             0.931034  63095.73        *
           MFP1  CHEMBL1201187             0.883721  63095.73        *
         RDKit7  CHEMBL1201187             0.884321  63095.73        *
        Pattern  CHEMBL1201187             0.947099  63095.73        *
        AP_bits  CHEMBL1201187             0.899791  63095.73        *
        TT_bits  CHEMBL1201187             0.851351  63095.73        *
            FP2  CHEMBL1201187             0.718121  63095.73        *
  hybridization  CHEMBL1201187             0.741546  63095.73        *
   substructure  CHEMBL1201187             0.954545  63095.73        *
          graph  CHEMBL1201187             0.739362  63095.73
        pubchem  CHEMBL1201187             0.825301  63095.73
      cdk_maccs  CHEMBL1201187             0.970149  63095.73        *
   klekota_roth  CHEMBL1201187             0.908163  63095.73        *
      *** ic50 ACTIVITY *** value prediction
      based on 11 locally validated fgps ---> 63095.730
["TT_bits", "substructure", "cdk_maccs", "Pattern", "FP2", "MFP1",
"klekota_roth", "AP_bits", "FeatMFP1", "RDKit7", "hybridization"]

5 rank
C-C chemokine receptor type 5 : Homo sapiens
      score: 9.690 on CHEMBL274 based on 11 fingerprints
Fingerprint type         Ligand  Tanimoto Similarity  activity Valid fg
       FeatMFP1  CHEMBL1201187             0.931034       0.2        *
           MFP1  CHEMBL1201187             0.883721       0.2        *
         RDKit7  CHEMBL1201187             0.884321       0.2        *
        Pattern  CHEMBL1201187             0.947099       0.2        *
        AP_bits  CHEMBL1201187             0.899791       0.2        *
        TT_bits  CHEMBL1201187             0.851351       0.2        *
            FP2  CHEMBL1201187             0.718121       0.2        *
  hybridization  CHEMBL1201187             0.741546       0.2        *
   substructure  CHEMBL1201187             0.954545       0.2        *
          graph  CHEMBL1201187             0.739362       0.2
        pubchem  CHEMBL1201187             0.825301       0.2
      cdk_maccs  CHEMBL1201187             0.970149       0.2        *
   klekota_roth  CHEMBL1201187             0.908163       0.2        *
      *** ic50 ACTIVITY *** value prediction
      based on 11 locally validated fgps ---> 1.272
["TT_bits", "substructure", "cdk_maccs", "Pattern", "FP2", "MFP1",
"klekota_roth", "AP_bits", "FeatMFP1", "RDKit7", "hybridization"]
```

Polypharmacology Browser 2 Prediction:

Targets predicted using NN(ECfp4) + NB(ECfp4). Save Table

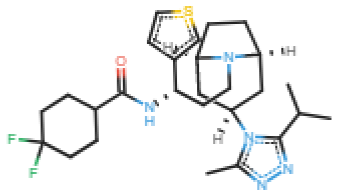

Query molecule

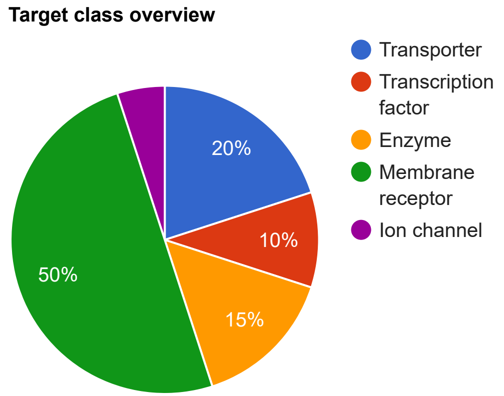

Rank	ChEMBL ID	Common name	Nearest neighbours
1	CHEMBL274 (https://www.ebi.ac.uk/chembl/target/inspect/CHEMBL274)	C-C chemokine receptor type 5	Show NN
2	CHEMBL240 (https://www.ebi.ac.uk/chembl/target/inspect/CHEMBL240)	HERG	Show NN
3	CHEMBL217 (https://www.ebi.ac.uk/chembl/target/inspect/CHEMBL217)	Dopamine D2 receptor	Show NN
4	CHEMBL268 (https://www.ebi.ac.uk/chembl/target/inspect/CHEMBL268)	Cathepsin K	Show NN
5	CHEMBL233 (https://www.ebi.ac.uk/chembl/target/inspect/CHEMBL233)	Mu opioid receptor	Show NN
6	CHEMBL2954 (https://www.ebi.ac.uk/chembl/target/inspect/CHEMBL2954)	Cathepsin S	Show NN
7	CHEMBL216 (https://www.ebi.ac.uk/chembl/target/inspect/CHEMBL216)	Muscarinic acetylcholine receptor M1	Show NN
8	CHEMBL237 (https://www.ebi.ac.uk/chembl/target/inspect/CHEMBL237)	Kappa opioid receptor	Show NN
9	CHEMBL338 (https://www.ebi.ac.uk/chembl/target/inspect/CHEMBL338)	Dopamine transporter	Show NN
10	CHEMBL264 (https://www.ebi.ac.uk/chembl/target/inspect/CHEMBL264)	Histamine H3 receptor	Show NN
11	CHEMBL313 (https://www.ebi.ac.uk/chembl/target/inspect/CHEMBL313)	Serotonin transporter	Show NN
12	CHEMBL344 (https://www.ebi.ac.uk/chembl/target/inspect/CHEMBL344)	Melanin-concentrating hormone receptor 1	Show NN
13	CHEMBL3227 (https://www.ebi.ac.uk/chembl/target/inspect/CHEMBL3227)	Metabotropic glutamate receptor 5	Show NN
14	CHEMBL4015 (https://www.ebi.ac.uk/chembl/target/inspect/CHEMBL4015)	C-C chemokine receptor type 2	Show NN
15	CHEMBL3837 (https://www.ebi.ac.uk/chembl/target/inspect/CHEMBL3837)	Cathepsin L	Show NN
16	CHEMBL304 (https://www.ebi.ac.uk/chembl/target/inspect/CHEMBL304)	Norepinephrine transporter	Show NN
17	CHEMBL2034 (https://www.ebi.ac.uk/chembl/target/inspect/CHEMBL2034)	Glucocorticoid receptor	Show NN
18	CHEMBL276 (https://www.ebi.ac.uk/chembl/target/inspect/CHEMBL276)	Muscarinic acetylcholine receptor M1	Show NN

19	CHEMBL222 (https://www.ebi.ac.uk/chembl/target/inspect/CHEMBL222)	Norepinephrine transporter	Show NN
20	CHEMBL235 (https://www.ebi.ac.uk/chembl/target/inspect/CHEMBL235)	Peroxisome proliferator-activated receptor gamma	Show NN

SwissTargetPrediction report:

Reference:
Gfeller D., Michielin O. & Zoete V.
Shaping the interaction landscape of bioactive molecules, *Bioinformatics* (2013) 29:3073-3079.

Query Molecule

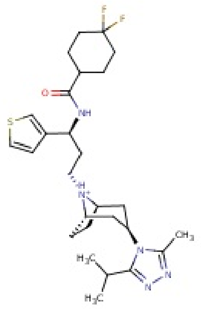

Frequency of Target Class

Target	Uniprot ID	Gene code	ChEMBL ID	Probability	# sim. cmpds (3D / 2D)	Target Class
C-C chemokine receptor type 1 *(by homology)*	P32246	CCR1	CHEMBL2413		958 / 54	Membrane receptor
C-C chemokine receptor type 2 *(by homology)*	P41597	CCR2	CHEMBL4015		977 / 54	Membrane receptor
C-C chemokine receptor type 3	P51677	CCR3	CHEMBL3473		958 / 54	Membrane receptor
C-C chemokine receptor type 5	P51681	CCR5	CHEMBL274		977 / 54	Membrane receptor
C-C chemokine receptor-like 2 *(by homology)*	C9JP23	CCRL2			875 / 54	Membrane receptor
Potassium voltage-gated channel subfamily H member 2	Q12809	KCNH2	CHEMBL240		526 / 5	Ion channel
Potassium voltage-gated channel subfamily H member 6 *(by homology)*	Q9H252	KCNH6			526 / 5	Ion channel
Potassium voltage-gated channel subfamily H member 7 *(by homology)*	Q9NS40	KCNH7			526 / 5	Ion channel
Sodium- and chloride-dependent glycine transporter 1	P48067	SLC6A9	CHEMBL2337		435 / 3	Transporter
Sodium- and chloride-dependent glycine transporter 2 *(by homology)*	Q9Y345	SLC6A5	CHEMBL3060		435 / 3	Transporter
Sodium-dependent proline transporter *(by homology)*	Q99884	SLC6A7			435 / 3	Transporter
Sodium- and chloride-dependent neutral and basic amino acid transporter B(0+) *(by homology)*	Q9UN76	SLC6A14			435 / 3	Transporter
Muscarinic acetylcholine receptor M2	P08172	CHRM2	CHEMBL211		201 / 3	Membrane receptor
Muscarinic acetylcholine receptor M4 *(by homology)*	P08173	CHRM4	CHEMBL1821		246 / 3	Membrane receptor
Muscarinic acetylcholine receptor M5 *(by homology)*	P08912	CHRM5	CHEMBL2035		204 / 3	Membrane receptor
