# Supplementary material for: Accelerating Drug Discovery by Early Protein Drug Target Prediction Based on a Multi-Fingerprint Similarity Search
Source: Molecules. 2019 Jun 14;24(12):2233. doi: 10.3390/molecules24122233 (PMC6631269; doi:10.3390/molecules24122233)
Supplement: Supplementary file 1 [file molecules-24-02233-s001.zip › File_S2.zip!/File_S2/Target_Prediction_compound#7.pdf]

MuSSel Prediction k_i:

1 rank

Canalicular multispecific organic anion transporter 1 : Rattus norvegicus

score: 4.145 on CHEMBL2073676 based on 5 fingerprints

Fingerprint type	Ligand	Tanimoto Similarity	activity	Valid fg
FeatMFP1	CHEMBL1743143	0.619048	1620.0	
MFP1	CHEMBL1743143	0.507463	1620.0	
RDKit7	CHEMBL1743143	0.899092	1620.0	*
Pattern	CHEMBL1743143	0.925813	1620.0	*
AP_bits	CHEMBL1743143	0.518750	1620.0	
TT_bits	CHEMBL2074578	0.423729	18300.0	
FP2	CHEMBL1743143	0.709779	1620.0	*
hybridization	CHEMBL1743143	0.765333	1620.0	*
substructure	CHEMBL2074700	0.666667	56900.0	
graph	CHEMBL2074712	0.609442	96600.0	
pubchem	CHEMBL2074712	0.824786	96600.0	
cdk_maccs	CHEMBL2074712	0.845238	96600.0	*
klekota_roth	CHEMBL1743143	0.536313	1620.0	

*** ki ACTIVITY *** value prediction

based on 5 locally validated fgps ---> 11046.981

["Pattern", "RDKit7", "hybridization", "cdk_maccs", "FP2"]

MuSSel Prediction IC_{50}:

```
1 rank
DNA topoisomerase I : Homo sapiens
     score: 9.090 on CHEMBL1781 based on 12 fingerprints
Fingerprint type        Ligand  Tanimoto Similarity  activity Valid fg
       FeatMFP1   CHEMBL72627             0.684211    1118.0        *
           MFP1  CHEMBL545250             0.586207     430.0        *
         RDKit7  CHEMBL545250             0.920808     430.0        *
        Pattern  CHEMBL159322             0.896690     340.0        *
        AP_bits  CHEMBL312690             0.574635    4597.0        *
        TT_bits  CHEMBL539032             0.638095    5500.0        *
            FP2  CHEMBL543849             0.791667    1100.0        *
  hybridization  CHEMBL545250             0.843165     430.0        *
   substructure  CHEMBL170532             0.724138    2100.0
          graph  CHEMBL539032             0.775862    5500.0        *
        pubchem   CHEMBL88605             0.840517   21000.0        *
      cdk_maccs  CHEMBL275538             0.878049    1494.0        *
   klekota_roth  CHEMBL539032             0.660256    5500.0        *
     *** ic50 ACTIVITY *** value prediction
     based on 12 locally validated fgps ---> 5875.206
["TT_bits", "cdk_maccs", "Pattern", "FP2", "MFP1", "klekota_roth",
"pubchem", "graph", "AP_bits", "FeatMFP1", "RDKit7", "hybridization"]

2 rank
DNA topoisomerase I : Mus musculus
     score: 7.201 on CHEMBL2814 based on 10 fingerprints
Fingerprint type        Ligand  Tanimoto Similarity  activity Valid fg
       FeatMFP1  CHEMBL445883             0.564103   12000.0
           MFP1   CHEMBL44044             0.533333   41000.0        *
         RDKit7      CHEMBL84             0.911797   20000.0        *
        Pattern  CHEMBL289665             0.852329    5000.0        *
        AP_bits  CHEMBL289665             0.478326    5000.0        *
        TT_bits   CHEMBL44044             0.539823   41000.0        *
            FP2      CHEMBL84             0.784722   20000.0        *
  hybridization     CHEMBL837             0.775510    4000.0        *
   substructure      CHEMBL65             0.586207   12000.0
          graph  CHEMBL289665             0.720339    5000.0
        pubchem  CHEMBL289665             0.842553    5000.0        *
      cdk_maccs  CHEMBL289665             0.886076    5000.0        *
   klekota_roth   CHEMBL44044             0.596273   41000.0        *
     *** ic50 ACTIVITY *** value prediction
     based on 10 locally validated fgps ---> 22518.935
["TT_bits", "cdk_maccs", "Pattern", "AP_bits", "MFP1", "klekota_roth",
"pubchem", "FP2", "RDKit7", "hybridization"]

3 rank
Nuclear receptor coactivator 3 : Homo sapiens
     score: 7.184 on CHEMBL1615382 based on 10 fingerprints
Fingerprint type         Ligand  Tanimoto Similarity  activity Valid fg
       FeatMFP1  CHEMBL1566908             0.675676    3860.0
           MFP1  CHEMBL1445386             0.532258    1049.0        *
         RDKit7  CHEMBL1200512             0.897051    1315.0        *
        Pattern  CHEMBL1200512             0.901508    1315.0        *
        AP_bits  CHEMBL1200512             0.520408    1315.0        *
        TT_bits  CHEMBL1445386             0.517857    1049.0        *
            FP2  CHEMBL1200512             0.762215    1315.0        *
  hybridization  CHEMBL1200512             0.760274    1315.0        *
   substructure  CHEMBL1566908             0.714286    3860.0
          graph  CHEMBL1200512             0.742489    1315.0
        pubchem  CHEMBL1200512             0.841202    1315.0        *
      cdk_maccs  CHEMBL1200512             0.857143    1315.0        *
```

```
   klekota_roth  CHEMBL1445386             0.593750    1049.0        *
      *** ic50 ACTIVITY *** value prediction
      based on 10 locally validated fgps ---> 1183.069
["TT_bits", "cdk_maccs", "Pattern", "AP_bits", "MFP1", "klekota_roth",
"pubchem", "FP2", "RDKit7", "hybridization"]

4 rank
Cytochrome P450 3A4 : Homo sapiens
      score: 6.051 on CHEMBL340 based on 8 fingerprints
Fingerprint type         Ligand  Tanimoto Similarity  activity Valid fg
       FeatMFP1  CHEMBL2048199             0.575000    4000.0
           MFP1       CHEMBL84             0.500000    1521.1
         RDKit7       CHEMBL84             0.911797    1521.1        *
        Pattern       CHEMBL84             0.847893    1521.1        *
        AP_bits  CHEMBL3634394             0.528283   10000.0        *
        TT_bits       CHEMBL84             0.517857    1521.1        *
            FP2       CHEMBL84             0.784722    1521.1        *
  hybridization       CHEMBL84             0.774052    1521.1        *
   substructure  CHEMBL1778481             0.785714    8900.0
          graph       CHEMBL84             0.673913    1521.1
        pubchem       CHEMBL84             0.834061    1521.1        *
      cdk_maccs       CHEMBL84             0.851852    1521.1        *
   klekota_roth       CHEMBL84             0.586420    1521.1
      *** ic50 ACTIVITY *** value prediction
      based on 8 locally validated fgps ---> 8007.669
["TT_bits", "cdk_maccs", "Pattern", "AP_bits", "pubchem", "FP2",
"RDKit7", "hybridization"]

5 rank
ATP-binding cassette sub-family G member 2 : Homo sapiens
      score: 6.008 on CHEMBL5393 based on 8 fingerprints
Fingerprint type         Ligand  Tanimoto Similarity  activity Valid fg
       FeatMFP1      CHEMBL837             0.500000     176.0
           MFP1       CHEMBL84             0.500000     170.0
         RDKit7       CHEMBL84             0.911797     170.0        *
        Pattern       CHEMBL84             0.847893     170.0        *
        AP_bits  CHEMBL2013174             0.484689    1400.0        *
        TT_bits       CHEMBL84             0.517857     170.0        *
            FP2       CHEMBL84             0.784722     170.0        *
  hybridization      CHEMBL837             0.775510     176.0        *
   substructure   CHEMBL551696             0.633333    9100.0
          graph       CHEMBL84             0.673913     170.0
        pubchem       CHEMBL84             0.834061     170.0        *
      cdk_maccs       CHEMBL84             0.851852     170.0        *
   klekota_roth       CHEMBL84             0.586420     170.0
      *** ic50 ACTIVITY *** value prediction
      based on 8 locally validated fgps ---> 272.749
["TT_bits", "cdk_maccs", "Pattern", "AP_bits", "pubchem", "FP2",
"RDKit7", "hybridization"]

6 rank
Alpha trans-inducing protein (VP16) : Herpes simplex virus (type 1 /
strain 17)
      score: 5.540 on CHEMBL4218 based on 7 fingerprints
Fingerprint type         Ligand  Tanimoto Similarity  activity Valid fg
       FeatMFP1  CHEMBL1566908             0.675676   18440.0
           MFP1  CHEMBL1200512             0.470588   41996.0
         RDKit7  CHEMBL1200512             0.897051   41996.0        *
        Pattern  CHEMBL1200512             0.901508   41996.0        *
        AP_bits  CHEMBL1200512             0.520408   41996.0        *
```

```
        TT_bits  CHEMBL1200512            0.404255   41996.0
            FP2  CHEMBL1200512            0.762215   41996.0        *
  hybridization  CHEMBL1200512            0.760274   41996.0        *
   substructure  CHEMBL1566908            0.714286   18440.0
          graph  CHEMBL1200512            0.742489   41996.0
        pubchem  CHEMBL1200512            0.841202   41996.0        *
      cdk_maccs  CHEMBL1200512            0.857143   41996.0        *
   klekota_roth  CHEMBL1200512            0.540541   41996.0
      *** ic50 ACTIVITY *** value prediction
      based on 7 locally validated fgps ---> 37322.814
["cdk_maccs", "Pattern", "AP_bits", "pubchem", "FP2", "RDKit7",
"hybridization"]

7 rank
Solute carrier family 22 member 2 : Homo sapiens
      score: 5.522 on CHEMBL1743122 based on 7 fingerprints
Fingerprint type     Ligand  Tanimoto Similarity  activity Valid fg
       FeatMFP1   CHEMBL84              0.465116   61000.0
           MFP1   CHEMBL84              0.500000   61000.0
         RDKit7   CHEMBL84              0.911797   61000.0        *
        Pattern   CHEMBL84              0.847893   61000.0        *
        AP_bits  CHEMBL603              0.467254    9700.0
        TT_bits   CHEMBL84              0.517857   61000.0        *
            FP2   CHEMBL84              0.784722   61000.0        *
  hybridization   CHEMBL84              0.774052   61000.0        *
   substructure   CHEMBL85              0.545455   11000.0
          graph   CHEMBL84              0.673913   61000.0
        pubchem   CHEMBL84              0.834061   61000.0        *
      cdk_maccs   CHEMBL84              0.851852   61000.0        *
   klekota_roth   CHEMBL84              0.586420   61000.0
      *** ic50 ACTIVITY *** value prediction
      based on 7 locally validated fgps ---> 60280.389
["TT_bits", "cdk_maccs", "Pattern", "pubchem", "FP2", "RDKit7",
"hybridization"]

8 rank
Multidrug and toxin extrusion protein 1 : Homo sapiens
      score: 5.522 on CHEMBL1743126 based on 7 fingerprints
Fingerprint type      Ligand  Tanimoto Similarity  activity Valid fg
       FeatMFP1    CHEMBL84              0.465116    1300.0
           MFP1    CHEMBL84              0.500000    1300.0
         RDKit7    CHEMBL84              0.911797    1300.0        *
        Pattern    CHEMBL84              0.847893    1300.0        *
        AP_bits   CHEMBL603              0.467254    1300.0
        TT_bits    CHEMBL84              0.517857    1300.0        *
            FP2    CHEMBL84              0.784722    1300.0        *
  hybridization    CHEMBL84              0.774052    1300.0        *
   substructure  CHEMBL1017              0.642857   17900.0
          graph    CHEMBL84              0.673913    1300.0
        pubchem    CHEMBL84              0.834061    1300.0        *
      cdk_maccs    CHEMBL84              0.851852    1300.0        *
   klekota_roth    CHEMBL84              0.586420    1300.0
      *** ic50 ACTIVITY *** value prediction
      based on 7 locally validated fgps ---> 1397.245
["TT_bits", "cdk_maccs", "Pattern", "pubchem", "FP2", "RDKit7",
"hybridization"]

9 rank
Multidrug and toxin extrusion protein 2 : Homo sapiens
      score: 5.522 on CHEMBL1743127 based on 7 fingerprints
```

Fingerprint type	Ligand	Tanimoto Similarity	activity	Valid fg
FeatMFP1	CHEMBL84	0.465116	8600.0	
MFP1	CHEMBL84	0.500000	8600.0	
RDKit7	CHEMBL84	0.911797	8600.0	*
Pattern	CHEMBL84	0.847893	8600.0	*
AP_bits	CHEMBL603	0.467254	7600.0	
TT_bits	CHEMBL84	0.517857	8600.0	*
FP2	CHEMBL84	0.784722	8600.0	*
hybridization	CHEMBL84	0.774052	8600.0	*
substructure	CHEMBL46	0.533333	160.0	
graph	CHEMBL84	0.673913	8600.0	
pubchem	CHEMBL84	0.834061	8600.0	*
cdk_maccs	CHEMBL84	0.851852	8600.0	*
klekota_roth	CHEMBL84	0.586420	8600.0	

*** ic50 ACTIVITY *** value prediction
based on 7 locally validated fgps ---> 8859.319
["TT_bits", "cdk_maccs", "Pattern", "pubchem", "FP2", "RDKit7", "hybridization"]

10 rank
Hypoxia-inducible factor 1 alpha : Homo sapiens
score: 5.522 on CHEMBL4261 based on 7 fingerprints

Fingerprint type	Ligand	Tanimoto Similarity	activity	Valid fg
FeatMFP1	CHEMBL84	0.465116	60.0	
MFP1	CHEMBL84	0.500000	60.0	
RDKit7	CHEMBL84	0.911797	60.0	*
Pattern	CHEMBL84	0.847893	60.0	*
AP_bits	CHEMBL383824	0.455711	57.0	
TT_bits	CHEMBL84	0.517857	60.0	*
FP2	CHEMBL84	0.784722	60.0	*
hybridization	CHEMBL84	0.774052	60.0	*
substructure	CHEMBL430393	0.655172	800.0	
graph	CHEMBL84	0.673913	60.0	
pubchem	CHEMBL84	0.834061	60.0	*
cdk_maccs	CHEMBL84	0.851852	60.0	*
klekota_roth	CHEMBL84	0.586420	60.0	

*** ic50 ACTIVITY *** value prediction
based on 7 locally validated fgps ---> 105.797
["TT_bits", "cdk_maccs", "Pattern", "pubchem", "FP2", "RDKit7", "hybridization"]

SwissTargetPrediction report:

Reference:
Gfeller D., Michielin O. & Zoete V.
Shaping the interaction landscape of bioactive molecules, *Bioinformatics* (2013) 29:3073-3079.

Query Molecule

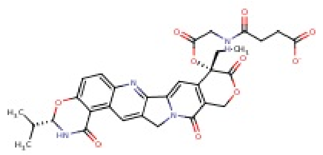

Frequency of Target Class

Target	Uniprot ID	Gene code	ChEMBL ID	Probability	# sim. cmpds (3D / 2D)	Target Class
22 kDa interstitial collagenase	P03956	MMP1	CHEMBL332		34 / 1	Metallo Protease
Stromelysin-1 *(by homology)*	P08254	MMP3	CHEMBL283		34 / 1	Metallo Protease
Collagenase 3	P45452	MMP13	CHEMBL280		34 / 1	Metallo Protease
Stromelysin-2 *(by homology)*	P09238	MMP10	CHEMBL4270		34 / 1	Metallo Protease
Macrophage metalloelastase *(by homology)*	P39900	MMP12	CHEMBL4393		34 / 1	Metallo Protease
Matrix metalloproteinase-27 *(by homology)*	Q9H306	MMP27			34 / 1	Metallo Protease
Neutrophil collagenase	P22894	MMP8	CHEMBL4588		5 / 1	Metallo Protease
Complex	P06756/P05106	ITGAV/ITGB3	CHEMBL1907598		44 / 3	Membrane receptor
Complex	P06756/P18084	ITGAV/ITGB5	CHEMBL2096675		6 / 3	Membrane receptor
Muscleblind-like protein 1	Q9NR56	MBNL1	CHEMBL1293317		5 / 3	Unclassified
Muscleblind-like protein 2 *(by homology)*	Q5VZF2	MBNL2			5 / 3	Unclassified
Muscleblind-like protein 3 *(by homology)*	Q9NUK0	MBNL3			5 / 3	Unclassified
DNA topoisomerase 1	P11387	TOP1	CHEMBL1781		0 / 160	Enzyme
DNA topoisomerase I, mitochondrial *(by homology)*	Q969P6	TOP1MT			0 / 160	Enzyme
Cytochrome P450 3A4	P08684	CYP3A4	CHEMBL340		0 / 1	Enzyme

Polypharmacology Browser 2 Prediction:

Targets predicted using NN(ECfp4) + NB(ECfp4). Save Table

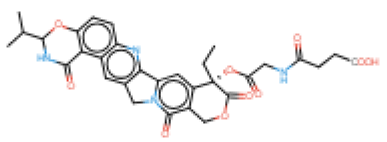

Query molecule

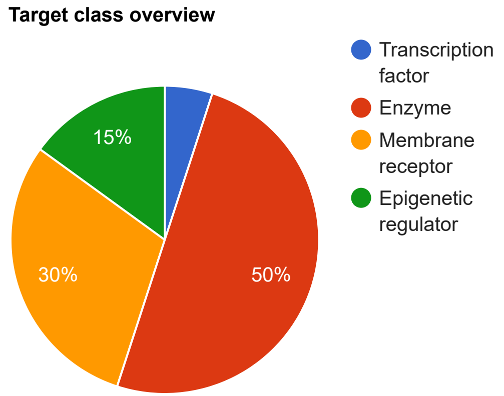

Rank	ChEMBL ID	Common name	Nearest neighbours
1	CHEMBL1781 (https://www.ebi.ac.uk/chembl/target/inspect/CHEMBL1781)	DNA topoisomerase I	Show NN
2	CHEMBL325 (https://www.ebi.ac.uk/chembl/target/inspect/CHEMBL325)	Histone deacetylase 1	Show NN
3	CHEMBL1865 (https://www.ebi.ac.uk/chembl/target/inspect/CHEMBL1865)	Histone deacetylase 6	Show NN
4	CHEMBL3192 (https://www.ebi.ac.uk/chembl/target/inspect/CHEMBL3192)	Histone deacetylase 8	Show NN
5	CHEMBL1945 (https://www.ebi.ac.uk/chembl/target/inspect/CHEMBL1945)	Melatonin receptor 1A	Show NN
6	CHEMBL2334 (https://www.ebi.ac.uk/chembl/target/inspect/CHEMBL2334)	Caspase-3	Show NN
7	CHEMBL1946 (https://www.ebi.ac.uk/chembl/target/inspect/CHEMBL1946)	Melatonin receptor 1B	Show NN
8	CHEMBL1835 (https://www.ebi.ac.uk/chembl/target/inspect/CHEMBL1835)	Thromboxane-A synthase	Show NN
9	CHEMBL217 (https://www.ebi.ac.uk/chembl/target/inspect/CHEMBL217)	Dopamine D2 receptor	Show NN
10	CHEMBL2069 (https://www.ebi.ac.uk/chembl/target/inspect/CHEMBL2069)	Thromboxane A2 receptor	Show NN
11	CHEMBL4829 (https://www.ebi.ac.uk/chembl/target/inspect/CHEMBL4829)	Acetyl-CoA carboxylase 2	Show NN
12	CHEMBL3892 (https://www.ebi.ac.uk/chembl/target/inspect/CHEMBL3892)	Sphingosine 1-phosphate receptor Edg-3	Show NN
13	CHEMBL220 (https://www.ebi.ac.uk/chembl/target/inspect/CHEMBL220)	Acetylcholinesterase	Show NN
14	CHEMBL204 (https://www.ebi.ac.uk/chembl/target/inspect/CHEMBL204)	Thrombin	Show NN
15	CHEMBL3105 (https://www.ebi.ac.uk/chembl/target/inspect/CHEMBL3105)	Poly [ADP-ribose] polymerase-1	Show NN
16	CHEMBL244 (https://www.ebi.ac.uk/chembl/target/inspect/CHEMBL244)	Coagulation factor X	Show NN
17	CHEMBL259 (https://www.ebi.ac.uk/chembl/target/inspect/CHEMBL259)	Melanocortin receptor 4	Show NN
18	CHEMBL4801 (https://www.ebi.ac.uk/chembl/target/inspect/CHEMBL4801)	Caspase-1	Show NN

19	CHEMBL248 (https://www.ebi.ac.uk/chembl/target/inspect/CHEMBL248)	Leukocyte elastase	Show NN
20	CHEMBL235 (https://www.ebi.ac.uk/chembl/target/inspect/CHEMBL235)	Peroxisome proliferator-activated receptor gamma	Show NN
