# Supplementary material for: Accelerating Drug Discovery by Early Protein Drug Target Prediction Based on a Multi-Fingerprint Similarity Search
Source: Molecules. 2019 Jun 14;24(12):2233. doi: 10.3390/molecules24122233 (PMC6631269; doi:10.3390/molecules24122233)
Supplement: Supplementary file 1 [file molecules-24-02233-s001.zip › File_S2.zip!/File_S2/Target_Prediction_compound#8.pdf]

MuSSel Prediction IC_{50}:

```
1 rank
Tyrosine-protein kinase BTK : Homo sapiens
     score: 8.563 on CHEMBL5251 based on 11 fingerprints
Fingerprint type         Ligand  Tanimoto Similarity  activity Valid fg
       FeatMFP1  CHEMBL3400977            0.675676     65.00
           MFP1  CHEMBL3674679            0.660000     16.00        *
         RDKit7  CHEMBL3400973            0.743785   1568.00        *
        Pattern  CHEMBL3402578            0.848787   1357.00        *
        AP_bits  CHEMBL3402576            0.732558     21.30        *
        TT_bits  CHEMBL3400973            0.619048   1568.00        *
            FP2  CHEMBL3400973            0.670157   1568.00        *
  hybridization  CHEMBL3402575            0.651261     38.80        *
   substructure  CHEMBL3402576            0.916667     21.30        *
          graph  CHEMBL3400973            0.754967   1568.00
        pubchem  CHEMBL3402576            0.913793     21.30        *
      cdk_maccs  CHEMBL3402576            0.938462     21.30        *
   klekota_roth  CHEMBL3649459            0.868852     39.35        *
     *** ic50 ACTIVITY *** value prediction
     based on 11 locally validated fgps ---> 308.391
["TT_bits", "substructure", "cdk_maccs", "Pattern", "AP_bits", "MFP1",
"klekota_roth", "pubchem", "FP2", "RDKit7", "hybridization"]
```

SwissTargetPrediction report:

Reference:
Gfeller D., Michielin O. & Zoete V.
Shaping the interaction landscape of bioactive molecules, *Bioinformatics* (2013) 29:3073-3079.

Query Molecule

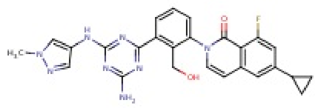

Frequency of Target Class

Target	Uniprot ID	Gene code	ChEMBL ID	Probability	# sim. cmpds (3D / 2D)	Target Class
Tyrosine-protein kinase TXK	P42681	TXK	CHEMBL4367		2 / 1	Tyr Kinase
Tyrosine-protein kinase BTK	Q06187	BTK	CHEMBL5251		2 / 1	Tyr Kinase
Tyrosine-protein kinase ITK/TSK	Q08881	ITK	CHEMBL2959		2 / 1	Tyr Kinase
Tyrosine-protein kinase Tec *(by homology)*	P42680	TEC	CHEMBL4246		2 / 1	Tyr Kinase
5-hydroxytryptamine receptor 1A	P08908	HTR1A	CHEMBL214		5 / 1	Membrane receptor
5-hydroxytryptamine receptor 1B	P28222	HTR1B	CHEMBL1898		5 / 1	Membrane receptor
Microtubule-associated protein tau	P10636	MAPT	CHEMBL1293224		18 / 1	Unclassified
Nitric oxide synthase, endothelial	P29474	NOS3	CHEMBL4803		1 / 1	Enzyme
Nitric oxide synthase, brain	P29475	NOS1	CHEMBL3568		1 / 1	Enzyme
Nitric oxide synthase, inducible	P35228	NOS2	CHEMBL4481		1 / 1	Enzyme
Muscarinic acetylcholine receptor M1	P11229	CHRM1	CHEMBL216		2 / 5	Membrane receptor
Muscarinic acetylcholine receptor M2 *(by homology)*	P08172	CHRM2	CHEMBL211		2 / 5	Membrane receptor
Muscarinic acetylcholine receptor M4 *(by homology)*	P08173	CHRM4	CHEMBL1821		2 / 5	Membrane receptor
Muscarinic acetylcholine receptor M3 *(by homology)*	P20309	CHRM3	CHEMBL245		1 / 5	Membrane receptor
D(2) dopamine receptor	P14416	DRD2	CHEMBL217		1 / 1	Membrane receptor

Polypharmacology Browser 2 Prediction:

Targets predicted using NN(ECfp4) + NB(ECfp4). Save Table

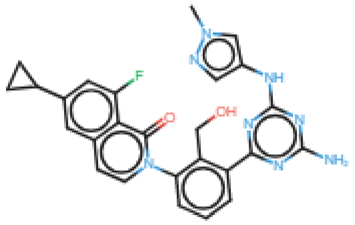

Query molecule

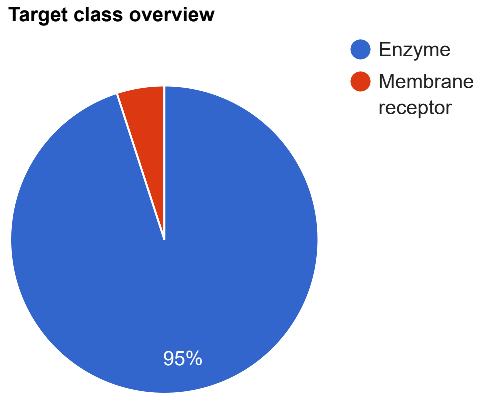

Rank	ChEMBL ID	Common name	Nearest neighbours
1	CHEMBL5251 (https://www.ebi.ac.uk/chembl/target/inspect/CHEMBL5251)	Tyrosine-protein kinase BTK	Show NN
2	CHEMBL279 (https://www.ebi.ac.uk/chembl/target/inspect/CHEMBL279)	Vascular endothelial growth factor receptor 2	Show NN
3	CHEMBL2971 (https://www.ebi.ac.uk/chembl/target/inspect/CHEMBL2971)	Tyrosine-protein kinase JAK2	Show NN
4	CHEMBL2842 (https://www.ebi.ac.uk/chembl/target/inspect/CHEMBL2842)	Serine/threonine-protein kinase mTOR	Show NN
5	CHEMBL2599 (https://www.ebi.ac.uk/chembl/target/inspect/CHEMBL2599)	Tyrosine-protein kinase SYK	Show NN
6	CHEMBL2276 (https://www.ebi.ac.uk/chembl/target/inspect/CHEMBL2276)	c-Jun N-terminal kinase 1	Show NN
7	CHEMBL4722 (https://www.ebi.ac.uk/chembl/target/inspect/CHEMBL4722)	Serine/threonine-protein kinase Aurora-A	Show NN
8	CHEMBL251 (https://www.ebi.ac.uk/chembl/target/inspect/CHEMBL251)	Adenosine A2a receptor	Show NN
9	CHEMBL1974 (https://www.ebi.ac.uk/chembl/target/inspect/CHEMBL1974)	Tyrosine-protein kinase receptor FLT3	Show NN
10	CHEMBL1936 (https://www.ebi.ac.uk/chembl/target/inspect/CHEMBL1936)	Stem cell growth factor receptor	Show NN
11	CHEMBL258 (https://www.ebi.ac.uk/chembl/target/inspect/CHEMBL258)	Tyrosine-protein kinase LCK	Show NN
12	CHEMBL4247 (https://www.ebi.ac.uk/chembl/target/inspect/CHEMBL4247)	ALK tyrosine kinase receptor	Show NN
13	CHEMBL2815 (https://www.ebi.ac.uk/chembl/target/inspect/CHEMBL2815)	Nerve growth factor receptor Trk-A	Show NN
14	CHEMBL2185 (https://www.ebi.ac.uk/chembl/target/inspect/CHEMBL2185)	Serine/threonine-protein kinase Aurora-B	Show NN
15	CHEMBL3717 (https://www.ebi.ac.uk/chembl/target/inspect/CHEMBL3717)	Hepatocyte growth factor receptor	Show NN
16	CHEMBL2534 (https://www.ebi.ac.uk/chembl/target/inspect/CHEMBL2534)	3-phosphoinositide dependent protein kinase-1	Show NN
17	CHEMBL4179 (https://www.ebi.ac.uk/chembl/target/inspect/CHEMBL4179)	c-Jun N-terminal kinase 2	Show NN
18	CHEMBL2147 (https://www.ebi.ac.uk/chembl/target/inspect/CHEMBL2147)	Serine/threonine-protein kinase PIM1	Show NN

19	CHEMBL2148 (https://www.ebi.ac.uk/chembl/target/inspect/CHEMBL2148)	Tyrosine-protein kinase JAK3	Show NN
20	CHEMBL301 (https://www.ebi.ac.uk/chembl/target/inspect/CHEMBL301)	Cyclin-dependent kinase 2	Show NN
