# Supplementary material for: Accelerating Drug Discovery by Early Protein Drug Target Prediction Based on a Multi-Fingerprint Similarity Search
Source: Molecules. 2019 Jun 14;24(12):2233. doi: 10.3390/molecules24122233 (PMC6631269; doi:10.3390/molecules24122233)
Supplement: Supplementary file 1 [file molecules-24-02233-s001.zip › File_S2.zip!/File_S2/Target_Prediction_compound#9.pdf]

MuSSel Prediction k_i:

```
1 rank
Cytochrome P450 19A1 : Homo sapiens
      score: 10.442 on CHEMBL1978 based on 12 fingerprints
Fingerprint type         Ligand  Tanimoto Similarity  activity Valid fg
       FeatMFP1  CHEMBL1627334            0.947368     100.0        *
           MFP1  CHEMBL1627334            0.827586     100.0        *
         RDKit7  CHEMBL3753034            0.712714     100.0
        Pattern  CHEMBL3752556            0.945518     100.0        *
        AP_bits  CHEMBL3752556            0.637540     100.0        *
        TT_bits   CHEMBL241993            0.769231     100.0        *
            FP2   CHEMBL241993            0.727273     100.0        *
  hybridization  CHEMBL3753034            0.881579     100.0        *
   substructure  CHEMBL1627334            0.928571     100.0        *
          graph  CHEMBL1627334            0.977273     100.0        *
        pubchem  CHEMBL3752422            0.940678     100.0        *
      cdk_maccs  CHEMBL1627334            0.947368     100.0        *
   klekota_roth  CHEMBL1627334            0.911765     100.0        *
      *** ki ACTIVITY *** value prediction
      based on 12 locally validated fgps ---> 97.735
["TT_bits", "substructure", "Pattern", "AP_bits", "MFP1", "klekota_roth",
"pubchem", "graph", "FP2", "FeatMFP1", "cdk_maccs", "hybridization"]

2 rank
Steryl-sulfatase : Homo sapiens
      score: 8.235 on CHEMBL3559 based on 10 fingerprints
Fingerprint type         Ligand  Tanimoto Similarity  activity Valid fg
       FeatMFP1  CHEMBL1835544            0.850000      30.0        *
           MFP1  CHEMBL1835544            0.540541      30.0
         RDKit7   CHEMBL482212            0.683849     250.0
        Pattern   CHEMBL482212            0.945111     250.0        *
        AP_bits  CHEMBL1835544            0.554252      30.0        *
        TT_bits  CHEMBL1945908            0.559524      23.0        *
            FP2  CHEMBL1835544            0.670455      30.0
  hybridization   CHEMBL482212            0.873362     250.0        *
   substructure  CHEMBL1835544            0.866667      30.0        *
          graph  CHEMBL1835544            0.931818      30.0        *
        pubchem  CHEMBL1835544            0.908333      30.0        *
      cdk_maccs  CHEMBL1835544            0.948718      30.0        *
   klekota_roth  CHEMBL1835544            0.797297      30.0        *
      *** ki ACTIVITY *** value prediction
      based on 10 locally validated fgps ---> 97.751
["TT_bits", "substructure", "Pattern", "klekota_roth", "pubchem",
"graph", "AP_bits", "FeatMFP1", "cdk_maccs", "hybridization"]

3 rank
Progesterone receptor : Bos taurus
      score: 8.225 on CHEMBL1909044 based on 10 fingerprints
Fingerprint type        Ligand  Tanimoto Similarity  activity Valid fg
       FeatMFP1     CHEMBL135            0.789474    4247.0        *
           MFP1     CHEMBL135            0.758621    4247.0        *
         RDKit7     CHEMBL135            0.722429    4247.0
        Pattern  CHEMBL282575            0.919105    1253.0        *
        AP_bits     CHEMBL135            0.558491    4247.0        *
        TT_bits     CHEMBL135            0.742424    4247.0        *
            FP2     CHEMBL135            0.675325    4247.0
  hybridization     CHEMBL135            0.905405    4247.0        *
   substructure     CHEMBL135            0.857143    4247.0
          graph    CHEMBL1766            0.914894      11.0        *
        pubchem     CHEMBL135            0.915254    4247.0        *
      cdk_maccs     CHEMBL135            0.842105    4247.0        *
```

```
   klekota_roth     CHEMBL135             0.878788    4247.0        *
      *** ki ACTIVITY *** value prediction
      based on 10 locally validated fgps ---> 2800.454
["TT_bits", "Pattern", "MFP1", "klekota_roth", "pubchem", "graph",
"AP_bits", "FeatMFP1", "cdk_maccs", "hybridization"]

4 rank
Glucocorticoid receptor : Homo sapiens
      score: 8.225 on CHEMBL2034 based on 10 fingerprints
Fingerprint type        Ligand  Tanimoto Similarity  activity Valid fg
       FeatMFP1     CHEMBL135             0.789474  18828.000        *
           MFP1     CHEMBL135             0.758621  18828.000        *
         RDKit7     CHEMBL135             0.722429  18828.000
        Pattern  CHEMBL282575             0.919105   2306.000        *
        AP_bits     CHEMBL135             0.558491  18828.000        *
        TT_bits     CHEMBL135             0.742424  18828.000        *
            FP2     CHEMBL135             0.675325  18828.000
  hybridization     CHEMBL135             0.905405  18828.000        *
   substructure     CHEMBL135             0.857143  18828.000
          graph    CHEMBL1766             0.914894      1.169        *
        pubchem     CHEMBL135             0.915254  18828.000        *
      cdk_maccs     CHEMBL135             0.842105  18828.000        *
   klekota_roth     CHEMBL135             0.878788  18828.000        *
      *** ki ACTIVITY *** value prediction
      based on 10 locally validated fgps ---> 13409.674
["TT_bits", "Pattern", "MFP1", "klekota_roth", "pubchem", "graph",
"AP_bits", "FeatMFP1", "cdk_maccs", "hybridization"]

5 rank
Androgen Receptor : Rattus norvegicus
      score: 8.206 on CHEMBL3072 based on 10 fingerprints
Fingerprint type         Ligand  Tanimoto Similarity  activity Valid fg
       FeatMFP1      CHEMBL135             0.789474   235.000        *
           MFP1      CHEMBL135             0.758621   235.000        *
         RDKit7      CHEMBL135             0.722429   235.000
        Pattern   CHEMBL282575             0.919105     6.981        *
        AP_bits      CHEMBL135             0.558491   235.000        *
        TT_bits      CHEMBL135             0.742424   235.000        *
            FP2      CHEMBL135             0.675325   235.000
  hybridization      CHEMBL135             0.905405   235.000        *
   substructure      CHEMBL135             0.857143   235.000
          graph  CHEMBL1200600             0.895833   857.000        *
        pubchem      CHEMBL135             0.915254   235.000        *
      cdk_maccs      CHEMBL135             0.842105   235.000        *
   klekota_roth      CHEMBL135             0.878788   235.000        *
      *** ki ACTIVITY *** value prediction
      based on 10 locally validated fgps ---> 1162.333
["TT_bits", "Pattern", "MFP1", "klekota_roth", "pubchem", "graph",
"AP_bits", "FeatMFP1", "cdk_maccs", "hybridization"]

6 rank
Corticosteroid binding globulin : Homo sapiens
      score: 8.148 on CHEMBL2421 based on 10 fingerprints
Fingerprint type        Ligand  Tanimoto Similarity  activity Valid fg
       FeatMFP1     CHEMBL135             0.789474  10000.00        *
           MFP1     CHEMBL135             0.758621  10000.00        *
         RDKit7     CHEMBL135             0.722429  10000.00
        Pattern  CHEMBL193482             0.861888  10000.00        *
        AP_bits     CHEMBL135             0.558491  10000.00        *
        TT_bits     CHEMBL135             0.742424  10000.00        *
```

```
           FP2     CHEMBL135            0.675325  10000.00
  hybridization     CHEMBL135            0.905405  10000.00        *
   substructure     CHEMBL135            0.857143  10000.00
          graph   CHEMBL93948            0.895833   1584.89        *
        pubchem     CHEMBL135            0.915254  10000.00        *
      cdk_maccs     CHEMBL135            0.842105  10000.00        *
   klekota_roth     CHEMBL135            0.878788  10000.00        *
      *** ki ACTIVITY *** value prediction
      based on 10 locally validated fgps ---> 9158.489
["TT_bits", "Pattern", "MFP1", "klekota_roth", "pubchem", "graph",
"AP_bits", "FeatMFP1", "cdk_maccs", "hybridization"]

7 rank
Estrogen receptor alpha : Homo sapiens
      score: 7.336 on CHEMBL206 based on 9 fingerprints
Fingerprint type         Ligand  Tanimoto Similarity  activity Valid fg
       FeatMFP1  CHEMBL1230314            0.789474      4.90        *
           MFP1      CHEMBL135            0.758621      0.16        *
         RDKit7      CHEMBL135            0.722429      0.16
        Pattern   CHEMBL282575            0.919105     18.00        *
        AP_bits      CHEMBL135            0.558491      0.16        *
        TT_bits      CHEMBL135            0.742424      0.16        *
            FP2      CHEMBL135            0.675325      0.16
  hybridization      CHEMBL135            0.905405      0.16        *
   substructure      CHEMBL135            0.857143      0.16
          graph      CHEMBL135            0.813953      0.16
        pubchem      CHEMBL135            0.915254      0.16        *
      cdk_maccs   CHEMBL135236            0.868421      8.00        *
   klekota_roth      CHEMBL135            0.878788      0.16        *
      *** ki ACTIVITY *** value prediction
      based on 9 locally validated fgps ---> 6.522
["TT_bits", "Pattern", "MFP1", "klekota_roth", "pubchem", "AP_bits",
"FeatMFP1", "cdk_maccs", "hybridization"]

8 rank
Serotonin transporter : Homo sapiens
      score: 7.310 on CHEMBL228 based on 9 fingerprints
Fingerprint type        Ligand  Tanimoto Similarity  activity Valid fg
       FeatMFP1     CHEMBL135            0.789474    5714.0        *
           MFP1     CHEMBL135            0.758621    5714.0        *
         RDKit7     CHEMBL135            0.722429    5714.0
        Pattern  CHEMBL282575            0.919105    1951.0        *
        AP_bits     CHEMBL135            0.558491    5714.0        *
        TT_bits     CHEMBL135            0.742424    5714.0        *
            FP2     CHEMBL135            0.675325    5714.0
  hybridization     CHEMBL135            0.905405    5714.0        *
   substructure     CHEMBL135            0.857143    5714.0
          graph     CHEMBL135            0.813953    5714.0
        pubchem     CHEMBL135            0.915254    5714.0        *
      cdk_maccs     CHEMBL135            0.842105    5714.0        *
   klekota_roth     CHEMBL135            0.878788    5714.0        *
      *** ki ACTIVITY *** value prediction
      based on 9 locally validated fgps ---> 4730.993
["TT_bits", "Pattern", "MFP1", "klekota_roth", "pubchem", "AP_bits",
"FeatMFP1", "cdk_maccs", "hybridization"]

9 rank
Estrogen receptor beta : Homo sapiens
      score: 7.308 on CHEMBL242 based on 9 fingerprints
Fingerprint type         Ligand  Tanimoto Similarity  activity Valid fg
```

```
       FeatMFP1  CHEMBL1230314             0.789474    19.000        *
           MFP1      CHEMBL135             0.758621     0.015        *
         RDKit7      CHEMBL135             0.722429     0.015
        Pattern  CHEMBL1627464             0.891192     0.025        *
        AP_bits      CHEMBL135             0.558491     0.015        *
        TT_bits      CHEMBL135             0.742424     0.015        *
            FP2      CHEMBL135             0.675325     0.015
  hybridization      CHEMBL135             0.905405     0.015        *
   substructure      CHEMBL135             0.857143     0.015
          graph      CHEMBL135             0.813953     0.015
        pubchem      CHEMBL135             0.915254     0.015        *
      cdk_maccs   CHEMBL135236             0.868421    24.000        *
   klekota_roth      CHEMBL135             0.878788     0.015        *
      *** ki ACTIVITY *** value prediction
      based on 9 locally validated fgps ---> 7.811
["TT_bits", "Pattern", "MFP1", "klekota_roth", "pubchem", "AP_bits",
"FeatMFP1", "cdk_maccs", "hybridization"]

10 rank
Dopamine transporter : Homo sapiens
      score: 7.282 on CHEMBL238 based on 9 fingerprints
Fingerprint type     Ligand  Tanimoto Similarity  activity Valid fg
       FeatMFP1  CHEMBL135             0.789474   11847.0        *
           MFP1  CHEMBL135             0.758621   11847.0        *
         RDKit7  CHEMBL135             0.722429   11847.0
        Pattern  CHEMBL691             0.891192     444.0        *
        AP_bits  CHEMBL135             0.558491   11847.0        *
        TT_bits  CHEMBL135             0.742424   11847.0        *
            FP2  CHEMBL135             0.675325   11847.0
  hybridization  CHEMBL135             0.905405   11847.0        *
   substructure  CHEMBL135             0.857143   11847.0
          graph  CHEMBL135             0.813953   11847.0
        pubchem  CHEMBL135             0.915254   11847.0        *
      cdk_maccs  CHEMBL135             0.842105   11847.0        *
   klekota_roth  CHEMBL135             0.878788   11847.0        *
      *** ki ACTIVITY *** value prediction
      based on 9 locally validated fgps ---> 9384.404
["TT_bits", "Pattern", "MFP1", "klekota_roth", "pubchem", "AP_bits",
"FeatMFP1", "cdk_maccs", "hybridization"]

11 rank
Aldehyde oxidase : Homo sapiens
      score: 7.282 on CHEMBL3257 based on 9 fingerprints
Fingerprint type     Ligand  Tanimoto Similarity  activity Valid fg
       FeatMFP1  CHEMBL135             0.789474     870.0        *
           MFP1  CHEMBL135             0.758621     870.0        *
         RDKit7  CHEMBL135             0.722429     870.0
        Pattern  CHEMBL691             0.891192     430.0        *
        AP_bits  CHEMBL135             0.558491     870.0        *
        TT_bits  CHEMBL135             0.742424     870.0        *
            FP2  CHEMBL135             0.675325     870.0
  hybridization  CHEMBL135             0.905405     870.0        *
   substructure  CHEMBL135             0.857143     870.0
          graph  CHEMBL135             0.813953     870.0
        pubchem  CHEMBL135             0.915254     870.0        *
      cdk_maccs  CHEMBL135             0.842105     870.0        *
   klekota_roth  CHEMBL135             0.878788     870.0        *
      *** ki ACTIVITY *** value prediction
      based on 9 locally validated fgps ---> 774.977
```

["TT_bits", "Pattern", "MFP1", "klekota_roth", "pubchem", "AP_bits", "FeatMFP1", "cdk_maccs", "hybridization"]

12 rank
Solute carrier organic anion transporter family member 1A1 : Rattus norvegicus
score: 7.274 on CHEMBL1781859 based on 9 fingerprints

Fingerprint type	Ligand	Tanimoto Similarity	activity	Valid fg
FeatMFP1	CHEMBL135	0.789474	4700.0	*
MFP1	CHEMBL135	0.758621	4700.0	*
RDKit7	CHEMBL135	0.722429	4700.0	
Pattern	CHEMBL1628111	0.882960	1100.0	*
AP_bits	CHEMBL135	0.558491	4700.0	*
TT_bits	CHEMBL135	0.742424	4700.0	*
FP2	CHEMBL135	0.675325	4700.0	
hybridization	CHEMBL135	0.905405	4700.0	*
substructure	CHEMBL135	0.857143	4700.0	
graph	CHEMBL135	0.813953	4700.0	
pubchem	CHEMBL135	0.915254	4700.0	*
cdk_maccs	CHEMBL135	0.842105	4700.0	*
klekota_roth	CHEMBL135	0.878788	4700.0	*

*** ki ACTIVITY *** value prediction
based on 9 locally validated fgps ---> 5510.140
["TT_bits", "Pattern", "MFP1", "klekota_roth", "pubchem", "AP_bits", "FeatMFP1", "cdk_maccs", "hybridization"]

13 rank
Estrogen sulfotransferase : Sus scrofa
score: 5.857 on CHEMBL3811 based on 7 fingerprints

Fingerprint type	Ligand	Tanimoto Similarity	activity	Valid fg
FeatMFP1	CHEMBL1627683	0.842105	13600.0	*
MFP1	CHEMBL1627683	0.857143	13600.0	*
RDKit7	CHEMBL194749	0.634449	16300.0	
Pattern	CHEMBL1627977	0.826087	10200.0	
AP_bits	CHEMBL1627683	0.521898	13600.0	
TT_bits	CHEMBL1627683	0.742424	13600.0	*
FP2	CHEMBL1627683	0.597561	13600.0	
hybridization	CHEMBL1627683	0.760331	13600.0	*
substructure	CHEMBL1627683	0.857143	13600.0	
graph	CHEMBL1627977	0.977273	10200.0	*
pubchem	CHEMBL1627977	0.771186	10200.0	
cdk_maccs	CHEMBL1627683	0.868421	13600.0	*
klekota_roth	CHEMBL1627683	0.808824	13600.0	*

*** ki ACTIVITY *** value prediction
based on 7 locally validated fgps ---> 12559.345
["TT_bits", "graph", "MFP1", "klekota_roth", "FeatMFP1", "hybridization", "cdk_maccs"]

14 rank
Carbonic anhydrase II : Homo sapiens
score: 3.784 on CHEMBL205 based on 5 fingerprints

Fingerprint type	Ligand	Tanimoto Similarity	activity	Valid fg
FeatMFP1	CHEMBL2391082	0.625000	21800.0	
MFP1	CHEMBL220493	0.567568	13.0	
RDKit7	CHEMBL220493	0.670366	13.0	
Pattern	CHEMBL425287	0.876206	232.0	*
AP_bits	CHEMBL220493	0.444444	13.0	
TT_bits	CHEMBL220493	0.608108	13.0	*
FP2	CHEMBL220493	0.536082	13.0	
hybridization	CHEMBL220493	0.743590	13.0	*

```
   substructure   CHEMBL220493             0.733333      13.0
          graph      CHEMBL898             0.674419    9370.0
        pubchem   CHEMBL220493             0.856000      13.0         *
      cdk_maccs  CHEMBL1946088             0.600000     380.0
   klekota_roth   CHEMBL220493             0.700000      13.0         *
     *** ki ACTIVITY *** value prediction
     based on 5 locally validated fgps ---> 34.259
["Pattern", "klekota_roth", "TT_bits", "pubchem", "hybridization"]

15 rank
Carbonic anhydrase I : Homo sapiens
     score: 3.773 on CHEMBL261 based on 5 fingerprints
Fingerprint type         Ligand  Tanimoto Similarity  activity Valid fg
       FeatMFP1  CHEMBL2391082             0.625000     760.0
           MFP1   CHEMBL220493             0.567568      15.0
         RDKit7   CHEMBL220493             0.670366      15.0
        Pattern   CHEMBL220493             0.865093      15.0         *
        AP_bits   CHEMBL220493             0.444444      15.0
        TT_bits   CHEMBL220493             0.608108      15.0         *
            FP2   CHEMBL220493             0.536082      15.0
  hybridization   CHEMBL220493             0.743590      15.0         *
   substructure   CHEMBL220493             0.733333      15.0
          graph      CHEMBL898             0.674419    8450.0
        pubchem   CHEMBL220493             0.856000      15.0         *
      cdk_maccs  CHEMBL1946088             0.600000    1040.0
   klekota_roth   CHEMBL220493             0.700000      15.0         *
     *** ki ACTIVITY *** value prediction
     based on 5 locally validated fgps ---> 1197.155
["Pattern", "klekota_roth", "TT_bits", "pubchem", "hybridization"]

16 rank
Carbonic anhydrase IX : Homo sapiens
     score: 3.773 on CHEMBL3594 based on 5 fingerprints
Fingerprint type         Ligand  Tanimoto Similarity  activity Valid fg
       FeatMFP1  CHEMBL2391082             0.625000   15700.0
           MFP1   CHEMBL220493             0.567568      32.0
         RDKit7   CHEMBL220493             0.670366      32.0
        Pattern   CHEMBL220493             0.865093      32.0         *
        AP_bits   CHEMBL220493             0.444444      32.0
        TT_bits   CHEMBL220493             0.608108      32.0         *
            FP2   CHEMBL220493             0.536082      32.0
  hybridization   CHEMBL220493             0.743590      32.0         *
   substructure   CHEMBL220493             0.733333      32.0
          graph  CHEMBL1628004             0.561644    1725.0
        pubchem   CHEMBL220493             0.856000      32.0         *
      cdk_maccs  CHEMBL2391082             0.551020   15700.0
   klekota_roth   CHEMBL220493             0.700000      32.0         *
     *** ki ACTIVITY *** value prediction
     based on 5 locally validated fgps ---> 37.136
["Pattern", "klekota_roth", "TT_bits", "pubchem", "hybridization"]

17 rank
Norepinephrine transporter : Homo sapiens
     score: 3.693 on CHEMBL222 based on 5 fingerprints
Fingerprint type         Ligand  Tanimoto Similarity  activity Valid fg
       FeatMFP1      CHEMBL691             0.636364     951.0
           MFP1   CHEMBL282575             0.583333   17087.0
         RDKit7      CHEMBL691             0.688940     951.0
        Pattern   CHEMBL282575             0.919105   17087.0         *
        AP_bits   CHEMBL282575             0.538905   17087.0         *
```

TT_bits	CHEMBL282575	0.641026	17087.0	*
FP2	CHEMBL691	0.619048	951.0	
hybridization	CHEMBL691	0.816327	951.0	*
substructure	CHEMBL1601669	0.687500	1000.0	
graph	CHEMBL1200623	0.790698	2080.0	
pubchem	CHEMBL691	0.830645	951.0	
cdk_maccs	CHEMBL691	0.820513	951.0	
klekota_roth	CHEMBL691	0.777778	951.0	*

*** ki ACTIVITY *** value prediction
based on 5 locally validated fgps ---> 10631.626
["Pattern", "hybridization", "TT_bits", "klekota_roth", "AP_bits"]

18 rank
Adenosine A3 receptor : Homo sapiens
score: 3.693 on CHEMBL256 based on 5 fingerprints

Fingerprint type	Ligand	Tanimoto Similarity	activity	Valid fg
FeatMFP1	CHEMBL691	0.636364	18610.0	
MFP1	CHEMBL282575	0.583333	12312.0	
RDKit7	CHEMBL691	0.688940	18610.0	
Pattern	CHEMBL282575	0.919105	12312.0	*
AP_bits	CHEMBL282575	0.538905	12312.0	*
TT_bits	CHEMBL282575	0.641026	12312.0	*
FP2	CHEMBL691	0.619048	18610.0	
hybridization	CHEMBL691	0.816327	18610.0	*
substructure	CHEMBL691	0.687500	18610.0	
graph	CHEMBL691	0.767442	18610.0	
pubchem	CHEMBL691	0.830645	18610.0	
cdk_maccs	CHEMBL691	0.820513	18610.0	
klekota_roth	CHEMBL691	0.777778	18610.0	*

*** ki ACTIVITY *** value prediction
based on 5 locally validated fgps ---> 14831.580
["Pattern", "hybridization", "TT_bits", "klekota_roth", "AP_bits"]

19 rank
Serotonin 2b (5-HT2b) receptor : Homo sapiens
score: 3.627 on CHEMBL1833 based on 5 fingerprints

Fingerprint type	Ligand	Tanimoto Similarity	activity	Valid fg
FeatMFP1	CHEMBL1405	0.590909	8676.0	
MFP1	CHEMBL282575	0.583333	7359.0	
RDKit7	CHEMBL282575	0.634737	7359.0	
Pattern	CHEMBL282575	0.919105	7359.0	*
AP_bits	CHEMBL282575	0.538905	7359.0	*
TT_bits	CHEMBL282575	0.641026	7359.0	*
FP2	CHEMBL1405	0.566265	8676.0	
hybridization	CHEMBL1405	0.775000	8676.0	*
substructure	CHEMBL1405	0.666667	8676.0	
graph	CHEMBL1405	0.767442	8676.0	
pubchem	CHEMBL1405	0.801587	8676.0	
cdk_maccs	CHEMBL282575	0.761905	7359.0	
klekota_roth	CHEMBL282575	0.753247	7359.0	*

*** ki ACTIVITY *** value prediction
based on 5 locally validated fgps ---> 7622.400
["Pattern", "hybridization", "TT_bits", "klekota_roth", "AP_bits"]

20 rank
Multidrug resistance-associated protein 1 : Homo sapiens
score: 3.202 on CHEMBL3004 based on 4 fingerprints

Fingerprint type	Ligand	Tanimoto Similarity	activity	Valid fg
FeatMFP1	CHEMBL1697724	0.535714	22000.0	
MFP1	CHEMBL1628111	0.583333	380.0	

```
        RDKit7  CHEMBL1628111             0.651366     380.0
       Pattern  CHEMBL1628111             0.882960     380.0        *
       AP_bits  CHEMBL1628111             0.470238     380.0
       TT_bits  CHEMBL1628111             0.666667     380.0        *
           FP2  CHEMBL1628111             0.525253     380.0
 hybridization  CHEMBL1628111             0.689420     380.0
  substructure  CHEMBL1628111             0.631579     380.0
         graph  CHEMBL1628111             0.564516     380.0
       pubchem  CHEMBL1628111             0.868852     380.0        *
     cdk_maccs  CHEMBL2075011             0.607843    1400.0
  klekota_roth  CHEMBL1628111             0.783784     380.0        *
     *** ki ACTIVITY *** value prediction
     based on 4 locally validated fgps ---> 388.636
["Pattern", "klekota_roth", "TT_bits", "pubchem"]

21 rank
Alpha-2c adrenergic receptor : Homo sapiens
     score: 2.852 on CHEMBL1916 based on 4 fingerprints
Fingerprint type        Ligand  Tanimoto Similarity  activity Valid fg
      FeatMFP1  CHEMBL282575             0.541667    3582.0
          MFP1  CHEMBL282575             0.583333    3582.0
        RDKit7  CHEMBL282575             0.634737    3582.0
       Pattern  CHEMBL282575             0.919105    3582.0        *
       AP_bits  CHEMBL282575             0.538905    3582.0        *
       TT_bits  CHEMBL282575             0.641026    3582.0        *
           FP2  CHEMBL282575             0.518868    3582.0
 hybridization  CHEMBL282575             0.681063    3582.0
  substructure  CHEMBL282575             0.666667    3582.0
         graph  CHEMBL282575             0.507246    3582.0
       pubchem  CHEMBL282575             0.746575    3582.0
     cdk_maccs  CHEMBL282575             0.761905    3582.0
  klekota_roth  CHEMBL282575             0.753247    3582.0        *
     *** ki ACTIVITY *** value prediction
     based on 4 locally validated fgps ---> 3582.000
["Pattern", "klekota_roth", "TT_bits", "AP_bits"]
```

MuSSel Prediction IC_{50}:

```
1 rank
Steryl-sulfatase : Homo sapiens
      score: 10.910 on CHEMBL3559 based on 13 fingerprints
Fingerprint type          Ligand  Tanimoto Similarity  activity Valid fg
       FeatMFP1  CHEMBL1627410             0.850000     840.0        *
           MFP1      CHEMBL135             0.758621   84000.0        *
         RDKit7  CHEMBL1947227             0.751716      35.0        *
        Pattern   CHEMBL482212             0.945111     220.0        *
        AP_bits  CHEMBL1947227             0.571053      35.0        *
        TT_bits      CHEMBL135             0.742424   84000.0        *
            FP2  CHEMBL1947227             0.762500      35.0        *
  hybridization  CHEMBL1627953             0.908297     120.0        *
   substructure   CHEMBL270250             0.928571      62.0        *
          graph  CHEMBL1823787             0.931818     126.0        *
        pubchem   CHEMBL270250             0.932773      62.0        *
      cdk_maccs  CHEMBL1627640             0.948718     130.0        *
   klekota_roth      CHEMBL135             0.878788   84000.0        *
      *** ic50 ACTIVITY *** value prediction
      based on 13 locally validated fgps ---> 16832.382
["TT_bits", "substructure", "cdk_maccs", "Pattern", "FP2", "MFP1",
"klekota_roth", "pubchem", "graph", "AP_bits", "FeatMFP1", "RDKit7",
"hybridization"]

2 rank
Tubulin beta chain : Bos taurus
      score: 10.783 on CHEMBL3394 based on 13 fingerprints
Fingerprint type          Ligand  Tanimoto Similarity  activity Valid fg
       FeatMFP1   CHEMBL265926             0.947368    4800.0        *
           MFP1   CHEMBL241993             0.733333   17000.0        *
         RDKit7   CHEMBL241993             0.692575   17000.0        *
        Pattern  CHEMBL1627838             0.903169    8600.0        *
        AP_bits  CHEMBL1627439             0.618056    1100.0        *
        TT_bits  CHEMBL1627439             0.787879    1100.0        *
            FP2   CHEMBL241993             0.727273   17000.0        *
  hybridization  CHEMBL1628145             0.945946    2400.0        *
   substructure   CHEMBL389126             0.928571    4900.0        *
          graph  CHEMBL1627436             0.813953    9400.0        *
        pubchem  CHEMBL1627439             0.932773    1100.0        *
      cdk_maccs   CHEMBL265926             0.897436    4800.0        *
   klekota_roth   CHEMBL265926             0.855072    4800.0        *
      *** ic50 ACTIVITY *** value prediction
      based on 13 locally validated fgps ---> 7904.002
["TT_bits", "substructure", "cdk_maccs", "Pattern", "FP2", "MFP1",
"klekota_roth", "pubchem", "graph", "AP_bits", "FeatMFP1", "RDKit7",
"hybridization"]

3 rank
Glucocorticoid receptor : Homo sapiens
      score: 10.562 on CHEMBL2034 based on 13 fingerprints
Fingerprint type          Ligand  Tanimoto Similarity   activity Valid fg
       FeatMFP1      CHEMBL135             0.789474  41422.000       *
           MFP1      CHEMBL135             0.758621  41422.000       *
         RDKit7      CHEMBL135             0.722429  41422.000       *
        Pattern   CHEMBL282575             0.919105   5074.000       *
        AP_bits      CHEMBL135             0.558491  41422.000       *
        TT_bits      CHEMBL135             0.742424  41422.000       *
            FP2      CHEMBL135             0.675325  41422.000       *
  hybridization      CHEMBL135             0.905405  41422.000       *
   substructure   CHEMBL469944             0.928571      1.300       *
          graph     CHEMBL1766             0.914894      2.573       *
```

pubchem	CHEMBL135	0.915254	41422.000	*
cdk_maccs	CHEMBL1256102	0.853659	15848.930	*
klekota_roth	CHEMBL135	0.878788	41422.000	*

*** ic50 ACTIVITY *** value prediction
based on 13 locally validated fgps ---> 26907.660
["TT_bits", "substructure", "cdk_maccs", "Pattern", "FP2", "MFP1", "klekota_roth", "pubchem", "graph", "AP_bits", "FeatMFP1", "RDKit7", "hybridization"]

4 rank
Androgen Receptor : Rattus norvegicus
score: 10.555 on CHEMBL3072 based on 13 fingerprints

Fingerprint type	Ligand	Tanimoto Similarity	activity	Valid fg
FeatMFP1	CHEMBL135	0.789474	353.00	*
MFP1	CHEMBL467987	0.785714	8511.38	*
RDKit7	CHEMBL135	0.722429	353.00	*
Pattern	CHEMBL282575	0.919105	10.00	*
AP_bits	CHEMBL467987	0.572993	8511.38	*
TT_bits	CHEMBL467987	0.757576	8511.38	*
FP2	CHEMBL135	0.675325	353.00	*
hybridization	CHEMBL135	0.905405	353.00	*
substructure	CHEMBL135	0.857143	353.00	*
graph	CHEMBL139841	0.933333	41200.00	*
pubchem	CHEMBL135	0.915254	353.00	*
cdk_maccs	CHEMBL135	0.842105	353.00	*
klekota_roth	CHEMBL135	0.878788	353.00	*

*** ic50 ACTIVITY *** value prediction
based on 13 locally validated fgps ---> 23995.105
["TT_bits", "substructure", "cdk_maccs", "Pattern", "FP2", "MFP1", "klekota_roth", "pubchem", "graph", "AP_bits", "FeatMFP1", "RDKit7", "hybridization"]

5 rank
Estrogen receptor alpha : Homo sapiens
score: 10.539 on CHEMBL206 based on 13 fingerprints

Fingerprint type	Ligand	Tanimoto Similarity	activity	Valid fg
FeatMFP1	CHEMBL135	0.789474	0.41	*
MFP1	CHEMBL135	0.758621	0.41	*
RDKit7	CHEMBL135	0.722429	0.41	*
Pattern	CHEMBL282575	0.919105	65.00	*
AP_bits	CHEMBL135	0.558491	0.41	*
TT_bits	CHEMBL135	0.742424	0.41	*
FP2	CHEMBL135	0.675325	0.41	*
hybridization	CHEMBL135	0.905405	0.41	*
substructure	CHEMBL1628029	0.928571	193.00	*
graph	CHEMBL1257167	0.891304	15848.93	*
pubchem	CHEMBL135	0.915254	0.41	*
cdk_maccs	CHEMBL1256102	0.853659	15848.93	*
klekota_roth	CHEMBL135	0.878788	0.41	*

*** ic50 ACTIVITY *** value prediction
based on 13 locally validated fgps ---> 4983.664
["TT_bits", "substructure", "cdk_maccs", "Pattern", "FP2", "MFP1", "klekota_roth", "pubchem", "graph", "AP_bits", "FeatMFP1", "RDKit7", "hybridization"]

6 rank
Estrogen receptor beta : Homo sapiens
score: 10.520 on CHEMBL242 based on 13 fingerprints

Fingerprint type	Ligand	Tanimoto Similarity	activity	Valid fg
FeatMFP1	CHEMBL135	0.789474	0.046	*

MFP1	CHEMBL135	0.758621	0.046	*
RDKit7	CHEMBL135	0.722429	0.046	*
Pattern	CHEMBL511812	0.900000	660.000	*
AP_bits	CHEMBL135	0.558491	0.046	*
TT_bits	CHEMBL135	0.742424	0.046	*
FP2	CHEMBL135	0.675325	0.046	*
hybridization	CHEMBL135	0.905405	0.046	*
substructure	CHEMBL1628029	0.928571	267.000	*
graph	CHEMBL1257167	0.891304	4045.760	*
pubchem	CHEMBL135	0.915254	0.046	*
cdk_maccs	CHEMBL1256102	0.853659	11857.690	*
klekota_roth	CHEMBL135	0.878788	0.046	*

*** ic50 ACTIVITY *** value prediction
based on 13 locally validated fgps ---> 1987.543
["TT_bits", "substructure", "cdk_maccs", "Pattern", "FP2", "MFP1", "klekota_roth", "pubchem", "graph", "AP_bits", "FeatMFP1", "RDKit7", "hybridization"]

7 rank
Progesterone receptor : Bos taurus
score: 10.479 on CHEMBL1909044 based on 13 fingerprints

Fingerprint type	Ligand	Tanimoto Similarity	activity	Valid fg
FeatMFP1	CHEMBL135	0.789474	32559.0	*
MFP1	CHEMBL135	0.758621	32559.0	*
RDKit7	CHEMBL135	0.722429	32559.0	*
Pattern	CHEMBL282575	0.919105	9608.0	*
AP_bits	CHEMBL135	0.558491	32559.0	*
TT_bits	CHEMBL135	0.742424	32559.0	*
FP2	CHEMBL135	0.675325	32559.0	*
hybridization	CHEMBL135	0.905405	32559.0	*
substructure	CHEMBL135	0.857143	32559.0	*
graph	CHEMBL1766	0.914894	84.0	*
pubchem	CHEMBL135	0.915254	32559.0	*
cdk_maccs	CHEMBL135	0.842105	32559.0	*
klekota_roth	CHEMBL135	0.878788	32559.0	*

*** ic50 ACTIVITY *** value prediction
based on 13 locally validated fgps ---> 19730.947
["TT_bits", "substructure", "cdk_maccs", "Pattern", "FP2", "MFP1", "klekota_roth", "pubchem", "graph", "AP_bits", "FeatMFP1", "RDKit7", "hybridization"]

8 rank
Androgen Receptor : Homo sapiens
score: 10.462 on CHEMBL1871 based on 13 fingerprints

Fingerprint type	Ligand	Tanimoto Similarity	activity	Valid fg
FeatMFP1	CHEMBL135	0.789474	19.1	*
MFP1	CHEMBL135	0.758621	19.1	*
RDKit7	CHEMBL135	0.722429	19.1	*
Pattern	CHEMBL3421871	0.881864	300.0	*
AP_bits	CHEMBL135	0.558491	19.1	*
TT_bits	CHEMBL135	0.742424	19.1	*
FP2	CHEMBL135	0.675325	19.1	*
hybridization	CHEMBL135	0.905405	19.1	*
substructure	CHEMBL135	0.857143	19.1	*
graph	CHEMBL236699	0.934783	410.0	*
pubchem	CHEMBL135	0.915254	19.1	*
cdk_maccs	CHEMBL135	0.842105	19.1	*
klekota_roth	CHEMBL135	0.878788	19.1	*

*** ic50 ACTIVITY *** value prediction
based on 13 locally validated fgps ---> 319.119

["TT_bits", "substructure", "cdk_maccs", "Pattern", "FP2", "MFP1", "klekota_roth", "pubchem", "graph", "AP_bits", "FeatMFP1", "RDKit7", "hybridization"]

9 rank
Serotonin transporter : Homo sapiens
score: 10.379 on CHEMBL228 based on 13 fingerprints

Fingerprint type	Ligand	Tanimoto Similarity	activity	Valid fg
FeatMFP1	CHEMBL135	0.789474	10755.0	*
MFP1	CHEMBL135	0.758621	10755.0	*
RDKit7	CHEMBL135	0.722429	10755.0	*
Pattern	CHEMBL282575	0.919105	3672.0	*
AP_bits	CHEMBL135	0.558491	10755.0	*
TT_bits	CHEMBL135	0.742424	10755.0	*
FP2	CHEMBL135	0.675325	10755.0	*
hybridization	CHEMBL135	0.905405	10755.0	*
substructure	CHEMBL135	0.857143	10755.0	*
graph	CHEMBL135	0.813953	10755.0	*
pubchem	CHEMBL135	0.915254	10755.0	*
cdk_maccs	CHEMBL135	0.842105	10755.0	*
klekota_roth	CHEMBL135	0.878788	10755.0	*

*** ic50 ACTIVITY *** value prediction
based on 13 locally validated fgps ---> 8800.438
["TT_bits", "substructure", "cdk_maccs", "Pattern", "FP2", "MFP1", "klekota_roth", "pubchem", "graph", "AP_bits", "FeatMFP1", "RDKit7", "hybridization"]

10 rank
Dopamine transporter : Homo sapiens
score: 10.351 on CHEMBL238 based on 13 fingerprints

Fingerprint type	Ligand	Tanimoto Similarity	activity	Valid fg
FeatMFP1	CHEMBL135	0.789474	14911.0	*
MFP1	CHEMBL135	0.758621	14911.0	*
RDKit7	CHEMBL135	0.722429	14911.0	*
Pattern	CHEMBL691	0.891192	559.0	*
AP_bits	CHEMBL135	0.558491	14911.0	*
TT_bits	CHEMBL135	0.742424	14911.0	*
FP2	CHEMBL135	0.675325	14911.0	*
hybridization	CHEMBL135	0.905405	14911.0	*
substructure	CHEMBL135	0.857143	14911.0	*
graph	CHEMBL135	0.813953	14911.0	*
pubchem	CHEMBL135	0.915254	14911.0	*
cdk_maccs	CHEMBL135	0.842105	14911.0	*
klekota_roth	CHEMBL135	0.878788	14911.0	*

*** ic50 ACTIVITY *** value prediction
based on 13 locally validated fgps ---> 10703.291
["TT_bits", "substructure", "cdk_maccs", "Pattern", "FP2", "MFP1", "klekota_roth", "pubchem", "graph", "AP_bits", "FeatMFP1", "RDKit7", "hybridization"]

11 rank
Aldehyde oxidase : Homo sapiens
score: 10.351 on CHEMBL3257 based on 13 fingerprints

Fingerprint type	Ligand	Tanimoto Similarity	activity	Valid fg
FeatMFP1	CHEMBL135	0.789474	80.0	*
MFP1	CHEMBL135	0.758621	80.0	*
RDKit7	CHEMBL135	0.722429	80.0	*
Pattern	CHEMBL691	0.891192	570.0	*
AP_bits	CHEMBL135	0.558491	80.0	*
TT_bits	CHEMBL135	0.742424	80.0	*

```
            FP2  CHEMBL135             0.675325      80.0        *
  hybridization  CHEMBL135             0.905405      80.0        *
   substructure  CHEMBL135             0.857143      80.0        *
          graph  CHEMBL135             0.813953      80.0        *
        pubchem  CHEMBL135             0.915254      80.0        *
      cdk_maccs  CHEMBL135             0.842105      80.0        *
   klekota_roth  CHEMBL135             0.878788      80.0        *
     *** ic50 ACTIVITY *** value prediction
     based on 13 locally validated fgps ---> 223.658
["TT_bits", "substructure", "cdk_maccs", "Pattern", "FP2", "MFP1",
"klekota_roth", "pubchem", "graph", "AP_bits", "FeatMFP1", "RDKit7",
"hybridization"]

12 rank
Arachidonate 15-lipoxygenase : Oryctolagus cuniculus
     score: 10.351 on CHEMBL4358 based on 13 fingerprints
Fingerprint type     Ligand  Tanimoto Similarity  activity Valid fg
       FeatMFP1  CHEMBL135             0.789474   11295.0        *
           MFP1  CHEMBL135             0.758621   11295.0        *
         RDKit7  CHEMBL135             0.722429   11295.0        *
        Pattern  CHEMBL691             0.891192    5678.0        *
        AP_bits  CHEMBL135             0.558491   11295.0        *
        TT_bits  CHEMBL135             0.742424   11295.0        *
            FP2  CHEMBL135             0.675325   11295.0        *
  hybridization  CHEMBL135             0.905405   11295.0        *
   substructure  CHEMBL135             0.857143   11295.0        *
          graph  CHEMBL135             0.813953   11295.0        *
        pubchem  CHEMBL135             0.915254   11295.0        *
      cdk_maccs  CHEMBL135             0.842105   11295.0        *
   klekota_roth  CHEMBL135             0.878788   11295.0        *
     *** ic50 ACTIVITY *** value prediction
     based on 13 locally validated fgps ---> 9771.506
["TT_bits", "substructure", "cdk_maccs", "Pattern", "FP2", "MFP1",
"klekota_roth", "pubchem", "graph", "AP_bits", "FeatMFP1", "RDKit7",
"hybridization"]

13 rank
Estrogen-related receptor alpha : Homo sapiens
     score: 10.317 on CHEMBL3429 based on 13 fingerprints
Fingerprint type         Ligand  Tanimoto Similarity  activity Valid fg
       FeatMFP1      CHEMBL135             0.789474       3.6        *
           MFP1      CHEMBL135             0.758621       3.6        *
         RDKit7      CHEMBL135             0.722429       3.6        *
        Pattern  CHEMBL2326071             0.857143     637.8        *
        AP_bits      CHEMBL135             0.558491       3.6        *
        TT_bits      CHEMBL135             0.742424       3.6        *
            FP2      CHEMBL135             0.675325       3.6        *
  hybridization      CHEMBL135             0.905405       3.6        *
   substructure      CHEMBL135             0.857143       3.6        *
          graph      CHEMBL135             0.813953       3.6        *
        pubchem      CHEMBL135             0.915254       3.6        *
      cdk_maccs      CHEMBL135             0.842105       3.6        *
   klekota_roth      CHEMBL135             0.878788       3.6        *
     *** ic50 ACTIVITY *** value prediction
     based on 13 locally validated fgps ---> 72.934
["TT_bits", "substructure", "cdk_maccs", "Pattern", "FP2", "MFP1",
"klekota_roth", "pubchem", "graph", "AP_bits", "FeatMFP1", "RDKit7",
"hybridization"]

14 rank
```

```
Estrogen-related receptor beta : Homo sapiens
      score: 10.317 on CHEMBL3751 based on 13 fingerprints
Fingerprint type          Ligand  Tanimoto Similarity  activity Valid fg
       FeatMFP1      CHEMBL135             0.789474       3.2         *
           MFP1      CHEMBL135             0.758621       3.2         *
         RDKit7      CHEMBL135             0.722429       3.2         *
        Pattern  CHEMBL2326071             0.857143     565.9         *
        AP_bits      CHEMBL135             0.558491       3.2         *
        TT_bits      CHEMBL135             0.742424       3.2         *
            FP2      CHEMBL135             0.675325       3.2         *
  hybridization      CHEMBL135             0.905405       3.2         *
   substructure      CHEMBL135             0.857143       3.2         *
          graph      CHEMBL135             0.813953       3.2         *
        pubchem      CHEMBL135             0.915254       3.2         *
      cdk_maccs      CHEMBL135             0.842105       3.2         *
   klekota_roth      CHEMBL135             0.878788       3.2         *
      *** ic50 ACTIVITY *** value prediction
      based on 13 locally validated fgps ---> 55.498
["TT_bits", "substructure", "cdk_maccs", "Pattern", "FP2", "MFP1",
"klekota_roth", "pubchem", "graph", "AP_bits", "FeatMFP1", "RDKit7",
"hybridization"]

15 rank
Estradiol 17-beta-dehydrogenase 1 : Homo sapiens
      score: 10.255 on CHEMBL3181 based on 13 fingerprints
Fingerprint type          Ligand  Tanimoto Similarity  activity Valid fg
       FeatMFP1   CHEMBL270250             0.789474     790.0         *
           MFP1  CHEMBL1627884             0.611111    4300.0         *
         RDKit7  CHEMBL1627455             0.711165    1000.0         *
        Pattern   CHEMBL270250             0.943860     790.0         *
        AP_bits  CHEMBL1627453             0.552632     420.0         *
        TT_bits  CHEMBL1627884             0.578947    4300.0         *
            FP2   CHEMBL270250             0.666667     790.0         *
  hybridization   CHEMBL270250             0.881579     790.0         *
   substructure   CHEMBL270250             0.928571     790.0         *
          graph  CHEMBL1627884             0.977273    4300.0         *
        pubchem   CHEMBL270250             0.932773     790.0         *
      cdk_maccs  CHEMBL1079595             0.800000     140.0         *
   klekota_roth  CHEMBL1627884             0.880597    4300.0         *
      *** ic50 ACTIVITY *** value prediction
      based on 12 locally validated fgps ---> 1724.509
["TT_bits", "substructure", "Pattern", "FP2", "MFP1", "klekota_roth",
"pubchem", "graph", "AP_bits", "FeatMFP1", "RDKit7", "hybridization"]

16 rank
Solute carrier family 22 member 2 : Rattus norvegicus
      score: 9.459 on CHEMBL1770032 based on 12 fingerprints
Fingerprint type      Ligand  Tanimoto Similarity  activity Valid fg
       FeatMFP1  CHEMBL135             0.789474   84800.0         *
           MFP1  CHEMBL135             0.758621   84800.0         *
         RDKit7  CHEMBL135             0.722429   84800.0         *
        Pattern  CHEMBL135             0.831858   84800.0
        AP_bits  CHEMBL135             0.558491   84800.0         *
        TT_bits  CHEMBL135             0.742424   84800.0         *
            FP2  CHEMBL135             0.675325   84800.0         *
  hybridization  CHEMBL135             0.905405   84800.0         *
   substructure  CHEMBL135             0.857143   84800.0         *
          graph  CHEMBL135             0.813953   84800.0         *
        pubchem  CHEMBL135             0.915254   84800.0         *
      cdk_maccs  CHEMBL135             0.842105   84800.0         *
```

```
   klekota_roth  CHEMBL135             0.878788   84800.0        *
      *** ic50 ACTIVITY *** value prediction
      based on 12 locally validated fgps ---> 81435.582
["TT_bits", "substructure", "cdk_maccs", "AP_bits", "MFP1",
"klekota_roth", "pubchem", "graph", "FP2", "FeatMFP1", "RDKit7",
"hybridization"]

17 rank
Voltage-gated L-type calcium channel alpha-1C subunit : Homo sapiens
      score: 9.459 on CHEMBL1940 based on 12 fingerprints
Fingerprint type     Ligand  Tanimoto Similarity  activity Valid fg
       FeatMFP1  CHEMBL135             0.789474   50000.0        *
           MFP1  CHEMBL135             0.758621   50000.0        *
         RDKit7  CHEMBL135             0.722429   50000.0        *
        Pattern  CHEMBL135             0.831858   50000.0
        AP_bits  CHEMBL135             0.558491   50000.0        *
        TT_bits  CHEMBL135             0.742424   50000.0        *
            FP2  CHEMBL135             0.675325   50000.0        *
  hybridization  CHEMBL135             0.905405   50000.0        *
   substructure  CHEMBL135             0.857143   50000.0        *
          graph  CHEMBL135             0.813953   50000.0        *
        pubchem  CHEMBL135             0.915254   50000.0        *
      cdk_maccs  CHEMBL135             0.842105   50000.0        *
   klekota_roth  CHEMBL135             0.878788   50000.0        *
      *** ic50 ACTIVITY *** value prediction
      based on 12 locally validated fgps ---> 47918.083
["TT_bits", "substructure", "cdk_maccs", "AP_bits", "MFP1",
"klekota_roth", "pubchem", "graph", "FP2", "FeatMFP1", "RDKit7",
"hybridization"]

18 rank
Solute carrier family 22 member 3 : Homo sapiens
      score: 9.459 on CHEMBL2073673 based on 12 fingerprints
Fingerprint type     Ligand  Tanimoto Similarity  activity Valid fg
       FeatMFP1  CHEMBL135             0.789474    2880.0        *
           MFP1  CHEMBL135             0.758621    2880.0        *
         RDKit7  CHEMBL135             0.722429    2880.0        *
        Pattern  CHEMBL135             0.831858    2880.0
        AP_bits  CHEMBL135             0.558491    2880.0        *
        TT_bits  CHEMBL135             0.742424    2880.0        *
            FP2  CHEMBL135             0.675325    2880.0        *
  hybridization  CHEMBL135             0.905405    2880.0        *
   substructure  CHEMBL135             0.857143    2880.0        *
          graph  CHEMBL135             0.813953    2880.0        *
        pubchem  CHEMBL135             0.915254    2880.0        *
      cdk_maccs  CHEMBL135             0.842105    2880.0        *
   klekota_roth  CHEMBL135             0.878788    2880.0        *
      *** ic50 ACTIVITY *** value prediction
      based on 12 locally validated fgps ---> 2880.000
["TT_bits", "substructure", "cdk_maccs", "AP_bits", "MFP1",
"klekota_roth", "pubchem", "graph", "FP2", "FeatMFP1", "RDKit7",
"hybridization"]

19 rank
Estrogen receptor alpha : Rattus norvegicus
      score: 9.459 on CHEMBL2724 based on 12 fingerprints
Fingerprint type     Ligand  Tanimoto Similarity  activity Valid fg
       FeatMFP1  CHEMBL135             0.789474       1.9        *
           MFP1  CHEMBL135             0.758621       1.9        *
         RDKit7  CHEMBL135             0.722429       1.9        *
```

```
        Pattern  CHEMBL135             0.831858       1.9
        AP_bits  CHEMBL135             0.558491       1.9         *
        TT_bits  CHEMBL135             0.742424       1.9         *
            FP2  CHEMBL135             0.675325       1.9         *
  hybridization  CHEMBL135             0.905405       1.9         *
   substructure  CHEMBL135             0.857143       1.9         *
          graph  CHEMBL135             0.813953       1.9         *
        pubchem  CHEMBL135             0.915254       1.9         *
      cdk_maccs  CHEMBL135             0.842105       1.9         *
   klekota_roth  CHEMBL135             0.878788       1.9         *
      *** ic50 ACTIVITY *** value prediction
      based on 12 locally validated fgps ---> 2.190
["TT_bits", "substructure", "cdk_maccs", "AP_bits", "MFP1",
"klekota_roth", "pubchem", "graph", "FP2", "FeatMFP1", "RDKit7",
"hybridization"]

20 rank
Estrogen receptor beta : Rattus norvegicus
      score: 9.459 on CHEMBL3021 based on 12 fingerprints
Fingerprint type     Ligand  Tanimoto Similarity  activity Valid fg
       FeatMFP1  CHEMBL135             0.789474       1.7         *
           MFP1  CHEMBL135             0.758621       1.7         *
         RDKit7  CHEMBL135             0.722429       1.7         *
        Pattern  CHEMBL135             0.831858       1.7
        AP_bits  CHEMBL135             0.558491       1.7         *
        TT_bits  CHEMBL135             0.742424       1.7         *
            FP2  CHEMBL135             0.675325       1.7         *
  hybridization  CHEMBL135             0.905405       1.7         *
   substructure  CHEMBL135             0.857143       1.7         *
          graph  CHEMBL135             0.813953       1.7         *
        pubchem  CHEMBL135             0.915254       1.7         *
      cdk_maccs  CHEMBL135             0.842105       1.7         *
   klekota_roth  CHEMBL135             0.878788       1.7         *
      *** ic50 ACTIVITY *** value prediction
      based on 12 locally validated fgps ---> 1.700
["TT_bits", "substructure", "cdk_maccs", "AP_bits", "MFP1",
"klekota_roth", "pubchem", "graph", "FP2", "FeatMFP1", "RDKit7",
"hybridization"]

21 rank
Testis-specific androgen-binding protein : Homo sapiens
      score: 9.459 on CHEMBL3305 based on 12 fingerprints
Fingerprint type     Ligand  Tanimoto Similarity  activity Valid fg
       FeatMFP1  CHEMBL135             0.789474      50.0         *
           MFP1  CHEMBL135             0.758621      50.0         *
         RDKit7  CHEMBL135             0.722429      50.0         *
        Pattern  CHEMBL135             0.831858      50.0
        AP_bits  CHEMBL135             0.558491      50.0         *
        TT_bits  CHEMBL135             0.742424      50.0         *
            FP2  CHEMBL135             0.675325      50.0         *
  hybridization  CHEMBL135             0.905405      50.0         *
   substructure  CHEMBL135             0.857143      50.0         *
          graph  CHEMBL135             0.813953      50.0         *
        pubchem  CHEMBL135             0.915254      50.0         *
      cdk_maccs  CHEMBL135             0.842105      50.0         *
   klekota_roth  CHEMBL135             0.878788      50.0         *
      *** ic50 ACTIVITY *** value prediction
      based on 12 locally validated fgps ---> 446.587
```

```
["TT_bits", "substructure", "cdk_maccs", "AP_bits", "MFP1",
"klekota_roth", "pubchem", "graph", "FP2", "FeatMFP1", "RDKit7",
"hybridization"]

22 rank
Solute carrier family 22 member 1 : Homo sapiens
      score: 9.459 on CHEMBL5685 based on 12 fingerprints
Fingerprint type     Ligand  Tanimoto Similarity  activity Valid fg
       FeatMFP1  CHEMBL135             0.789474    5730.0        *
           MFP1  CHEMBL135             0.758621    5730.0        *
         RDKit7  CHEMBL135             0.722429    5730.0        *
        Pattern  CHEMBL135             0.831858    5730.0
        AP_bits  CHEMBL135             0.558491    5730.0        *
        TT_bits  CHEMBL135             0.742424    5730.0        *
            FP2  CHEMBL135             0.675325    5730.0        *
  hybridization  CHEMBL135             0.905405    5730.0        *
   substructure  CHEMBL135             0.857143    5730.0        *
          graph  CHEMBL135             0.813953    5730.0        *
        pubchem  CHEMBL135             0.915254    5730.0        *
      cdk_maccs  CHEMBL135             0.842105    5730.0        *
   klekota_roth  CHEMBL135             0.878788    5730.0        *
      *** ic50 ACTIVITY *** value prediction
      based on 12 locally validated fgps ---> 7986.176
["TT_bits", "substructure", "cdk_maccs", "AP_bits", "MFP1",
"klekota_roth", "pubchem", "graph", "FP2", "FeatMFP1", "RDKit7",
"hybridization"]

23 rank
Glucose-6-phosphate 1-dehydrogenase : Homo sapiens
      score: 8.592 on CHEMBL5347 based on 11 fingerprints
Fingerprint type         Ligand  Tanimoto Similarity  activity Valid fg
       FeatMFP1  CHEMBL1412489             0.789474   29100.0        *
           MFP1   CHEMBL467987             0.785714   72500.0        *
         RDKit7   CHEMBL467987             0.702768   72500.0        *
        Pattern  CHEMBL1412489             0.874346   29100.0        *
        AP_bits   CHEMBL467987             0.572993   72500.0        *
        TT_bits   CHEMBL467987             0.757576   72500.0        *
            FP2   CHEMBL467987             0.666667   72500.0        *
  hybridization   CHEMBL467987             0.873913   72500.0        *
   substructure  CHEMBL1412489             0.800000   29100.0
          graph  CHEMBL2057301             0.813953    4300.0        *
        pubchem  CHEMBL1412489             0.887097   29100.0        *
      cdk_maccs  CHEMBL1412489             0.780488   29100.0
   klekota_roth   CHEMBL467987             0.867647   72500.0        *
      *** ic50 ACTIVITY *** value prediction
      based on 11 locally validated fgps ---> 51686.893
["TT_bits", "Pattern", "FP2", "MFP1", "klekota_roth", "pubchem", "graph",
"AP_bits", "FeatMFP1", "RDKit7", "hybridization"]

24 rank
Glucose-6-phosphate dehydrogenase-6-phosphogluconolactonase : Plasmodium
berghei
      score: 8.556 on CHEMBL1741212 based on 11 fingerprints
Fingerprint type         Ligand  Tanimoto Similarity  activity Valid fg
       FeatMFP1  CHEMBL1412489             0.789474    4020.0        *
           MFP1   CHEMBL467987             0.785714   20900.0        *
         RDKit7   CHEMBL467987             0.702768   20900.0        *
        Pattern  CHEMBL1412489             0.874346    4020.0        *
        AP_bits   CHEMBL467987             0.572993   20900.0        *
        TT_bits   CHEMBL467987             0.757576   20900.0        *
```

```
           FP2   CHEMBL467987            0.666667   20900.0        *
  hybridization   CHEMBL467987            0.873913   20900.0        *
   substructure  CHEMBL1736559            0.812500   35700.0
          graph  CHEMBL1412489            0.777778    4020.0        *
        pubchem  CHEMBL1412489            0.887097    4020.0        *
      cdk_maccs  CHEMBL1412489            0.780488    4020.0
   klekota_roth   CHEMBL467987            0.867647   20900.0        *
      *** ic50 ACTIVITY *** value prediction
      based on 11 locally validated fgps ---> 14726.341
["TT_bits", "Pattern", "FP2", "MFP1", "klekota_roth", "pubchem", "graph",
"AP_bits", "FeatMFP1", "RDKit7", "hybridization"]

25 rank
M18 aspartyl aminopeptidase : Plasmodium falciparum 3D7
      score: 7.575 on CHEMBL1293263 based on 10 fingerprints
Fingerprint type         Ligand  Tanimoto Similarity  activity Valid fg
       FeatMFP1  CHEMBL1412489            0.789474    1220.0        *
           MFP1  CHEMBL1412489            0.700000    1220.0        *
         RDKit7  CHEMBL1412489            0.664062    1220.0
        Pattern  CHEMBL1412489            0.874346    1220.0        *
        AP_bits  CHEMBL1412489            0.569343    1220.0        *
        TT_bits  CHEMBL1412489            0.652778    1220.0        *
            FP2  CHEMBL1412489            0.654321    1220.0        *
  hybridization  CHEMBL1412489            0.841667    1220.0        *
   substructure  CHEMBL1412489            0.800000    1220.0
          graph  CHEMBL1412489            0.777778    1220.0        *
        pubchem  CHEMBL1412489            0.887097    1220.0        *
      cdk_maccs  CHEMBL1412489            0.780488    1220.0
   klekota_roth  CHEMBL1412489            0.828571    1220.0        *
      *** ic50 ACTIVITY *** value prediction
      based on 10 locally validated fgps ---> 1220.000
["TT_bits", "Pattern", "AP_bits", "MFP1", "pubchem", "graph", "FP2",
"FeatMFP1", "klekota_roth", "hybridization"]

26 rank
Mannose-6-phosphate isomerase : Homo sapiens
      score: 7.575 on CHEMBL2758 based on 10 fingerprints
Fingerprint type         Ligand  Tanimoto Similarity  activity Valid fg
       FeatMFP1  CHEMBL1412489            0.789474   29007.0        *
           MFP1  CHEMBL1412489            0.700000   29007.0        *
         RDKit7  CHEMBL1412489            0.664062   29007.0
        Pattern  CHEMBL1412489            0.874346   29007.0        *
        AP_bits  CHEMBL1412489            0.569343   29007.0        *
        TT_bits  CHEMBL1412489            0.652778   29007.0        *
            FP2  CHEMBL1412489            0.654321   29007.0        *
  hybridization  CHEMBL1412489            0.841667   29007.0        *
   substructure  CHEMBL1412489            0.800000   29007.0
          graph  CHEMBL1412489            0.777778   29007.0        *
        pubchem  CHEMBL1412489            0.887097   29007.0        *
      cdk_maccs  CHEMBL1412489            0.780488   29007.0
   klekota_roth  CHEMBL1412489            0.828571   29007.0        *
      *** ic50 ACTIVITY *** value prediction
      based on 10 locally validated fgps ---> 29007.000
["TT_bits", "Pattern", "AP_bits", "MFP1", "pubchem", "graph", "FP2",
"FeatMFP1", "klekota_roth", "hybridization"]

27 rank
Norepinephrine transporter : Homo sapiens
      score: 7.407 on CHEMBL222 based on 10 fingerprints
Fingerprint type         Ligand  Tanimoto Similarity  activity Valid fg
```

FeatMFP1	CHEMBL691	0.636364	959.0	
MFP1	CHEMBL282575	0.583333	17229.0	*
RDKit7	CHEMBL691	0.688940	959.0	*
Pattern	CHEMBL282575	0.919105	17229.0	*
AP_bits	CHEMBL282575	0.538905	17229.0	*
TT_bits	CHEMBL282575	0.641026	17229.0	*
FP2	CHEMBL691	0.619048	959.0	
hybridization	CHEMBL691	0.816327	959.0	*
substructure	CHEMBL3769872	0.733333	33.0	
graph	CHEMBL1200623	0.790698	2098.0	*
pubchem	CHEMBL691	0.830645	959.0	*
cdk_maccs	CHEMBL691	0.820513	959.0	*
klekota_roth	CHEMBL691	0.777778	959.0	*

*** ic50 ACTIVITY *** value prediction
based on 10 locally validated fgps ---> 7547.598
["TT_bits", "cdk_maccs", "Pattern", "MFP1", "klekota_roth", "pubchem", "graph", "AP_bits", "RDKit7", "hybridization"]

28 rank
Adenosine A3 receptor : Homo sapiens
score: 6.617 on CHEMBL256 based on 9 fingerprints

Fingerprint type	Ligand	Tanimoto Similarity	activity	Valid fg
FeatMFP1	CHEMBL691	0.636364	32925.0	
MFP1	CHEMBL282575	0.583333	21780.0	*
RDKit7	CHEMBL691	0.688940	32925.0	*
Pattern	CHEMBL282575	0.919105	21780.0	*
AP_bits	CHEMBL282575	0.538905	21780.0	*
TT_bits	CHEMBL282575	0.641026	21780.0	*
FP2	CHEMBL691	0.619048	32925.0	
hybridization	CHEMBL691	0.816327	32925.0	*
substructure	CHEMBL691	0.687500	32925.0	
graph	CHEMBL691	0.767442	32925.0	
pubchem	CHEMBL691	0.830645	32925.0	*
cdk_maccs	CHEMBL691	0.820513	32925.0	*
klekota_roth	CHEMBL691	0.777778	32925.0	*

*** ic50 ACTIVITY *** value prediction
based on 9 locally validated fgps ---> 27997.013
["TT_bits", "cdk_maccs", "Pattern", "MFP1", "klekota_roth", "pubchem", "AP_bits", "RDKit7", "hybridization"]

29 rank
Nuclear receptor subfamily 1 group I member 3 : Homo sapiens
score: 6.144 on CHEMBL5503 based on 8 fingerprints

Fingerprint type	Ligand	Tanimoto Similarity	activity	Valid fg
FeatMFP1	CHEMBL691	0.636364	3000.0	
MFP1	CHEMBL691	0.527778	3000.0	*
RDKit7	CHEMBL691	0.688940	3000.0	*
Pattern	CHEMBL691	0.891192	3000.0	*
AP_bits	CHEMBL691	0.465574	3000.0	
TT_bits	CHEMBL691	0.487805	3000.0	
FP2	CHEMBL691	0.619048	3000.0	
hybridization	CHEMBL691	0.816327	3000.0	*
substructure	CHEMBL2311171	0.714286	1200.0	
graph	CHEMBL2311171	0.790698	1200.0	*
pubchem	CHEMBL691	0.830645	3000.0	*
cdk_maccs	CHEMBL691	0.820513	3000.0	*
klekota_roth	CHEMBL691	0.777778	3000.0	*

*** ic50 ACTIVITY *** value prediction
based on 8 locally validated fgps ---> 2674.102

```
["cdk_maccs", "Pattern", "MFP1", "klekota_roth", "pubchem", "graph",
"RDKit7", "hybridization"]

30 rank
UDP-glucuronosyltransferase 1-1 : Homo sapiens
      score: 5.353 on CHEMBL1287617 based on 7 fingerprints
Fingerprint type     Ligand  Tanimoto Similarity  activity Valid fg
       FeatMFP1  CHEMBL691             0.636364   21000.0
           MFP1  CHEMBL691             0.527778   21000.0         *
         RDKit7  CHEMBL691             0.688940   21000.0         *
        Pattern  CHEMBL691             0.891192   21000.0         *
        AP_bits  CHEMBL691             0.465574   21000.0
        TT_bits  CHEMBL691             0.487805   21000.0
            FP2  CHEMBL691             0.619048   21000.0
  hybridization  CHEMBL691             0.816327   21000.0         *
   substructure  CHEMBL691             0.687500   21000.0
          graph  CHEMBL691             0.767442   21000.0
        pubchem  CHEMBL691             0.830645   21000.0         *
      cdk_maccs  CHEMBL691             0.820513   21000.0         *
   klekota_roth  CHEMBL691             0.777778   21000.0         *
      *** ic50 ACTIVITY *** value prediction
      based on 7 locally validated fgps ---> 21000.000
["Pattern", "MFP1", "pubchem", "klekota_roth", "hybridization", "RDKit7",
"cdk_maccs"]

31 rank
Solute carrier family 22 member 2 : Homo sapiens
      score: 5.353 on CHEMBL1743122 based on 7 fingerprints
Fingerprint type     Ligand  Tanimoto Similarity  activity Valid fg
       FeatMFP1  CHEMBL691             0.636364    2200.0
           MFP1  CHEMBL691             0.527778    2200.0         *
         RDKit7  CHEMBL691             0.688940    2200.0         *
        Pattern  CHEMBL691             0.891192    2200.0         *
        AP_bits  CHEMBL691             0.465574    2200.0
        TT_bits  CHEMBL691             0.487805    2200.0
            FP2  CHEMBL691             0.619048    2200.0
  hybridization  CHEMBL691             0.816327    2200.0         *
   substructure  CHEMBL691             0.687500    2200.0
          graph  CHEMBL103             0.767442   26700.0
        pubchem  CHEMBL691             0.830645    2200.0         *
      cdk_maccs  CHEMBL691             0.820513    2200.0         *
   klekota_roth  CHEMBL691             0.777778    2200.0         *
      *** ic50 ACTIVITY *** value prediction
      based on 7 locally validated fgps ---> 2200.000
["Pattern", "MFP1", "pubchem", "klekota_roth", "hybridization", "RDKit7",
"cdk_maccs"]

32 rank
Multidrug and toxin extrusion protein 1 : Homo sapiens
      score: 5.353 on CHEMBL1743126 based on 7 fingerprints
Fingerprint type     Ligand  Tanimoto Similarity  activity Valid fg
       FeatMFP1  CHEMBL691             0.636364   21100.0
           MFP1  CHEMBL691             0.527778   21100.0         *
         RDKit7  CHEMBL691             0.688940   21100.0         *
        Pattern  CHEMBL691             0.891192   21100.0         *
        AP_bits  CHEMBL691             0.465574   21100.0
        TT_bits  CHEMBL691             0.487805   21100.0
            FP2  CHEMBL691             0.619048   21100.0
  hybridization  CHEMBL691             0.816327   21100.0         *
   substructure  CHEMBL691             0.687500   21100.0
```

```
          graph  CHEMBL691             0.767442   21100.0
        pubchem  CHEMBL691             0.830645   21100.0        *
      cdk_maccs  CHEMBL691             0.820513   21100.0        *
   klekota_roth  CHEMBL691             0.777778   21100.0        *
      *** ic50 ACTIVITY *** value prediction
      based on 7 locally validated fgps ---> 21100.000
["Pattern", "MFP1", "pubchem", "klekota_roth", "hybridization", "RDKit7",
"cdk_maccs"]

33 rank
Multidrug and toxin extrusion protein 2 : Homo sapiens
      score: 5.353 on CHEMBL1743127 based on 7 fingerprints
Fingerprint type     Ligand  Tanimoto Similarity  activity Valid fg
       FeatMFP1  CHEMBL691             0.636364   20200.0
           MFP1  CHEMBL691             0.527778   20200.0        *
         RDKit7  CHEMBL691             0.688940   20200.0        *
        Pattern  CHEMBL691             0.891192   20200.0        *
        AP_bits  CHEMBL691             0.465574   20200.0
        TT_bits  CHEMBL691             0.487805   20200.0
            FP2  CHEMBL691             0.619048   20200.0
  hybridization  CHEMBL691             0.816327   20200.0        *
   substructure  CHEMBL691             0.687500   20200.0
          graph  CHEMBL691             0.767442   20200.0
        pubchem  CHEMBL691             0.830645   20200.0        *
      cdk_maccs  CHEMBL691             0.820513   20200.0        *
   klekota_roth  CHEMBL691             0.777778   20200.0        *
      *** ic50 ACTIVITY *** value prediction
      based on 7 locally validated fgps ---> 20200.000
["Pattern", "MFP1", "pubchem", "klekota_roth", "hybridization", "RDKit7",
"cdk_maccs"]

34 rank
Mu opioid receptor : Homo sapiens
      score: 5.353 on CHEMBL233 based on 7 fingerprints
Fingerprint type     Ligand  Tanimoto Similarity  activity Valid fg
       FeatMFP1  CHEMBL691             0.636364   26769.0
           MFP1  CHEMBL691             0.527778   26769.0        *
         RDKit7  CHEMBL691             0.688940   26769.0        *
        Pattern  CHEMBL691             0.891192   26769.0        *
        AP_bits  CHEMBL691             0.465574   26769.0
        TT_bits  CHEMBL691             0.487805   26769.0
            FP2  CHEMBL691             0.619048   26769.0
  hybridization  CHEMBL691             0.816327   26769.0        *
   substructure  CHEMBL691             0.687500   26769.0
          graph  CHEMBL691             0.767442   26769.0
        pubchem  CHEMBL691             0.830645   26769.0        *
      cdk_maccs  CHEMBL691             0.820513   26769.0        *
   klekota_roth  CHEMBL691             0.777778   26769.0        *
      *** ic50 ACTIVITY *** value prediction
      based on 7 locally validated fgps ---> 26769.000
["Pattern", "MFP1", "pubchem", "klekota_roth", "hybridization", "RDKit7",
"cdk_maccs"]

35 rank
Kappa opioid receptor : Homo sapiens
      score: 5.353 on CHEMBL237 based on 7 fingerprints
Fingerprint type       Ligand  Tanimoto Similarity  activity Valid fg
       FeatMFP1  CHEMBL68354             0.640000       3.8
           MFP1    CHEMBL691             0.527778   25894.0        *
         RDKit7    CHEMBL691             0.688940   25894.0        *
```

```
        Pattern    CHEMBL691            0.891192   25894.0        *
        AP_bits    CHEMBL691            0.465574   25894.0
        TT_bits    CHEMBL691            0.487805   25894.0
            FP2    CHEMBL691            0.619048   25894.0
  hybridization    CHEMBL691            0.816327   25894.0        *
   substructure    CHEMBL691            0.687500   25894.0
          graph    CHEMBL691            0.767442   25894.0
        pubchem    CHEMBL691            0.830645   25894.0        *
      cdk_maccs    CHEMBL691            0.820513   25894.0        *
   klekota_roth    CHEMBL691            0.777778   25894.0        *
      *** ic50 ACTIVITY *** value prediction
      based on 7 locally validated fgps ---> 25894.000
["Pattern", "MFP1", "pubchem", "klekota_roth", "hybridization", "RDKit7",
"cdk_maccs"]

36 rank
Adenosine A2a receptor : Homo sapiens
      score: 5.353 on CHEMBL251 based on 7 fingerprints
Fingerprint type     Ligand  Tanimoto Similarity  activity Valid fg
       FeatMFP1  CHEMBL691            0.636364   20931.0
           MFP1  CHEMBL691            0.527778   20931.0        *
         RDKit7  CHEMBL691            0.688940   20931.0        *
        Pattern  CHEMBL691            0.891192   20931.0        *
        AP_bits  CHEMBL691            0.465574   20931.0
        TT_bits  CHEMBL691            0.487805   20931.0
            FP2  CHEMBL691            0.619048   20931.0
  hybridization  CHEMBL691            0.816327   20931.0        *
   substructure  CHEMBL691            0.687500   20931.0
          graph  CHEMBL691            0.767442   20931.0
        pubchem  CHEMBL691            0.830645   20931.0        *
      cdk_maccs  CHEMBL691            0.820513   20931.0        *
   klekota_roth  CHEMBL691            0.777778   20931.0        *
      *** ic50 ACTIVITY *** value prediction
      based on 7 locally validated fgps ---> 20931.000
["Pattern", "MFP1", "pubchem", "klekota_roth", "hybridization", "RDKit7",
"cdk_maccs"]

37 rank
Serotonin 4 (5-HT4) receptor : Cavia porcellus
      score: 5.353 on CHEMBL5017 based on 7 fingerprints
Fingerprint type     Ligand  Tanimoto Similarity  activity Valid fg
       FeatMFP1  CHEMBL691            0.636364    7953.0
           MFP1  CHEMBL691            0.527778    7953.0        *
         RDKit7  CHEMBL691            0.688940    7953.0        *
        Pattern  CHEMBL691            0.891192    7953.0        *
        AP_bits  CHEMBL691            0.465574    7953.0
        TT_bits  CHEMBL691            0.487805    7953.0
            FP2  CHEMBL691            0.619048    7953.0
  hybridization  CHEMBL691            0.816327    7953.0        *
   substructure  CHEMBL691            0.687500    7953.0
          graph  CHEMBL691            0.767442    7953.0
        pubchem  CHEMBL691            0.830645    7953.0        *
      cdk_maccs  CHEMBL691            0.820513    7953.0        *
   klekota_roth  CHEMBL691            0.777778    7953.0        *
      *** ic50 ACTIVITY *** value prediction
      based on 7 locally validated fgps ---> 7953.000
["Pattern", "MFP1", "pubchem", "klekota_roth", "hybridization", "RDKit7",
"cdk_maccs"]

38 rank
```

Tubulin : Bos taurus

score: 5.303 on CHEMBL2111464 based on 7 fingerprints

Fingerprint type	Ligand	Tanimoto Similarity	activity	Valid fg
FeatMFP1	CHEMBL1627442	0.652174	660.0	
MFP1	CHEMBL299613	0.687500	1400.0	*
RDKit7	CHEMBL299613	0.669318	1400.0	
Pattern	CHEMBL1627442	0.885563	660.0	*
AP_bits	CHEMBL299613	0.553265	1400.0	*
TT_bits	CHEMBL299613	0.735294	1400.0	*
FP2	CHEMBL299613	0.597701	1400.0	
hybridization	CHEMBL299613	0.755639	1400.0	*
substructure	CHEMBL1627442	0.866667	660.0	*
graph	CHEMBL299613	0.614035	1400.0	
pubchem	CHEMBL299613	0.785185	1400.0	
cdk_maccs	CHEMBL299613	0.744186	1400.0	
klekota_roth	CHEMBL299613	0.819444	1400.0	*

*** ic50 ACTIVITY *** value prediction

based on 7 locally validated fgps ---> 1070.962

["TT_bits", "substructure", "Pattern", "MFP1", "AP_bits", "klekota_roth", "hybridization"]

39 rank

Cytochrome P450 3A4 : Homo sapiens

score: 5.240 on CHEMBL340 based on 7 fingerprints

Fingerprint type	Ligand	Tanimoto Similarity	activity	Valid fg
FeatMFP1	CHEMBL410242	0.625000	1500.0	
MFP1	CHEMBL410242	0.512195	1500.0	
RDKit7	CHEMBL410242	0.644468	1500.0	
Pattern	CHEMBL410242	0.910596	1500.0	*
AP_bits	CHEMBL410242	0.539945	1500.0	*
TT_bits	CHEMBL410242	0.511628	1500.0	*
FP2	CHEMBL410242	0.557895	1500.0	
hybridization	CHEMBL410242	0.825397	1500.0	*
substructure	CHEMBL410242	0.722222	1500.0	
graph	CHEMBL1451	0.895833	49100.0	*
pubchem	CHEMBL469227	0.834711	2770.0	*
cdk_maccs	CHEMBL539168	0.711111	3364.0	
klekota_roth	CHEMBL410242	0.721519	1500.0	*

*** ic50 ACTIVITY *** value prediction

based on 7 locally validated fgps ---> 6546.329

["TT_bits", "Pattern", "AP_bits", "pubchem", "graph", "klekota_roth", "hybridization"]

40 rank

Multidrug resistance-associated protein 4 : Homo sapiens

score: 4.945 on CHEMBL1743128 based on 7 fingerprints

Fingerprint type	Ligand	Tanimoto Similarity	activity	Valid fg
FeatMFP1	CHEMBL1697724	0.535714	30000.0	
MFP1	CHEMBL3349339	0.583333	50000.0	*
RDKit7	CHEMBL3349339	0.651366	50000.0	
Pattern	CHEMBL3349339	0.882960	50000.0	*
AP_bits	CHEMBL3349339	0.470238	50000.0	*
TT_bits	CHEMBL3349339	0.666667	50000.0	*
FP2	CHEMBL3349339	0.525253	50000.0	
hybridization	CHEMBL3349339	0.689420	50000.0	*
substructure	CHEMBL3349339	0.631579	50000.0	
graph	CHEMBL3349339	0.564516	50000.0	
pubchem	CHEMBL3349339	0.868852	50000.0	*
cdk_maccs	CHEMBL2074591	0.627451	80000.0	
klekota_roth	CHEMBL3349339	0.783784	50000.0	*

*** ic50 ACTIVITY *** value prediction
based on 7 locally validated fgps ---> 39849.623
["TT_bits", "Pattern", "MFP1", "pubchem", "AP_bits", "klekota_roth", "hybridization"]

41 rank
Protein-tyrosine phosphatase 2C : Homo sapiens
score: 4.881 on CHEMBL3864 based on 7 fingerprints

Fingerprint type	Ligand	Tanimoto Similarity	activity	Valid fg
FeatMFP1	CHEMBL461415	0.555556	2270.0	
MFP1	CHEMBL1642763	0.526316	19900.0	*
RDKit7	CHEMBL1642763	0.662971	19900.0	
Pattern	CHEMBL1642763	0.881443	19900.0	*
AP_bits	CHEMBL518627	0.475000	3910.0	*
TT_bits	CHEMBL1642763	0.600000	19900.0	*
FP2	CHEMBL1642763	0.576087	19900.0	
hybridization	CHEMBL1642763	0.742647	19900.0	*
substructure	CHEMBL1642763	0.578947	19900.0	
graph	CHEMBL230006	0.727273	9600.0	
pubchem	CHEMBL1642763	0.856000	19900.0	*
cdk_maccs	CHEMBL461199	0.704545	6400.0	
klekota_roth	CHEMBL1642763	0.800000	19900.0	*

*** ic50 ACTIVITY *** value prediction
based on 7 locally validated fgps ---> 16500.755
["TT_bits", "Pattern", "MFP1", "pubchem", "AP_bits", "klekota_roth", "hybridization"]

SwissTargetPrediction report:

Reference:
Gfeller D., Michielin O. & Zoete V.
Shaping the interaction landscape of bioactive molecules, *Bioinformatics* (2013) 29:3073-3079.

Query Molecule

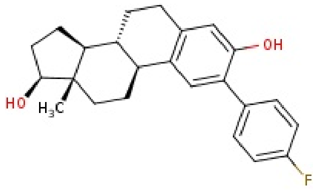

Frequency of Target Class

Target	Uniprot ID	Gene code	ChEMBL ID	Probability	# sim. cmpds (3D / 2D)	Target Class
cDNA FLJ16289 fis, clone OCBBF2009920, highly similar to TUBULIN BETA-3 CHAIN *(by homology)*	A8K854	TUBB3			3 / 12	Structural
Uncharacterized protein *(by homology)*	I3L2F9				3 / 12	Structural
Tubulin beta-4A chain *(by homology)*	P04350	TUBB4A	CHEMBL3838		3 / 12	Structural
Tubulin beta chain *(by homology)*	P07437	TUBB	CHEMBL5444		3 / 12	Structural
Tubulin beta-4B chain *(by homology)*	P68371	TUBB4B	CHEMBL1848		3 / 12	Structural
Melanocyte-stimulating hormone receptor *(by homology)*	Q01726	MC1R	CHEMBL3795		9 / 12	Membrane receptor
Tubulin beta-3 chain *(by homology)*	Q13509	TUBB3	CHEMBL2597		3 / 12	Structural
Tubulin beta-2A chain *(by homology)*	Q13885	TUBB2A			3 / 12	Structural
Tubulin beta-6 chain *(by homology)*	Q9BUF5	TUBB6			3 / 12	Structural
Tubulin beta-2B chain *(by homology)*	Q9BVA1	TUBB2B			3 / 12	Structural
Steryl-sulfatase	P08842	STS	CHEMBL3559		10 / 56	Enzyme
Arylsulfatase D *(by homology)*	P51689	ARSD			10 / 56	Enzyme
Arylsulfatase E *(by homology)*	P51690	ARSE			10 / 56	Enzyme
Arylsulfatase F *(by homology)*	P54793	ARSF			10 / 56	Enzyme
Arylsulfatase H *(by homology)*	Q5FYA8	ARSH			10 / 56	Enzyme

Polypharmacology Browser 2 Prediction:

Targets predicted using NN(ECfp4) + NB(ECfp4). Save Table

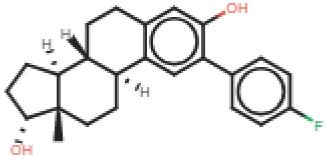

Query molecule

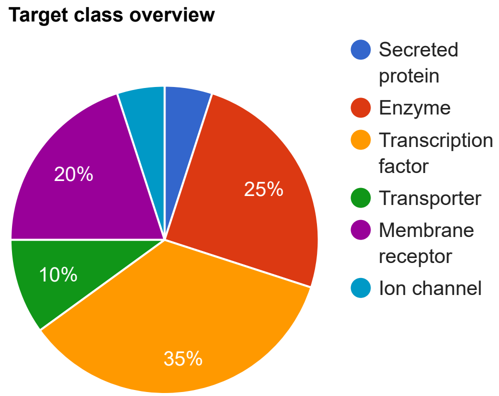

Rank	ChEMBL ID	Common name	Nearest neighbours
1	CHEMBL206 (https://www.ebi.ac.uk/chembl/target/inspect/CHEMBL206)	Estrogen receptor alpha	Show NN
2	CHEMBL242 (https://www.ebi.ac.uk/chembl/target/inspect/CHEMBL242)	Estrogen receptor beta	Show NN
3	CHEMBL3559 (https://www.ebi.ac.uk/chembl/target/inspect/CHEMBL3559)	Steryl-sulfatase	Show NN
4	CHEMBL3181 (https://www.ebi.ac.uk/chembl/target/inspect/CHEMBL3181)	Estradiol 17-beta-dehydrogenase 1	Show NN
5	CHEMBL3305 (https://www.ebi.ac.uk/chembl/target/inspect/CHEMBL3305)	Testis-specific androgen-binding protein	Show NN
6	CHEMBL228 (https://www.ebi.ac.uk/chembl/target/inspect/CHEMBL228)	Serotonin transporter	Show NN
7	CHEMBL1871 (https://www.ebi.ac.uk/chembl/target/inspect/CHEMBL1871)	Androgen Receptor	Show NN
8	CHEMBL1978 (https://www.ebi.ac.uk/chembl/target/inspect/CHEMBL1978)	Cytochrome P450 19A1	Show NN
9	CHEMBL2034 (https://www.ebi.ac.uk/chembl/target/inspect/CHEMBL2034)	Glucocorticoid receptor	Show NN
10	CHEMBL208 (https://www.ebi.ac.uk/chembl/target/inspect/CHEMBL208)	Progesterone receptor	Show NN
11	CHEMBL3072 (https://www.ebi.ac.uk/chembl/target/inspect/CHEMBL3072)	Androgen Receptor	Show NN
12	CHEMBL1994 (https://www.ebi.ac.uk/chembl/target/inspect/CHEMBL1994)	Mineralocorticoid receptor	Show NN
13	CHEMBL340 (https://www.ebi.ac.uk/chembl/target/inspect/CHEMBL340)	Cytochrome P450 3A4	Show NN
14	CHEMBL1781859 (https://www.ebi.ac.uk/chembl/target/inspect/CHEMBL1781859)	Solute carrier organic anion transporter family member 1A1	Show NN
15	CHEMBL3602 (https://www.ebi.ac.uk/chembl/target/inspect/CHEMBL3602)	Sigma opioid receptor	Show NN
16	CHEMBL339 (https://www.ebi.ac.uk/chembl/target/inspect/CHEMBL339)	Dopamine D2 receptor	Show NN
17	CHEMBL224 (https://www.ebi.ac.uk/chembl/target/inspect/CHEMBL224)	Serotonin 2a (5-HT2a) receptor	Show NN

18	CHEMBL217 (https://www.ebi.ac.uk/chembl/target/inspect/CHEMBL217)	Dopamine D2 receptor	Show NN
19	CHEMBL205 (https://www.ebi.ac.uk/chembl/target/inspect/CHEMBL205)	Carbonic anhydrase II	Show NN
20	CHEMBL240 (https://www.ebi.ac.uk/chembl/target/inspect/CHEMBL240)	HERG	Show NN
